# Multi-level strategies to improve equitable timely person-centred osteoarthritis care for diverse women: qualitative interviews with women and healthcare professionals

**DOI:** 10.1186/s12939-023-02026-x

**Published:** 2023-10-07

**Authors:** Anna R. Gagliardi, Angelina Abbaticchio, Madeline Theodorlis, Deborah Marshall, Crystal MacKay, Cornelia M. Borkhoff, Glen Stewart Hazlewood, Marisa Battistella, Aisha Lofters, Vandana Ahluwalia

**Affiliations:** 1 Toronto General Hospital Research Institute, University Health Network, 200 Elizabeth Street, 13EN-228, Toronto, ON M5G2C4 Canada; 2 University of Calgary, 2500 University Drive NW, Calgary Alberta T2N1N4, Canada; 3 West Park Healthcare Centre, 82 Buttonwood Ave, York, M6M2J5 Canada; 4 Child Health Evaluative Sciences, Research Institute, Hospital for Sick Children, Toronto, ON Canada; 5 Dalla Lana School of Public Health, University of Toronto, Toronto, ON Canada; 6 William Osler Health System, Brampton, ON Canada

**Keywords:** Osteoarthritis, Women’s health, Access, Person-centred care, Health care quality, Inequities, Barriers, Implementation strategies, Qualitative interviews

## Abstract

**Background:**

Women are more likely to develop osteoarthritis (OA), and have greater OA pain and disability compared with men, but are less likely to receive guideline-recommended management, particularly racialized women. OA care of diverse women, and strategies to improve the quality of their OA care is understudied. The purpose of this study was to explore strategies to overcome barriers of access to OA care for diverse women.

**Methods:**

We conducted qualitative interviews with key informants and used content analysis to identify themes regarding what constitutes person-centred OA care, barriers of OA care, and strategies to support equitable timely access to person-centred OA care.

**Results:**

We interviewed 27 women who varied by ethno-cultural group (e.g. African or Caribbean Black, Chinese, Filipino, Indian, Pakistani, Caucasian), age, region of Canada, level of education, location of OA and years with OA; and 31 healthcare professionals who varied by profession (e.g. family physician, nurse practitioner, community pharmacist, physio- and occupational therapists, chiropractors, healthcare executives, policy-makers), career stage, region of Canada and type of organization. Participants within and across groups largely agreed on approaches for person-centred OA care across six domains: foster a healing relationship, exchange information, address emotions, manage uncertainty, share decisions and enable self-management. Participants identified 22 barriers of access and 18 strategies to overcome barriers at the patient- (e.g. educational sessions and materials that accommodate cultural norms offered in different languages and formats for persons affected by OA), healthcare professional- (e.g. medical and continuing education on OA and on providing OA care tailored to intersectional factors) and system- (e.g. public health campaigns to raise awareness of OA, and how to prevent and manage it; self-referral to and public funding for therapy, greater number and ethno-cultural diversity of healthcare professionals, healthcare policies that address the needs of diverse women, dedicated inter-professional OA clinics, and a national strategy to coordinate OA care) levels.

**Conclusions:**

This research contributes to a gap in knowledge of how to optimize OA care for disadvantaged groups including diverse women. Ongoing efforts are needed to examine how best to implement these strategies, which will require multi-sector collaboration and must engage diverse women.

**Supplementary Information:**

The online version contains supplementary material available at 10.1186/s12939-023-02026-x.

## Background

Osteoarthritis (OA) is the most common type of arthritis affecting millions worldwide, more than doubling in prevalence between 1990 and 2019 from 247.51 to 527.81 million cases [1, 2]. There is no cure for OA and it often worsens over time. OA symptoms such as chronic joint pain and functional impairment restrict the activities of daily living, and can contribute to other chronic conditions such as depression, diabetes and heart disease [1, 2]. Hence, OA considerably impacts quality of life. With appropriate early management, the OA can be prevented or its progress delayed. According to clinical practice guidelines, first-line management of common forms of OA (e.g. hand, hip, knee) includes physical activity, heat and/or therapeutic cooling, acupuncture, braces/orthoses, pharmacologic and non-pharmacologic pain control, steroid injections, self-management programs and cognitive behavioral therapy [3]. Once OA progresses to the point where these options no longer ameliorate symptoms, second-line management options include surgery or joint replacement [3].

Women are disproportionately impacted by OA. Women are far more likely than men to develop hand, knee and hip OA, and experience greater OA severity, pain and disability compared with men [4]. However, women are less likely to receive first-line and second-line OA management, particularly racialized or immigrant women of colour with lower education or income [5–9]. Furthermore, racialized immigrant women have low rates of physical activity [10], an important way to prevent and manage OA, due to multiple interacting issues such as gender (primary role is to care for family), culture (not commonly practiced by women) and socioeconomic (little income for non-essential pursuits) constraints [11].

Research shows that numerous factors may influence access to care for persons with OA. For example, a systematic review of eight studies including 129 healthcare professionals in five countries revealed four barriers: they do not consider OA to be serious, lack knowledge of how to manage OA, possess personal beliefs at odds with guideline recommendations and feel that patients have unrealistic expectations [12]. A scoping review of 36 studies on barriers of OA care as perceived by healthcare professionals identified patient- (e.g. negative attitudes to lifestyle interventions), clinician- (e.g. lack of knowledge to promote behaviour change) and system- (little support for interdisciplinary collaboration) level barriers [13]. Interviews with 33 physical therapists similarly found that patient, clinician and healthcare system factors were barriers of OA care [14]. While it appears that complex, interacting, multi-level determinants influence access to and quality of OA care, these studies focused on clinician perspectives; none asked clinicians about OA care specifically for disadvantaged groups including women, and none directly consulted members of disadvantaged groups including diverse women about factors contributing to inequities in OA care. We lack information on the multi-level barriers to high-quality OA care faced by diverse women.

Greater knowledge is also needed regarding strategies (e.g. policy, programs, interventions, tools) that can address barriers and support high-quality OA care for disadvantaged groups including women. A scoping review identified only 10 studies published before 2010 on strategies to reduce inequities in OA care among various disadvantaged groups [15]. Strategies largely targeted individuals with OA through self-management education; few were aimed at the clinician or healthcare system level. In an updated review, we identified only 11 eligible studies published in 2010 or later, of which only two focused solely on women, and all strategies targeted persons with OA via joint replacement decision aids or self-management education [16]. Thus, insight is needed on multi-level strategies that address barriers of timely access to first-line management and improve the quality of OA care for diverse women.

Person-centred care (PCC) is a widely-advocated approach that has been associated with improved patient healthcare experiences and outcomes by tailoring healthcare interactions and treatment to individualized clinical needs and preferences [17]. International efforts based on systematic review and key informant consensus have generated insight on quality indicators of person-centred care specifically for those with OA such as address diverse patient characteristics, and OA symptoms or limitations; explore the impact of OA on patient lives; consider the time and financial burden of treatment, and patient preferences when care planning; and empower persons to identify outcomes important to them and support them in achieving those goals [18–21]. It is unknown if these or possibly other indicators of high-quality OA care are relevant to diverse women.

Given gendered inequities in OA care [5–9], a lack of prior research on barriers from the perspective of diverse women with OA [12–14], and the need for strategies to improve access to and quality of OA care for diverse women [15, 16], the overall aim of this study was to explore how to achieve person-centred OA care for diverse women. The specific objective was to interview diverse women with OA, as well as clinicians and health system managers about barriers of, and strategies that could support access to equitable timely person-centred OA care for diverse women.

## Methods

### Approach

We chose a qualitative research design that enabled us to thoroughly explore key informant views about barriers to care faced by diverse women with OA and recommended strategies to overcome those barriers and achieve equitable person-centred OA care among diverse women [22]. While there are many approaches in qualitative research, we employed qualitative description, which is commonly used in health services research to capture explicit details about expectations, preferences and suggestions for improving care [23]. We followed guidance to optimize rigor in conducting the study [24] and complied with the Consolidated Criteria for Reporting Qualitative Research [25]. The research team included a 13-member advisory group of diverse women with OA, and 13 healthcare professionals (family physician, rheumatologists, physiotherapist, pharmacist) and health services researchers with expertise in the topics of OA, person-centred care, equity and women’s health. The research team assisted with planning study design and data collection, and analyzing and interpreting findings. This study was approved by the Research Ethics Board at the University Health Network in Toronto, Canada. All participants provided written informed consent prior to participating in an interview. There was no prior relationship between the interviewers and participants.

### Sampling and recruitment

We used maximum variation sampling to recruit diverse women and healthcare professionals of differing specialties. Eligible women were aged 40 + with suspected or confirmed OA, able to understand and speak English language, and in addition to Caucasian women, represented the ethno-cultural immigrant groups most common to Canada: Chinese, Filipino, Indian, Pakistani, and African and Caribbean Black [26], who varied non-mutually exclusively in other attributes such as age and province in Canada. Eligible healthcare professionals included practicing clinicians most likely to interact with persons with OA for first-line care (family physicians, nurse practitioners, physical therapists, occupational therapists, chiropractors, and community pharmacists) and healthcare decision-makers (healthcare executives or managers, or government policy-makers) from across Canada. We aimed to interview 35 women (5 in each of the 7 ethno-cultural groups) and 35 professionals (approximately 5 of each specialty or profession) for a total of 70 participants. Sample size in qualitative research is determined concurrent with data collection and analysis to the point of theoretical saturation, which is often reached in qualitative research after 12 to 15 interviews [22, 27]. We recruited key informants between September 8, 2022 and December 9, 2022 through various organizations across Canada including advocacy groups, community agencies and professional societies who circulated a study information sheet to their constituents or members directing interested persons to contact the study coordinator. Additional file 1 provides a full description of our recruitment strategy. We also employed snowball sampling, whereby we asked members of our women advisory group and those participating in interviews to refer others to us.

### Data collection

We collected data by semi-structured telephone interviews from September 14, 2022 to December 15 2022. AA (woman, MPH-trained) conducted interviews with women and MT (woman, MPH-trained) conducted healthcare professional interviews. ARG (woman, PhD-trained with expertise and experience in qualitative research) trained and supervised AA and MT. Additional file 2 provides the interview questions. In general, questions focused on how to enable equitable timely person-centred first-line OA care and advice for diverse women. Questions about PCC were derived from an established PCC framework developed by McCormack et al. [28] that was further tailored by our prior work on what constitutes PCC for diverse women [29, 30]. Interview questions explored views and experiences of PCC, barriers affecting access to or quality of OA care among diverse women, and strategies to overcome those barriers and provide gender-equitable OA care. We explored barriers and strategies at the patient-, healthcare professional- and system-levels as was revealed in prior research [12–14]. For training purposes, ARG conducted the first woman and healthcare professional telephone interviews while AA and MT listened. Then ARG listened to one interview conducted by each of AA and MT, followed by discussion to optimize interview technique. Thereafter, AA and MT conducted all interviews, regularly consulting ARG for expert advice and feedback, and to establish the point of thematic saturation through review and discussion of themes. The mean interview length was 36 min (range 20 to 60 min). A transcriptionist transformed audio-recordings into verbatim transcripts.

### Data analysis

We used content analysis and constant comparison to inductively identify, expand or merge themes in transcripts of recorded interviews, and develop a codebook of themes and exemplar quotes, using Microsoft Office Word and Excel to manage data [22]. As a pilot test, AA, MT and ARG independently analyzed the first two transcripts from both women and healthcare professional interviews, then discussed coding technique and themes. AA and MT then coded an additional three interviews each for ARG review and feedback. Thereafter, AA and MT coded the remaining transcripts, periodically consulting with each other and with ARG to review and refine coding. We used summary statistics to describe participants and text to describe key themes. We tabulated and compared themes by group (women, clinicians, and decision-makers). Output included approaches that constitute person-centred care, and barriers of and strategies to enable equitable timely access to person-centred care for diverse women.

## Results

### Participant characteristics

Additional file 3 provides detailed characteristics of individual participants. Tables 1 and 2 summarize women and healthcare professional characteristics, respectively. We interviewed 27 women and 31 healthcare professionals. Women varied by ethno-cultural group, age, region of Canada, level of education, location of OA and years with OA. Healthcare professionals were largely women (26, 83.9%) working in healthcare delivery organizations (22, 71.0%) but varied by profession, career stage, region of Canada and type of organization.

**Table 1:** Characteristics of participating women

Characteristic	n (% of 27)
Ethno-cultural group	
African	2 (7.4)
Caribbean	2 (7.4)
European (White)	6 (22.2)
Chinese	5 (18.5)
Filipino	4 (14.8)
Indian	4 (14.8)
Pakistani	4 (14.8)
Age (years)	
40 to 59	10 (37.0)
60 to 69	11 (40.7)
70 +	5 (18.5)
Not reported^a^	1 (3.7)
Region of Canada	
Alberta	5 (18.5)
British Columbia	3 (11.1)
Manitoba	3 (11.1)
Nova Scotia	1 (3.7)
Ontario	14 (51.9)
Quebec	1 (3.7)
Education level	
Secondary	2 (7.4)
Post-secondary	12 (44.4)
Post-graduate	13 (48.1)
Location of OA	
Fingers only	1 (3.7)
Knee(s) only	12 (44.4)
Knee(s) and other	13 (48.1)
Neck, wrists and hands only	1 (3.7)
Years with OA	
< 10	17 (63.0)
10–19	3 (11.1)
20–29	6 (22.2)
30 +	1 (3.7)

^a^Preferred not to specify

**Table 2:** Characteristics of participating healthcare professionals

Characteristics	n (% of 31)
Profession	
Chiropractor	5 (16.1)
Healthcare executive	6 (19.4)
Family physician	1 (3.2)
Nurse practitioner	2 (6.5)
Occupational therapist	4 (12.9)
Pharmacist	3 (9.7)
Physiotherapist	7 (22.6)
Policy-maker	3 (9.7)
Gender	
Woman	26 (83.9)
Man	5 (16.1)
Career stage	
Early	4 (12.9)
Middle	8 (25.8)
Late	19 (61.3)
Region of Canada	
Ontario	15 (48.4)
British Columbia	7 (22.6)
Alberta	2 (6.5)
Manitoba	2 (6.5)
Newfoundland	2 (6.5)
Yukon Territory	2 (6.5)
Nova Scotia	1 (3.2)
Type of organization	
Charity	2 (6.5)
Commercial	3 (9.7)
Government	1 (3.2)
Health care	22 (71.0)
Quality improvement	2 (6.5)
Research	1 (3.2)

### Interview findings

Additional files 4 and 5 include all data from interviews with women and healthcare professionals, respectively. Themes with select quotes are discussed here, noting similarities and differences between women and healthcare professionals regarding what constitutes person-centred OA care for diverse women, barriers faced by diverse women in accessing OA care, and strategies that could improve access to equitable timely person-centred OA care for diverse women.

### Person-centred OA care

Table 3 summarizes key informant views about what constitutes person-centred OA care for diverse women organized by PCC domain. In this section, we describe and compare women, clinician, and healthcare executive/policy-maker participant views about existing or needed approaches to achieve six domains of person-centred OA care: foster a healing relationship, exchange information, address emotions, manage uncertainty, share decisions and enable self-management.

**Table 3:** Key informant views about person-centred OA care for diverse women

PCC domain (key themes)	Women	Clinicians	Executives/Policy-makers
Foster a healing relationship welcoming manner, eye contact, casual friendly conversation, active listening	[The doctor] asked me how are you feeling? How I can help you? Giving me the good smiling face. And then when I’m explaining, she’s listening carefully what I’m trying to tell her (21 woman Pakistani age 65) They were all polite to me…they welcomed me, and they did listen to my problems and I was happy about that (15 woman African age 45)	Building rapport by facing the person, having good eye contact, making sure that they hear and understand what I’m saying… (17 family physician late career) I usually talk about personal stuff…food and all that kind of stuff and I really get to know them or their family member. I like to ask about their day-to-day and what they do. I just try to get to know them on a human level (16 chiropractor early career)	I’m not aware of any proactive policies that help build relationships…I can’t think of a policy that either is specific to women or to marginalized groups (15 policymaker quality improvement late career)
Exchange information explore the impact of OA on daily life	He asked me what are my activities? Am I mostly staying at home just doing chores and not exercising (10 woman Filipino age 48) A lot of the questions involved the impact that the knee was having on my work..what is it that you want to do in your life and how is it being impacted (06 woman Caucasian age 65)	Sometimes people are more forthcoming with their goals of the treatment session or where they want to improve in their life, what sort of activities they want to get better at or resume or work towards. Sometimes it’s not evident and needs to be specifically asked (01 physiotherapist late career) You need to understand what their daily routine is like. What is it that you do, how do you spend your day? They’ll tell you if it’s assembly work, if they’re working in an office, if they’re sitting a lot of the time or if they’re at home looking after 8 children (25 pharmacist mid career)	I know there’s some work being done and greater attention being put onto how to meet the needs of a diverse audience. I think a lot of this probably still in its infancy…I can’t point to anything that I’m aware of that has would be a strong resource for women (31 executive late career)
Address emotions ask about and acknowledge the emotional impact of OA, and offer reassurance or referrals to other healthcare professionals	If I am feeling down because of my health problem, then [the doctor] talks to me and she would spend more time during my visit and talk to me about that…. And she kept on saying that if I am feeling down again, just make an appointment, she will talk to me again (23 woman Chinese age 70) She described about this is a chronic process and it will progress with the time. But with the management, healthy lifestyle and weight reduction, we can delay this process (22 woman Pakistani age 57)	I think we do this with everybody. We ask how they’re managing and what kind of support they have and what that impact is like (10 occupational therapist late career) Tell them it must be so hard and I understand how it’s affecting you so greatly. I can see that this is upsetting (08 occupational therapist late career) I have a counsellor who works in my clinic so usually I will refer to her if I noticed any sort of distress in regards to recently finding out about the diagnosis or having difficulty living with it (13 chiropractor early career)	A lot of primary care practitioners have the electronic medical record which have a lot of flow sheets [with] a lot of guidance documents in there… So if there are some concerns [with mental health], you can walk them through the flow sheet of what it is that you need to get addressed (19 healthcare executive late career) The OA tool under non-pharmacological self-management refers to mental health counsellor if available (15 policymaker quality-improvement late career)
Manage uncertainty Acknowledge uncertainty, offer encouragement, provide advice to prevent or delay worsening, and manage symptoms	She told me that since is a mild case, you got to look after yourself, and when you have any pain, use these medication or topical ointments (18 woman Indian age 69) They talked about how it can get worse. They give me solutions like exercises or physiotherapy services about how I can manage it better instead of concentrating on how much worse it can get (07 woman African age 43)	Managing expectations of treatment is part of it because there’s no cure to it. When it comes to non-pharmaceutical things, it’s only gonna work as well as the person’s gonna engage into it…so, highlighting that active participation in their treatment is critical for best outcomes (17 family physician late career) We usually tell people that OA is a mystery, we don’t have a crystal ball, we don’t know whether it’s going to affect a lot of their joints or maybe just one joint… Education is key…to being proactive in recognizing the symptoms and managing it with exercise or physio (25 pharmacist mid career)	---
Share decisions Describe merits of different options but allow patient to choose options they prefer	We talked about the various treatment options and the medications that I could choose and how I would adjust my lifestyle…He asked me about my preferences because I was willing to take medications and exercise but I refused the option of surgery (07 woman African age 43) What I like is that they didn’t impose because it’s up to me to do what I want. They [asked] me if I’m into physical fitness and I told them no. They didn’t give negative feedback on that. They said, if you’re considering exercising that would be good for your health (10 woman Filipino age 48)	I do like telling patients that I’m here to help them understand osteoarthritis and the treatment options and hopefully understand some of the pros and cons of what’s available to them so that they can make an informed decision (06 occupational therapist mid career) Any treatment is the patient’s decision. I can’t force anybody to do anything or take anything. And I never try. I use other patients as examples because I find that people respond to that if they know that somebody else did something and it helped (17 family physician late career)	I don’t know of what’s happening in practice but the policies that are being developed are patient- and family-centred, and there’s a greater push for healthcare providers to have a collaborative decision-making approach (14 government policymaker mid career)
Enable self-care Provide advice or resources to support self-management	He gave me a prescription to get a brace and he recommended that I get physio if that was something that I was able to access out of pocket (03 woman Caribbean age 48) If the pain is really severe [they said] I can take Tylenol or Advil. They said several regular exercises like 15-min walk everyday would be good (05 woman Filipino age 40)	I explain to them what the best practice recommendations are and I try to find ways to integrate it into whatever it is they do in their normal life; for example, there might be a Muslim woman who has to pray five times a day and she has to get down on her knees—try to integrate some sort of activity in that fashion (05 physiotherapist late career) We try and give people some tools that they can adopt to feel that they have some control over their arthritis or some pretty diverse toolbox that they can draw on in certain situations…We have some physical paper resources, we have some places that we can refer them to if they’re wanting to really delve into more some guided programs, a variety of some websites or hand-outs or us one-on-one guidance (06 occupational therapist mid career)	We also send every patient away, surgical or not, with a guide that we call “Living your Best Life with OA” It has general education about what OA is and it has managing OA (30 executive healthcare late career) We do these small group “Ask Anything” sessions for people who have come to at least one education session and maybe just want to know a bit about surgery. They want to know a bit about injections. They want to know a little bit about other options for them (24 executive late career)

Women and clinicians identified similar key components of PCC in each domain. For example, themes for foster a healing relationship included a welcoming manner, eye contact, casual friendly conversation and active listening, all used toward building rapport. The key theme for exchange information noted by both women and clinicians was to explore the impact of OA on daily life. To address emotions, women and clinicians said that it was important for clinicians to ask about and acknowledge the emotional impact of OA, and address feelings and concerns with information, reassurance or referrals to other healthcare professionals. Regarding the uncertainty of whether or how OA might progress, women and clinicians both emphasized managing expectations by explicitly discussing the uncertainty, but at the same time, providing encouragement of what women could proactively do to prevent progression or manage symptoms. In the domain of share decisions, participants of both groups highlighted the theme of choice among options for managing OA, with women wanting to know the range of options without any being imposed on them, and clinicians describing the pros and cons of options but letting patients decide how to proceed. A key theme articulated by women and clinicians in the domain of enable self-care was providing advice, information or resources about lifestyle behaviours and other options for preventing the progression and managing the symptoms of OA.

In addition to these themes, a few clinicians described how they tailor care to intersectional factors such as gender and ethno-cultural group such as ensuring privacy, offering a female healthcare professional and providing translated materials or an interpreter.

We always have a private space in terms of a closed door… If anybody has any diverse needs in terms of needing a female therapist or a male therapist, we try to make accommodations as needed (02 physiotherapist mid career)

We have translated sections on [our] website…We have educational materials translated into different languages to make it accessible (24 executive late career).

In contrast, healthcare executive or policy-maker participants had little insight on what constitutes person-centred OA care, and acknowledged that policies lacked guidance on person-centred OA care for diverse groups.

I can’t think of a policy that either is specific to women or to marginalized groups (15 policymaker quality improvement late career)

I think a lot of this probably still in its infancy…I can’t point to anything that I’m aware of that has would be a strong resource for women (31 executive late career)

Most women participants said that healthcare professionals did foster a healing relationship and engage in two-way exchange of information to understand patient needs and goals. However, women described a lack of person-centred OA care in other PCC domains. For example, many women said that healthcare professionals focused on prescribing a management option but did not address emotions associated with OA.

Unless you bring this to your doctor, she doesn’t ask those emotional or what you’re feeling questions (14 woman Chinese age 67)

There were no touchy, feely questions. I didn’t find a sympathetic or empathetic ear with the medical doctor (09 woman Caucasian age 70)

Regarding managing uncertainty, most women said that healthcare professionals advised them that OA is a natural part of ageing without providing advice on how to manage OA.

He said unfortunately this is the natural process and that it will get worse by the time of your age. I felt that the doctor mean that he can’t do anything because this is how the disease is (20 woman Pakistani age 44)

Most women said that healthcare professionals did not practice shared decision-making, where management options were discussed and they were invited to express preferences. Instead, they were given no options by healthcare professionals who told them that OA was a natural part of ageing, or limited options with no opportunity to discuss preferences.

I guess I’m frustrated because I don’t feel like I’m at the stage that needs a knee replacement…it seems kind of extreme for someone who can go up and down stairs, walk a few kilometres, skate and downhill ski. I feel like there should be something in the middle (06 woman Caucasian age 65)

For the domain of enable self-care, while some women participants were given information about how to manage symptoms or referred to other healthcare professionals, many were not.

My doctor can give me more information and teach any kind of exercise to help me to decrease the problem. But they don’t have time (16 woman Chinese age 62)

Doctor never give me any advice…just give me painkillers. If I got what kind of exercise, a diet plan, how to live day-by-day with pain, then it would be better and easier for me to live with arthritis (27 woman Indian age not reported)

### Barriers of OA care

Table 4 summarizes what key informants viewed as barriers of equitable timely person-centred OA care among diverse women. In this section, we describe and compare women, clinician, and healthcare executive/policy-maker participant views about barriers faced by diverse women in access to OA care, where barriers are categorized as patient, healthcare professional or healthcare system level.

**Table 4:** Key informant views about barriers of equitable timely person-centred OA care

Barrier level	Theme	Participant group		
		Women	Clinicians	Executives or Policy-makers
Patient Influenced by patient attributes, knowledge or behaviour, or experienced by patients	Women delay seeking care (unclear who to see, time constraints, other commitments)	x	x	x
	Cost—no health benefits for therapy or cannot afford to take time off work	x	x	x
	Language limitations prevent help-seeking and challenge communication	x	x	x
	No family doctor to manage OA or refer to others	x	x	x
	OA dismissed by clinicians due to age, not considered serious, particularly if women do not self-advocate/ask questions	x	x	x
	Challenge adopting new or unfamiliar activities such as exercise	x	x	x
	No or limited technology access or ability	x	x	---
Clinician Influenced by clinician attributes, knowledge or behaviour, or experienced by clinicians	Clinicians lack knowledge about OA	x	---	x
	Little or no interpreters or translated take-home material	x	---	---
	Short appointment time limits ability to develop rapport, and discuss concerns and management	---	x	x
	Lack of intersectional training, awareness or support	---	x	x
	Patients may not disclose personal information	---	x	---
	Ensuring women have a female clinician and/or private space	---	x	---
System Influenced by the way that health care is planned, organized, delivered, staffed and funded	Shortage of healthcare professionals or services prioritized for conditions of greater severity	x	x	x
	Long wait times for tests or referrals to specialists	x	x	x
	No coordinated system of interdisciplinary care	---	x	x
	Therapists have limited scope of practice	---	x	---
	Therapy services not publicly funded	---	x	---
	Lack of focus and funding for public health campaigns to prevent OA	---	x	x
	Lack of diversity among healthcare professionals	---	---	x
	Lack of policies or programs specific to women with OA	---	---	x

#### Patient level

At the patient level, 7 themes were very similar within and across participant groups, and included: women delay seeking care (unclear who to see, time constraints, other commitments prioritized over self-care), no health benefits to cover the cost of therapy or cannot afford time away from work, language limitations prevent help-seeking or challenge communication, not connected with a family doctor to manage OA or refer to others, OA dismissed by healthcare professionals, challenging to adopt new or unfamiliar activities such as exercise, and no or limited technology access or ability. The most commonly-articulated theme differed across groups, possibly reflecting different perspectives, experiences or priorities. For example, the theme mentioned most often by women participants was OA being dismissed by clinicians as an inevitable part of ageing with few options to prevent or manage it, particularly if women did not have the confidence or ability to self-advocate or ask questions.

If a person with ethnicity, color, comes to the family doctor and says I have osteoarthritis, do you think they will take care of you? No, they will just tell you that is very common when you grow older, it’s part of aging, there’s nothing much we can do… so nothing will be done until it becomes severe (26 woman Filipino age 67)

He said this is the problem you have now and you have to live with it. That was not appreciated. Definitely when we would get older, then we will have some problems, but I am 44, I don’t think I should go with this problem (20 woman Pakistani age 44)

In contrast, the barrier most often mentioned by clinicians was inability to pay for therapy or afford time away from work, and the barrier most often stated by executives/policy-makers was that women delay help-seeking due to time constraints and other commitments.

Monetary barriers. We’re not covered entirely by provincial healthcare here and not everyone has third-party insurance coverage (03 chiropractor early career)

Many women are caregivers to either kids and/or parents and/or both and they play that role and therefore it limits their ability to access appointments or get to appointments or put their health first (29 executive charity late career)

#### Healthcare professional level

At the healthcare professional level, participants identified 6 themes including: lack of knowledge about OA; lack of intersectional training, awareness or support; short appointment times limit ability to establish rapport and discuss OA concerns or management, patients may not disclose personal information, ensuring women have a female clinician and/or private space; and few interpreters or translated take-home material. However, there was little similarity within or across participant groups. For example, one woman and one executive/policy-maker said that clinicians lacked knowledge about OA, and one clinician mentioned ensuring women have a female clinician and/or private space.

In my experience and on their own admission, family physicians have virtually no competency in arthritis and in orthopaedics, and as my family physician of many years who’s now retired said to me, I think I had three hours of orthopaedics in medical school (11 woman Caucasian age 72)

Sometimes it would be best if it was a female, they might be more comfortable in terms of clothing and having dressing rooms so women feel comfortable because [they] might take certain clothing off to show certain parts of their body (05 physiotherapist late career)

The most frequently noted barriers by clinicians and executives/policy-makers were short appointment times and lack of knowledge or awareness of how to address intersectional factors such as gender and culture.

You’re dealing with people that have been practicing for a number of years and for them to even understand what it means to provide culturally sensitive care at the provider lens, I don’t think that is well understood. I think the new grads definitely get it. I think some of the non-physicians who have gone through additional training also get it (19 healthcare executive late career)

The length of your appointment visits are definitely a barrier to having these good comprehensive holistic conversations and assessment (23 nurse practitioner early career)

#### Healthcare system level 

At the healthcare system level, participants identified 8 themes including: shortage of healthcare professionals or services prioritized for conditions of greater severity, long wait times for tests or referrals to specialists, no coordinated system of interdisciplinary care, therapists have limited scope of practice, therapy services not publicly funded, lack of focus and funding for public health campaigns to raise awareness about OA, lack of diversity among healthcare professionals, and lack of policies or programs specific to women with OA. Only a few therapist participants referred to the barriers of lack of public funding for and limited scope of practice among therapists, and only a few executives/policy-makers noted the lack of intersectional diversity among healthcare professionals, and the lack of policies or programs specific to women with OA. Two themes similar across all three participant groups were shortage of healthcare professionals and long wait times.

It’s very hard to book an appointment here in our area. It’s overpopulated for a small town. You can’t get in immediately. I think we only have two chiropractors here and there are a lot of people here that experience back pain because most of us are working in the plant (05 woman Filipino age 40)

We have 1.6 OT’s for a huge geographic region and there’s just no way to not grow a wait list (06 occupational therapist mid career)

Two themes similar across clinicians and executives/manager participants were lack of a coordinated system of interdisciplinary OA care and lack of public health campaigns to raise awareness about OA.

For OA there’s not that OA expert, so there’s a gap in care where I don’t think anybody really is fully responsible for making sure people living with osteoarthritis get all the care that they need (31 executive charity late career)

Instead of putting the healthcare dollars into joint replacements, put healthcare dollars into educating people about pain and about healthy movement. Our healthcare is setup to be reactive, not proactive and that’s the problem with OA (08 occupational therapist late career)

### Strategies to improve OA care

Table 5 summarizes what key informants recommended as strategies to overcome barriers of, and support equitable timely person-centred OA care for diverse women. In this section, we describe and compare women, clinician, and healthcare executive/policy-maker participant views about strategies needed to improve OA care, where strategies are categorized as patient, healthcare professional or healthcare system level. Strategies included 5 patient, 4 healthcare professional and 9 system level approaches, respectively.

**Table 5:** Key informant views about strategies to improve equitable timely person-centred OA care for diverse women

Strategy	Patient	Clinician	Executive/Policy-Maker
Patient Level			
Offer education sessions about OA and self-management (women-only, group, in-person and virtual, multiple languages, across Canada, free, community centres)	We should have community awareness workshops or information sessions about arthritis (20 woman Pakistani age 44)	What would be wonderful to see is if group classes were offered in every province in different languages and to be small enough that… it can be tailored a little bit but not so small that we don’t service a lot of people at the same time (08 occupational therapist late career)	More education and information available out in the community. In churches or places of worship or places where they go to socialize (21 policymaker quality-improvement late career)
Offer educational material (brochures physicians can hand out, posters in community settings and online, in different languages, include culturally relevant information)	If there’s education material. Maybe a video or something like that in different languages that will be helpful, so that we have a better understanding. Maybe have a plan for what’s next like what would we expect let’s say in one year and 5 years or 10 years you know what should we be doing so it can help us cope with that better (17 woman Chinese age 66)	Patient tools in multiple languages that can at least give them an idea of the condition as well as medication…They should be available on one Canada-wide site (26 pharmacist mid career)	There definitely needs to be consideration for offering education materials in other languages (30 executive healthcare late career)
Consider patients’ cultural needs and economic circumstances when offering treatment, or self-care advice and/or programs (language, interpreters, cost of services such as physiotherapy)	If the doctor would feel that the patient does not understand what he’s talking about, he should request for an interpreter or else the patient won’t understand what the doctor is talking about (05 woman Filipino age 40)	I do know that aquafit is one of our biggest recommendations with OA. But I don’t necessarily think aquafit is culturally sensitive. I feel like there must be other forms of exercise like dance or something that would be equally beneficial and that diverse women might embrace more (12 physiotherapist late career)	---
Offer peer support groups to help with self-care (virtual and in-person, multiple languages)	Peer-to-peer support. Women who are struggling with arthritis and have found ways to get around it can share that with women who are just starting to have issues (08 woman Caucasian age 70)	---	---
Regular follow up from doctors to monitor OA symptoms and self-care	When there is a lot of information to absorb and you’re working against cultural and language barriers, patients need to have more than one visit (19 woman Chinese age 46)	Often when you see your nurse practitioner or physician again, it’s the first point of diagnosis and you don’t really know what to ask yet…either nurses or someone else that can ask questions or intentionally do a meaningful follow up is important to probe, how are things going? What’s working well? Do you have questions? (23 nurse practitioner early career)	---
Healthcare Professional Level			
Medical or continuing education on diagnosing and managing persons with OA	Maybe [doctors] need more education and training especially in arthritis. Because there is no treatment for arthritis and arthritis is very painful and it is very common (27 woman Indian age not reported)	Maybe some sort of continuing education. I think webinars are a good way to go especially now. It seems like a lot of the continuing education that I’m doing is virtual these days (13 chiropractor early career)	Starting from curriculum development all the way to training current providers and on-going training and some sort of performance management system where they demonstrate that understanding and they’re applying it (14 government policymaker mid career)
Education or training on providing person-centred OA care tailored for diverse persons	[Healthcare providers] should have cultural awareness training for them to understand different cultures especially for women (12 woman Filipino age 54)	Cultural training about different areas of the world and different… cultural or belief or approaches to different things (07 physiotherapist late career)	Educating the staff and people who come to access care to understand their needs better and provide the care from a very culturally appropriate place (14 government policymaker mid career)
Access to interpreters	---	I haven’t really found [help] for people who may not speak English… we have a lot of people who have immigrated from the Philippines, from India… I don’t really know anyone who speaks those languages (16 chiropractor early career)	If there is a language barrier, [there should be] some sort of interpretation services available to support the clinician and the patient so that they can communicate (21 policymaker quality-improvement late career)
OA assessment tools	---	Decision-making tools to say what’s your threshold if we’re referring to ortho, recommended initial assessment and follow up in 4 to 6 weeks or whatever, assess for falls risk if it’s a hip or a knee arthritis. All of us like things that are easy to follow and help provide consistent care (23 nurse practitioner early career)	---
Healthcare System Level			
Public health campaign to raise awareness of how to prevent and manage OA (available in different languages)	The government can possibly have health promotional activities on osteoarthritis…help people understand the symptoms and how to better manage it. I feel that information can be useful (07 woman African age 43)	Having things in clinic like posters or hand-outs goes a long way even to get people to recognize that their symptoms might be [OA] and that this is something you should talk to your doctor about and there’s things that can help (17 family physician late career)	A public education campaign… why don’t we try and get a clear message that early osteoarthritis doesn’t equal knee replacement which many people do believe, and trying to change the thinking (22 executive research late career)
Self-referral to therapy (for those without family physician)	---	I wonder if the concept of self-referral to a clinic or to an educational program would be an option to help improve accessibility because if they don’t have a family doctor…that’s a significant barrier (25 pharmacist mid career)	There probably needs to be mechanism to access care without a primary care provider (30 executive healthcare late career)
Dedicated inter-professional OA clinics	A specific arthritis clinic that you can go and have your questions answered or learn how to minimize your symptoms…including a physiotherapist, massage therapist, dietary, nutrition, exercise, kinesiology…it’s something I’m sure could be run by a nurse or nurse-practitioner (02 woman Caucasian age 72)	Our health care system is not set-up to be very accessible [for] clients on low income with no vehicle and limited access to public transportation. They’re faced with having appointments at all these different place. Could care be provided in more of a central, a one-stop? (06 occupational therapist mid career)	A lot of this kind of care…can be provided by other members of the team. Your nurse could do a lot of the counselling, if there’s medication, a pharmacist in-house could do a lot of the counselling related to that. Many teams have physiotherapists (19 healthcare executive late career)
Increase number of healthcare professionals for OA	If the government can provide more doctors in the towns like us where we are overpopulated then it would be better (05 woman Filipino age 40)	---	More primary care nurses, extended care nurses, nurse practitioners would be part of the solution. So people get knee pain and then they can talk to a nurse (22 executive research late career)
Increase diversity among healthcare professionals (gender, ethnicity)	More women nurses should be available just to increase the ability for expressing one-self from women to women (15 woman African age 45)	---	We need more diverse healthcare providers, physicians, nurses… even at the policy-level we need a more diverse representation (14 government policymaker mid career)
Publicly fund therapists	If they want to give relief for people suffering with arthritis we have to get free access to our physiotherapies (25 woman Pakistani age 60)	Having better health coverage for the people who are gonna work directly with the OA. A lot of the diverse women that I deal with are either recent immigrants or from lower socio-economic status and [not] having to worry about the financial burden of dealing with their health would make a big difference on them actually continuing with their treatment plans (03 chiropractor early career)	People will have different access to various supports. So trying to make sure we get that high quality care to everybody (31 executive charity late career)
Expand scope of practice of therapists, pharmacists	---	With some training and with presence of some more medical directives, we could streamline the patient into the right channel much better than what we are doing now… like blood work, for example, all my patients who present to me with some inflammatory presentations; if I’m able to send them for tests (04 physiotherapist late career)	Just give our therapist the autonomy to see who’s in front of them and if it is somebody who requires an extra visit or requires something that’s not part of necessarily a standard protocol that we do offer them the ability to do that (29 executive charity late career)
Develop healthcare policy to address the needs of diverse women	---	Prioritizing care for both women and diverse women. Recognizing that these patients may require more resources and or different types of resources and or the providers may need more training and sometimes even more time allocated for the assessments. I think it is just that recognition that these are patients in the system that aren’t getting equitable care (12 physiotherapist late career)	There needs to be an overarching equity and diversity and inclusion policy which also covers cultural competency, cultural appropriateness, cultural sensitivity and safety, which would then have more subsets of how to reach specific populations and specific healthcare settings (14 government policymaker mid career)
National OA strategy and standardized pathway for OA care	---	Canada and the provinces need a much better developed OA strategy and that has to cross from government level to primary care down to the first line clinicians… it needs to be a full drawn-out pathway for a client to follow (06 occupational therapist mid career)	There needs to be the digital and maybe physical infrastructure to support people getting the right tools, the right information, the right people. Some of it comes under funding but a lot of it comes down to coordination and getting all the different parts of the ecosystem to be connected (31 executive charity late career)

#### Patient level 

At the patient level, participants of all groups recommended education sessions and educational material offered at no cost in multiple languages in a variety of formats. In particular, only women emphasized that such information be provided as early as possible to prevent or delay OA progression.

Healthcare providers should not wait until you have to go to the orthopaedic specialist. They can let people know that this is what you have to do to prevent it. It will help the healthcare system in the long run. They won’t have to pay for all these joint replacements (Woman 24 Caribbean age 67)

Women and clinicians agreed on two additional themes. Both groups said that clinicians should consider cultural needs and economic circumstances when offering treatment or self-management advice; and clinicians should offer regular follow-up to monitor OA symptoms and self-care. Women participants also thought that peer support from others women would facilitate self-management.

#### Healthcare professional  level 

At the clinician level, participants of all three groups recommended medical and continuing education for clinicians on diagnosing and managing persons with OA, and on cultural awareness so that they could tailor care for diverse persons. Both clinician and executive/policy-maker participants underscored the need for interpreters to support patient-provider communication. Clinician participants also identified the need for OA assessment tools.

Decision-making tools to say what’s your threshold if we’re referring to ortho, recommended initial assessment and follow up in 4 to 6 weeks or whatever, assess for falls risk if it’s a hip or a knee arthritis. All of us like things that are easy to follow and help provide consistent care (23 nurse practitioner early career)

#### Healthcare system level

At the system level, many participants from all three groups emphasized the importance of public health campaigns delivered in different languages to raise awareness of how to prevent and manage OA. All three participant groups also recommended dedicated interprofessional OA clinics to provide holistic OA diagnosis and management, and public funding of physiotherapists, chiropractors and occupational therapists so that all persons affected by OA could access these supports.

People will have different access to various supports. So trying to make sure we get that high quality care to everybody (31 executive charity late career)

Women and executive/policy-maker participants agreed on two themes: increase the number of healthcare professionals for OA and increase the diversity of healthcare professionals.

More women nurses should be available just to increase the ability for expressing one-self from women to women (15 woman African age 45)

Clinician and executive/policy-maker participants articulated several similar themes: allow persons with OA to self-refer to therapy rather than requiring referral from a family physician, expand the scope of practice of therapists to include services such as ordering tests or referring to other specialists as a way to coordinate and streamline OA care, develop policies that address the broad range of healthcare needs of diverse women, and a national OA strategy with a standardized pathway for persons with OA.

There needs to be an overarching equity and diversity and inclusion policy which also covers cultural competency, cultural appropriateness, cultural sensitivity and safety, which would then have more subsets of how to reach specific populations and specific healthcare settings (14 government policymaker mid-career)

Canada and the provinces need a much better developed OA strategy and that has to cross from government level to primary care down to the first line clinicians…it needs to be a full drawn-out pathway for a client to follow (06 occupational therapist mid-career)

Executive/policy-maker participants also recommended that ongoing efforts to plan and implement strategies engage diverse women in the process.

Engaging with women so that there is a patient voice to the needs. Having a partnership. What is it that the patient can bring to the table to solve the problem? That type of partnership in planning is needed, not just the experience but the solutions (15 policymaker quality-improvement late career)

There’s a lot of work to be done about meaningful patient engagement…part of that could be engagement of diverse women in the program planning (19 healthcare executive late career)

In terms of assessing the cultural appropriateness of our teaching materials that, to me, would be bringing together groups of people to start to dialogue about our program and where there might be some room for improvement or consideration. I would love to do that (30 executive healthcare late career)

Bring in the different communities, the different people that you’re focused on, to try to understand where exactly do they get information, how do we connect with them in the most efficient way? (31 executive charity late career)

## Discussion

Interviews with 27 diverse women and 31 clinicians, healthcare executives and policy-makers from across Canada revealed insight on what constitutes and challenges person-centred OA care, and strategies needed to improve equitable timely access to person-centred OA care for diverse women. Despite concordant views between women and clinicians about what constitutes person-centred OA care, women said clinicians did little to address emotions, manage uncertainty, share decisions or enable self-care. Instead, clinicians told women that OA was a natural part of ageing and offered no or limited options to prevent further decline or manage symptoms. While executives and policy-makers had little insight, they acknowledged that policies lacked guidance on PCC for diverse persons. All three participant groups identified similar patient-level barriers of OA care but groups differed in the barrier most commonly mentioned: women – OA dismissed by clinicians; clinicians – inability to pay for therapy; and executives/policy-makers – women delay help-seeking due to multiple commitments that constrain time. Although participants identified 22 barriers of access, there was little similarity across groups in barriers at the healthcare professional (e.g. lack of OA knowledge or cultural training) and system (e.g. shortage of healthcare professionals) levels. Participants identified 5 patient-, 4 healthcare professional- and 9 system-level strategies needed to overcome and address those barriers.

These findings confirm and build on prior research. Earlier initiatives revealed that person-centred OA care included: address diverse patient characteristics, explore the impact of OA on patient lives; consider the time and financial burden of treatment, and patient preferences when care planning; and empower persons to identify outcomes important to them and support them in achieving those goals [18–21]. Our research confirmed that these aspects of PCC are relevant to diverse women and identified many additional approaches to person-centred OA organized across six PCC domains. MacKay et al. interviewed patients with early OA symptoms, who said they consulted with clinicians but received limited guidance to manage symptoms [31]. Similarly, among 354 persons with OA in Denmark, Norway, Portugal and the United Kingdom, the median proportion of quality indicators met in primary care consultations for knee OA was 48% (range 28% to 64%) [32]. Canadian participants of this study also reported that clinicians told them OA was a natural part of ageing and gave them few if any options to manage OA, but our study differs in its focus on diverse women, and on identifying barriers and strategies to improve access to ideal OA care. Other research identified multi-level barriers of access to and quality of OA care, but those studies largely consulted clinicians [12–14]. While our study confirmed many of the barriers identified in earlier work, we expanded on those barriers by consulting diverse women, and healthcare executives and policy-makers. As a result, we identified additional barriers particular to diverse women with OA. Previous syntheses identified a paucity of published research on strategies that improve access to and quality of OA care for disadvantaged groups, with very few studies focused on women, and all strategies targeting patients via joint replacement decision aids or self-management education [15, 16]. This study is unique in that it identified 18 multi-level strategies from the perspective of different types of key informants.

The findings suggest several implications. Many women participants noted that clinicians did not address emotions, manage uncertainty, share decisions or enable self-care, which has been documented in prior research [33, 34]. Instead, the main barrier they noted was that clinicians dismissed OA as an inevitable aspect of ageing. However, women identified approaches to achieving PCC in each of these domains that clinicians could adopt; for example, address emotions – acknowledge the emotional toll of OA and reassure them that options exist to manage symptoms; manage uncertainty – recognize the uncertainty of OA progression but offer encouragement and empower them with management options; share decisions – describe the range of management options, explore preferences and refrain from judging choices; and enable self-care – provide verbal and printed advice, and refer women to other sources of advice and OA management including specialists, programs and educational material. A scoping review of 30 studies of 2,876 patients’ perceived health information needs for OA found that patients wanted information in a variety of formats and from different sources on pharmacologic and non-pharmacologic OA management, and preferred information from healthcare professionals, printed educational materials, television and support groups over the Internet, which they regarded as unreliable [35]. Analysis of online information about knee OA treatment on 10 web sites in each of 10 countries (Brazil, China, France, Germany, India, Indonesia, Japan, Mexico, Russia, United States) found that quality and consistency of recommendations differed widely [36]. Thus, further research may be needed to assess the quality of online information for all forms of OA, and to develop high-quality resources that persons affected by OA can reliably use.

While clinicians also identified similar approaches for what constitutes person-centred OA care, they mentioned a lack of knowledge about OA and its management, and inability to establish rapport, exchange information, and discuss OA symptoms and management within short appointment times. Participants in all groups agreed that clinicians required medical and continuing education on OA, which can likely be developed through partnerships involving professional societies, licensing colleges, and arthritis charities or foundations. Clinician participants also said they would benefit from OA assessment tools, referring to resources such as surveys or checklists that could assist with diagnosis, assess risk of progression or outcomes and offer suggestions for optimal management. Available tools include performance-based tests of physical function (e.g. chair-stand or stair-climb tests) [37], or self-report questionnaires that assess pain and/or physical function [38], however, they largely focus on hip and/or knee OA, and it is unclear if they accommodate intersectional diversity. Furthermore, such tools do not assist clinicians in predicting OA progression or making decisions about optimal management. Further research is needed to identify and evaluate such tools if they exist, or alternatively, to develop such tools. Clinical practice guidelines represent another source of information to support professional practice. Our analysis of 36 guidelines on the overall management of OA published from 2003 to 2021 in 8 regions or countries found that few noted a greater prevalence of OA among particular groups such as women, acknowledged barriers of OA care among disadvantaged groups, or offered guidance on how to tailor OA care for diverse persons [39]. Developers could strengthen guidelines to better support clinicians in diagnosing and managing OA among diverse persons.

Given our focus on ethno-cultural diversity, this research highlighted that language limitations may prevent or help-seeking and challenge communication about OA management, clinicians may not have access to interpreters or translated educational material, women may not have access to a female healthcare professional, clinicians may not have a sufficiently private space for physical assessments, and women might find it difficult to adopt new or unfamiliar physical activities. Corresponding recommendations included women-only group educational sessions in community settings and educational material available in multiple languages that includes culturally-relevant OA management options. Future research to evaluate the quality of currently-available OA educational material should also evaluate whether it is available in multiple languages and culturally-acceptable. All participant groups recommended training for clinicians on how to foster culturally-safe care. In our prior research, content analysis of curriculum at medical schools across Canada found that students may not receive training in women's health or how to operationalize person-centred care [40, 41]. This suggests that medical curriculum could be updated to accommodate these concepts and emphasizes the importance to addresses these concepts in continuing education. Women participants who mentioned the barrier of being dismissed said this was particularly true for women lacking the ability or confidence to ask questions or self-advocate. In prior research, diverse women with various health issues said that a list of questions could help them to voice concerns [ARG REFS]. Simple one-page question prompt lists alone provided to patients in advance of appointments are proven to increase patient confidence to ask questions, and satisfaction with communication or care received; and reduce anxiety about health status or treatment [29]. Ongoing research is warranted to develop and evaluate the use and impact of an OA question prompt list.

System-level strategies recommended by participants of this study may be challenging to implement. For example, persons with OA may not be affiliated with a primary care clinician to provide OA advice or refer them to other healthcare professionals, and affected persons may not be able to afford therapy that could alleviate symptoms and prevent OA from worsening. These, and other barriers such as short appointment times, could be addressed with dedicated one-stop inter-professional OA clinics or centres. It is not known if such organizations exist, but future research could synthesize published research or perform an observational study of inter-professional OA care models. Ongoing efforts are also needed to plan how to design and implement other participant recommendations including policies specific to diverse women with OA, public health campaigns to raise awareness of OA, and how to prevent and manage it, publicly-funded therapy services, and a national strategy to coordinate OA management. Such efforts are likely to be collaborative, and as recommended by executive/policy-maker participants, must engage diverse women.

Strengths of this research include the use of rigorous qualitative methods that complied with qualitative research reporting standards [22–25, 27], input and guidance from an interdisciplinary research team that included healthcare professionals and diverse women with OA, inclusion of interview participants with diverse characteristics and perspectives, use of an existing PCC framework to organize results [28], thematic saturation of the findings, and concordance within and among participant groups on what constitutes person-centred OA care and strategies needed to overcome barriers. Some limitations should also be noted. Despite considerable extensive recruitment efforts (Additional file 1), women participants largely had high levels of education; and healthcare professionals were largely women and included few primary care clinicians. This research took place during the COVID-19 pandemic, which may have affected willingness to take part in research. Future research may be required to consult with ethno-culturally diverse women of lower socioeconomic status and primary care clinicians. This research was conducted in Canada, so the results may not be relevant to other jurisdictions with differing cultures and healthcare systems.

## Conclusions

We aimed to explore how to improve equitable timely access person-centred OA care for diverse women with OA. Interviews with 27 diverse women and 31 clinicians, healthcare executives and policy-makers from across Canada revealed numerous approaches to person-centred OA care, 22 barriers of access, and 18 multi-level strategies needed to improve access. Key strategies for diverse women included educational sessions and materials that accommodate cultural norms offered in different languages and formats for persons affected by OA; and for clinicians included medical and continuing education on OA and on providing OA care tailored to intersectional factors. Participants recommended numerous system-level strategies: public health campaigns to raise awareness of OA, and how to prevent and manage it; self-referral to and public funding for therapy (e.g. physiotherapy), greater number and ethno-cultural diversity of healthcare professionals, healthcare policies that address the needs of diverse women, dedicated inter-professional OA clinics, and a national strategy to coordinate OA care. Ongoing efforts are needed to examine how best to implement these strategies, which will require multi-sector collaboration and must engage diverse women.

### Supplementary Information

**Additional file 1. **Recruitment strategies.

**Additional file 2. **Interview guide.

**Additional file 3. **Participant characteristics.

**Additional file 4. **Themes and quotes – women.

**Additional file 5. **Themes and quotes – healthcare professionals.

## Data Availability

All data generated or analysed during this study are included in this published article and its supplementary information files.

## Abbreviations

OA: Osteoarthritis
PCC: Person-centred care
