# Supplementary material for: Multi-level strategies to improve equitable timely person-centred osteoarthritis care for diverse women: qualitative interviews with women and healthcare professionals
Source: Int J Equity Health. 2023 Oct 7;22:207. doi: 10.1186/s12939-023-02026-x (PMC10559457; doi:10.1186/s12939-023-02026-x)
Supplement: Supplementary file 1 — Additional file 1. Recruitment strategies. [file 12939_2023_2026_MOESM1_ESM.docx]

**Additional File 1. Strategies used to recruit key informants**

**Women**

| **Source** | **Examples** | **Number contacted** | **Number agreed to help recruit** | **Strategies employed by those who agreed to help** |
| --- | --- | --- | --- | --- |
| Research team co-investigators and collaborators (healthcare professionals and health services researchers) | - Canadian Arthritis Patient Alliance - Arthritis Consumer Experts - Schroeder Arthritis Institute - Bone and Joint Canada | 4 | 4 | Referral – shared recruitment script or poster via member newsletter, e-blast or social media post |
| Snowball sampling – previous participants asked to share study recruitment message with their network | N/A | 20 | 16 | Referral/word of mouth |
| 13-member women advisory panel | N/A | 13 | 13 | Referral/word of mouth |
| Community agencies or centres across Canada with a focus on supporting immigrant populations and/or women | - South Asian Women’s Centre - Filipino Centre Toronto - Immigrant Women Services Ottawa - Brooks and County Immigration Services (BCIS) - COSTI Immigrant Services - Neepawa and Area Immigrant Settlement Services - DIVERSEcity - YMCA | 108 | 30 | Referral – shared recruitment request with internal and external stakeholders via member newsletter, e-blast, social media post, WhatsApp |
| Community health clinics across Canada (non-profit organizations focused on delivering primary health care and health promotion in community settings) | - All relevant centres registered with the Canadian Association of Community Health Centres - South-Asian Health Institute (SAHI) at Fraser Health | 122 | 5 | Distributed poster via email, social media, or posted on walls of facility |
| Arthritis and self-management related organizations across Canada | - Arthritis Society - Arthritis Research Canada (Patient Advisory Board) - Self-Management BC | 28 | 10 | Shared recruitment script or poster with members via email or during group session, and posted recruitment message on website |
| Fitness centres across Canada | - Goodlife Fitness centres | 83 | 0 | --- |
| Student university cultural societies/clubs across Canada | - African Students Associations - Black Students Associations - Chinese Students Associations - Filipino Students Associations - Pakistani Students Associations - Indian Students Associations | 84 | 11 | Shared recruitment script or poster via member newsletter, e-blast or social media post |
| Physiotherapy or chiropractor clinics across Canada | - Centres registered as a Publicly-Funded Physiotherapy (Ontario) - Centre identified on Google using key terms like “physiotherapy clinics” and “<province or city name>” | 297 | 12 | Referral/word of mouth – shared recruitment script or poster directly with patients |
| Total | --- | 759 | 101 | --- |

**Healthcare professionals**

| **Source** | **Examples** | **Number contacted** | **Number agreed to help recruit and/or participate** | **Strategies employed by those who agreed to help** |
| --- | --- | --- | --- | --- |
| Research team co-investigators and collaborators (healthcare professionals and health services researchers) | - Women’s College Hospital - Neighborhood Pharmacy Association of Canada | 8 | 7 | Referral – shared recruitment script or poster via member newsletter, e-blast or social media po st |
| Snowball sampling – participants of prior studies asked to share recruitment message with their network | N/A | 20 | 1 | Referral/word of mouth |
| Arthritis-related organizations across Canada, government policy organizations, researchers or individuals in the arthritis field who may be connected to potential participants in their network | - Arthritis Rehabilitation and Education Program, Arthritis Society - Arthritis Research Canada - Alberta Bone & Joint Strategic Clinical Network - Ontario Ministry of Health - Health Quality Ontario - University of Ottawa | 56 | 11 | Shared recruitment script or poster with colleagues via email, directed recruitment request to others in organization, and met with research team to suggest recruitment avenues |
| Professional colleges and associations | - Nurse Practitioner Association of Ontario - Manitoba Chiropractors Association - Society of Rural Physicians of Canada - College of Physicians and Surgeons of Ontario | 76 | 22 | Shared recruitment script or poster with members via email, newsletter, e-bulletin, and posted recruitment message on website |
| Family health teams and health clinics across Canada | - Thunder Bay Regional Health Sciences Centre - London Middlesex Primary Care Alliance - Comox Valley Vision of Family Practice - City of Lakes Family Health Team | 40 | 7 | Referral/word of mouth – shared recruitment script or poster with members via email, newsletter, e-bulletin |
| Mass recruitment emails to clinicians and professionals whose contact information was obtained from publicly available databases and directories | - College of Physicians and Surgeons of Ontario - Canadian university family medicine department faculty lists (e.g. McMaster University Department of Family Medicine) - Sunnybrook Health Sciences Centre - Alberta College of Pharmacy - Government of Saskatchewan | 516 | 11 | Research team reached out to clinicians and professionals via email (individually and mass email) |
| Total | --- | 716 | 59 | --- |
