# Supplementary material for: Multi-level strategies to improve equitable timely person-centred osteoarthritis care for diverse women: qualitative interviews with women and healthcare professionals
Source: Int J Equity Health. 2023 Oct 7;22:207. doi: 10.1186/s12939-023-02026-x (PMC10559457; doi:10.1186/s12939-023-02026-x)
Supplement: Supplementary file 2 — Additional file 2. Interview guide. [file 12939_2023_2026_MOESM2_ESM.docx]

**Additional File 2. Interview guide**

| Component | Women | Clinicians | Executives/managers/policy-makers |
| --- | --- | --- | --- |
| Person-centred care (general)  My first question is about offering OA care and advice that is specific to the needs and preferences of diverse women. | Think about your visits with doctors, nurses or other healthcare providers related to OA symptoms or care.   1. What WAS good about these visits or your OA care? (PROMPT: what did they say or do that helped you understand OA and know how to manage symptoms?) 2. What was NOT good about these visits or your OA care? | How do you tailor discussions about OA, or offer treatment or self-care advice that is specific to diverse women? | What constitutes person-centred OA care for diverse women? |
| Person-centred care (specific)  Next I will ask a few questions to more fully explore how to personalize OA care and advice for diverse women | Foster healing relationship  What did your healthcare provider do or say to welcome you before they asked about your health problem?  Exchange information  When talking with you about OA and how to manage it, how well did healthcare providers understand your OA symptoms and its impact on your life?  Address emotions/concerns  How well did healthcare providers learn if you were feeling upset or stressed out about OA and its impact on your life?  Manage uncertainty  How well did healthcare providers explain the chances that OA might not improve or get worse, or how well different treatments might work?  Share decisions  How well did healthcare providers involve you in talking about or choosing different options for treating or managing OA?  Enable self-care  How did healthcare providers prepare you for next steps for your OA care? | Foster healing relationship  What do you do to get to know diverse women before talking about their health problem?  Exchange information  How do you address the unique needs and circumstances of diverse women when providing OA information, or advice about OA treatment or self-care?  Address emotions/concerns  How do you assess and address diverse women’s feelings or emotions about OA and its impact on their lives?  Manage uncertainty  How do you explain to diverse women the uncertainty of OA progression or how well different forms of treatment or self-care might improve their OA symptoms?  Share decisions  How do you prepare and involve diverse women to discuss or make decisions about first-line OA treatment or management?  Enable self-care  How do you prepare diverse women for next steps in their OA treatment or self-care? | In what way do current strategies support equitable access to person-centred OA care and advice for diverse women? [Prompt for six PCC domains]  How did/does your organization do that? |
| Barriers of access/quality  My next set of questions are about access and barriers to OA care for diverse women | Patient  Have you faced any barriers to getting OA care or advice? Please explain  Clinician  What barriers do you think healthcare providers face in providing OA care or advice?  System  What other barriers might prevent you from getting OA care and advice? | What barriers do you face in providing first-line OA care or advice to diverse women?  What other barriers might limit diverse women’s access to OA care and advice? [Prompt for patient or healthcare system factors] | What do you think are the barriers faced by diverse women in accessing early diagnosis and management of OA? [Prompt for patient, clinician and healthcare system barriers] |
| Recommended strategies  My last set of questions are about strategies needed to improve access to and person-centred tailored OA care for diverse women. What should be done so that women of different cultural groups, age, education, or income across Canada get the OA care and advice they need? | Patient  What kinds of information, advice or supports would help you manage OA? In what ways would you prefer to receive OA information or advice?  Clinician  What do doctors, nurses or other health care providers need to be able to help diverse women get the OAcare and advice they need?  System  What should be done by government or healthcare organizations to improve diverse women’s access to OA care and advice? | Clinician  What strategies would help you to provide OA care and advice to diverse women?  What strategies might benefit other types of clinicians or professionals that offer support or services to patients with OA?  Patient  What could help patients to access, understand and comply with OA first-line treatment and advice?  System  What healthcare system strategies or policies are needed to improve access to and person-centred OA care for diverse women? | What strategies are needed to ensure that diverse women access the OA care and advice they need? [Prompt for patient, clinician and healthcare system strategies]  What infrastructure, resources or approaches are needed to prompt, incentivize, or implement those strategies? |
