# Supplementary material for: Multi-level strategies to improve equitable timely person-centred osteoarthritis care for diverse women: qualitative interviews with women and healthcare professionals
Source: Int J Equity Health. 2023 Oct 7;22:207. doi: 10.1186/s12939-023-02026-x (PMC10559457; doi:10.1186/s12939-023-02026-x)
Supplement: Supplementary file 3 — Additional file 3. Participant characteristics. [file 12939_2023_2026_MOESM3_ESM.docx]

**Additional File 3. Characteristics of included participants**

**Women**

| **ID** | **Age** | **Years in Canada** | **Ethno-cultural group** | **City/Province** | **OA location** | **Years with OA** | **Education** |
| --- | --- | --- | --- | --- | --- | --- | --- |
| 01 | 69 | Born in Canada | Caucasian | Toronto, ON | Shoulder, knees | 8 | Post-graduate |
| 02 | 72 | Born in Canada | Caucasian | Halifax, NS | Knee, foot, shoulder, fingers | 25 | Post-graduate |
| 03 | 48 | 10+ | Black, Caribbean | Mississauga, ON | Knee | 7 | Post-graduate |
| 04 | 55 | 4 | South Asian, Indian | George Town, ON | Knees, fingers | 6 | Post-graduate |
| 05 | 40 | 5 | East Asian, Filipino | Neepawa, MB | Knees, wrists, neck | 3 | Bachelors |
| 06 | 65 | Born in Canada | Caucasian | Toronto, ON | Knees, hip, shoulder, fingers | 25 | Post-graduate |
| 07 | 43 | 12 | Black, African | Toronto, ON | Knees | 5 | High school |
| 08 | 70 | Born in Canada | Caucasian | Ottawa, ON | Hands, hip, knees | 25 | Bachelors |
| 09 | 70 | Born in Canada | Caucasian | Toronto, ON | Hand, knee | 20 | Post-graduate |
| 10 | 48 | 5 | East Asian, Filipino | Neepawa, MB | Knee | 3 | Bachelors |
| 11 | 72 | Born in Canada | Caucasian | Toronto, ON | Hips, knees, neck, spine, hands | 23 | Post-graduate |
| 12 | 54 | 12 | East Asian, Filipino | Winnipeg, MB | Knee | 1 | Bachelors |
| 13 | 67 | 45 | South Asian, Indian | Surrey, BC | Knees | 2 | Bachelors |
| 14 | 67 | 25 | East Asian, Chinese | Toronto, ON | Knees | 3 | College |
| 15 | 45 | Born in Canada | Black, African | Vancouver, BC | Knees | 3 | Bachelors |
| 16 | 62 | 32 | East Asian, Chinese | Richmond hill, ON | Knees, back, finger | 2 | College |
| 17 | 66 | 40 | East Asian, Chinese | Markham, ON | Knees | 4 | Post-graduate |
| 18 | 69 | 20+ | South Asian, Indian | Nanaimo, BC | Knees | 2 | Post-graduate |
| 19 | 46 | Born in Canada | East Asian, Chinese | Toronto, ON | Neck, wrists, hands | 15 | Post-graduate |
| 20 | 44 | 15 | South Asian, Pakistani | Calgary, AB | Knees | <1 | Post-graduate |
| 21 | 65 | 26 | South Asian, Pakistani | Calgary, AB | Hands, knees, back | 2 | College |
| 22 | 57 | 25 | South Asian, Pakistani | Toronto, ON | Knees | 2 | Post-graduate |
| 23 | 70 | 48 | East Asian, Chinese | Edmonton, AB | knees, shoulder, fingers, foot | 10 | High school |
| 24 | 67 | 51 | Black, Caribbean | Quebec City, QC | Knees | 49 | Bachelors |
| 25 | 60 | 12 | South Asian, Pakistani | Calgary, AB | Knees and many other joints | 20 | Post-graduate |
| 26 | 67 | 30 | East Asian, Filipino | Edmonton, AB | Fingers | 5 | Bachelors |
| 27 | * | 30 | South Asian, Indian | Toronto, ON | Shoulder, knees, hands, fingers | 15 | College |

* preferred not to specify

**Healthcare professionals**

| **ID** | **Profession** | **Gender** | **Province/City** | **Academic status** | **Organization type** | **Years in role** | **Career stage** |
| --- | --- | --- | --- | --- | --- | --- | --- |
| 01 | Physiotherapist | Woman | Parksville, BC | Community | Health care | 42 | Late |
| 02 | Physiotherapist | Woman | Toronto, ON | Academic | Health care | 13 | Middle |
| 03 | Chiropractor | Woman | Selkirk, MB | Community | Health care | 5 | Early |
| 04 | Physiotherapist | Woman | Brampton, ON | Community | Health care | 25 | Late |
| 05 | Physiotherapist | Man | Ottawa, ON | Community | Health care | 16 | Late |
| 06 | Occupational Therapist | Woman | Vancouver, BC | Community | Health care | 15 | Middle |
| 07 | Physiotherapist | Woman | Ottawa, ON | Community | Health care | 23 | Late |
| 08 | Occupational Therapist | Woman | Penticton, BC | Community | Health care | 17 | Late |
| 09 | Physiotherapist | Woman | Pickering, ON | Community | Health care | 35 | Late |
| 10 | Occupational Therapist | Woman | Vancouver, BC | Community | Health care | 29 | Late |
| 11 | Chiropractor | Man | Winnipeg, MB | Community | Health care | 57 | Late |
| 12 | Physiotherapist | Woman | Toronto, ON | Academic | Health care | 22 | Late |
| 13 | Chiropractor | Woman | Mount Pearl, NL | Community | Health care | 5 | Early |
| 14 | Policy maker | Woman | Halifax, NS | N/A | Government | 13 | Middle |
| 15 | Policy maker | Woman | Toronto, ON | N/A | Quality improvement | 30 | Late |
| 16 | Chiropractor | Woman | Whitehorse, YK | Community | Health care | 2 | Early |
| 17 | Family Physician | Woman | St. John's, NL | Community | Health care | 6 | Middle |
| 18 | Occupational Therapist | Woman | Victoria, BC | Community | Health care | 30 | Late |
| 19 | Executive | Woman | Toronto, ON | N/A | Health care | 17 | Late |
| 20 | Chiropractor | Woman | Whitehorse, YK | Community | Health care | 13 | Middle |
| 21 | Policy maker | Woman | Toronto, ON | N/A | Quality improvement | 32 | Late |
| 22 | Executive | Man | Vancouver, BC | N/A | Research | 22 | Late |
| 23 | Nurse Practitioner | Woman | Toronto, ON | Academic | Health care | 2 | Early |
| 24 | Executive | Woman | Vancouver, BC | N/A | Health care | 21 | Late |
| 25 | Pharmacist | Woman | Woman | Community | Commercial | 15 | Middle |
| 26 | Pharmacist | Man | Lacombe, AB | Community | Commercial | 6 | Middle |
| 27 | Pharmacist | Woman | Newmarket, ON | Community | Commercial | 27 | Late |
| 28 | Nurse Practitioner | Woman | Toronto, ON | Academic | Health care | 8 | Middle |
| 29 | Executive | Man | Toronto, ON | N/A | Charity | 22 | Late |
| 30 | Executive | Woman | Calgary, AB | N/A | Health care | 17 | Late |
| 31 | Executive | Woman | Toronto, ON | N/A | Charity | 15 | Late |
