# Supplementary material for: Multi-level strategies to improve equitable timely person-centred osteoarthritis care for diverse women: qualitative interviews with women and healthcare professionals
Source: Int J Equity Health. 2023 Oct 7;22:207. doi: 10.1186/s12939-023-02026-x (PMC10559457; doi:10.1186/s12939-023-02026-x)
Supplement: Supplementary file 4 — Additional file 4. Themes and quotes – women. [file 12939_2023_2026_MOESM4_ESM.docx]

**Additional File 4. Themes and quotes from interviews with women**

Person-centred OA care

*To answer my first questions, think about your visits with doctors or nurses or other healthcare providers about OA. What was good about your osteoarthritis care? What was not so good about your osteoarthritis care?*

| Theme | Black | East Asian | South Asian | Caucasian |
| --- | --- | --- | --- | --- |
| **Foster a healing relationship**  Extend friendly greeting, make eye contact, speak in respectful manner, avoid judgmental attitude  *Q: What does your healthcare provider do or say to welcome you before they ask about your health problem?* | GOOD  ***Created environment conducive to speaking***  The environment was just between me and [my doctor]. It was a private space and calm environment. It created a space to open up and talk to someone… I felt comfortable to speak out because there was good rapport between me and him (Woman 07 African age 43).  ***Healthcare providers listen***  [Healthcare providers] were all polite to me when they received me, they welcomed me, and they did listen to my problems and I was happy about that (Woman 15 African age 45).  ***Healthcare providers are welcoming***  I had [no healthcare providers] that had made me feel uncomfortable that I would have sensed… they welcomed me (Woman 24 Caribbean age 67).  POOR  -- | GOOD  ***Tried to speak the same language***  The effort of my latest chiropractor made me feel comfortable…he even tried to speak some Filipino words to make me feel comfortable (Woman 05 Filipino age 40).  ***Good relationship fostered over time***  We don’t need to welcome. The family doctor so many years we know each other (Woman 16 Chinese age 62).  ***Doctor use positive tone or body language***  My doctor’s quite good. Her tone is good (Woman 14 Chinese age 67).  POOR  ***Doctors are rushed***  The specialist he really very short time spend with me. He didn’t do much. He just come in and gave the injection that’s it. The injection was about a minute or two and then I’m done and I go home… it would be good to give me some information or a link for me to check out more information (Woman 17 Chinese age 66).  Once in a while I go to a walk-in doctor, and I think they are inpatient because maybe they have a long list of clients to see (Woman 14 Chinese age 67). | GOOD  ***Healthcare providers listen***  [The doctor] asked me how are you feeling? how I can help you?... giving me the good smiling face. And then when I’m explaining, she’s listening carefully what I’m trying to tell her (Woman 21 Pakistani age 65).  I went to my family doctor and he’s very humble and he talked to me and listened to me nicely (Woman 25 Pakistani age 60).  ***Good relationship fostered over time***  My family doctor is an excellent family doctor, she’s very empathetic… she’s been my family doctor for the past 20 years and it’s a very trusting relationship. She always welcome me, she always encourage me and she also support me and say kind words and always answer my questions if I have any questions, so she’s very supportive (Woman 22 Pakistani age 57).  ***Doctor use positive tone or body language***  It was welcoming… his body language is very kind, polite and gentle (Woman 20 Pakistani age 44).  POOR  ***Doctors are rushed***  The doctors are very busy these days. They don’t care about how you feel. They like to jump to what you want to tell them, what do you want know, or what hurts, quickly… he wants to come to the real issue and even though I do ask him doctor, how are you doing? Are you very busy? I try to make a conversation before I tell him what hurts me. But from my experience I see that doctors are not as welcoming as they used to be… they don’t have time to make conversation. They’re always in a rush (Woman 13 Indian age 67).  I wished that the doctor had spent more time. Doctors have a certain amount of time but they should have slightly more [time] say about 15, 20 minutes for each patient so it’s more personalized and person-centred… I was respected but had the doctor [made time for more conversation] then it would have been a more personal touch other than the doctor touching the knee and saying where’s the pain? (Woman 18 Indian age 69).  Doctor don’t have time. Doctor just write a prescription. They don’t have much time to talk and give advice or look at how I’m suffering. She don’t have time to look at me in my eyes or anything (Woman 27 Indian age not reported).  ***Doctors do not show interest in patient as a person***  I want [doctors] to be more kind, to be more caring, asking how are you doing? How’s your family? Something that makes patients feel connected for the long term. But that’s not there anymore, totally gone (Woman 13 Indian age 67). | GOOD  ***Good relationship fostered over time***  I have had the same GP for 30 years or more. She was lovely, she sent me to a rheumatologist who I liked a lot. I think she has all the time in the world for you; you can spend an hour to an hour and a half in her office as she examines you. She’s incredible in terms of how she treats people (Woman 01 Caucasian age 69).  I have developed a relationship with my primary care physician. I think there’s mutual respect that’s developed over time as opposed to just about arthritis because by the time you reach 70, you’ve got more than one issue and I see her every 3 months (Woman 08 Caucasian age 70).  POOR  ***Doctors are rushed***  The surgeons are in and out of the room in one second. Surgeons are surgeons, right? You’re in, you’re out. They tell you the x-rays look great, and you’re done. I’m not gonna say anybody’s been rude or nasty to me or dismissive; that’s just what they do. Where I go for physio at <hospital name>, the physiotherapist just laughs and says, [the surgeon has] got a foot and a half out the door before they walk in which is exactly right (Woman 01 Caucasian age 69). |
| **Exchange information**  Listen to concerns, prompt for additional details, understand needs, goals, circumstances and preferences, use lay language, ensure privacy  *Q: When talking to you about osteoarthritis and how to manage it, what questions did healthcare providers ask you to understand your osteoarthritis symptoms and its impact on your life?* | GOOD  ***Discussed impact of OA on daily life***  [My care provider] took time to explain to me…I had someone who was listening to me… also them sharing information on how I can deal with [OA] and manage my pain (Woman 07 African age 43).  [Healthcare providers] asked me about how I was feeling, what my pain feels like. Where are my joints sore? Have I ever been injured? What makes my pain worse?...I felt like the questions were very detailed… they wanted to know the information so they can provide the correct diagnosis (Woman 07 African age 43).  [Healthcare providers] asked me what kind of foods that I eat, my weight, how many times I exercise… they also asked about what I do which makes me move from place to place, if I am not exercising (Woman 15 African age 45).  I would come in and say I have problems with my knees, they would look at my knees… they asked what [activities] I do and about my weight and exercise (Woman 24 Caribbean age 67).  POOR  ***Little to no discussion about impact of OA on daily life***  I was the one who asked a few questions… I did see [the specialist] once and he recommend that we get the x-rays, then I saw him another time for talking about what the x-rays were, he showed me the images, but he never gave me any information or asked me anything… it was just a straightforward visit. I took the time to probably go on-line and Google things myself (Woman 03 Caribbean age 48). | GOOD  ***Discussed impact of OA on daily life***  [my healthcare provider] asked about my diet… I shared to them like when did [OA symptoms] start happening. They asked, what do I eat? What do I do every day? Do I exercise? What do I do after work and what do I do at work? (Woman 05 Filipino age 40).  [Healthcare providers] asked me what are my activities? Am I mostly staying at home just doing chores and not exercising? I told them I’m not really having regular exercise… I’m just at home doing chores (Woman 10 Filipino age 48).  POOR  ***Little to no discussion about impact of OA on daily life***  [The doctor] just asked me about my lifestyle… what exercises I’m doing and that’s all that I remember (Woman 12 Filipino age 54).  [My doctor] doesn’t ask the impact on my life. She just asks how do you feel? What makes you uncomfortable? What kind of action you cannot make? And she test me here and there because she wants to find the symptom. Then she refer me to do blood test, to do x-ray, to do ultrasound. I think she’s more concentrated on looking at the report to diagnosis my problem… I don’t think she will ask so deep, deep into things… she doesn’t need to do extra mile to ask me [several questions] because I’m not their only patient (Woman 14 Chinese age 67).  [Healthcare providers] didn’t ask me anything. I told [my doctor] everything. I told them my problem… I asked him to do the x-ray to find out the problem (Woman 16 Chinese age 62).  [The specialist] didn’t say much to be honest. He asked about the knee pain and if [it doesn’t] seem [to be] going away he say the jelly injection is good and that’s all. No further follow up. Not much time spent explaining anything. Even a nurse at the front counter say the doctor is too busy. So [OA care] could be better because I think this OA upset lots of people right now (Woman 17 Chinese age 66).  It was a quick visit. [The occupational therapist] didn’t really ask a lot to learn about the OA probably because I’m in the profession that I’m in and I was trained a little bit on hand therapy. So I volunteered information to her, so maybe that’s why she didn’t ask (Woman 19 Chinese age 46).  The clinic visit sometimes depends on the mood of the doctor. When she’s so busy, she doesn’t have time to discuss about things with you in the clinic. If you can see that the whole clinic lounge or a waiting room is full of patients, you know [the appointment] is going to be 5-minutes or less and that’s it. They give you the prescription and that’s it. But if there will be times when the clinic visit is not really that busy, then she has time to explain everything that you ask her to. So it’s frustrating and sometimes you get angry when you leave because all they want to do is get rid of you because more patients are waiting for the room (Woman 26 Filipino age 67).  ***Doctor should help patient understand OA***  Nothing has been said… what [healthcare providers] could have said, what do you know about osteoarthritis? What do you think we can do for your osteoarthritis? What would help you manage your osteoarthritis at home? (Woman 26 Filipino age 67).  It seems like when you have to see a doctor they will entertain you of course but they will just give you something for you to relieve the pain. [OA] is not really well explained… they just give me readings and that’s it…they should explain [OA] (Woman 12 Filipino age 54). | GOOD  ***Discussed impact of OA on daily life***  [My doctor] asked about my work and my family history. He asked do you have arthritis in your family. And he asked about my home, like how many stairs are you going up and down. And he asked how do you clean your home? How many kids do you have? When are you suffering this pain? (Woman 25 Pakistani age 60).  I was diagnosed 2 years ago [with arthritis] but it’s not that bad… it have no impact so far on my life. So I don’t think she asked about that before because I didn’t show any concern about the arthritis to her… [the doctor] asks is there any symptom or any problem you want to discuss with me? Or is there any pain? ... she give me the open-ended questions, so she said if there anything that I want to share with her regarding my condition (Woman 22 Pakistani age 57).  POOR  ***Little to no discussion about impact of OA on daily life***  [OA] is really affecting my life because I am a mom of two teenager, I am a working woman I’m really, really good sports person and I have a really big social set up. So [OA] really affect my daily routine… the doctor should realize my position right? He should ask me what are your activities and then how it will affect your daily life and then if he feels that I should go for counselling, he recommend me to go for a counselling… depression, stress, anxiety, those kinds of issues (Woman 20 Pakistani age 44).  I think there should be more time for the doctor to talk to their patients, so they can know if there’s any background information or a family history and how patients are handling pain and can improve their quality of life (Woman 13 Indian age 67).  [Healthcare providers] just say, when did you get the pain and how are you feeling? Can you walk and do you get [knee pain]? But I would have loved if somebody asked me about the genetic factor, whether my parents had it or not. Or about temperature change like does your pain increase when it rains or when there’s humidity in the air. What sort of foods do you eat? (Woman 18 Indian age 69).  ***Doctor should help patient understand OA***  [The doctor] listened to me what I was telling her. I was explaining my pain… showing my hands, showing my legs that I’m feeling pain on these joints and on my knee when I’m bending the knee it give me more pain. She just only listen and she did not explain what is the reason, why this? She should explain me more about my bones, how they work (Woman 21 Pakistani age 65).  [The doctor] diagnosed my arthritis and at that time [I knew it’s arthritis] but I didn’t know what is [meant by] osteoarthritis and doctor didn’t guide me [or ask me] anything (Woman 27 Indian age not reported). | GOOD  ***Discussed impact of OA on daily life***  A lot of the questions involved the impact that the knee was having on my work. Now [the questions] focus a little more on what is it that you want to do in your life and how is it being impacted. And actually for the first time [healthcare providers] are asking about my sleep; how much it’s waking me up at night (Woman 06 Caucasian age 65)  Physiotherapists ask me questions like when does it hurt? Is there anything you do that makes it hurt more? Hurt less? Let’s try some acupuncture. Their goal is to get you moving again or moving with less pain and they’ll ask questions about range of motion, or they’ll manipulate your body and figure out your range of motion and your degree of pain…I probably have the most beneficial information from a physiotherapist because  they have the time, they have the half hour, the 40-minute appointment and while they’re working on you, you can talk and ask them questions (Woman 08 Caucasian age 70).  The physiotherapist was proactive. I think we would talk about the nature of the pain and when it started to occur and triggers (Woman 09 Caucasian age 70).  I’ve been asked many times over the years about activities of daily living and what I’m able to do and what I’m not able to do. And my physiotherapist does address my movement restrictions in terms of assessing what therapy needs to be provided (Woman 11 Caucasian age 72).  POOR  ***Little to no discussion about impact of OA on daily life***  In the earlier years it would have been nice if they tried to understand how much physical activity was in my activities of daily living as well as my work life. I think it would have been helpful for me to have a little bit of direction on the modification of activities or information on how to adapt at work and in activities outside of work so that I could maintain a higher more comfortable level of functioning (Woman 06 Caucasian age 65).  I guess [my doctor] could have asked me more questions in terms of understanding the nature and the extent of how I was feeling at the time (Woman 09 Caucasian age 70). |
| **Address emotions and concerns**  Actively inquire about feelings, acknowledge concerns, express empathy, note that such feelings are normal or common, suggest strategies to cope or mitigate emotions  *Q: You may have had some worries about your osteoarthritis. How did healthcare providers learn if you were feeling worried or upset or stressed out about osteoarthritis and its impact on your life?* | GOOD  ***Discussed emotional impact of OA***  [Healthcare providers] were asking me about the pain and how it was affecting me... I told them I was very irritable and the pain was affecting my social life and my relationship with others… they were concerned about how I was feeling and I was open to tell them about my experiences and emotions (Woman 07 African age 43).  [Healthcare providers] advised me on the medication to help my pain go away and they talked to me about my mental health about how I should be handling my emotions because these are stressors of life and I have to know how to deal with them (Woman 07 African age 43).  When I arrived at the doctor’s I was worried and scared of the outcome of what I would be told but was trying my very best not to show it but they read my body language as I was explaining my symptoms… the doctor noticed that I was shaking a lot and asked if I was worried and I said yes… [the doctor] talked to me and we made a friendly chat. [The doctor] asked about my symptoms and that helped reduced the tension… when the results for the x-ray were being processed, they left me with one doctor so I wasn’t lonely and I had someone to talk to and it helped to reduce the tension as I’m waiting for the results (Woman 15 African age 45).  POOR  ***Little to no discussion about emotional impact of OA***  No nothing of that nature… there were no consistent check in [about how I was feeling] (Woman 03 Caribbean age 48).  I did get some healthcare professional who listened to my needs or my concerns and help me understand…when you live with something for so long it comes like second nature. I didn’t have any excessive feelings or emotional problems dealing with [OA]. When [OA] did start to happen and affect me, I just accepted it… I didn’t go to [the doctor] with any [emotional] concerns (Woman 24 Caribbean age 67). | GOOD  ***Discussed emotional impact of OA***  If I am feeling down because of my health problem, then [the doctor] talks to me and she would spend more time during my visit and talk to me about that… she probably think that I’m not up to a point where I need a mental care consultant or something and so she just talk to me. And she kept on saying that if I am feeling down again, just make an appointment, she will talk to me again… I’m still functioning, I still can drive, I still can walk, I still can do my daily chores. So she probably think that I’m not that bad yet. I think I’m not that bad yet but some days I’m not that good either (Woman 23 Chinese age 70).  POOR  ***Little to no discussion about emotional impact of OA***  I was concerned about deformity in my hand which is why I was also there for splinting. So in addition to the pain, I was worried about deformity…the [occupational therapist] did not specifically [tell me how to cope] because I think the splinting itself would help with [my concerns]. She just validated my reasons for being there said the splints will help with preventing [deformity] (Woman 19 Chinese age 46).  Unless you bring this to your doctor, she doesn’t ask those emotional or what you’re feeling questions… no she will not ask those questions, just diagnosis and then see in what way she can help… as long as I know that she cares about her patients… and she has the attitude that she want to help the patients and cure your disease then I think it’s okay… I think she tries her best to cure my problem (Woman 14 Chinese age 67).  I think [healthcare providers] are not saying this kind of thing much. Only because I ask question, they will answer my question… if I had some doubt or fear or worry, I’ll ask them what can I do? So they will comfort me. But as usual, if I don’t ask, I don’t think they will talk too much to me about [emotions]… maybe [healthcare providers] should ask what we are feeling, ask what we worry about… they just treat your body right? (Woman 16 Chinese age 62).  I don’t think [healthcare providers] were worried about [my OA] at that time. All they want to do is just finish your clinic visit and that’s all… some [healthcare providers] are more supportive than others (Woman 26 Filipino age 67).  Most of us when we have osteoarthritis of course we are worried. My doctor just explained to me that getting to a certain age we can experience all of that, but you can prevent [OA] if you do some of the things that the chiropractor will tell you (Woman 12 Filipino age 54). | GOOD  ***Discussed emotional impact of OA***  He told me don’t take stress because stress also influences your pain… he told me [OA] is a natural process of degeneration so don’t worry with age it comes. He told me to think about good things, to be happy. Your life is precious do whatever you like to do… so I become stress-free and good (Woman 04 Indian age 55).  When I was upset about my arthritis, I got relief when I went to my family doctor… I was very mentally relaxed when I talked to him and he was very kind to me… he talked to me very nicely and he tried to tell me that you are suffering this pain but you can get relief… I was very satisfied when I see my doctor (Woman 25 Pakistani age 60).  ***Doctor is open to discussing emotional impact of OA***  I never showed any worries to her [for OA] so far… not necessarily for the arthritis but for any other for example, for my diabetes, so it can translate in that situation. She’s always very sympathetic. She’s always very supportive and she take time to discuss and she make sure that I have all the answers to any question I might have, so I’m really satisfied with her attitude and behaviour towards me (Woman 22 Pakistani age 57).    POOR  ***Little to no discussion about emotional impact of OA***  A holistic touch about the whole treatment, rather than talking about medicine and symptom centric treatment would have been how are you feeling or do you feel segregated from your friends or how do you feel if you cannot go for a walk. Previously you would jog and now you cannot… it could be a more doctor friendly level, rather than an impersonal level (Woman 18 Indian age 69).  If [doctors] have no time to talk or have a connection with you, then I don’t think they even care about knowing how the pain is affecting your life unless we tell them... I describe [how I am feeling] and he does listen but there’s no response except, okay take these pills (Woman 13 Indian age 67).  This [osteoarthritis] is very depressing and is very sadness in my life… I’m worrying about [OA]… and doctor said you have arthritis, so you have to live with that and if you have pain you have to take painkiller and do exercise. No other advice or anything… if doctor or nurse or any health provider can help me on how to live with arthritis day-by-day… help me to find out any resource where I can go and do exercise or maybe lower the painkiller or do any other activities so I can [reduce the] sadness in my life (Woman 27 Indian age not reported).  Doctor should not stress me out that this is the problem and that you have to live with it. Psychologically it should not be horrible or scary. Doctor should tell me what exercises I should do to heal my pain and how much supplements and what diet will be good for the arthritis patient instead of saying because you are getting older this problem will persist (Woman 20 Pakistani age 44).  ***Doctors expressed little to no empathy***  I never see my doctors empathetic, counsellors are empathetic, but doctors are not empathetic.They should have some level of empathy during their conversation with the patient (Woman 20 Pakistani age 44). | GOOD  ***Discussed emotional impact of OA***  [Healthcare providers] could tell [I was upset] because of my bursting into tears in their offices. I’ve had a pretty traumatic experience over the last almost 25 years with the various [surgical] complications that I’ve had. I have not been shy about expressing my frustration and my distress that is a daily part of my existence. And I worry greatly about [OA] getting much worse as I age. So I’ve been very explicit about my concerns… and [healthcare providers] were able to address some of my concerns… I’ve been referred for ultrasound and I’ve been referred for physio (Woman 11 Caucasian age 72).  POOR  ***Little to no discussion about emotional impact of OA***  To be honest I’m not sure that anyone ever asked about how it was affecting me emotionally or mentally - other than fatigue and sleeping issues because I was very limpy physically… but no, [emotional or mental support] hasn’t been a big component of my care to date (Woman 06 Caucasian age 65).  ***Doctors expressed little to no empathy***  I do remember going for an x-ray on my knee and I said to the x-ray technician it hurts when I run and he said, well that’s stupid… basically you’re a stupid woman for running. I’ve had bad experiences like that. I think if [healthcare providers] said good for you for staying active and trying to run it would have been better than saying it’s your own fault… having empathy and saying you got a problem and let’s resolve it so you can get back to doing what you need to do for your health (Woman 08 Caucasian age 70).  When I have an injury or when things hurt I get much more sympathy and empathy from a physiotherapist [compared to a doctor] because they have the time… there are people out there who deal with arthritis patients who emphasize. There are others who just want to rule out if there’s anything else that needs to be done (Woman 08 Caucasian age 70).  There were no touchy, feely questions… I didn’t find a sympathetic or empathetic ear with the medical doctor (Woman 09 Caucasian age 70). |
| **Manage uncertainty**  Offer rationale for tests or treatment, describe likelihood of risks and benefits using words, statistics or pictures  *Q: In some people, osteoarthritis may get worse over time, and treatment may or may not improve symptoms. How did healthcare providers explain the chances that osteoarthritis might get worse or how well different treatments might work?* | GOOD  ***Discussed uncertainties around OA***  [Healthcare providers] talked about how [OA] can get worse… they give me solutions like exercises or physiotherapy services about how I can manage it better instead of concentrating on how much worse it can get… I know I can get worse but my doctor usually encourages me and helps me (Woman 07 African age 43).  ***Described prognosis or outcomes using words***  [Doctors] explained to me that since I’m suffering from knee osteoarthritis, running and jumping also hiking would make it worse for me since my cartilages aren’t really strong… I can’t really remember if they were numbers but what I can remember most was the words they told me (Woman 15 African age 45).  POOR  ***Little to no discussion about uncertainties around OA***  He just said to me that it could possibly progress the older I got; that was it. And if I needed to continue doing sports and so on that is what I needed to do. There’s not a lot of conversation about it. It felt a little rushed in terms of the time. He had a lot of people waiting (Woman 03 Caribbean age 48). | GOOD  --  POOR  ***Little to no discussion about uncertainties around OA***  [My chiropractor] talked about risks really quickly… he just kind of summarized what’s in [the consent form]… [he explained] this is the benefits of the chiropractor…then they said there’s a risk but a really small chance of getting paralyzed or something… he mentioned about [how the OA might get worse] but he discussed more on the benefits of doing chiropractic… they said if I don’t change my diet and if I don’t exercise it’s gonna get severe (Woman 05 Filipino age 40).  We haven’t dig deeper into the chances of my osteoarthritis getting worse. [My doctor] is just telling me to watch my weight… I think then she said we will have a deeper discussion about the osteoarthritis in our next appointment (Woman 10 Filipino age 48).  She never explained the chances that [OA] getting worse. She never talk to me like that, no. Maybe what happened is I just did the x-ray last month… so she want to let the specialist see me first and then make the conclusion (Woman 14 Chinese age 67).  Honestly, [doctors] didn’t tell me about how [OA] would get worse but because my mom have this problem… I know what can happen in my future (Woman 16 Chinese age 62).  [My doctor] is telling me that for now I just have to see my chiro and if it gets worse then it’s the time that we have [more conversation]… she told that it might get worse if it is not treated immediately… she says if you do some self-care, it will complicate or it will get worse if you didn’t get the right treatment. So it’s better for you to seek a doctors advice instead of you treating yourself (Woman 12 Filipino age 54).  No we didn’t discuss [about OA getting worse]. It was a tightly timed appointment. She had half an hour to do my history, do my splints, make sure everything was good and send me off (Woman 19 Chinese age 46).  No [healthcare providers] didn’t say anything about [OA getting worse] but I feel it myself, that this getting slowly, slowly creeping to the worse side (Woman 23 Chinese age 70). | GOOD  ***Described prognosis or outcomes using words***  My specialist had that MRI x-ray in front of me and he explained to me this is what your knee looks like now and this is how it may look after another 6 months or so if I don’t take an action (Woman 13 Indian age 67).  Because osteoarthritis was there on the x-ray when I was being diagnosed she then described about this is a chronic process and it will progress with the time. But with the management [like] healthy lifestyle and weight reduction we can delay this process (Woman 22 Pakistani age 57).  ***Discussed uncertainties around OA***  [The doctor] told me that since [my OA] is a mild case, you got to look after yourself and when you have any pain use these medication or topical ointments. And she says, in case it does get worse then come to me again (Woman 18 Indian age 69).  I was suffering knee pain. So he explained me that it’s because the age factor and the workload and if it will [become] worse and you can’t walk or you can’t get relief after the painkillers then I will put your name in the list [for joint replacement] but otherwise if you can bare it don’t worry about it. One time I’m suffering very severe pain so he wrote some x-rays. Always he help me (Woman 25 Pakistani age 60).  POOR  ***Little to no discussion about uncertainties around OA***  The doctor said that if you are not going to pay attention on your diet or supplements, then it will go worse. He said unfortunately this is the natural process and that it will get worse by the time of your age. I felt that the doctor mean that he can’t do anything because this is how the disease is (Woman 20 Pakistani age 44).  [The doctor] did not explain. She did not explain anything for how [OA] would become worse. Which I have no clue with that (Woman 21 Pakistani age 65).  Doctor didn’t explain to me anything. She told me that by age, [OA] gets worse because recently I complain [about] more pain (Woman 27 Indian age not reported). | GOOD  ***Described prognosis or outcomes using statistics***  [The surgeons] both told me I would have 100% recovery (Woman 01 Caucasian age 69).  I think they were good with [discussing the chances of osteoarthritis getting worse] because right from the get-go… they said, this is not gonna get better it’s only gonna get worse which actually at the time was a little depressing. I was given 60% chance you’ll be better or 30/30, 30 better, 30 same, 30 worse (Woman 06 Caucasian age 65).  POOR  ***Little to no discussion about uncertainties around OA***  I have never had conversation [about OA getting worse]. Originally when I saw the rheumatologist it was basically just to get a treatment plan (Woman 08 Caucasian age 70)  There was no explanation of the future and how it would get worse. It was just the present… how to deal with [the OA] on a day-to-day basis (Woman 09 Caucasian age 70).  Not to my recollection was there any explanation of the [chances of OA getting worse] … osteoarthritis is understood to be fixable… I think it’s still very uncommon to think about osteoarthritis as a chronic condition with or without joint replacement… there’s very limited awareness of the [possibility] of osteoarthritis getting worse following surgical intervention (Woman 11 Caucasian age 72). |
| **Share decisions**  Describe treatment or management options, assess interest in shared decisions, provide information to enable shared decisions, suggest factors to consider in making decisions  *Q: How did healthcare providers involve you in talking about or choosing different options for treatment or self-care?* | GOOD  ***Discussed options for treating or managing OA***  We talked about the various treatment options and the medications that I could choose and how I would adjust my lifestyle… [the doctor] asked me about my preferences because I was willing to take medications and exercise but I refused the option of surgery (Woman 07 African age 43).  POOR  ***Little to no discussion about options for treating or managing OA***  No, we didn’t have that type of conversation… I didn’t get that level of support, no. He basically just gave me advice to do exercise, physiotherapy and he gave me a prescription for a brace (Woman 03 Caribbean age 48).  ***Current treatment options focused only on surgery***  [Healthcare providers] gave me a lot of different choices going along [for OA management] like exercise or injections… for the injections [the doctor said] it’s not a permanent solution but it should help for a couple of months… in more recent years the only thing [suggested to me] is have a knee replacement because the person that I was seeing is an orthopaedic surgeon, and I didn’t particularly want to do it… he’s not willing to give you alternate solutions, he says you have no choice, you have to replace it, and then tries to convince you that that’s the best solution right now and I don’t know if that is absolutely necessary (Woman 24 Caribbean age 67). | GOOD  ***Discussed options for treating or managing OA***  [Healthcare providers] are nice. What I like is that they didn’t impose because it’s up to me to do what I want. They [asked] me if I’m into physical fitness and I told them no. They didn’t give negative feedback on that. They said, if you’re considering exercising that would be good for your health. And if you are considering having fruits and vegetables for your healthy diet that would improve your condition. They have given me options… they are pleasant, and they discuss clearly (Woman 10 Filipino age 48).  POOR  ***Little to no discussion about options for treating or managing OA***  [The specialist] just said that this is the treatment and that’s about it to be honest. I wasn’t involved in much decision-making…they could have a plan for me to see if [injections] work and then what if this injection doesn’t work, what will the plan B or something like that. It wasn’t spelled out, like I wish I could know more (Woman 17 Chinese age 66).  It seems like [healthcare providers] assume that you already know about OA and what will complicate it…it's better if they will explain to me better about osteo, what are the symptoms, what are the dos and don’ts if you have osteo, what preventative measures should be done to improve your osteo…instead of you have osteo, so I will just give you medications and then you have do some x-rays and then you’ll just go to a chiro and that’s it…and getting my opinion about what can be done…and [asking] what is your day-to-day work or what is the nature of your job (Woman 12 Filipino age 54).  I remember the [occupational therapist] asking a little bit about my activities to help her choose the material for the splinting itself. And then just continually asking for feedback every time she tried it on. And asking me to move and everything to make sure that it was comfortable and well fitted, so that I would wear it.  I think, if she’d had the time maybe it would have been useful for her to just provide a quick overview about treatment options, making sure that the patient understands the natural course of osteoarthritis, how not to make it worse or to manage it. I think even just a quick screen to ensure that the patient is aware of all of those things would have been useful (Woman 19 Chinese age 46).  I did tell [my doctor] sometimes that because of my [OA] pain, especially my shoulder, it wakes me up in the middle of the night. Then she says, well go to physio and they will teach you do some appropriate exercise to relieve the pain, but I find that is not helpful… the exercise that they gave me when I go to visit at physio, it’s a temporary relief, it’s not long term… I told [my doctor] that I do the exercise that [the physiotherapist] taught me but it’s not helping me that much to relieve my pain sometimes… [my doctor] didn’t tell me any options. She just told me to take it easy and not do too much heavy stuff or not do anything that is causing the pain (Woman 23 Chinese age 70). | GOOD  ***Discussed options for treating or managing OA***  The specialist gave me choices between two things. Either to get the surgery done to replace the knee or do pain management… and [he said] it’s up to you to make a choice… then I inquired about pain management and he gave me different choices, he said you can take these pills, you can take a brace and put it on your knee… he suggested which [brace] is good for me. He gave me different choices until I have to replace [my knee]. But he says, [management] can delay the process for knee replacement and it can help you to walk… [the specialist] welcomed me to ask any questions. He didn’t rush to leave me (Woman 13 Indian age 67).  [My doctor] give me all the options which are available and encourage me to research more about that. And if I have any questions just ask her… whenever I have a question or concerns, I’m very comfortable to talking with my doctor and she always give me options and other strategies or if I don’t want to take medication that’s okay because it’s my own choice (Woman 22 Pakistani age 57)  POOR  ***Little to no discussion about options for treating or managing OA***  I usually like to tell everything, [to my doctor] like I can’t sleep at night… I cannot walk anymore. My weight is going up. He listens but he doesn’t really give me any suggestions. He says take these pills… and see me after two weeks and see how you will do. He doesn’t have time to explain the proper posture you can take when you have that pain or how much you can walk or to stop walking if it hurts more or something that the doctors know more to do or not to do. I think these days their focus is only prescriptions… which I don’t really like to take pills for pain (Woman 13 Indian age 67).  If you will ask me the scaling from 1 to 10 [on the options given], I would say 3 ½ … He didn’t explain me very well. He said if your pain gets worse then you can go for the pain management supplements. But he didn’t tell me about the exercises that I should do and what I should not do… he said you can have steroid injections for pain. That’s the only solution for that. And I don’t want to take steroid because I had taken one time for my shoulder and it really give me the side effects, my periods were irregular, I had mood swings and my appetite was affected by this (Woman 20 Pakistani age 44).  [Doctors] didn’t mention anything about treatment options. All they said was you should rest. But nothing about exercise, diet, or reducing stress (Woman 18 Indian age 69).  Doctor, she did not explain anything to me… she [should] tell me what type of food I have to eat, what medicine I have to take, will it have affect on me or not… because [the doctor] studied and she’s more familiar of the diseases and medicines. That’s what I’m expecting from her but so far she didn’t tell anything other than that I have to walk or take Vitamin D (Woman 21 Pakistani age 65).  [My doctor] didn’t tell me anything about how can you go do this treatment and that treatment. He just said it’s too early to change your knees. He didn’t explain to me [different treatment options] and this is better and this is not better for you. He didn’t tell me anything (Woman 25 Pakistani age 60).  [The doctor] said, for arthritis there is not any other treatment the only option is painkiller and exercise but I don’t [have] much belief in painkiller… [The doctor] said why you don’t take painkiller… day-by-day your problem is getting worse and why you don’t live like good life with your medication, if you don’t take medication then you suffer; why are you suffering? (Woman 27 Indian age not reported). | GOOD  ***Discussed options for treating or managing OA***  When I was told that I have to have the surgery, I was also told if I wanted to wait, I could wait but that eventually I would have to have [surgery]. The knee surgeon gave me different options like having injections (Woman 01 Caucasian age 69).  I’ve never been told this is what you need to do... I found [healthcare providers] have always presented me with options of what we can try and these are the risks and potential benefits (Woman 06 Caucasian age 65).  The only caution that has consistently been raised in my surgical care for osteoarthritis has been the potential risk of infection post-surgery… I was involved in selecting a physiotherapist… I had a choice of having in home physiotherapy for which I would pay or going to an outpatient physiotherapy program that was covered by OHIP at the hospital … I was involved in getting post-surgical inpatient rehab… I was actively involved in those decisions. I was presented with the options and I made a decision about those options (Woman 11 Caucasian age 72).  POOR  ***Current treatment options focused only on surgery***  I guess I’m frustrated because I don’t feel like I’m at the stage that needs a knee replacement and I feel like that’s all they have to offer me… it seems kind of extreme for someone who can go up and down stairs, walk a few kilometres, skate, and downhill ski… I feel like there should be something in the middle… healthcare providers are not helpful to me because I know the drills. I’ve just hit this plateau… it’s like don’t bother coming to our office, we’ve looked at it (Woman 06 Caucasian age 65).  ***Little to no discussion about options for treating or managing OA***  I don’t know what’s available. That’s the problem when you don’t know you’re basically told, oh that’s just a sign of aging or because you had some breaks. You don’t even know what range of options are out there. It’s kind of like grin and bear it, take Tylenol and don’t not exercise, move your limbs even if it hurts but that’s all. I’m thinking I’m just gonna have to live with this nonsense. And everyone I know feels that way (Woman 09 Caucasian age 70). |
| **Enable self-care**  Set expectations for follow-up care, offer advice on self-care, provide take-home information, refer to other sources of information or support  *Q: How did healthcare providers prepare you for next steps for your osteoarthritis care? This refers to your next visits with them and to self-care.* | GOOD  ***Offered self-care education, advice, or support***  [My doctor] told me about on-line resources… I indicated [to the specialist] that I was interested in continuing some exercises and continuing in sports… he recommended exercise. He gave me a prescription to get a brace and he recommended that I get physio if that was something that I was able to access out of pocket. And that was the extent of the care that I got for the OA (Woman 03 Caribbean age 48).  At the start, I did not have information and awareness about [OA]. I was confused because I did not know how to manage the disease… was I supposed to do exercises? … [since going to my care provider] I’ve been referred to a lot of information on how to maintain healthy weight, diet, how to maintain my blood sugar levels and how to deal with [OA] symptoms… I feel like the information was helping me a lot (Woman 07 African age 43).  [Healthcare providers] prepared me for next steps by offering me a schedule for what I’m supposed to do to improve my condition… for example, on lifestyle, the dieting, the exercise… honestly, these would improve my care before the next appointment and now I can stay active, eat balanced diet to lose weight… keep my pain under control. All these things I could do them at home (Woman 07 African age 43).  What I liked was I received proper care in terms of medications and advices which I was able to follow… I was overweight so [the doctor] advised me to reduce my weight… they advised me to exercise and not to eat red meat and sugary foods which would increase OA level in the body… [the doctor] said if I decide not to choose right, then I am going to suffer more (Woman 15 African age 45).  ***Referral to programs or professionals***  I got a recommendation from my general practitioner to a specialist when I expressed that I had knee pain (Woman 03 Caribbean age 48).  ***Doctor set expectations for follow-up care***  [The doctor] wrote me an appointment for the next visit… and told me to always visit the hospital whenever I feel I am not okay (Woman 15 African age 45).  POOR  ***Doctor did not set expectations for follow-up care***  When someone says to you, this is what you have and it’s gonna progress anyways and maybe you get to a point when you get older where you may need a knee replacement. It’s almost like they’re just saying, hey this is gonna happen, there’s not a lot of things that you can do in between. That’s really what I got from my conversation with the specialist and so it doesn’t allow you to say, okay is there an on-going treatment or on-going support. Do I come and see you every 3 years? Something to that effect… a more fulsome support on a regular basis would be useful. Maybe something every year that you go in, see someone who can give you some support (Woman 03 Caribbean age 48).  ***Not offered referral to programs or professionals***  What I would have preferred since I was somebody who was introduced to weight loss that they recommend me a physical exercise person who can be there, 3 or 4 times a week who can make it seem compulsory to me. Weight loss and doing exercise was a new thing in my life and [healthcare providers] didn’t really provide support… I had to look for a way myself of losing weight (Woman 15 African age 45).  ***Offered little to no self-care education, advice, or support***  Some [healthcare professionals] offered advice. Some suggested physiotherapy and gave me exercises I think… I could have had more… it’s only when [Google] came along and then you would be able to research and find other opinions and more information rather than what you would get just going into a doctor’s office for 5 minutes… [advice] wasn’t tailored to my individual needs. They’d say, okay this is a list [of options] you can try (Woman 24 Caribbean age 67). | GOOD  ***Offered self-care education, advice, or support***  First, I visited the chiropractor and then a physiotherapist. They said that the main thing why I have [OA] is my posture… if I stand or sit, I slouch, especially I work in front of the computer for 8 hours… So they said to have proper posture (Woman 05 Filipino age 40).  If the pain is really severe [healthcare providers told me] I can take Tylenol or Advil… they said several regular exercises like 15-minute walk everyday would be good (Woman 05 Filipino age 40).  Physiotherapist gave me that exercise and some articles to read about caring for my body or caring for my overall wellness… chiro gave me insole support… I know [healthcare providers] have the best advice they gave me. It’s just for me to watch out for my diet (Woman 10 Filipino age 48).  My doctor recommend for me to see the chiropractor… after seeing my doctor and then I talk all the symptoms and then he recommended me for an x-ray and then he also gave me some medications and then I felt relieved about it… [my doctor] explained a little bit about what is osteoarthritis, she even gave me some readings about osteoarthritis and then some exercises that I should do (Woman 12 Filipino age 54).  ***Referral to programs or professionals***  [My chiropractor] referred me to specific groups here in <city name>, we have <community program name> and they have nutritionists who are running that program… I am happy he referred me to <community program name>… what I really appreciate what my chiropractor did is he informed me about an independent women’s group talking about women’s health… this group is more accessible compared to the doctors… this not-for-profit group would give you the same advice as what the doctors would give you (Woman 05 Filipino age 40).  [My doctor] asked me about my health practices and diet. I told her that our [eating habits] at home contributes to my unhealthy diet and weight gain. And I didn’t do much exercise… then she referred me to a dietician (Woman 10 Filipino age 48).  POOR  ***Offered little to no self-care education, advice, or support***  [My chiropractor and physiotherapist] said it’s not severe yet… so I don’t need those things like special shoes or an adjustable desk… so [pain] keeps coming back and then every time I visit them, I get the same advice (Woman 05 Filipino age 40).  [My doctor] said you can find available information on the internet about managing osteoarthritis… I was waiting for her to give me particular websites, but I didn’t push on that. I should have asked is there a best recommended website because there are lots of information you can find in the internet. I should have got that advice from the doctor… so I just do that myself (Woman 10 Filipino age 48).  [The doctor] recommended me to take calcium and to do some exercise… she didn’t refer me any information or education… I use my own personal effort to search what food is good, what kind of exercise is good for you. So very lucky I’m retired. If I’m not retired then I don’t have that time to invest in searching all those information to help prevent this kind of disease (Woman 14 Chinese age 67).  At this stage I will say [my family doctor] did not say much. He said that if not that serious better not to take the medicine because it hurt the stomach… I’m seeing my family doctor and he told me don’t overuse [the joint… keep it warm with a heat pad or something will help… do some exercise to strengthen my muscle so the joints is more steady and stretching… I study [OA self-care] myself (Woman 16 Chinese age 62).  [Healthcare providers] can give me more information and teach any kind of exercise to help me to decrease the problem. But they don’t have time honestly (Woman 16 Chinese age 62).  I learned [self-management] through the internet. I didn’t get much from the health professional about osteoarthritis care to be honest… when I asked the nurses if I need to inject my other knee she didn’t say much… I wish there was a little bit more education or more support that way I can look it up myself in the future [instead of] just you can ask if you need to repeat the injection… the specialist is not really into teaching you do any therapy and things like that because at that time I went for the injection it was still doing COVID 19. I just went for a few minute and then I have to leave. He didn’t do much education about it… I think [the specialist] just give me an information sheet and some medication company that published that and I don’t find it very helpful because they just talk about the different injections and medication or something and I don’t want to take medication and I hope I don’t have to do the injection too often. So, I’m still hoping to maybe find more areas how can I help myself… so that the pain won’t happen again (Woman 17 Chinese age 66).  I wish especially the specialist in this area could have link me up with some support or send some information to me about the self-care…they don’t have the time to explain to me further information which is not so patient-centred… I just have to look things up myself to initiate everything. I don’t think I’m being cared for as much as I could have… when I ask information it very short and yes and no answer. There’s no explanation and no information sheet or any hand-out for me [on self-care]. So not so person-centred (Woman 17 Chinese age 66).  Volunteering information about joint protection and reducing stress on joints would have been useful… discussing pain management strategies as well compression, acetaminophen or [other] evidence-based management for osteoarthritis (Woman 19 Chinese age 46).  If I do have some pain on my joints or whatever, [healthcare providers] advise me to go for physio but the physio treatment is out of my own pocket… my hope is somebody can tell me how to [manage OA] on a daily basis, do some exercise or do something that would help my osteoarthritis so that it won’t get any worse. But I don’t know where I can get that kind of advice… I told [healthcare providers] that I have pain all over my joints, but they don’t really direct me to anywhere and it seems that they are trying to avoid my problem. She kept on saying, if the Tylenol can help me just go ahead and take that when you’re in pain unless really bad then maybe they would do something, I don’t know (Woman 23 Chinese age 70).  At the moment, my osteoarthritis is very mild and so there’s not much care that the doctors have told me about except for it is an osteoarthritis, that’s it, what else can we do? [Healthcare providers] just told me that if it’s a little bit uncomfortable all I have to do is just put some pain ointment or do some self-massage at home for the arthritis symptoms on my fingers (Woman 26 Filipino age 67). | GOOD  ***Offered self-care education, advice, or support***  [The doctor] told me about the medications… he told me about naturopathy and how to massage and to eat well… [include] omega 3 and calcium in diet… I am feeling better after getting these things… he told me do regular exercise. He told me about physiotherapy. It will be beneficial for you he told me… he told me about making a chart [for exercise and diet] and to follow these charts, then you’ll see a difference (Woman 04 Indian age 55).  [The specialist] gave me the contact information for the brace person in the same building that I can go to look at different kinds of braces… he suggested to use a cream on my knee… he prescribed medicine. He said this will reduce the pain and then you can sleep better at night (Woman 13 Indian age 67).  When [the doctor] gave me the information about [OA], she said that there is no permanent solution… it’s going with the age… the best thing is [to do] exercises or lose a little bit weight. So that is a good thing that she was giving me the healthy tips… [she also said take] Tylenol which will relieve [the pain] a little bit (Woman 21 Pakistani age 65).  ***Referral to programs or professionals***  I suggested to [my doctor] that please send me to the specialist who knows more about joint pains, and he did (Woman 13 Indian age 67).  [The doctor] checked me very well and he said, you are not in this condition that we can put your name in the list [for surgery], you can eat only painkillers… I was suffering from long time this and it was not bearable for me and I was not holding my insurance too. I said I can’t eat too much painkiller because my stomach is not baring this painkiller. So he said, do you have insurance? I said, no. He said okay, I can give you a [referral] to the physiotherapist and they will handle you 3 times for [OA]… the physiotherapist gave me 3 sessions and after this they told me some exercises for the pain (Woman 25 Pakistani age 60).  ***Doctor set expectations for follow-up care***  She’s a family physician, so whenever I book an appointment they will ask you, why are you booking an appointment? So, I will say for my joint pain. And that’s the way that we work together (Woman 22 Pakistani age 57).  POOR  ***Offered little to no self-care education, advice, or support***  [My doctor] said that I should not do my workout as much as I am doing. He also recommended that I should drink milk and add more calcium in my diet, that’s all… I am not aware of the exercises that I have to do. Just only that I am adding calcium and vitamin D in my diet… other than that, I don’t know (Woman 20 Pakistani age 44).  I was not told that self-management is a big thing in managing pain for osteoarthritis. I would have wished that the doctor had mentioned about how good diet using anti-inflammatory foods would help you. Going to a chiro or a massage therapist would have helped… I wish the doctor had gone into more details about the type of exercise I could do. And also, had recommended to me to go to the website of the Arthritis Society of Canada because when I studied it on my own I saw that there was a wealth of information… all of that was not suggested to me. I study and talk to my friends and family and I got more advice from them (Woman 18 Indian age 69).  [The physiotherapist] told me the exercises but it’s my fault, I didn’t do it… on their end, they were doing their job good because they were giving me good treatment but maybe I didn’t follow them that’s why I didn’t get the relief… maybe they will give me some kind of other routine work, like how can you extend your life, how can you do your [exercise] and how can you deal with [pain]… maybe it will be better for me. They just told me [a lot] of exercises. Maybe if they will guide [me] it will be better (Woman 25 Pakistani age 60).  Doctor never give me any advice or anything… I was expecting doctor to give me proper guidance [about] how to handle arthritis and I’m so sad because I have arthritis. I’m upset [that] day-by-day you get disabled… [my doctor] just give me painkillers… If I got what kind of exercise, a diet plan, how to live day-by-day with pain, send me to a specialist, then it would be better and easier for me to live with arthritis (Woman 27 Indian age not reported).  ***Not offered referral to programs or professionals***  I was left in the dark and I had to go back to searching reliable websites. The Arthritis Society gave me the information. I don’t expect the doctor to have a long conversation but referring me to websites or referring me to dietician or exercise specialist or a massage therapist that would have really completed the picture… in some cultures some people have more fried or processed foods so the dietician or nutritionist has to get in touch with them and say, okay you’ve got to stop taking that, mellow it down. Those kinds of dietary changes which I don’t expect the doctor to explain but he should be open to say, I’ll refer you to a dietician down the road if things don’t improve or even initially it’s better to [refer] in the very beginning (Woman 18 Indian age 69).  [The doctor] should give me the [referral to] physiotherapist or specialist or a surgeon. That is was what I was expecting but she didn’t mention anything about that… she should give me this before when she heard that I have this type of problems (Woman 21 Pakistani age 65).  ***Doctor did not set expectations for follow-up care***  The doctor didn’t call me for my x-ray and ultrasound report. I called to my clinic and the assistant said your x-ray and ultrasound are there. And then I said I want to book an appointment because doctor didn’t call me… he should have followed up with me and ask me how your knee is? Do you still have pain? Is the pain more worse or you have less pain? (Woman 20 Pakistani age 44). | GOOD  ***Referral to programs or professionals***  If I need physio, [my family doctor] will send me to physio which is very comforting because physio has just that one specific issue you come with. You’re going there to deal with a particular ailment. So they’re prodding more because they want to get to the bottom of [the issue] and since it’s a sharp focus of the visit the treatment is much more beneficial than a reference to a doctor (Woman 08 Caucasian age 70).  POOR  ***Offered little to no self-care education, advice, or support***  Well [doctors] could have given me some options either an exercise program or I don’t know if there are medicines that I should take or dietary supplements; there was none of that support; everything I had to figure out for myself (Woman 02 Caucasian age 72).  Initially [self-care advice] was centred around activity avoidance… it was frustrating because [the doctor told me to] stop skiing, don’t run, don’t play soccer and as a 40-year-old person who coached a lot of their kids teams that wasn’t compatible with my lifestyle… from hearing other clients talk about [the doctor] and his team, I mean that [advice] was pretty standard. He was more about saying stop doing things, too bad for you, you got a shitty knee (Woman 06 Caucasian age 65).  I don’t find anything good about my OA care. There’s an acknowledgment from my medical doctor that there is OA and that I just have to manage the pain. There is no care…  beyond saying take Tylenol if the pain becomes worse, there’s nothing from the medical area (Woman 09 Caucasian age 70).  I don’t know what’s out there except for what I’ve read and everything I read is pretty superficial… I mean if [a website] says exercise, I can do that… I’m thinking to myself is there more to the care that I don’t know about and how would I find out about it. Maybe [my OA] is not bad enough but I know people who have very bad arthritis and maybe worse than mine, yet they seem to be in the same boat. We just grin and bear it. I wasn’t unhappy with my advice nor was I happy because it was just this is what it’s gonna be like (Woman 09 Caucasian age 70).  I feel like I’m left to manage [my OA] on my own (Woman 08 Caucasian age 70).  I have had lots of advice and expertise with respect to exercise and physical activity. I’ve had virtually no advice at all with respect to diet… Some people have diet raised with them because they are heavy and therefore they’re told to lose weight to manage their arthritis but that’s never been an issue for me. So I’m talking not about diet in terms of weight loss or diet associated with physical fitness. I’m talking about nutrition that will address inflammation. And for me with osteoarthritis that’s never been raised as something that I should pay attention to (Woman 11 Caucasian age 72).  ***Not offered referral to programs or professionals***  When there is an adverse outcome from a surgical procedure my experience has been limited effort by surgeons to investigate and refer me to another non-surgical professional other than a physiotherapist who might be able to treat the non-surgical issues [because] I’m in chronic pain…the ability of the surgeons to recognize that you obviously have a problem, your problem is beyond my scope of and areas of expertise, however I recognize that it’s a significant issue for you and therefore I’m going to find somebody who can potentially address the issue (Woman 11 Caucasian age 72). |

Barriers to accessing care

| Theme | Black | East Asian | South Asian | Caucasian |
| --- | --- | --- | --- | --- |
| Patient-level barriers | ***Women delay seeking care (unclear who to see, time constraints, other commitments)***  It’s difficult to navigate the healthcare system as to which specialist is responsible for what (Woman 03 Caribbean age 48).  Who [to see] at what point was my confusion. I’m not sure if there is a doctor that deals with arthritis. What kind of doctor would see you for osteoarthritis? Is that a general practitioner? Or is it supposed to be an orthopaedic? (Woman 24 Caribbean age 67).  ***Cost – no health benefits for therapy or cannot afford to take time off work***  If you don’t have extended health benefits some of the resources that you may need to tap into like physio or a brace is quite expensive. It can be a deterrent to getting that type of support (Woman 03 Caribbean age 48).  ***No family doctor to manage OA or refer to others***  Getting a referral from your family doctor if you don’t have a family doctor… I think it’s a huge barrier (Woman 03 Caribbean age 48).  ***OA dismissed by clinicians due to age, not considered serious, particularly if women do not self-advocate/ask questions***  I was not told that there would be any treatment based on my age. [Healthcare providers] just said, this is what it would progress to and if it gets really bad then you probably will be able to get treatment the older you get… there’s support when you get over 50 or 60 when you have OA… I think there could have been more care for those who don’t fit in that profile of being a senior with OA (Woman 03 Caribbean age 48).  I think sometimes people think that women are more resilient, or they can just deal with their pain and sometimes we are not that strong, and we need the care and sometimes moms need the support of the families and also the financial support would really help (Woman 07 African age 43).  Osteoarthritis is not a life-threatening illness, so it doesn’t get that much attention like every other disease that would be sort of life-threatening that would affect the main organs like your heart or your lungs. Because you can still function with osteoarthritis (Woman 24 Caribbean age 67).  ***Challenge adopting unfamiliar activity such as exercise***  Another barrier is participating in physical activities because sometimes it requires a trainer and you really have to spend a lot [of money]… and sometimes we are feeling low and sad and don’t even have the motivation to wake up and exercise (Woman 07 African age 43).  The change of lifestyle is not really an easy thing to manage… I was not ready to undergo the new lifestyle changes… I had to ask myself whether I’d be able to manage my new lifestyle including the weight loss journey and doing the exercises which is not my routine (Woman 15 African age 45). | ***Women delay seeking care (unclear who to see, time constraints, other commitments)***  Sometimes I just self-medicated… just because sometimes it’s my theory or thinking that oh [the symptoms] will just go away in a couple of days… because maybe it’s just part of getting older… but it keeps on coming back and forth [and getting worse]. So it’s just lately that I see my doctor about [my OA] (Woman 12 Filipino age 54).  Especially women, they need to take care of the family, and they put themselves in the last priority… sometimes maybe [women lack] knowledge or education and then they don’t know how to search the information or ask for help (Woman 16 Chinese age 62).  I don’t know any kind of resource that can help or any other health provider that can help because I don’t have this information… maybe there’s not enough advertising. I never aware we have this kind of care available to us… I know there’s a heart society and an Alzheimer’s society and Cancer Society but I never heard about osteoarthritis (Woman 14 Chinese age 67).  I try to learn how to treat myself and keep [my knee] healthier and [prevent it from] getting worse. I try to protect it but I don’t know how. I want to find out… I can search on the internet and search the YouTube but I haven’t got a chance to do it. And other than that, I don’t know how to get supports (Woman 16 Chinese age 62).  I don’t really know what other options I have. Except take my doctor’s advice going for physio and I do go for massage myself and then I do my exercise at home… a lot of my friends are saying that they don’t see my problem. They see that I’m a healthy person and I can do this and that. But I told them no… when people see me I’m not that bad so I tell myself that I’m not that bad. I still can go driving, go shopping, doing cooking at home, looking after my family, that is a necessity for me to do… but if I can get help to relieve my pain more then it’s even better (Woman 23 Chinese age 70).  ***Language barriers prevent help seeking or challenge communication***  A lot of my friends who don’t really speak English would be hesitant to go to the doctor because they can’t even describe what they’re feeling. For Filipinos, we tend to go to our friend who can do massage… it’s traditional Filipino… these are not professional, these are not licensed therapists, but we often go there if we feel something in our back… instead of going to the doctor, waiting for the appointment and then struggling to explain what you feel, we just tend to self-medicate (Woman 05 Filipino age 40).  Some of my friends do not go to the doctor because sometimes it’s getting way too long to wait… or they fear going to a doctor because they will not understand it… they resort to self-medication because they think it would be better but actually it’s not (Woman 10 Filipino age 48).  I think the English language is a barrier… like the way [immigrant women] get information and understand could be a barrier… and the cultural practices of each family… we may be not comfortable talking to the doctor (Woman 10 Filipino age 48).  Women of different cultures [tend to be] shy and intimidated to go to a hospital or to a clinic… because if you are new to a country…and then if you wanted to see a doctor it’s really different from where you came from. [In Canada] it’s really hard [for immigrant women] to explain themselves and they feel that [healthcare providers] get annoyed if they don’t understand them. So it seems like [healthcare providers] are not that patient or maybe they don’t understand the culture of the person (Woman 12 Filipino age 54).  For people who have language barrier I think it would be pretty hard because they have to struggle with the language and even talking to the specialist there could be a problem. So they may need somebody accompany them to the office… even all the medical jargon and medical knowledge and so they could feel a little bit left out or impersonal (Woman 17 Chinese age 66).  I work in an area that is a really high immigrant population, there’s language barriers as well to accessing [OA] services. Services are often not always available in their language of preference and even if there are print materials they are also often not translated into their language or preference (Woman 19 Chinese age 46).  Some people they don’t know English that well, they don’t understand… they don’t even know how to tell the doctors [about their symptoms]… they can’t express themselves. They don’t know any medical terms… they won’t get the care that they want unless physically you can see that they have problem then maybe the doctors will do something (Woman 23 Chinese age 70).  ***Cost – no health benefits for therapy or cannot afford to take time off work***  Other issues are even if [immigrant women] are referred to a public system they sometimes can’t afford to take the time off work. So things like paid sick days or vacation time could also impact a patients ability to access osteoarthritis care because if they’re choosing between putting food on the table and going to an appointment, they’re not gonna go to the appointment unless it’s something that is really dire or emergent (Woman 19 Chinese age 46).  [I am in a] rural area…some of us will go to <city name> [to access a doctor]… it will take an hour to go there and an hour to go back. It takes up your time. If we are working, we’ll be absent from our work and that’s not a good thing (Woman 10 Filipino age 48)  [The physiotherapist] advice is quite helpful, but OHIP doesn’t cover [physiotherapy]. I need to pay by myself. It’s quite expensive… and I lost my job… I can’t keep on go to see her. After we settle my major problem then of course I won’t continue… but this kind of treatment it help osteoarthritis… If I can go every week to do the massage I will be getting better. If I go to see the chiropractor, physiotherapy, acupuncture… I think I will be better too (Woman 16 Chinese age 62).  I’m not covered so I have to pay of my own pocket about $500 for injections. It’s a lot of money. If somebody don’t have the money they may not get the care to be honest… they may have to suffer more (Woman 17 Chinese age 66).  Many patients don’t have the financial means to go to a private provider. So things like physiotherapy, occupational therapy, often are not covered in the public healthcare system… it’s hard for [women] who can’t afford it to access the therapy that they need to keep themselves healthy and well and to keep their pain managed (Woman 19 Chinese age 46).  ***No or limited technology access or ability***  At some points, I experience a difficult time accessing medical doctors…It’s not really that accessible during pandemic to book an appointment with the doctor (Woman 10 Filipino age 48).  Because of the [COVID-19] pandemic I haven’t seen [my doctor] in-person. So we can’t talk too much…I can only get medicine or something, that’s it…over the phone, not really help… My family doctor tried to refer me to the specialist but I am still waiting… because now after pandemic it’s really really hard to find the doctor and get an appointment (Woman 16 Chinese age 62).  ***OA dismissed by clinicians due to age, not considered serious, particularly if women do not self-advocate/ask questions***  I don’t think [healthcare providers] really see [OA] as stressful…they just ask how do you feel today…then they just do the regular thing that they always do whenever I visit them (Woman 05 Filipino age 40).  [Healthcare providers asked me] nothing at all. It’s just brushed off because osteoarthritis is not really that important at the moment to be managed because it was just very mild (Woman 26 Filipino age 67).  [Healthcare providers] greet you, they tell you what are your problems… I’m not sure whether there is a limited amount of time that doctors should interact with patients but the care has really changed lately since 20, 30 years ago. Now when you go to your family doctor there’s a note on the examination room that only one question will be entertained during your clinic visit. So if you have other questions, you have to go back again in to see your family doctor and that’s unrealistic. So then you have to choose to which one is the most important for you during that clinic visit. Osteoarthritis is always the last that should be mentioning but now it bothers me because when you look at my hands it doesn’t look great anymore. It’s ugly looking…they could have said okay we are sorry that only one medical complaint that you can do right now. But maybe next time you should be focusing on osteoarthritis. We have to do something about it. Maybe we can also start referring you to some medical practitioners that can help you with your osteoarthritis before it becomes really bad. At the moment it’s not even a prevention, it’s just the band-aid treatment, take your medication or rub something on your hands if it bothers you (Woman 26 Filipino age 67).  Sometimes it’s so achy that it affects your whole person, not just physically, emotionally too… maybe I should talk to my doctor, because I have several family doctors… but none of them send me for any test. So they just suspect me having [OA] because I complained about my knuckles, my wrist, my shoulders, my knees, my elbows and the joint on my feet. And they just say that well maybe you have osteoarthritis. So I don’t know whether I do have [OA]… I don’t even know where there’s any test that can confirm whether I have it or not (Woman 23 Chinese age 70).  If the patient didn’t speak, like quiet person, then they won’t talk that much… maybe some [women] don’t know how to ask, they just stuck there and they just do the treatment and maybe just worry like my mom (Woman 16 Chinese age 62).  I think if women with osteoarthritis have some knowledge [about OA] then they would go to the family doctor and discuss and say that I really need treatment for my osteoarthritis because it’s just starting now and I don’t want it to become severe or more debilitating. Otherwise, if a person with ethnicity, color, comes to the family doctor and says I have osteoarthritis, do you think they will take care of you? No, [healthcare providers] will just tell you that [OA] is very common when you grow older… it’s part of aging. There’s nothing much we can do. You need a woman [that is] very informative of what’s going on with their body and osteoarthritis... what I really don’t like is those [women] that don’t have any knowledge about what osteoarthritis is about, so nothing will be done until it becomes severe (Woman 26 Filipino age 67). | ***Women delay seeking care (unclear who to see, time constraints, other commitments)***  But because it’s too late when I went to doctor. And I advised to other people if you have any disease, if you feel something different you should go to doctor at the right time… we get [pain] and we just take painkiller and get relief and then we move to work and we avoid these things but we should not avoid (Woman 04 Indian age 55).  Ethnicity [is a barrier to OA care] because South Asian woman that’s the taboo… if we take care about our family, that means that we are super mom, super wife and super woman. But if we care about ourselves, then we just receive a label that we are selfish… if you will do the research about the YMCA or fitness centre, you can see that very less South Asian woman go to the gym. Why, because there are issues or barriers related with bad comments… another barrier is when we have young children. How are we gonna take care of all our children because husbands are not really very supportive in South Asian culture (Woman 20 Pakistani age 44).  Sometimes [immigrant women] come [to Canada], they working too hard to survive and settle down and working hard for the kids…immigrant women don’t [have time] to care about their health…they work and they want to settle down kids so they ignore when they have [health] problem…so they don’t know [how] to take care of their self and they don’t know what is the cause of osteoarthritis (Woman 27 Indian age not reported).  ***Language barriers prevent help seeking or challenge communication***  [Immigrant women] have language barriers some of them are not able to converse well in English and they don’t get enough family support (Woman 18 Indian age 69).  For immigrant woman sometime there are language [barriers] (Woman 27 Indian age not reported).  ***No or limited technology access or ability***  Since COVID-19 my doctor still likes to see people on phone and if something’s wrong he wants us to send him pictures… he only sees patients in-person only for very urgent matters… so when you discuss things on phone like my knee hurts, I cannot sleep at night, I cannot walk properly it’s hard to tell where your knee hurts… but if you see a doctor in-person then he can touch where the pain comes from… if a person is in pain the colour on their face turns pale. [Doctors] cannot see [how much we suffer] unless they see us in-person… [doctors] should see their patients in-person and no more telephone calls only because that’s ridiculous (Woman 13 Indian age 67).  ***OA dismissed by clinicians due to age, not considered serious, particularly if women do not self-advocate/ask questions***  I feel that I can’t come over from my stress because my coping mechanism about my stress is exercise and I can’t do my work because of [OA]… and when I see my doctor he said that because of the age [I have OA] and then I said, no, I’m not that much old… he said that you had it before but you were not paying attention… [the doctor] said this is the problem you have now and you have to live with it. That was not appreciated… definitely when we would get older, then we will have some problems. But I am 44, I don’t think I should go with this problem (Woman 20 Pakistani age 44).  Male [doctors] can never imagine [women’s] daily routine or the way that we have to survive because of what we are juggling. Being a mom, being a worker, being a wife, so I think that he can never imagine my situation and he doesn’t know how much I will take a stress about [OA]. The scary thing is that if [the OA] will get worse and worse then how I will take care of my family and myself (Woman 20 Pakistani age 44).  I am personally feeling that because everyone is telling you are getting older… oh you’re getting old… somewhere, somehow I have to accept yes, my age is maybe, that’s why like that. But I saw the older friends of mine, they are pretty much good (Woman 21 Pakistani age 65).  If the process was faster at the family physician’s right from the beginning… if I got a referral to a specialist faster, then my knee pain wouldn’t have gotten to that extent that it is now (Woman 13 Indian age 67).  [Doctors] want people to discuss only one problem. If a patient has two or three issues it could be connected to each other but they said, okay then make another appointment about that. That’s very disappointing and it’s very ridiculous because if your head hurts it could be due to acid in your body, it could be some inflammation. It could be due to the related issue but they just want to know about one ailment at any one time (Woman 13 Indian age 67).  I don’t think many women of colour know that they should be taking [vitamins] to keep themselves healthy. Doctors don’t have time to do that. Doctors don’t tell you to start taking [vitamins] unless you ask them. Should I take any vitamins? Then they say, okay yah, you should be taking this. But they don’t initiate any such conversation (Woman 13 Indian age 67).  [When the doctor first] diagnosed me with osteoarthritis I didn’t have that much pain and doctor didn’t take [my OA] seriously… but now I am in [more] pain (Woman 27 Indian age not reported).  ***Cost - no health benefits for therapy or cannot afford to take time off work***  Some [immigrant women] can’t afford to go to the gym because the gym memberships are expensive. There is a financial barrier for sure (Woman 20 Pakistani age 44).  Not all immigrant women are working [In Canada] and most of us are not holding insurance and physiotherapy is not free. They don’t have money to spend on [allied health professionals]. So that’s why the women mostly don’t go to the [physiotherapy] and they suffer and they eat too much painkillers because it’s very expensive… our main barrier is insurance (Woman 25 Pakistani age 60). | ***OA dismissed by clinicians due to age, not considered serious, particularly if women do not self-advocate/ask questions***  What I do have is a lot of discomfort and pain and their response about that is, well just get used to it (Woman 01 Caucasian age 69).  Basically, [my doctor] just said, oh it’s only osteoarthritis and that was it. There was no support of any kind either medically or emotionally or mentally (Woman 02 Caucasian age 72).  I remember at the time they just said, oh it’s just osteoarthritis. It’s good that you don’t have rheumatoid. It’s a negative really that they downplayed it and they didn’t seem to take it too seriously. It was a very minimalizing experience that it’s just osteoarthritis and basically you live with it. There was no treatment offered (Woman 02 Caucasian age 72).  I don’t think doctors, unless they are a rheumatologist, pay a lot of attention to regular old OA… I almost get the attitude, well it’s only arthritis. It’s not well respected in the field… it’s just a sort of take these medications and learn to live with it attitude that I get… I think it’s a very underappreciated condition (Woman 08 Caucasian age 70).  I am wondering whether [OA] is taken seriously. Is that a barrier? Unless you’re crippled, the medical profession does not take any complaint as seriously… I don’t even get questioned about [OA]. They’re waiting for me to bring it up… I just don’t really see [OA] as a priority issue (Woman 09 Caucasian age 70).  I don’t feel like I have someone to really talk to about [my OA] because there’s so many other bigger problems that the doctors are dealing with… when I go see her, we are usually dealing with something that has a higher priority… the stiffness in my fingers and the pains in my legs aren’t necessary top of mind and I’m conscious of taking up too much of [my doctor’s] time. I’m usually dealing with cholesterol issues or other medical issues that I have… I’m left feeling I just have to live with OA and use the medications and advice I’ve been given over the years (Woman 08 Caucasian age 70)  I’m pretty proactive about searching things out and making a lot of phone calls and I got my surgeries really quickly and especially given [COVID-19] so no complaints in that. I mean I really advocated for myself. I called the hospital every single week to see if they had any openings. I don’t feel like I have an access problem because I push hard but if I was more passive about it, I would have a different experience (Woman 01 Caucasian age 69).  Eventually I had to have two knee replacements, but it was me doing the push, push, push all the time. I didn’t find the care from [healthcare providers] to be proactive. They were only responding because I was a well-spoken, educated person really pushing for the care that I needed. I read everything I could on the internet from reliable sources from different arthritis organizations… and I always feel sorry for people who don’t have those individual resources or some kind of mentor or guide so that they can read some things and figure out what they should do to support themselves (Woman 02 Caucasian age 72).  Because of my age, I would like to say gender as well. It was like oh nothing we can do for you…initially it was hard to get the knee looked at because I was young and complaining… [healthcare providers said] you’re not trying hard enough to bend it, you’re not doing the exercises enough to straighten it…they were convinced that I just wasn’t trying… now that I’m older it’s like what do you expect… I find it frustrating because one of the young doctors actually said, do you really think you should be skiing at your age?... in my experience with my male clients they very rarely tell them to quit their sport teams. Whereas I felt like everyone was very quick to tell me to quit my sport teams (Woman 06 Caucasian age 65).  With respect to the medical doctor it was ah, life wear and tear, aging… it was just this is life yes you’re gonna have issues and you just have to move forward… it’s part of the ageing process, suck it up (Woman 09 Caucasian age 70).  ***No or limited technology access or ability***  In the age of the internet, patients have a lot more access to information. I’m not sure that’s true in the immigrant population who are still probably struggling with all kinds of issues… for a woman in a less privileged position there are probably many obstacles from language to finance to understanding… I can imagine how hard it would for someone who’s trying to navigate in a new country and many times a new language without getting attitude… I don’t know how an immigrant women perhaps with limited language skills access information about self-management (Woman 08 Caucasian age 70).  ***Women delay seeking care (unclear who to see, time constraints, other commitments)***  Self-care is usually not really a high priority. Especially when you’re female because you’re looking after everybody else. That’s probably the real reason why women don’t take care of themselves as they don’t have the time… they put themselves last (Woman 09 Caucasian age 70. |
| Clinician-level barriers | -- | ***Little or no interpreters or translated take-home material*** Having patients who require an interpreter is sometimes a bit challenging because those appointments take a lot longer. it’s hard to give them the same volume of information in the same amount of time that we have because the appointment just goes slower. You just have to wait for everything to be translated. Also not having materials translated into different languages for patients to take home because we know patients don’t remember much of their medical appointments in general (Woman 19 Chinese age 46) | -- | ***Clinicians lack knowledge about OA***  The first line of interaction by a patient is likely with their family physician. In my experience and on their own admission, family physicians have virtually no competency in arthritis and in orthopaedics and as my family physician of many years who’s now retired said to me, I think I had 3 hours of orthopaedics in medical school and he said, you’ve learned more than I’ve ever known in the years that you’ve been a patient and I’ve learned more from you than I ever learned in medical school… with my current family physician he’s happy to refer me to whoever I think I’d like to be referred to but does not have the expertise to refer to anybody other than a physiotherapist (Woman 11 Caucasian age 72). |
| System-level barriers | -- | ***Shortage of healthcare professionals or services prioritized for conditions of greater severity***  It’s very hard to book an appointment here in our area. It’s overpopulated for a small town… you can’t get [an appointment] immediately… I think we only have two chiropractors here and there are a lot of people here that experience back pain because most of us are working in the plant. The work is very hard. When we want to book an appointment it’s always fully booked… even a lot of us don’t have family doctors here (Woman 05 Filipino age 40).  The serious thing is access to doctors. The doctors are really scarce here, they don’t have doctors here that can cater to the population (Woman 10 Filipino age 48).  [The doctor] says that anything that you don’t feel good about yourself, you have to see a doctor right away. But the problem with that is sometimes you won’t see your doctor just because it’s hard to schedule an appointment because we are lacking doctors here… so what I did when I really felt that pain, I went to an emergency so that somebody will see me (Woman 12 Filipino age 54).  [The doctor] could have [given more information] but she’s so busy… if I book today, maybe 2, 3 months later I get the appointment, very difficult to get a doctor appointment… we don’t have enough family doctor everywhere (Woman 14 Chinese age 67).  I would assume something as minor as finger joint pain from osteoarthritis likely wouldn’t be of higher priority if I were to be referred to the public system to a hand clinic for splinting. It was bothersome for me. I wasn’t functionally disabled but I suspect that if I had gone through the regular route, then it would have taken longer for me to be seen… these hand clinics in hospitals deal with much more acute and functionally debilitating injures as well as post-op so these patients are much higher priority. So that was partly why I went to the private system because I didn’t want to burden the public system (Woman 19 Chinese age 46). | -- | ***Shortage of healthcare professionals or services prioritized for conditions of greater severity***  Right now, you’d feel quite guilty to go and see your doctor about your arthritis unless you’re crawling… you’d have to feel very severe about your condition to go and bother your doctor now with it because medical resources are scarce (Woman 02 Caucasian age 72).    I think there’s a lot of women who are suffering in silence… you can’t go to emergency for stiff fingers. People are forced to go to emergency with things that normally would be handled at an urgent care clinic or something, but they are not available… 25 or 30 years ago when I first entered the healthcare system with arthritis as an issue it was a different world. I probably saw a rheumatologist within 2 months. It was very easy to access a specialist, and I don’t think it is now… I know the resources available are very limited… the wait times in emergency are horrendous and people can’t get a primary care physician… doctors are retiring and leaving their practices and there’s no one to take their place or practice or patients… it’s a real crisis in <city name> now… people are not even getting the basic information about how to manage their OA. I would think people are probably stuck and living with their pain without resources… that’s when we all say, yah the healthcare’s pretty broken (Woman 08 Caucasian age 70).  It can be very difficult to get buy-in or certainly funding for osteoarthritis care or programs just because it’s not gonna be perceived as being a big concern, it’s just arthritis (Woman 08 Caucasian age 70).  ***Long wait time for tests or referrals to specialists***  When they finally decided that my knees were severe enough for a knee replacement, I waited a year to see the orthopaedic surgeon. Then I waited a year to have the first knee done and I waited 9-months for the second knee to be done and that’s 13 years ago and I understand the waiting periods are much longer now (Woman 02 Caucasian age 72). |

Strategies to improve OA care for diverse women

*What should be done to ensure that diverse women get the osteoarthritis care and advice they need?*

PATIENT-LEVEL

Offered to persons with OA to improve knowledge, confidence, behaviour, OA symptoms, OA status, or quality of life

| Theme | Black | East Asian | South Asian | Caucasian |
| --- | --- | --- | --- | --- |
| Offer education sessions about OA and self-management to persons with OA (women-only, group, in-person and virtual, multiple languages, across Canada, free, at workplaces or community centres)  Offer educational material (brochures physicians can hand out, posters in community settings and online, in different languages, include culturally relevant information) | ***Meetings in community settings (multiple formats)***  Information sessions being available that people can go to on a monthly basis, quarterly basis… where you can talk about things that affect the community and have those in the communities itself. And also having it available in various formats virtual as well as in-person… if you have someone talking about weight-loss then you know that there’s some type of link to osteoarthritis and weight-loss… more information sessions, more information available in community groups could be useful to guide people as to how to manage [OA] and what are some of the things that you have to do (Woman 03 Caribbean age 48).  ***Instructor should be a healthcare professional***  It could be having local healthcare nurses or whoever it is that could advise you on [OA] symptoms to look for… I think it would be useful getting the information directly from the health advisors or healthcare providers because right now we’re getting the information on Google and we’re not sure the source of that information (Woman 03 Caribbean age 48).  [Doctors and nurses] can hold a seminar or conference [in the hospitals] whereby they can host a group of women patients who are in the hospital… even once a month whereby they give advice on nutrition and also advising women to do the exercises. Somebody will most likely take advice from the doctor more seriously (Woman 15 African age 45).  ***Information or resources delivered by healthcare provider***  I think there can be more information [about OA] through pamphlets… when you’re sitting in a general practitioner’s office or a doctor’s office or going into the hospital… And if you’re feeling [OA symptoms] what should you do you navigate the system itself (Woman 03 Caribbean age 48).  I would see a family physician and then they will recommend physiotherapy. You’d go to physiotherapy for a while, 6 to 8 treatments and that helps. And then it’s a year, another two year passes and then do you recommend more physiotherapy? Right now I would like to go to the gym… but I don’t know to get a physiotherapist to help me again with exercises. I think that’s the cure right now for the arthritis, you just to go physiotherapy. There’s nothing else…then stuff you read somewhere says if you have arthritis you shouldn’t eat cheese, tomatoes, potatoes, eggplants and… I think if there was general information out there coming from your physician or people who are knowledgeable in the field… in terms of your diet if it helps your osteoarthritis. The exercises that [can] help rather than just surgery… just a little extra information on [different ways] to manage OA (Woman 24 Caribbean age 67). | ***Meetings in community settings***  If one of the chiropractors in <city name> would visit the women’s group there and explain to us [how to manage OA] instead of going to the doctor and ask your question, you can ask your question and the entire group will be able to learn from the question that you asked (Woman 05 Filipino age 40).  To have more local group like <city program name> would be a big help because they can always invite women to join them in conversation circle and then they would engage them in how to cook or prepare food that are healthy (Woman 05 Filipino age 40).  I think the settlement office should invite immigrant women and then there’s someone there from the health office to talk about healthcare and about the osteoarthritis… an entity or a government office who will invite these women and then invite healthcare provider to talk about osteoarthritis. And then they should ask women’s opinion [about] how they feel or if they are so shy talking about [OA], they can just email or they can talk to the office who’s doing the presentation for help (Woman 12 Filipino age 54).  ***Information or resources delivered via community organizations***  It would be good putting posters in designated areas and handing out leaflet in the immigrant settlement services for people of colour… I notice lots of immigrant people of colour are coming to the immigrant family services to access information… settlement providers can make them aware of osteoarthritis and link them to medical providers (Woman 10 Filipino age 48).  I think it’s better to have an organization like the Arthritis Society, Cancer Society, and then from there you can educate the people and provide help or give information. So that people can be more aware of [OA] so then they can take care… at the earlier stage. Preventative better for you than curing. It’s harder for older people [or those already suffering] to manage but information for them I think is also necessary (Woman 14 Chinese age 67).  I think [OA] education should be available through [community] organizations that deals with ethnic groups… that [information about] osteoarthritis is very important, that the care should start while the symptoms are mild and to ask your doctor to refer you to a specialist or other medical practitioners that can help you with your osteoarthritis before it gets severe. So the dissemination of information is very important particularly to these women of [different] ethnicities… flyers can be done like for example, do you want to know information about osteoarthritis? Are you able to access computer and learn about osteoarthritis care? Maybe leaflets that you can provide to the different organizations so that when women come into the centre they will be able to look into it and says, oh I have osteoarthritis, I think I’m starting to have symptoms, maybe I should check with my family doctor so that I can start my care (Woman 26 Filipino age 67).  ***Instructor should be a healthcare professional***  It would be better if [information about OA] comes from a doctor or a specialist because for Filipinos, we always take doctor’s or specialist’s advice seriously compared to a women’s group in the town but the women’s group is more engaging so it’s well attended compared to going to a doctor (Woman 05 Filipino age 40).  [Healthcare providers] should give [immigrant women] healthcare awareness about osteoarthritis. For example, some information sessions about osteoarthritis [symptoms] and about self-care… to understand if it is serious or not and learn to take care of ourselves as we grow older… there should be someone there who will translate in their language so they fully understand about it (Woman 12 Filipino age 54).  ***Information or resources delivered by healthcare provider (multiple formats)***  I think when the family doctor finds patients that have [OA], maybe they can have the kit with different information or journals to read about how [women] need to take care of themselves in their daily life and what they need to eat… if it can be on the internet because women they need to take care the family and everything. So they can’t go out very easy. So a convenient way that they can assess those kinds of information can definitely help them know more about their condition and cope with it (Woman 16 Chinese age 62).  I prefer if [healthcare providers] can send me the link or some brochure and then we can study by ourselves… if [the information material] can list somebody to call to answer our question that would be nice too like a professional or the nurse that know more about OA so they know how to answer our question… maybe more efficiency, than call your family doctor (Woman 16 Chinese age 62).  If there was any information on-line or whatever that I can get it, it would be helpful for healthcare providers to send me that… because of my pain I want to learn more about osteoarthritis. What the symptoms of it are, what the signs of it or what can we do to improve our daily lifestyle… more basic educational information… some tools that you can buy to help me to do some daily chore…maybe I don’t know how to search it myself. But I don’t see any of those information on the websites.  (Woman 23 Chinese age 70).  ***Information or resources delivered by healthcare provider (multiple formats, languages)***  If there’s education material… maybe a video or something like that… in [different] languages that will be helpful, so that [diverse women] have a better understanding. Maybe have a plan for what’s next like what would we expect let’s say in one year and 5 years or 10 years you know what should we be doing so it can help us cope with that better (Woman 17 Chinese age 66).  ***More support from healthcare provider to manage OA***  [Healthcare providers] are so busy… I just hope they will maybe have more sympathy towards the patients and do a little bit more education or motivate [patients] to do better self-care. Or maybe refer more to the physiotherapist or occupational therapist, then they know how to deal with [OA] better. Because I don’t think a doctor or nurse have all the answers, so at least they can refer a little bit more. Or have some healthy tips or information and so when the patient visits them they don’t feel so alone (Woman 17 Chinese age 66). | ***Meetings for women in perimenopause or menopause***  Beginning when the women go into their menopause or pre-menopause there should be some consultation session on-line or in-person from doctors or health authorities on what to expect or what women should be doing to take care of their bone health like taking the vitamins or minerals or whatever is needed for making your bones strong. And even on things to avoid [to prevent OA] … many, many women they don’t know that menopause can cause your bones to get weak or how to make your bone health better (Woman 13 Indian age 67).  ***Meetings in community settings***  There’s no knowledge, no information, no education about [OA]. My children have celiac disease and there are workshops available and there is some psycho educational and groups program that are available. [At work] we had education session about dementia and Alzheimer’s disease. I never heard about that there are those kinds of things available in our community for awareness of arthritis… we should have something because arthritis is a problem. But how it will affect your daily life? How you will take stress? … my suggestion is that we should have community awareness workshops or information sessions about arthritis as we are having dementia, schizophrenia and PTSD (Woman 20 Pakistani age 44).  ***Meetings in community settings (remote areas)***  The Arthritis Society of Canada should have their volunteers present to us and to go to the various community, including the remote communities in B.C.…so reach out to them and speak to them and explain how arthritis can be treated…and the doctor should be respectful and delve into self-care, not just talk about conventional medicine (Woman 18 Indian age 69).  ***Information or resources delivered by healthcare provider***  The doctor can introduce [women with OA] to websites, say the Arthritis Society of Canada… they have webinars and there are speakers that are doctors talking about pain and how one can improve their joints. They have an exercise webinar where the fitness instructors come in (Woman 18 Indian age 69).  [Immigrant women] need resource and health education, health seminar, health workshop [from healthcare providers], so they can get advice and care from there because doctor in Canada, they don’t have much time and then they don’t want to do much. In Canada, the doctor just prescribe medication (Woman 27 Indian age not reported).  ***Sessions delivered by healthcare professionals***  [Immigrant women] didn’t get any special teaching over here and if you go to the doctor… in the early [stages] they should teach us. They should tell us about [OA] like if you will do this and that, you can feel better or you can suffer little bit less… maybe [healthcare providers] will explain how can you keep yourself [in] good condition. Maybe we can know if [OA] is genetic too and it will move in our kids. So maybe when they teach us, we can teach our children and [prevent them] from getting this disease… most of our women in my age they can’t get access to internet because lack of education there is a big difference from here and our background. So they are not in touch with the Google, with the internet, even most of us maybe don’t have email addresses. And if they have email, they don’t bother check what is this. If they will check, it’s very hard to read because the language barriers. So mostly [immigrant women] prefer to go to that doctor face-to-face who can talk in our own language. We can explain good to them, and they should explain to us. And I think it will be much better (Woman 25 Pakistani age 60).  ***Information or resources delivered by healthcare provider (multiple formats)***  I think that the most important thing is education and awareness about osteoarthritis… I think these are the strategies the health providers should consider… for example, if a patient visit the family physician, that family physician can talk about basic information about the osteoarthritis and the options [for treatment or management]. In the internet there’s so many resources over there, maybe some support groups, so that healthcare provider can guide the patient to resources, so the patient can research about [OA]. And also some flyers or handouts. By using the multi-modality [approach] to educate the patient because for me, education is the foundation for any chronic disease (Woman 22 Pakistani age 57).  ***Information or resources delivered in the workplace***  There should be some help available [in the workplace] … there should be information given by companies to their employees or staff like how your health can be kept or how you can be safe from osteoarthritis and what measures you should be taking (Woman 13 Indian age 67).  ***Instructor should be a healthcare professional***  Experts should [provide] information, the opinion are more better… I prefer to get solid information [from healthcare providers] about any of my problems to understand better. Or about how to handle these things (Woman 21 Pakistani age 65). | ***Meetings in community settings***  The Nova Scotia medical system has public programs on various illnesses or situations. I know they have some mental health ones. They have parenting ones. They have ones for dieting and exercise. I would like to see programs in there that members of the public can attend and have them in communities. In Nova Scotia a lot of people of colour live in more, not isolated communities, but they live together in communities… I would like to see those kinds of programs for everyone, so that everyone is comfortable going in their own community for these programs because a lot of the advice that can be given doesn’t have to be individual medical advice (Woman 02 Caucasian age 72).  I think providing education… exercise, stretching, postural awareness, diet, and calcium intake… in cultural community centres for women of color (Woman 06 Caucasian age 65).  I think community programming… I can go online and look for myself but sometimes searching is not that easy and you don’t usually get something local… I don’t want something from somewhere else. I want to hear what my local medical community has to say… preferably in-person [information] sessions because I get more out of it. I’m engaged more (Woman 09 Caucasian age 70).  I think work within the structures that are in place… perhaps shift the focus to community agencies whose mandate is to deal with immigrant female populations where immigrant women are already gathered for language skills, resume writing skills, childcare… recruit immigrant women who are already in treatment for OA and expand programming to give them access to dedicated professionals… more physiotherapists and more nurse practitioners and more patient peer advocates to offer lectures, workshops or sessions to give information [about self-management] and give sympathy and understanding… let immigrant women who are struggling with arthritis know that they don’t have to suffer in silence, that there are things that can be done…we need to create a system where this under-serviced group of women are taking ownership or given the tools to take ownership of their arthritic health (Woman 08 Caucasian age 70).  ***Resources or sessions delivered by healthcare provider (multiple formats, languages)***  Video exercises are nice like types of exercises something that’s easy to follow and educating people on appropriate diet choices, like portion size and cutting certain foods out of your life. And I think that could be done on internet or video or pamphlets. A lot of people in my cohort probably more so than younger people like to have [something] in hand to look at and refer to… but I feel like doctors and physio’s and nurse practitioners could direct people to those sources but not too many, it can be overwhelming if you get too much (Woman 06 Caucasian age 65).  If there are resources out there, people have to be made aware of their availability…then you’d have to look at each of those groups and issues [they are facing] and say is that information not accessible to the person because it might be available on the internet and depending upon your ability to browse, you may not find it. Or sometimes you may have to be guided. So perhaps availability of an online webinar that someone can go through in their leisure if they have access to online services. And for people who don’t care to do on-line anything all you can do is make information available in different forms and then it’s up to the individuals to do what they have to do to get the information but it’s got to be accessible (Woman 09 Caucasian age 70).  ***Instructor should be a healthcare professional***  I like it when [education] comes from a trusted authority… someone that seems to be involved in women’s health whether it’s a physiologist that works with people with movement disorders or a medical doctor that’s involved with prevention and care of arthritis or even a fitness instructor that has knowledge or can sight sources… I like it when they can say, I have this information, yes it’s beyond my scope of training but this is where it comes from. I feel like having a trusted source is important (Woman 06 Caucasian age 65).  ***Questions prompt tools***  Medical brains could draft a list of questions that a doctor may ask the person with osteoarthritis in subsequent visits… questions about what to look for and to know how to report back if things are getting worse… the patient would then get that list. That way the person with osteoarthritis, knowing what questions in advance the doctor may ask, could properly prepare for future visits (Woman 09 Caucasian age 70). |
| Consider patients’ cultural needs and economic circumstances when offering treatment, or self-care advice and/or programs  (language, interpreters, cost of services such as physiotherapy) | Often times when you start experiencing [OA] in certain diverse communities you don’t necessarily want to take the pharmaceutical stuff… Maybe if there could be healthcare providers talking about alternative medicines (Woman 03 Caribbean age 48). | If the doctor would feel that the patient does not understand what he’s talking about, he should request for an interpreter or else the patient won’t understand what the doctor is talking about (Woman 05 Filipino age 40).  Some of the immigrants here don’t have access to interpreters… I think it’s very important to listen to the details of what the medical professional is saying… [immigrants] can avail of the interpretation services provided by the immigrant settlement offices in the area so they will be confident enough that there will be somebody who can translate to them and improve understanding between the medical professional and the client… if [doctors] can link patients with interpretation services offices especially if they have clients with other cultural backgrounds they can better discuss osteoarthritis (Woman 10 Filipino age 48).  If we go to a certain hospital or a clinic they must have an interpreter because even though you know how to talk in English sometimes it’s really hard for us, like different cultures, to explain how we feel… it’s not only me, there are some other people who doesn’t want to go to a doctor sometimes because it’s really hard for them to explain how they felt… because of the language barriers (Woman 12 Filipino age 54).  If [there’s women who don’t speak English well], I would say hopefully there are other people who is willing to volunteer to translate their concern to the doctor in English. Then maybe they would get better care (Woman 23 Chinese age 70).  Populations that do not speak English as a first language perhaps may have distrust with the medical system, having longer appointments to develop that rapport and maybe follow up to develop that rapport and trust I think is really important because just seeing patients once, throwing a ton of information at them I don’t think necessarily it’s a very good experience for really any patient. So I think there needs to be more culturally sensitive care and more care that is sensitive to the needs of [culturally diverse] populations and the challenges like the cultural differences, the distrust, the language barriers, etc. that they may face within the public healthcare system (Woman 19 Chinese age 46). | People from different cultures don’t embrace conventional medicine right away. Some of them like to do self-management… like essential oil massages and put a hot compress, [the pain] will go. That kind of attitude is there… the doctor should touch upon massage, chiro, and aqua-fitness exercises... the doctor needs to be open to that and not say, you need to take a Tylenol, nothing else. So it should be a balance between the two (Woman 18 Indian age 69).  I would recommend if [immigrant women] don’t have a family member who can communicate to the doctor about their problem then I think one could have a nurse or a bilingual translator or another healthcare provider when an immigrant woman says I’ve got problems speaking, I’m not very fluent speaking in English (Woman 18 Indian age 69). | I have arthritis in one foot and lots of times they’ll say, walking is the best exercise. But then if you have arthritis in your foot, you don’t feel like exercising, so what’s an alternative. So looking for alternatives to ease the symptoms and take care of yourself other than the advice of just take a Tylenol (Woman 02 Caucasian age 72).  I feel like you should never take away hope. I’m more a fan of modifying activities as opposed to saying stop doing this or stop doing that. What I think would be helpful is if [healthcare providers] would explain that things have a cost. You can do that but you’re probably gonna have a swollen knee and it’s gonna hurt for a couple days. Do you want to pay that cost? How important is that activity to you? I feel like that would be a helpful approach for people like me. Rather than just saying don’t do it (Woman 06 Caucasian age 65). |
| Offer peer support groups to help with self-care (virtual and in-person, multiple languages) | Patients can be taught [OA self-care] in an on-line group. Maybe a group where there is a lot of people who have the same condition and sharing the same experiences, I think could serve us well (Woman 07 African age 43).  I think that women should come together and form groups whereby they carry out their daily physical exercises and encourage themselves… it can be face-to-face to do exercises together. It can make it more fun and interesting being in a group of people… so you don’t get tired and you get pushed to keep on doing the exercises… and also have the talks about the osteoarthritis experience. Already experienced women can advise the others who maybe are developing the symptoms or even those who do not have the symptoms… women can learn more about care and improving the symptoms of OA… it can be on-line (Woman 15 African age 45). | It would be great to have [peer support groups] we can link up to the, let’s say osteoarthritis society and it could have different cultural groups or something like that… some [women] who suffer from [OA] can take the lead to form a society and then other people can join together… we can learn from them and then see what they eat or any supplement maybe should be taking or any kind of exercise we can do or what kind of fun activity we can do to support each other too… that can benefit everybody because I’m sure that this problem affect the population quite a bit and we can all benefit from it… the hospital may have some support groups because they have the facility there and they could have the physiotherapy or occupational therapist there too… I’m feeling alone and I don’t know many people out there that need the same support. So it really could be helpful to have a channel that I can join a society or support group (Woman 17 Chinese age 66). | I think it should be a peer support group or psycho-education groups. We should have an expert in [OA] and others who are suffering from this issue, who are managing [OA] themselves…information or advice should be offered and there should be psychological and physiological support…the peer support program should be language- and culture-based based (Woman 20 Pakistani age 44).  I’m thinking about not only arthritis, but any other disease or health concern for women’s health…what to do about the healthcare?... It’s better [if its in-person] some kind of group program because on-line I can only read and you can get so many different advice and you don’t know which one is the right one… [in a group] you can get idea what other people health problem, what they do, how they care of themselves, how is doctor helping them (Woman 27 Indian age not reported). | I would love to see dedicated services or organizations for immigrant women to include some of these healthcare issues that the healthcare system isn’t able to address… like peer-to-peer support. Women who are struggling with arthritis and have found ways to get around it can share that with women who are just starting to have mobility issues (Woman 08 Caucasian age 70). |
| Regular follow up from doctors to monitor OA symptoms and self-care | -- | When there is a lot of information to absorb and you’re working against cultural and language barriers, patients need to have more than one visit. They need to at least be able to be offered the option of a follow up to check in on them, make sure that they are actually following recommendations. After you see them once and you send them off, did they actually absorb anything? Are they actually able to incorporate any of the [recommendations] in their daily life (Woman 19 Chinese age 46). | I understand the doctors have tons of patients but they need to have something in the system… some sort of a pop up saying this patient is due for a follow up… in people who have pain or pain is getting worse and they have some questions, the follow up would be a big step in giving [patients] psychological relief or comfort and at the same time the doctor would be able to assess how the situation is. Is [OA] becoming worse? Does it require more medication or change of prescription or what?... if an [immigrant] lady doesn’t know what to do and she goes into taking the wrong medications or doing self-medication, follow up for said people is a must (Woman 18 Indian age 69). | -- |

CLINICIAN-LEVEL

Offered to healthcare professionals to improve knowledge, confidence, behaviour, or how they provide OA care (e.g., skills)

| Theme | Black | East Asian | South Asian | Caucasian |
| --- | --- | --- | --- | --- |
| Medical or continuing education on diagnosing and managing persons with OA | Maybe some money could go into educating healthcare providers on how to do more preventatives [on OA] rather than just say, oh yah sit around and wait for a knee replacement (Woman 03 Caribbean age 48).  [Healthcare providers] should also be digging in to learn more about the state of osteoarthritis… they should always be doing their research and writing down the new symptoms that each patient brings (Woman 15 African age 45). | Training clinicians out in the community to better provide osteoarthritis care. Providing training courses for community-based therapist to boost the ability for community to manage [OA] patients could be an option. The Arthritis Society provides training courses and that’s something that I think could be augmented (Woman 19 Chinese age 46).  Education through for [healthcare providers]… putting posters to their clinic rooms can remind doctors that oh, if my patient is talking about osteoarthritis what should I do? Should I give them minimal information? Or should I do some tests now? Offering those advice to the doctor [so the doctor can inform] the patient (Woman 26 Filipino age 67). | Maybe [doctors] need more education and training especially in arthritis. Because there is no treatment for arthritis and arthritis is very painful and it is very common (Woman 27 Indian age not reported). | As a general principle the competency of family physicians as the first line of contact about the understanding and management of osteoarthritis needs to be increased. The competency and willingness of surgeons to facilitate proactive follow up by other health professions when there is a post-surgical problem or a problem that they can’t address as surgeons is very important… joint replacement is not the end of osteoarthritis and there needs to be on-going management and expertise to deal with [OA] (Woman 11 Caucasian age 72). |
| Education or training on providing person-centred OA care tailored for diverse persons | ***--*** | [Healthcare providers] could train about how to take care the patient and [address] their emotion or their feeling. I don’t know whether they are training or not… but I hope they can have this kind of training and can input this element in their treatment, that would be better (Woman 16 Chinese age 62).  [Healthcare providers] should have cultural awareness training for them to understand different cultures especially for women (Woman 12 Filipino age 54).  I think cultural sensitivity training is important [to care for diverse women]. I work in an area where there’s a lot of South Asian immigrants and there’s definitely cultural differences, I have noticed in how different cultures interpret pain and manage it. I think recognizing that when you’re working with cultures that have a stronger belief in alternative treatment that aren’t necessarily evidence-based for OA or tendency to lean more towards passive therapy, for example, these populations require more education and perhaps more time for that education. And they need resources in their own like native languages for that education to be effective… so incorporating some training about how different cultures foresee and manage pain and being able to have public resources that are in different languages for [diverse] women is I think really important (Woman 19 Chinese age 46). | I would say we should have ethnic appropriate advisors and they should know about the issue in [South Asian] culture, right? If we go to the Caucasian [healthcare provider], then she say, okay go to the gym. But what are the barriers for us that [prevent] us from going to the gym? ... culturally appropriate training sessions [for healthcare providers] would be more appreciated (Woman 20 Pakistani age 44).  It’s difficult to know about the different cultures but generally speaking [healthcare providers] should know about and respect whatever the barriers are. They could be education, transportation, or stigma against a certain [health] problem. I’m not expecting the doctor to delve into that in detail but just be aware and be respectful of lifestyle changes, management and some holistic treatment which really work (Woman 18 Indian age 69).  I think the education of doctors is also very important because [Canada] is multi-cultural. So I think there should be an education of the doctors about how to interact with different cultures because people from different cultures have different beliefs regarding the treatment and I think there’s a sensitivity around these things. I think the health providers need to be very aware of the culture sensitivities and they should have thorough information on this so [patients] can have a trusting relationship with physicians, [which would] help with compliance [and will make] treatment more beneficial… if patient is not having trust with the health provider or health provider don’t know how to communicate or how to interact in regard to the sensitivities that would be really not good for the patient and her health… it’s not kind of like one to two year or one class, it’s an on-going process (Woman 22 Pakistani age 57).  [Healthcare providers] need more training on the immigrant women… they have to learn how to deal with immigrant women because immigrant women background is different than [in Canada] (Woman 27 Indian age not reported). | I think it would be good if doctors or their teams learn more about the person and not just looked at the x-ray… the approach needs to be more holistic (Woman 01 Caucasian age 69).  I think everybody should have diversity and inclusion training to understand more about how people are the same and different in so many different kinds of ways (Woman 01 Caucasian age 69). |

SYSTEM-LEVEL

Developed and/or offered by health systems or government to improve access to OA care, advice and support

| Theme | Black | East Asian | South Asian | Caucasian |
| --- | --- | --- | --- | --- |
| Health promotion or public health campaign to raise awareness of how to prevent and manage OA  (available in different languages) | [Education] could be disseminated through digital marketing. It could be through advertisements (Woman 03 Caribbean age 48)  What I can say on health education is the government can possibly have programs and health promotional activities on osteoarthritis so people can be aware of this condition… I feel what can be taught is some program or courses, like what caused someone to get this condition and help people understand the symptoms and how to better manage it. I feel that information can be useful (Woman 07 African age 43).  We need education… make sure that people know what are the root causes of [OA] and how to prevent it… make sure that they target everyone it can affect… say osteoarthritis is not only for the old. When you’re young, you can do “x, y, z” [to prevent it]… and make more information available… even the gym teacher in schools with kids can make sure kids wear proper footwear… inform people early, inform athletes, people who are active on their knees, workers who do jobs that [can affect] joints… if you start early education then when you get older you won’t have this. It’s like what we do for smoking… if you keep smoking for a long time it’s not gonna be cool anymore. We do all sorts of advertising for everything else so we can do it for osteoarthritis too (Woman 24 Caribbean age 67). | I think it’s better to have an organization like the Arthritis Society, Cancer Society, and then from there you can educate the people and provide help or give information. So that people can be more aware of [OA] so then they can take care…at the earlier stage. Preventative better for you than curing right? It’s harder for older people [or those already suffering] to manage but information for them I think is also necessary… I think through advertisements, and like if all kinds of society they can handle some free sessions, zoom sessions to talk about how to handle these kind of things. How you can reduce your pain? How to do exercise? What support you need and all kind of foods you should avoid (Woman 14 Chinese age 67).  I think education or more information on prevention in our younger age to take care before [OA] happens. I haven’t got any information for prevention for the osteoarthritis… maybe Public Health, maybe they can give us workshop and people with interest can join free of charge to let people know how to prevent [OA]… and promote this on the TV, YouTube or internet (Woman 16 Chinese age 62).  ***Include different languages***  Have more group classes. They could be on-line. They can be even just a recorded module or video that’s available. Something that’s readily accessible for people seeking more information about their condition. I find having in-person classes with an interpreter are really engaging… they’re interactive, people can ask you their questions and [healthcare providers] can address the specific questions that people have which often are common to their experience… I think more public education would be really, really useful that’s readily accessible, interactive, in [different] languages, and doesn’t take a lot of time to do because again [diverse] populations don’t necessarily always have the resources or the time to take off half a day to do a course in osteoarthritis. Having flexible times to access these educational modules or classes would be great (Woman 19 Chinese age 46).  The healthcare system which ever department is responsible for [health] promotion… should promote [OA] more so that people who have that kind of problem can get more information about it… you can [promote] it on social media, Facebook or Instagram, TV… you can tell people that you can go to this site or you can phone this number to get more information if you are interested… right now I don’t see any. So I don’t know where I can go (Woman 23 Chinese age 70).  Maybe advertise [OA] on TV about osteoarthritis care so that everyone sees it and that would be national or global… it’s just the same thing as the diabetes, asthma on TV. What treatment do you need? What are the symptoms? If a lot of people sees it then they’re all aware that oh maybe I have osteoarthritis (Woman 26 Filipino age 67). | Awareness is a main part… many, many people are not aware how to manage their condition but they just take medicine… this awareness or education plays a vital role… they can be educated through some advertisement on TV or on phone, on Facebook because these things are electronic and more popular in South Asian countries…it is better to educate [women with OA] through these tools… by the government or hospitals (Woman 04 Indian age 55).  ***Include different languages***  I think [healthcare providers] should [provide pamphlets or information brochure about OA] translated into different languages for all women of colour… so women can read them and they should be made available at every doctor’s office, at a medical clinic and community organizations… providing information widely and broadly in different languages can help women stay healthy from osteoarthritis. (Woman 13 Indian age 67). | I think it would be great if in society in general there was a lot more about prevention rather than pain management and if there was a lot more education on diet and exercise… I think public education campaigns are great. Think about COVID and wearing masks and getting vaccines and how to wash your hands properly. People may choose or choose not to follow the advice but it’s pretty hard to escape at knowing it right? ... maybe if [older person’s] lifestyles were different, arthritis would be much less of an issue just like if obesity wasn’t such a big problem diabetes would be much less of an issue… the more we can understand how to better take care of ourselves and our bodies in the long run I think people are gonna be healthier (Woman 01 Caucasian age 69). |
| Implement dedicated OA clinics | -- | -- | -- | I know they have diabetic clinics. Maybe they need to have an arthritis clinic that’s the same kind of thing… it would be something outside the normal physician/doctor office either through Arthritis Society or some medical centre offering a specific arthritis clinic that you can go and have your questions answered or learn how to minimize your symptoms. When I get very bad arthritis in my fingers what can I do? ... [the centre] would be multiple disciplines so they could be allied health including a physiotherapist, massage therapist, dietary, nutrition, exercise, kinesiology… it’s something I’m sure could be run by a nurse or nurse-practitioner. I don’t think it has to be a doctor (Woman 02 Caucasian age 72). |
| Increase number of healthcare professionals for OA | [I think] having more specialists that will be able to help people through OA (Woman 03 Caribbean age 48).  I feel like more doctors on this condition because we have to wait sometimes long before we get an appointment, so more employment in the field could really do us well (Woman 07 African age 43). | If we wait for one hour and then they just talk to us in like 10 minutes, we understand that because [doctors] are understaffed. If the government can provide more doctors in the towns like us where we are overpopulated then it would be better… the doctors can take more time talking to the patient, explaining everything, they won’t be in a hurry for the next patient (Woman 05 Filipino age 40).  I do think more attention to funding for services whether it be provided virtually or in-person. The Arthritis Society provides really great services but they’re often underfunded and they face a lot of challenges in terms of their demand and wait list with what resources they have available… I think funding is one of the major issues because that also leads into the number of healthcare providers available that can provide this care and the education information that patient’s require (Woman 14 Chinese age 67). | -- | -- |
| Increase availability of diverse (i.e., gender, ethnicity) healthcare professionals | Women express themselves truly to other women. So what I think should be improved is more [women] nurses should be added to the hospitals. In the hospital where I was, a man served me. So I think more women nurses should be available just to increase the ability for expressing one-self from women to women (Woman 15 African age 45). | I think the hospitals or clinics [should] employ somebody who understands other languages or diversity… they [should] employ some other non-Canadians or from other races (Woman 12 Filipino age 54). | -- | -- |
| Publicly fund therapists | -- | The government should put more free care and different kind of treatment to help. Some acupuncture could maybe help… if covered by OHIP that would be great (Woman 16 Chinese age 62).  I do think physiotherapy that is funded for patients would be really helpful (Woman 14 Chinese age 67). | Maybe sometimes patient needs some physiotherapy [not just family doctor]. There should be accessibility to that because sometimes these things are not covered and maybe this is a financial barrier for many patients to access that kind of service… having access to these allied health professionals it would prevent the complications. And it will improve the quality of life of the patient (Woman 22 Pakistani age 57).  If they want to give relief for people suffering with arthritis we have to get free access to our physiotherapies and some other things which are using for our relief like heating pads (Woman 25 Pakistani age 60). | Access to physiotherapy as an integral part of the management of osteoarthritis within the publicly insured system I think would be a huge benefit to people to ensure that they have the support they need (Woman 11 Caucasian age 72). |
