# Supplementary material for: Multi-level strategies to improve equitable timely person-centred osteoarthritis care for diverse women: qualitative interviews with women and healthcare professionals
Source: Int J Equity Health. 2023 Oct 7;22:207. doi: 10.1186/s12939-023-02026-x (PMC10559457; doi:10.1186/s12939-023-02026-x)
Supplement: Supplementary file 5 — Additional file 5. Themes and quotes – healthcare professionals. [file 12939_2023_2026_MOESM5_ESM.docx]

**Additional File 5. Themes and quotes from interviews with healthcare professionals**

Person-centred OA care
*Clinicians: How do you tailor discussions about OA, or offer treatment or self-care advice that is specific to diverse women?*

*Executives/policy-makers: What constitutes person-centred OA care for diverse women?*

| Theme | Clinicians | Executives/Policy-makers |
| --- | --- | --- |
| Inquire about past and desired work-related and recreational activities (exchange information) | ***Explore physical activity patterns and goals (exchanging information)***  I ask them what they want to get out of their session with me and what they do in their life; how they pass their days, any occupation or recreational pursuits or other pass times… what sort of things they might be interested in getting involved in... Everybody’s different in terms of their background, what they’ve done in the past and what they might consider to do. And from that, I try to pick up some direction for my therapy to be aimed at (01 physiotherapist late career).  Trying to understand a little bit more about them personally from a functional perspective... often their goals might be related to a functional task or a number of tasks or a things that they do on a daily basis, so it’s tearing that down a little bit more (10 occupational therapist late career).  When I’m first diagnosing OA, the first one for everyone is more conservative management which is gonna be pain medication, weight loss, lifestyle modifications. I ask people what that would look like in their lifestyle, what does exercise look like for you or what sort of mobility would you be interested in? What sort of pain medications have you used in the past? What worked well? What’s your diet like? What’s your access or ability to change your diet life in terms of feasibility, financially or cooking for a family (23 nurse practitioner early career).  ***Friendly conversation (fostering a healing relationship)***  The first meeting is just talking with them throughout… I like to chat with my patients. So asking what they like to do, what their family is comprised of, what they do for work, vacations they’ve been on. Just getting an idea of what their general lifestyle is (03 chiropractor early career). | *--* |
| Consider intersectionality (e.g., gender, ethno-cultural group, socioeconomic status) when discussing OA risk factors, management options, or developing policies | ***Ethno-cultural considerations***  Depending on a person’s background sometimes they may have conditions that are more associated with one ethnic background. So, for example, sickle cell disease or anything that will cause changes in the bone (02 physiotherapist mid career).  I deal with a large East Indian population [and] I find that they want to take care of themselves but they know they can’t change their duties at home or what they’re doing at work. They’re still very much like “I need to keep working fully”. I can’t take them on lighter duties or breaks to let the inflammation calm down. I got to work with ways of ensuring that they can do their duties but in a better way. Or do other things to help manage the symptoms… I also find that working with diverse women; they can seem to be more open to using non-medicinal alternatives… I also greatly encourage omega-3 use on a regular basis [and] to limit dairy and sugar and wheat that can be prone inflammatory. And to increase turmeric or curcumin use either through their dishes which is very handy with an East Indian population because it’s part of their traditional cultural dishes, or through a supplement base (03 chiropractor early career).  Tailoring the arthritis discussion is being open to any of their less conventional medical practices. So asking about do you ascribe to any natural healing practices or cultural practices that you feel make a difference towards your OA or that you want to discuss today? (06 occupational therapist mid career).  Some will be very committed that some alternative health method is very supportive to them and I’ll just ask them [to] tell me a little bit more about that. Help me understand how it’s impacting [you] (09 physiotherapist late career).  If we need to have a translator we have access to translating services. We may often bring a family member in as well (18 occupational therapist late career).  With a different diverse population sometimes when you look at what people do at home or how many people they’re preparing for and what their activities [are] or their ability to work, exercise into the lifestyle isn’t always as feasible or something that they would prioritize depending on the group (23 nurse practitioner early career).  Talking about immigrants, a lot of times I tailor [the discussion about pain medication] because the product that seems to be a household item may be really foreign to them… Tylenol might not really mean anything; may be it comes in another name altogether in their country. Or the concerns or worries the cultural background, that maybe this is not good or taking medication is toxic. I think really exploring their beliefs of pain management overall would be something that I’m cognisant about (27 pharmacist late career).  ***Gender considerations***  In terms of female specifically, trying to plan around care-giving needs… [and] a lot of the people have gone to physiotherapy and a lot of their exercises that they’re given are non-weight-bearing. For women specifically because of bone density going down we always educate [about] the importance of functional weight-bearing exercise to protect their joints and their bones (02 physiotherapist mid career).  Looking at some of the risk factors that contribute [to] women who are developing arthritis or have developed arthritis… looking at post-menopausal women in terms of what is the impact of hormonal changes on all of their tissues but specifically joints and the soft tissues that support their joints (09 physiotherapist late career).  ***Financial considerations***  My discussion on OA care are often is around pain medication and the first line of medication a lot of times can be over the counter… so when I think about diverse group, I think about access whether they have medication coverage… first line is a topical NSAID which is not covered our provincial plan right now. So I see that it is really becoming a challenge, so I tailor that thinking… we do not probe on income, but I do ask if they have private insurance coverage for medication… because otherwise prescriptions go out and becomes very costly and really not accessible to them. And on the other hand, if someone has access to coverage… letting them know the fact of this being an option firstl ine for safety and efficacy and this is the cost. Would you consider? Would that be an option for you? (27 pharmacist late career) | ***Ethno-cultural considerations***  I’m not aware of any specific policies around osteoarthritis care for immigrant women. I don’t even know if [there are] polices that specific. It is more about an overarching policy around equity, diversity, inclusion and inter-sexuality which then trickles down into different policies and it is the change in attitude and mindset of people who are writing the policies and who are using those guidelines or tools and it should be built in competencies and performance management... That work is happening but I don’t know how much of it has trickled down… [into] specific policies… There’s certainly an increasing awareness that there is a gap and I think there’s an opportunity to dig deeper… and movement for more inclusive and culturally specific [policy] in a lot of areas (14 government policymaker mid career).  In team-based primary care they do everything… as much as possible from the lens of equity [and] diversity but it’s also evidence-based… there is a real push for everybody in the healthcare system and especially in team-based care to look at the people that they’re serving and taking a diversity EDI lens to it… while every Ontarian needs access to team-based care, there are certain pockets of the province that were needed more… when we look at the lens of who we actually are serving, is it really reflective of those who need it the most? (19 healthcare executive late career).  With recruiting our advisory committee, we bring together a group of experts and we really work to make sure that we have a diverse group in the development of the standards including people [from diverse backgrounds] with lived experience… we try to weave equity [considerations] within the quality standard… the quality standards and our guiding principles underlying the quality standard talk about respect for the rights and dignity of people and [respecting] self-identified, cultural, ethnic and religious background… when we’re developing the standard we go through a number of questions: What can the quality standard do to mitigate variations in access, experience and outcomes? And these are things that we talk to our advisory committee about in developing the standard. What best practices could be adopted to address variation in the quality of care or outcomes when stratified by variables such as race, income and geographic location? It’s just a way for us to be thinking about equity throughout development… also as we’re thinking about dissemination and implementation, we ask the committee “how can health equity be addressed in the dissemination and implementation of the quality standard” … So things like social determinants of health. As we’re developing the standard we’re asking our committee to guide us in that area. And we consider that in our strategy for dissemination and implementation… based on the clinical guidelines and what we hear from people with lived experience, [our committee] prioritize key areas for improvement in Ontario (21 policymaker quality-improvement late career).  ***Consider social determinants of health when targeting primary care efforts***  Those who need team-based care the most are usually the ones that are more vulnerable in the system… If you’re in a community like <city name> where there are a lot of immigrants coming in or a lot of individuals that are just struggling with social determinants of health, healthcare is gonna be very low on the priority list. They’re not gonna go seek out care because they can’t afford time off work, they can’t do what needs to be done to prevent the hospitalization and they may not even go to the hospital even if they are not well. There are a lot of questions being asked by [care] teams around are we even in the right communities to ensure that we’re serving the people as close to home as possible; in the communities that they live with the languages that they speak. And the last couple of years, we’ve seen a deliberate pivot away from offering up access to team-based care to the more privileged… there are so many other individuals in the community that actually need this type of care… everybody’s going through this social accountable piece and the moral obligation around this very valuable resource needs to go to those who really would benefit the most (19 healthcare executive late career).  ***Financial considerations***  We also have a “recommendations for adoption document” that’s also posted with the quality standard and we have identified a number of equity issues within there around financial barriers, so lack of extended health insurance coverage. A lot of people that fall into this group also maybe don’t have jobs that would pay for services like physiotherapy services, occupational therapy, weight management programs, things that aren’t covered by Ontario’s Provincial Healthcare Plan. And also specific populations like newcomers and refugees wouldn’t have those and then would also be impacted by lack of knowledge and access to programs that might be available to them (21 policymaker quality-improvement late career).  ***Equity and diversity considerations***  Of the policies that I am aware of, they are very seldom specific to a gender in terms of osteoarthritis… we encourage diversity in our GLA:D providers. So we train therapists to be able to deliver GLA:D programs and we want to ensure that we had a variety of ages and ethnic representation within our providers because there is often a comfort level with common connection. And when the GLA:D program is offered, providers are encouraged to offer best times for clients, so more evening or weekend for women who may not be in a position to take time off work… but the GLA:D program itself, although you may make modifications in how you teach it, the exercises are standardized. The education portion of it could be culturally improved or culturally adapted… if you have concurrent other medical conditions that may reduce your improvement you are excluded from the GLA:D database program. That can be a divider between women with other disabilities, so women [with] different disabilities; fibromyalgia being one, would be an excluder from accessing the GLA:D program. I’m trying to use GLA:D as an example of where a policy comes down into operations and where the provider or the community that’s providing it may enhance and embrace population needs or where they may exclude needs. We always [apply] a lens for equity on who will benefit and who’s left out. And that’s the lens that we have tried to use with a lot of the OA initiatives…how can you enhance your programs to include more and how can you target your program (15 policymaker quality-improvement late career).  in [our] strategic plan equity, diversity and inclusion is the lens that we work through… we consulted with the arthritis community… to ask have they thought what [fiunding] should be allocated within that community and they prioritized mid career and early-career scholarships… we partnered with the Arthritis Society so that puts money into the arthritis domain but then in the competition [for funding]… we don’t have a targeted area towards research in OA for marginalized diverse women… we’re not funding that space directly… we have a group that are called PERA, the Patient Engagement in Research and Ambassadors… osteoarthritis patients are represented in that group. And then in terms of policy there are competitions in the institute where you have to have patient engagement, that’s one of criteria in the research. So for example, in networks, patient voices have to be included… [we] also co-invests, there’s two large [funding] programs… If you’re doing work in communities that are marginalized… that’s a criteria that would get you points… the [second] competition… they’re about health system intervention and changing the health system and that has an equity element that you have to say hey, addressing improvement in health and making health more equitable… there’s no direct funding opportunity where if you’re working in diverse women with OA… other than funding opportunities within the <institute name> mandate where we’re considering equity in the evaluation criteria (22 executive healthcare late career). |
| Treat all patients the same | I don’t treat diverse women differently than others. I just discuss with them the treatment for osteoarthritis based on the research studies and based on the guidelines for osteoarthritis and I do it the same for any background; the ethnicity and the diversity is not what’s gonna change my delivery of education for these women (04 physiotherapist late career).  We always try to stick by a straight plan for everybody because if you start asking questions that they may perceive as irrelevant, “why would you ask me and not somebody else; why would you not ask a man the same question”. It has to be pretty standardized (05 physiotherapist late career).  We do the same recommendations for conservative management typically across the board but patient-specific. When it comes specifically to tailoring it for ethnic groups… we focus majorly as a physio and encouraging fitness and strengthening (07 physiotherapist late career).  It really makes no difference to me what their ethno-background is because I’m able to communicate with them in English or French, the mechanism of osteoarthritis is basically the same in all people (11 chiropractor late career).  I don’t know if I tailor my care or my recommendations based on the women that are coming in (13 chiropractor early career).  I treat everybody the same. The main thing is figuring out if there is somebody coming from a different background. It’s figuring out what their daily routines are and trying to tailor based on that what they’re doing. So essentially everybody’s gonna be treated the same as far as treatment-wise except the slight changes are gonna be dependent on what they’re doing every day. There might be some things such as prayer and somebody with knee pain that you got to find different modifications for those individuals that might be a bit different than somebody else with OA that is not doing that as frequently (20 chiropractor mid career). | In short there is not tailoring. We follow a provincial care path. We use standardized toolkits, standardized orders. We all use the same teaching material and teaching classes… we approach taking care of total joint replacement patients, OA patients in a similar way. There has not been consideration for sort of sub-groups of the population per se… I think we treat every patient the same which I realize isn’t enough. I would say we don’t have any specific programs and it’s not something we talk about, I’m ashamed to say… as a society we’re starting to learn that treating everybody the same is not enough right? We have to start to think more about individuals as individuals and make arrangements in that way (30 executive healthcare late career). |
| Educate about reason for referral and/or role of specialist care (e.g., PT, OT) in managing OA | ***Educate about role of specialist care in OA***  I work in a private clinic so if you were coming to the clinic there is some assumption that - well some of them don’t know much about physio… that has to be sometimes explained to people if they have a different perception of what the physiotherapist would do for them (01 physiotherapist late career).  I introduce myself and I love to know why they’ve been referred because often times, people are like, really? So we discuss this is why you’ve been referred and this is what I do and this is who we are (08 occupational therapist late career).  They sometimes they don’t know exactly why they’re here. Or they may not know about OT or what OT can provide… introducing OT and arthritis OT specifically; the types of things that we help support people with so that they at least have an understanding about our discipline and our role… We talk about what they’ve identified [as a problem] and what kinds of things occupational therapy can do and we ask for their agreement to address that and highlight the role that each of us will play in addressing that particular concern or issue (10 occupational therapist late career).  ***Inquire about prescriptions***  Typically with osteoarthritis, a lot of people usually we’ll see them presenting a prescription. So usually when we’re assessing a prescription we’re always looking for indication. Why are you starting this medication? Or why are you re-filling it? (26 pharmacist mid career) | *--* |
| Bring one’s own gendered and ethnocultural perspectives to providing care | Where I work we’re all women so I think that we already bring the lens of being a woman and supporting other women. I feel like having open conversations about that it’s been really helpful in our department. I personally have a few friends who are not European white, and we’ve certainly had conversations about health and access to health or being supported in healthcare (10 occupational therapist late career). | *--* |
| Use visuals to discuss OA (exchange information) | I use more visuals. Sometimes I guess Google pictures and then I'll show people their x-rays and then I also have skeleton models in my office, so I tend to use those a lot to describe what’s going on (17 family physician late career). | *--* |
| Set expectations for disease progression and symptom improvement (manage uncertainty) | I try to ask people where they’re at, what they’ve done and then set expectations so they can fit the condition. If this isn’t something that’s going to go away or we can’t necessarily fix and then it will affect mobility especially it’s often lower joints, like the hips or knees. So trying to motivate or encourage the lifestyle stuff knowing that ultimately if all that fails, [there is] surgery and other invasive options that aren’t necessarily a cure (23 nurse practitioner early career). | *--* |
| Educate about OA and the need for early intervention (exchange information) | People need to understand, women as well as men, that there are certain symptoms of osteoarthritis that need attention and possibly with early intervention. You can prevent serious damage just with the exercises, the physio. A lot of times people think if their knee hurts they shouldn’t walk but with osteoarthritis that’s the best medicine you can have. As a pharmacist frontline if I saw someone with symptoms, if I saw it in their hands, if they complained about sore knees or a sore back I would ask them to discuss it with their family doctor (25 pharmacist mid career).  With OA, mostly we’re looking at non-pharm especially in the early stages. We’re always looking at being active just because the sedentary nature [of patients] and with morning stiffness and everything that comes with it… with reduced range of motion, stiffness and pain with initial movement it can really be a determinant or detrimental to the progress of OA. And especially in people with jobs, like with the repetitive nature or heavy lifting (26 pharmacist mid career). | I think one of the significant challenges that we have [about OA] is around awareness and ensuring that people understand their symptoms, their early warning signs and understanding what to do. So from a broad scale perspective there would be education resources that are available if people know where to get them that could support that early phase of OA symptoms when it starts. So there certainly [is] a host of sort of digital tools or resources that I think people can access that would be available to anybody that has access to that technology... some of it is just general awareness about what you’re looking for and there are several different organizations that do provide quality evidence-based information but people obviously need to know where to look and how to look for it… A lot of the other [resources] rely on your primary healthcare provider… There are some healthcare providers that are pretty good at directing people to such available resources outside the clinic and then some just don’t have those as readily assessable for people or to direct to. So I think it’s in variability and how people would be directed to these resources but the reality is, a lot of it is what you get directed through Google, through your browser and making sure that the right content appears at the top of the list (31 executive charity late career). |
| Tailor treatment based on patient goals (exchange information) | I don’t know if I necessarily treat diverse patients differently than any other patients. I feel that whenever I tailor treatment to a patient it’s based off of patient goals… one of the things that we always ask is what can’t you do now that you could do before because of the pain you’re experiencing or what are the specific goals that you would like to achieve? What are your functional requirements? For some women that may be I need to be able to stand and cook for whatever hours of the day or I need to clean my house or I need to take care of my children. For other women it could be something like, I need to climb up the stairs. I need to be able to drive long distances. So I feel that I tailor my treatment based on the goals and needs of the patient (12 physiotherapist late career).  I try to get to know the patient better about what their barriers are to [to getting] care, to exercise, to getting treatment, and try to come up with different accommodations to get them to do their exercises (16 chiropractor early career). |  |
| Include family members in care plans | Often we will get their children or their caretakers to come with them to help with translation and usually try to educate the family members as well. I also like to give hand-outs with illustrations of exercise instructions [for example]. I would like to have [hand-outs] that have different languages on them but most of the time their caretaker or child who takes them to the appointments can read English and help them with the instructions. So that’s one of the ways that I try to help with their treatment plan is to make sure that everybody who’s there is on board and can also assist them (16 chiropractor early career). |  |
| Refer to other professionals (enable self-management) | I don’t tailor my advice specifically to [the needs of diverse women]… I talk to them about things that they can do and who to see. I actually will redirect them to their family physician or if they have a rheumatologist to go to see if they can get back to the rheumatologist to discuss treatment options... I don’t change their medications, I just make suggestions, I talk to them about trying to move as much as they can (28 nurse practitioner mid career). |  |
| Current policies are not customized to meet needs of diverse women | -- | The OA quality standards do encourage communication with patients. They do encourage education with patient as a key treatment parameter. But they don’t specify customized needs for marginalized women. And should that be included or not? The way that the quality standards were developed was looking at the evidence at the time. And in OA when we look at the evidence we often look at the joints that are most affected; hip, knee and hand… within the standards we talk about joint protection and the need for reassessment. It’s not just a diagnosis. The standards address pharmacological and non-pharmacological treatment. But they don’t go down to who are the most vulnerable with the most significant osteoarthritis. We haven’t looked at it from the lens of [who is most vulnerable] and are our recommendations reasonable. But there is the lens of what are the most affected joints and how is non-pharmacological treatment a starting point instead of an add-on (15 policymaker quality-improvement late career).  to my knowledge there aren’t particular programs [for diverse women] which is why I think this research is really important… I just think a lot more is needed and diverse women don’t have equitable access to OA care (22 executive research late career).  I don’t know if there’s anything specifically that exists for diverse women that I’m aware of… I actually think that’s part of the gap... almost in a way like a passive barrier is that we think about diversity, we think about gender… the IDEAs work focuses on communities and Indigenous communities it doesn’t focus as much on gender lately and it doesn’t focus specifically on diverse genders…  The Arthritis Rehabilitation and Education Program, our strategy which we’re just starting to embark upon, is enabling equitable access for individuals. With the lens of mostly rural areas… If I’m being honest, we haven’t specifically dug into gender specifically, it’s been more around Indigenous groups, marginalized groups, not so much a gender gap that might exist; it has not been on our radar as much and it should be… We also are looking at all of our materials to make sure that they are equitable and accessible for individuals who may not have the same level of literacy as others. So we’re really trying to look through that lens of education… are looking at individualizing our treatments more so that it isn’t cookie cutter… we’re trying to give the therapist some autonomy to really treat every person as an individual and not just have a cookie cutter approach (29 executive charity late career). |
| Beginnings of efforts to target marginalized populations | -- | I do think there have been some advances in terms of some investments. I’m thinking from a provincial lens anyway. And in Ontario, we have [an arthritis] education program run through Arthritis Society Canada, the GLA:D program that has been invested in to help people who have OA that may not be joint replacement candidates or if they’re on a long list for that. I think there’s been some work over the last 10, 15 years around the Rapid Assessment Clinics… I don’t [know] if they specifically deliberately been focused on bringing equitable access. I’d have to say maybe not, that they are programs that people can access, just in nature they were overall have access but not for specific groups… I would say there’s been a good intention and fits and spurts of initiatives. For instance, I think there was something called the Get a Grip Program which is to train physicians better in how to manage OA and engage physicians in that. I think it is fits and spurts and the sustainability of that has often not been effective (29 executive charity late career). |

Person-centred OA care approaches

| **Theme** | Clinicians | Executives/policy-makers |
| --- | --- | --- |
| **Foster a healing relationship**  Extend friendly greeting, make eye contact, speak in respectful manner, avoid judgmental attitude | ***Offer sex compatible therapist***  If anybody has any diverse needs in terms of needing a female therapist or a male therapist… we try to make accommodations as needed (02 physiotherapist mid career).  ***Engage in friendly discussion***  Seeing what their daily living is then saying, “oh I understand. I have a nephew, and he loves to play and wrestle and climb but I know that I’ve got to be careful lifting him or running with him because it could be hard on your knees” (03 chiropractor early career).  I’m a person of color and I usually talk about cultural things with [patients]. A lot of my patients are Filipino and we have similar Asian cultural practices. I usually talk about personal stuff… food and all that kind of stuff and I really get to know them or their family member… I like to ask about their day-to-day [activities] and what they do... I just try to get to know them on a human level. I find it’s really helpful for them to open up and to trust me and listen to my advice (16 chiropractor early career).  ***Start with introduction***  I like to start the session with an introduction to myself and the clinic and then a general introduction of what the referral says (06 occupational therapist mid career).  If I’ve never met someone, I obviously introduce myself and let them introduce themselves and ask what brings them in and usually clarify some pieces of their history (23 nurse practitioner early career).  If it’s the first time... introduce myself, my role... and how I will be involved in their care (28 nurse practitioner mid career)  ***Create a comfortable environment***  We try to have nice furniture so that people can be comfortable sitting and ensure that people feel they are okay to spend time telling us what experiences they’ve had and what they want to share. I think we’re very aware people maybe can’t sit and so [telling them] please stand and move around if you need to. Or if this is allowing space for them to share what they feel comfortable sharing… my experience is that you can start to develop a bit of rapport, a bit of ability for people to feel comfortable in that space but that does take time sometimes for people to share (10 occupational therapist late career).  I guess building rapport by facing the person, having good eye contact, making sure that they hear and understand what I’m saying… Giving them time to describe medical history things like that. In a more acute scenario, like a walk-in scenario it’s more of a, hi, my name is Dr. <name>, what brings you in today? And then let the patient talk about their current ailment and then work through questions after giving them a couple of minutes and then just narrowing the focus of the appointment in order to understand the problem (17 family physician late career).  ***Offer a private space***  We always have a private space in terms of a closed door (02 physiotherapist mid career).  We use a private office space and it’s quiet and comfortable (10 occupational therapist late career). | ***No policies encourage building rapport***  I’m not aware of any proactive policies that help build relationships. I think there are providers within certain work settings, a community health centre for example, where they are able to actualize care. But I can’t think of a policy that either is specific to women or to marginalized groups (15 policymaker quality-improvement late career). |
| **Exchange information**  Listen to concerns, prompt for additional details, understand needs, goals, circumstances and preferences, use lay language, ensure privacy | ***Provide or accommodate an interpreter or translation services***  If English is not their first language we will arrange for an interpreter if needed or they can bring a family member, but we always encourage an interpreter (02 physiotherapist mid career).  If they can bring a family member along that can translate, that’s much better. If not, I’ll try to direct them to websites or have something printed out and then Google translate that I can hand to them at the next appointment (03 chiropractor early career).  If we have a language barrier we usually use iPad translation or family members if they are available or if we know the language of the patient (04 physiotherapist late career).  If a woman that I’m working with has language barriers we’re hoping that that was identified prior to the appointment so that we can arrange an interpreter or a family support if they’re more comfortable with that coming into the appointment (06 occupational therapist mid career).  When it seems to meet the needs of everyone there, we’ll have the translation done by the family member. We do have access to a translator service that we could do on the iPad should we need to but in our capacity and the fact that we book patients far out, people seem to often prefer to bring a family member, maybe they just gotten use to that in our medical system… we just leave that up to the client that they know a translator service is available but I have to say that I’ve never used it (08 occupational therapist late career).  Family members come to appointments with someone, that could be a friend or a family member that helps to interpret if the client feels comfortable with that… we can arrange for an interpreter to come in and be in-person in the space and work with them to understand the client’s issues and concerns. We also have access to some virtual interpreter services. So we do use those although I think they’re not quite as effective but there’s still of a role for them to play [in] interpreting and making sure that we understand correctly (10 occupational therapist late career).  If they don’t speak English obviously, I’d try to get a translator to help with that and there’s a phone translator available in Newfoundland. And if that’s not the case, if someone in their family is able to translate, that’s helpful but not ideal (17 family physician late career).  We will look into whether language is a barrier. We will look into any written material that we have that has been translated. We have quite a bit that has been translated into Mandarin for example, and Punjabi (18 occupational therapist late career).  If there’s language barriers we invite a family member to come and be there so they can interpret anything that isn’t clear. I’ve experienced that many times where someone has come because the patient just couldn’t understand enough (25 pharmacist mid career).  Dealing with people that might not have English as their first language too, a lot of times it’s nice if they’re able to have one of their kids with them who can translate or a family member or friend if English is difficult for them to understand. There are some good resources for patient counselling that are not in English… patient or disease-specific, brochures essentially like booklets or leaflets or medication education resources… if I can find something that’s in their language that might be a little bit more effective than myself explaining it to them (26 pharmacist mid career).  It’s good to understand them, be able to translate what you’re saying to them... We do have translators' services that we can use for the patient (28 nurse practitioner mid career).  ***Inquire about patient barriers to treatment or management***  I like to ask about [diverse women’s] access to certain things. So how easy it for them to get to their appointment or how much time in the day do they have to do exercises or do they have access to a facility where they can do exercises…and then we try to organize a treatment plan that works for them and [ensure] they can actually get to the appointments or do the exercises (16 chiropractor early career).  ***Explore daily activity patterns and goals***  [Some]times people are more forthcoming with their goals of the treatment session or where they want to improve in their life, what sort of activities they want to get better at or resume or work towards. Sometimes it’s not evident and needs to be specifically asked (01 physiotherapist late career).  I like to see what their daily activities of daily living are. What are they doing? Are they in a bakery kneading bread all day? Or are they at home watching grand kids? (03 chiropractor early career).  Whenever we’re doing the assessment and we find that there is low function and no physical function or there is an increase in BMI due to the lower function, we don’t discuss straight forward about weight-loss. We discuss about staying fit first before losing the weight because it’s a sensitive point for patients, and some of them have already tried lots of weight-loss and they were not successful… most of my patient populations are Indian but I could see that some of them are very sensitive to the topic of weight-loss and some of them are very open to discuss weight management (04 physiotherapist late career).    [We ask about] social history including current situation at home… if they have stairs to manage… if they have children or if they’re a care provider, what they’re dealing with day-to-day and physically what they have to do throughout the day… what their occupation is, if they’re working (05 physiotherapist late career).  [I ask] really open [questions] like “what is your understanding of why you were sent here or what were you expecting of this appointment or hoping to achieve” because I think if we ask too many guiding questions, if I’m like “you’re here for your OA [in your] hands and the doctor sent you for a splint” then they may get really stuck in, “okay I’m just gonna see you and get a splint”… get their perspective of why they requested to come here or why the doctor might have sent them and what they’re hoping for or what their biggest issues are (06 occupational therapist mid career).  [We] focus on if they are doing exercise what specific exercise… whether it's daily walks or going to the gym or what is their fitness routine (07 physiotherapist late career).  With occupational therapy we’re afforded to be so client-centred. I find a lot of professions aren’t as lucky as us to really focus the session around that… I’m all about them and the appointment. What is important to you? What do you want to do? You can tell me and then we can work together on that… look at what’s important to the client, self-care, productivity and leisure depending on what they’re identifying as their goal… we generally take a history where we delve into their top 3 goals for the session within the scope of their referral (08 occupational therapist late career).  Looking at issues around their general level of nutrition, the type of work that they do and how that can contribute to joint stresses and their work-life balances… some women are working in very heavy care like personal support worker environments where they’re doing very heavy-lifting where they’re more prone to injury where other women who are in very sedentary jobs also have huge problems because of inactivity or just an inability to change their position during the day (09 physiotherapist late career).  We want to understand where they’re coming from and what their knowledge base is… to inform them with resources or strategies to troubleshoot their own personal life. A lot of it’s just understanding someone’s circumstances and being able to help start them on their journey of learning about what they can do for themselves… people may want to understand how they can modify some of the tasks that they do on a daily basis… there’s a number of functional questions that we ask to understand basics about their home situation, if they live with anybody, if they’re working. How active they are and how much time they’re being active. What are their goals for coming into the program… they sometimes they don’t know exactly why they’re here (10 occupational therapist late career).  I start with where is your pain located? What’s aggravating it? What’s easing it? … What activities do you need to get back to doing? Are there any specific goals that you want to achieve? What is it that you want to be able to do that you can’t do now? … I just keep asking the same type of questions in different ways to really say, listen, I’m here to help you achieve your goals. So if your goal is to be pain-free I need to understand what you need to be pain-free for so I can customize the exercises and the activities and the treatment to meet that goal (12 physiotherapist late career).  I do a general questionnaire on all of my intake forms that asks a few lifestyle questions, like how active the patient is per day, how many minutes of exercise are they getting… what their day-to-day schedule looks like…I also ask about water intake, what their diet may look like. I do that for everyone who comes in to give me a better picture of their health before I even see them (13 chiropractor early career).  Starting with age, when I’m tailoring my approach to assessment and diagnosis and management, it’s more to do with what was their function; I may not [ask] specifically for young people but I mean they may have a little bit more function or more goals for more intense activities. So related to age, I’d probably focus on goals of treatment with regards to the intensity of activity that they’re hoping to do. Especially if they have like young kids (17 family physician late career).  Often there will be a family member with them, and I will get to know a little bit about their family. I’ll ask them questions related to their role within the family unit because as an OT, I’m looking at ADL’s and we do try to determine what the clients’ goals are so in doing that in the interview we are getting to know more about their role within the culture and within their family unit… In terms of the ADL’s I’ll ask what their goals are for coming in to see us… usually from that then you can determine, oh they would like to be able to spend more time with their grandchildren or be more involved in the care of their grandchildren... I’ll ask about what brings them joy? What would they like to able to do better or with less discomfort? … talking them about their activities, doing an activity analysis, getting a sense of, if I was making them a splint, what type of splint would be most benefit to them depending on the activities that they’re doing and what would be culturally acceptable for them to wear or not to wear or different ways of doing activities (18 occupational therapist late career).  During just a first visit, people come here for a complaint most of the time. But aside from talking about that information, I always start talking about where they’re coming from, if they have a family, their kids, their job. If they’re recent immigrants or not; how do they like it? Just getting to know how they are, sometimes that takes a little bit of time; depending on the timelines I do have, I’ll often just tell a patient that let’s refocus on what you’re here for but we’ll talk more about these things as I’m working on a patient after that. Like their activities, what they like to do? How they spend their time, etc.? That’s usually a big part of my first visit (20 chiropractor mid career).  I like to get to know people in terms of asking what’s your lifestyle like at home? Do you live by yourself? Do you live with others? What are some of your duties at home? Are you working? Are you not? Are you a caretaker? Are you cooking a lot?... When we think about especially older women who is the common demographic, especially if they’re caretakers of family members, young kids in particular or more elderly family members at home, is that they’re like I’m constantly moving, constantly up off of my knees and on the ground. I’m cleaning, I’m moving things depending on what the ergonomics [the movements] might actually be aggregating it and putting a higher burden on the joints often affected… I’ll talk to them about the types of activities they’re doing, if it’s a lot of cleaning or if it’s playing with children, are you sitting on the floor? Are there cushions or assisted aids for you to stand up… go through a lot of ergonomics and exercises to say how can we incorporate these into the activities you are doing at home (23 nurse practitioner early career).  You need to understand what their daily routine is like… what is it that you do; how do you spend your day? They’ll tell you if it’s assembly work, if they’re working in an office, if they’re sitting a lot of the time. Or if they’re at home looking after 8 children (25 pharmacist mid career).  I ask about physical activities and maybe that’s when if the person has osteoarthritis or has limitations with activity, then they will mention, maybe I’m able to walk for this distance or I’m not able to walk at all because I have this pain to my knee or pain to my hips or I have osteoarthritis (28 nurse practitioner mid career).  ***Discuss x-rays with patient***  Often times I show them a video that explains the work we’re doing…the video explains how when you start to restore normal function to the body, the body starts to repair itself. When I examine them, then I have the ammunition there and the information to request x-ways which I usually do and we check out what’s going on with the x-rays.  And once I get all that information, the clinic test and the x-ray, then we have a sit down and we look at the x-rays and I tell them what needs to be done to correct it and we start the process if they’re interested (11 chiropractor late career).  ***Inquire about medical and family history***  We really treat each referral that comes in individually… we do a full subjective history including their medical background, any family history that they might have and then do a physical assessment… [for] family medical history, they usually come to us for OA but sometimes they have no diagnosis of OA specifically or if I see a secondary diagnosis then we look at their family history to see if there’s any inflammatory arthritis (02 physiotherapist mid career).  I get the really detailed subjective history and I focus on the past medical history… I do my subjective assessment and I do my objective assessment. Then I explain every single thing on the x-rays for them (04 physiotherapist late career).  Mainly gathering information such as their first language, date of birth, medical history (05 physiotherapist late career).  We don’t do as much as a familial medical history, it’s more personal history. People usually who have a familial history of something do offer it up but we don’t ask that specifically in our intake… when we’re asking about the joint history we go over any conservative management that’s being done to-date… they do a physical exam and go over the x-rays with people and then [we] make recommendations (07 physiotherapist late career).  Looking at things like family history, traumas that they’ve gone through physically in their life that have contributed to their development of a traumatic level of arthritis… often with arthritis we see it within families so there’s very commonly that genetic component that I explore with them… I take a long time to talk to patients and get that personal history, that health history, that genetic history and try to help them and me put together what’s causing what (09 physiotherapist late career).  We have a history form that was ask them to fill out [ahead of time] that outlines some of their medical background, medications, if they’ve worked with other healthcare professionals, if there’s an x-ray report… [we also ask] a couple questions in regards to safety, like a screening for falls (10 occupational therapist late career).  [Patients] have to fill out a [4-page] comprehensive examination history… when I take the history I question it further if I need to have more information about it… after they tell me what’s happened, how they got to be that way and they’ve answered my questions, my main interest then is to see how the body is actually working so I can determine what needs to be corrected (11 chiropractor late career).  We’re doing their best possible medication history and a lot of time if you have a new immigrant or a refugee… we have Alberta Netcare which is our provincial-wide electronic health record. And a lot of people they might have just gotten Alberta Health Number and there’s really no history on there… a lot of people bring in these medications or lists that I’ve never heard of right? So it could be the same drug here but it just goes by a different name somewhere else (26 pharmacist mid career).  I take the time to ask about their past medical history and to understand that... their allergies, their medications, their past medical history... as I get to know that, depending on what [medications] they are on, I discuss the specifics with them (28 nurse practitioner mid career).  ***Inquire about social support network***  We go into their social history. Surgery can be very successful at managing their osteoarthritis. However, we do know that social support after surgery is very important as part of their recovery. So, we always ask is there somebody at home or do they have what we call a co-pilot. It doesn’t even need to be someone in the home but even a neighbour or a friend or family that lives close by… we find out where they’re living, is it an apartment? Is it a home with stairs? Who is around that could potentially help them to support their recovery after surgery? (02 physiotherapist mid career).  Do they have a supportive person that they can kind of share their worries with. Some just don’t have anybody to talk to (09 physiotherapist late career).  Often they are involved in care-giving for their grandchildren, so will ask about their support systems… is it difficult for them to come in to see us to get an idea of transportation and what their role is there. Are they able to drive, are they not, are they dependent on family members or friends? … we talk about having to delegate or the option of potentially delegating some tasks is that an option for them in terms of their family unit and their culture (18 occupational therapist late career).  ***Explore impact of OA on daily life***  Our first question [is] what brings you in and then what is the thing that limits you the most? (02 physiotherapist mid career)  I ask them about how does their osteoarthritis impact their life because lots of people live with pain, lots of people have different limitations but some people don’t perceive it as a limitation on their life. And this question is the most important for me to understand the effect of arthritis on their life and on how they are dealing with osteoarthritis on their day-to-day living. Some of them even cry whenever I ask them this question because the impact obviously is much stronger than anyone else who does not have the same level of pain or decreased function (04 physiotherapist late career).  We ask for functional limitations of the patient and their day-to-day life whether it’s within the house, outside of the house, work duties… their ability to do stairs and whether they can reciprocate stairs or not reciprocate stairs, difficulty putting their shoes and socks on, their walking tolerance if their walking is limited and if it is, to what distance or time they can tolerate walking, whether the pain wakes them up at night (07 physiotherapist late career).  We do talk about how their arthritis is impacting them, in what ways and what they really want to achieve during our session together… if they say to me, I’m having difficulty with this certain task… I’m tailoring my approach to try and find a tool that will make it easier, a technique to make it easier… we will sit down and ask them specifically what are you having difficulty with, what’s important to you? What do you need to be able to do? What do you want to be able to do? And what do you have to do? And breaking that down into are they having difficulty completing it (08 occupational therapist late career).  Help people look at the big picture of lifestyle and postures and positions and how all of that can contribute but those are all areas where you can intervene and be successful and try to help people understand that some of their ideas, their attitudes can contribute equally to their situation and helping them reframe their thinking… I try to look at everything… Talk to them about their family, their home environment, their work environment, their stressors; what are the things that are putting physical, emotional stress on their lives. What are the things that are hopeful or good in their life that’s maybe helpful towards [a] better situation for their joints… very holistic and say, tell me everything about what’s going well in your life, what’s not going well (09 physiotherapist late career).  I like to ask about their day-to-day activities and how it's being impacted and how they feel about it… I ask them if they’re able to do the things they like to do and if [their condition] gets in the way of playing with their grandchildren or taking care of them… I had one patient with pretty bad knee OA and originally her daughter would say, if it hurts don’t walk, don’t move, don’t do anything, just rest… I could tell the patient was feeling very frustrated but her daughter would just say, well don’t use it, don’t go for walks when she wanted to take her grandchild to the park… I discussed the importance of consistent movement and it changed even her daughter's perspective about what was going on with her (16 chiropractor early career).  We do a pain history. Where’s the most prominent symptom. Where’s the pain? Does is go anywhere else? When did it start? Is it constant? Does it come and go? Does it wake you up at night? What type of pain is it? Is it burning, sharp shooting, aching? Is it causing you not to be able to do things? What’s the severity of it? Have you taken anything to help it? Has that worked? Have you done any different exercises or gone to any therapies that have helped or not helped? Swelling, redness, heat to it? Was it some sort of traumatic thing that started or does it come on over longer period of time? What kind of activities do you do? Are there repetitive motions? Are you running or climbing things? Or is this work related? Is there associated numbness and tingling?... [are] other joints affected? And whether or not there’s a family history of not really osteoarthritis but maybe other inflammatory arthritis that may cause these similar symptoms… [and] there’s non-verbal cues, you’re looking for how someone sits or stands or moves when they’re coming into the office. Because if those things are affected then you’re looking at something that’s really affecting probably everything. Whether or not it’s affecting their work if they work outside the home or is it affecting their life inside the home. Whether or not they’re able to do tasks either in or out of the house. If they have children or other people to take care of; does that affect that (17 family physician late career).  When does that affect you most? How do you feel about taking pain medications if people are resistant or people are taking a lot? And say what does work well because often times a lot of people are given a chronic diagnosis like this, it’s like you’re just gonna have it forever... It’s just arthritis, deal with the pain when it comes. Asking people how do you feel about it? What they know about it? With pain I’m like, has this been going on for a while? Have you had anything like this before? What works well in the past? Just asking a little bit about what brought you in today… what worries you about what’s going on? How’s your health been? (23 nurse practitioner early career).  Ask people how their sleep is because pain can affect sleep, so are you sleeping well? How do you describe your mood? Those kinds of questions can give you a little bit of insight into what they might be experiencing (25 pharmacist mid career).  I like to understand currently how OA affect this person in front of me. It can be certain tasks or daily activities or occupational or vocational stuff. I find that it is most helpful to explain [OA] in that context to them… what currently are their limitations of what they’re doing or things or tasks they long to do but not able to… I find that linking possible outcomes or treatment goals to their own setting in their cultural appropriate way or whatever is meaningful to them helps to explain [outcomes] (27 pharmacist late career)  ***Explore impact of OA on daily life through assessment tools***  We use a start Back scale which gives us some objective data and the Oswestry Disability Index. It gives them an opportunity to functionally rate their performance and then we look at the start Back which gives a lot of questions, it digs deep into their sense of control and/or chronicity of their situation… try to use some of those tools to guide questions and also leave them with some open questions and is there other things you want to share or anything else you’d like to tell me (09 physiotherapist late career).  I’ll want to get like a good evaluation of their disease state. I like to use the tool Scholar… it’s just an acronym for Symptoms, Characteristics, History, Onset, like when did it start as a sudden change or has this been a slow gradual decline. Location, so joints affected for example, is it your knee, is it your hip, is it your hand, is it in your back? And then the A and R are, Aggravating factors, so what makes it worse? nd then Remitting factors or Relieving factors, so like what makes it better? (26 pharmacist mid career).  ***Explore impact of OA through discussion and active listening***  I usually go back to [the patient] and get their interpretation or ideas of what’s wrong or what the problem is. I don’t really think that often the formal referrals capture honestly what the client’s living or experiencing or what their quality of life is… drawing on some of the skill sets learned, like through motivational interviewing so that you’re really getting the client’s perspective on what their lived experiences and what their barriers are. Trying to be quite open to anything they bring up and then delving a bit deeper into that… I find as an OT we have a little bit of that flexibility where we don’t have to get so into the medical model even though we work in a medical system that if they’re bringing up “this is my biggest barrier” and then it’s like tell me a bit more about that… Or often they’ll identify challenges with navigating the healthcare system and the more medical model… Clients often identify concerns maybe about different medication or different more traditional medical treatment. Sometimes more often if they are of an ethnic background… so just listening, being an open active listener and really hearing what they say and not being fearful. I think clinicians get nervous about offending clients if they don’t identify as the same ethnicity or know a lot about that culture or that person’s personal history. I think most clients are pretty open if you just ask the questions (06 occupational therapist mid career).  ***Use lay language or visuals when communicating***  I actually try to keep it fairly consistent [among cultures]. The only thing that probably changes is just understanding if there’s cultural barriers or language barriers that the information may not be understood quite as well… I just would give an extra opportunity for that person to ask more questions and try to ensure that they understand the message that’s being delivered (05 physiotherapist late career)  I would just probably draw more on our visuals and props like bringing out physical examples of devices or of a joint, bring in a model out or bringing diagrams and pictures… just drawing on those that you’re not only relying on the verbal but that they’re seeing it in front of them (06 occupational therapist mid career).  When we are explaining something we’re not trying to use really big terminology that is maybe very confusing for people… we don’t always know what education background someone has; we are very aware that for some people, English is their second language or maybe a third language (10 occupational therapist late career).  Using visuals like pictures or diagrams or models that I have in my office can be easier if you’re trying to explain something to someone whose you know first language may not be English (17 family physician late career).  [consider] education levels for sure, keeping it extremely simple and sometimes diagrams are the best visuals to show what’s happening in the joint and talk about strategies for that (25 pharmacist mid career).  ***Explore patient’s understanding of osteoarthritis***  Any history of x-rays, have those been explained to them. Do they know what’s going on with their joint? Do they know what I mean when we talk about osteoarthritis? Do they understand what that diagnosis even is? A lot of people don’t know what osteoarthritis is, so do they understand what this is? (08 occupational therapist late career)  The most important thing is explaining to patients what OA is and that is non-inflammatory and that there are things that can be done to modify; that sometimes, though there may be no modifications or if somebody is not interested in any modifications on how to move or do certain things, it’s explained to them that it’s not necessarily gonna make matters worse in their joint or their areas of problem. But that it might just increase pain levels for a temporary period of time. I think for people to understand what OA is and how it can progress and what could make it worse… then they can make that choice whether they are willing to make a modification of if they can, depending on what they’re doing (20 chiropractor mid career).  A lot of times people are like, oh it’s just arthritis, or you get told once you have arthritis and someone does like a 3-minute, lifestyle, take care of yourself, mobility that’ll make a difference. And then from there on out, people are like, you know what it is and you know what you should be doing and often times people don’t. I try and ask them what they understand about arthritis and if they know other people who’ve had it (23 nurse practitioner early career).  ***Inquire about use of complementary alternative medicines***  A lot of times too, especially today and this is with almost with all patients regardless, is just the use of like complementary and alternative medicines and natural health products. It’s really important that us pharmacists establish what they’re taking and what for because not only for drug interactions but just for safety, for efficacy, is that something that actually is proven to help or is that actually making your condition worse (26 pharmacist mid career).  In erms of cultural background pain management as a pharmacist, one question I often ask, as opposed to making assumption, [is] how is medication being managed at home. Whether the person is self medicating or self-directing on their care versus there is a family or spouse involved and often inviting that person or probe if there is such a person that would be a really pertinent to the discussion of management… complementary alternative medicines, it is a piece that can be having a rich cultural influence. I ask in a non-judgemental manner about use of complementary medication and I would use verbiages as non-prescription, natural remedies or whatnot. Often I do get a variety of information or treatment, do you see an herbalist or acupuncturist? I believe that gives me a more complete, wholesome [view] of their approach to care and when this is shared with me what other kind of ways that management has been on-going, only then I would be able to see the full picture and then assessing on their perceived benefits of these treatments (27 pharmacist late career). | ***Provide or accommodate an interpreter or translation services***  [the Quality Standards mention] language being a basic tool for communication and an essential part of safe care and needs for those. So making sure if the person is not able to communicate with the care provider that there is somebody available that can help to interpret for them (21 policymaker quality-improvement late career).  We have translated sections on [our] website. So it’s Punjabi, traditional Chinese, simplified Chinese and Farsi. From a language perspective that was something that was identified early that OA affects obviously lots of different populations, so trying to make our information as accessible as possible. We have educational materials translated into different languages to make it accessible. We’re running a series of education sessions all done in Cantonese and we’re gonna teach it with a translator, to make it more accessible for different populations (24 leader healthcare late carer).  Some of our materials are [translated], I think in terms of outward facing materials. I think there’s work to be done there for sure, we do have some French classes… that’s part of our mandate with the Ministry because we’re funded by the Ministry of Ontario… we run group sessions on Zoom now and most are English, some are in French and then any other languages, we just don’t have the team strength in terms of diverse languages to offer that. So there is a gap there for sure (29 executive charity late career).  ***Inquire about satisfaction with previous care***  A lot of tools are implementing patient satisfaction tools, post visits. So it may not be specific to OA but it could be generic enough around like, did you get the care that you needed when you came into the clinic? Tell us a little bit about yourself? That’s where you may be able to get a better handle on, I don’t feel like my provider knew enough about OA or yes, I was I was able to adequately get my questions answered (19 healthcare executive late career).  ***No specific strategy to explore needs of diverse women with OA***  I’m thinking something like a 811 in British Columbia phone line if they’re getting health advice and I don’t know anything like that for communities and things in different languages (22 executive research late career).  I know there’s some work being done and greater attention being put onto how to meet the needs of diverse audience have in particular. I think a lot of this probably still in its infancy in a sense... over the last couple of years we have spent some time looking to understand which of the communities in Canada and our populations that we serve. If we can think about how to specifically target those individuals differently; how could we impact those people but I can’t point to anything that I’m aware of that has would be a strong resource for women in that particular demographic (31 executive charity late career).  ***Explore needs of diverse groups***  We have had our staff in… cultural sensitivity training as well as Rainbow Health, for LGBTQ. We’ve done some specific courses and provided those to staff just to make them more aware and understand better… We have an IDEA’s working group which is really looking both within our own teams of how we recognize and celebrate and support diversity but also even outside of our wall which is with the people we serve… our mission team is the one that basically builds our website and puts resources on… I know there has been a commitment to look at diversity in some of the materials that come out of like for support and education… there’s one [webinar]that was Women in Arthritis, so there are some modules that are geared towards women specifically (29 executive charity late career).  We invest in research, we support patient advocacy and we also develop patient education tools and resources… we are investing in work to understand sex related, gender related, differences in arthritis care or its impact. We are looking at understanding some pieces around different cultures and different backgrounds and how that affects their arthritis experience in the healthcare environment right now… the foundation of our advocacy is really around making sure that people have timely equitable access to care and that is a very large significant part of the advocacy portfolio that can span a lot of different places. And then the last piece around our education, it’s always a balance for us in terms of trying to reach as many people as we can possibly can… We know that there are many people that need access to these tools and resources at the same time though we know that the generic kinds of communication, education will not reach everybody. So trying to pinpoint certain communities, or certain groups of individuals at least it’s a start to understand how we can partner with different community groups, different community leaders and how do we try our best to make sure that those individuals have access to the tools and resources that they need… we’ve adapted [our most population osteoarthritis information resources] into different languages, Arabic, Punjabi and others. So working with different members of the community; whether they’re community groups that are helping us to do the adaptation or helping to do the dissemination and helping to influence how we position that content. We’ve done some outreach in the communities to help us as we work through those projects (31 executive charity late career). |
| **Address emotions and concerns**  Actively inquire about feelings, acknowledge concerns, express empathy, note that such feelings are normal or common, suggest strategies to cope or mitigate emotions | ***Assess indirectly through discussion or body language***  I get a feeling for how their energy is generally… they might mention that they’re depressed or they’re sad that they’re not able to do the things they used to be able to do (01 physiotherapist late career).  Aside from just talking to them and seeing if they’re really concerned about it, reading their body language, I don’t do a follow-up questionnaire or anything based on that (03 chiropractor early career).  Sometimes you don’t have to ask, you can just tell because they become emotional talking about it. As far as asking, often times that comes out just by asking, what is the goal of treatment? What is the goal of you being here? … How much are you willing to make a change? That’s usually the best way I ask. I don’t have anything else specific that I ask typically… And often times that will come out like, “I can’t do this and it’s really affecting the ways of me dealing with my kids or my partner, etc.” (20 chiropractor mid career).  Stress is a big factor. You can usually tell when examining someone if stress is a factor because they show a stress pattern in the musculoskeletal system and that gives me an opportunity to discuss how they can handle their stress or try to find out what the source of their stress is. And sometimes doing that allows them to make decisions about how they’re gonna handle stuff in their life. Women express their feelings a lot more than men usually (11 chiropractor late career).  People see me when they’re in pain. Lots of them, you can actually see their emotions. Sometimes people are angry, sometimes people are tearful, so sometimes you don’t have to ask how [OA] is impacting [them] at an emotional level because you can hear the frustration in their voice sometimes… you can hear the fears… like I can’t go up the stairs now… does that mean that I’m not gonna be able to care for my family? ... so I ask questions but I feel like you can sense the impact this is having on them from an emotional perspective (12 physiotherapist late career).  Two things come to mind. The OA [of the] hands as well as the lower body knees and hips. Hands is limiting what they do and often women are in terms of household chores to their work, like cooking or whatever, open jars… not able to do the things they like… it really starts that vicious cycle that it’s very tricky and that ties back to the pain management, how to try to break that and address you know the whole person (27 pharmacist late career).  Depends on... what they tell me. “Oh, the pain I’m feeling it debilitates me, I’m unable to walk or I’m unable to sleep... it reduces my ability to do my routine stuff at home” and all that. That’s basically the effect to which I get into it. I don’t go delving to details because that’s not the purpose of the visit (28 nurse practitioner mid career).  ***Assess directly through discussion or assessment tools***  We ask what their pain is, how it is affecting them, is it causing any anxiety or depression? How is this affecting the family… that would be on our initial part of it and then it would be, if you’re comfortable, how is it affecting you, how is it causing you anxiety? Usually, the answer is… “I can’t do “x” or “y” or “z”, therefore I’m getting upset about it and I have to do “x, y, z” (05 physiotherapist late career).  I think we do this with everybody. We ask how they’re managing and what kind of support they have and what that impact is like... People don’t always present in a certain way when they’re sad or they’re frustrated or they’re distressed about something, they can have various different expressions and things may not be translated. So it’s a matter of clarifying… that impact and acknowledge the experiences that they’ve had and give them an opportunity to hopefully share that information so we can address some of the concerns or empower them to address them (10 occupational therapist late career).  How do I assess [diverse women’s feelings or emotions about OA and its impact on their lives]? Probably just by asking them. How is it affecting their role within the family unit? How is it affecting their work inside or outside the home? How is it affecting the family? Because of the change of role within the family unit… I don’t have any particular assessment [for emotions]. I will ask about the emotional impact for sure. We have a social work counsellor here on staff, and we have a history form and we do ask people about whether they’re experiencing any stress or anxiety, because we ask about any co-morbidities. So it’ll often come up in that line of questioning (18 occupational therapist late career).  We want them to understand that pain can cause mood changes that it can affect their sleep and it can certainly cause depression if it goes untreated. So, it’s important for them to understand that they need to find ways to manage their pain because if they can manage their pain, they’ll be more active, they’ll possibly be able to exercise a little bit to help with them with management of the OA. But we really have to delve into how much pain and depression are they experiencing because if they are, we need to tackle the pain issues. Some people will say I can live with it and their mood is fine... Some women tend not to complain [about pain] at all. If you use a VAS score…which is a slider that goes from 1 to 10 to summarize pain to the patient… or a facial pain scale and you say “where on this scale would you put your intensity of pain on average over the past week” that is a good insight into what they’re dealing with on a regular daily basis… that’s one of the tools that I’ve used in my practice (25 pharmacist mid career).  I usually use the [Patient Health Questionnaire-9]; it’s kind of my go to for mental health assessment… to assess [major depressive disorder] and to see how they’re doing. Those are patient reported… I’ve never done like a [Hamilton Depression Rating Scale] or something like that, like a clinic remission reported one. I usually do patient reported tools as a baseline if I don’t know them or the monitoring parameter to see if there are any improvements… it’s used as needed… usually just based on clinical judgement if I think that they might be depressed or anxious, and the thing about the PHQ-9 is just a baseline, it’s not really identifying underlying cause. It just identifies that hey, they might be depressed and maybe they need additional treatment… I’m pretty comfortable in the mental health assessment (26 pharmacist mid career).  ***Acknowledge distress and offer reassurance that OA can be managed***  If they come in already having been diagnosed [with OA] they’re usually very anxious about it… and they’re like “that’s it, I’m done”. And I’m like well not really. It’s there but so are your wrinkles and we live with them. It doesn’t mean that you can’t live life. At the beginning is generally that calming explanation part where it’s okay, you’ve got it, we’ll probably all get it at some point. It’s learning to live with it and managing it and keeping symptoms under control and keeping it from progressing (03 chiropractor early career).  I love to be compassionate with my patients and I don’t like to ignore these feelings because these are the most important. So I stop for a second and I show them my understanding and show them how much I understand the impact on their life, on their day-to-day… I try to calm them down to let them know that things could be done to assess with the pain (04 physiotherapist late career).  I think it’s also important to have a bit of a plan of what you might work towards in the session but not to get so stuck on that that you can’t pause and take the time to delve in deeper because often our clients are getting emotional. I think with osteoarthritis often not being heard because it’s often shrugged off by everyone like the physicians and family and friends because everyone just hears “it’s just OA”. And then when they come and actually get to explain like oh, I’m not able to prepare meals for my family anymore and that was a huge identity for me. I think just being really open, active listening and delving more into their story [and] acknowledging their emotions and the pain and frustrations but then seeing what positives we can build on that. You don’t want to spend the whole session with crying and emotions and frustrations because they don’t leave really with a tangible goal or something that they can work on… you don’t want to go so deep that the whole appointment is that and then they leave just really emotionally drained… There almost is a bit of an element to normalizing because I think lots of women don’t have a clear understanding that others are out there and having similar lived experiences and struggles. It’s nice to draw on “this has been very successful for some of our clients, do you want to try that?” … and give them some element of hope (06 occupational therapist mid career).  Tell them it must be so hard and I understand how it’s affecting you so greatly. I can see that this is upsetting and I can understand that you want to be able to do these things; it’s very difficult because it’s a big loss in your life that you can’t do these things anymore… it’s not uncommon for people to cry during our assessment… reflective listening, empathy, supportive, understanding and then focus on what can we do to make you feel better about the loss that you’re experiencing… When we start talking about what they can do to change things for the better I feel like it does help because we’re really problem focused… trying to help them to feel more optimistic about their osteoarthritis… realizing that we can do things to be helpful and hopeful. It’s not an all or nothing situation (08 occupational therapist late career).  Sometimes that’s even a hard thing to acknowledge that they are feeling down about the whole situation. So giving them a safe space to talk about because very often their son, daughter, a spouse is in the waiting room and they often don’t want to burden their family… I talk a lot about how positive attitude is so much a part of it. If we can feel we got some level of control and some sense of power within chronic diseases that can certainly contribute positively to the outcomes that we want to see for ourselves. But acknowledging that it is an inflammatory disease that some of it is outside of our control (09 physiotherapist late career).  Acknowledge what they’re experiencing and maybe that’s not helpful but to help them understand that these are common experiences or these are things that many people feel and hopefully they can feel maybe a little bit [less] isolated by that or understand that they’re also taking really good steps by coming and seeking help… We have access to so many resources and I would usually start with saying, this is the first step, learning about this, understanding… what kind of things you can do to make small changes to try to work on addressing the different barriers or challenges you have… if you can be curious and recognize that nothing changes quickly that do you have realistic expectations about what’s happening. There’s no magic, it’s just a matter of starting somewhere and letting yourself continue on that journey and be willing to keep guiding or be guided if there’s times when you need that support. Really this is a self-management journey and we want to support you being in control of what you do (10 occupational therapist late career).  You may not have time and you’re just kind of at the diagnosis stage... You’re doing a primary assessment. I don’t think that would be in my first round of questioning unless the person was obviously upset about it in the clinic, so tearful or feeling anxious or expressing those types of feelings. But definitely as time goes on in second, third, fourth or whatever appointment, yes we talk about how their mood is, how is that affecting because it becomes a vicious circle of if your mood gets low you’re probably not moving or exercising as much which then worsens symptoms of arthritis. So [it’s] definitely in my questioning bank but probably not a first meeting unless it was obvious in the interview (17 family physician late career)  ***Offer resources or supports to manage emotional reactions to OA***  Part of a treatment or dealing with [their emotions] would be to provide some strategies to manage those things otherwise to provide resources of how they can get help for things like that, because that’s not my area (05 physiotherapist late career).  We do in certain circumstances refer to a social worker to help with that aspect to things (08 occupational therapist late career).  I usually will direct them back to their physician to review their medications and if there’s anything that may be even contributing to those feelings and/or is there some type of intervention that should be in place from their doctor. I talk to them about counselling options and speaking with their family physician or seeking supportive counselling if that is an option and what’s in the OHIP realm and funded realm versus the unfunded realm... Try to circle it back into that whole circle of care. Look at other resources that they have too. Sometimes they have other resources that they are working with like a nurse practitioner (09 physiotherapist late career).  I might ask [my patients] to find themselves a psychologist…some people already have a psychiatrist or psychologist looking after them or a psychologist and I encourage them to continue or else if not, then I will see what I can do to refer them to a psychiatrist or psychologist…if a person is really tense and tied up in knots because of the stress and you loosen [their] ability to handle their stress they start to make their own decisions…and I see this as my treatments cause positive changes in their musculoskeletal system the emotional changes that follow that (11 chiropractor late career).  I have a counsellor who works in my clinic as well. So usually I will refer [patients] to her if I noticed any sort of distress surrounding the patient in regards to recently finding out about [OA] diagnosis or just even having difficulty living with it (13 chiropractor early career).  When I feel [their condition] is more complex or if they need more support Yukon has something called the Chronic Condition Support Program paid for by the government if someone has a chronic condition. They have a team of exercise physiologist, doctors, and other support staff. They do things such as supervise exercise programs or phone call check-in’s and they also have info-sessions that they give for certain chronic conditions. So sometimes I will refer them to see if they can get any more help…If I find they’re having a lot of anxiety and other mental health complaints along with the condition, I try to talk to them and make them feel heard and seen. But if it is something that’s more out of scope of what I can do for them, I do try to refer them [to other sources] (16 chiropractor early career).  I’ll find out if they’re having any outside help with [their stress or anxiety]. Are they seeing a counsellor? Are they seeing anyone else in the community that is helping them? And if they’re not, we have a social work counsellor here so I’ll ask them if they would be interested in meeting with her because that’s her area of expertise. And if not, then… we do talk about mindfulness, [and] I’d look into outside resources for them that they maybe interested in if they were wanting to look into some stress management, anxiety management type of programs (18 occupational therapist late career).  If there is anxiety about [OA], there’s some resources [and] websites to send people to… And I usually have a list of counsellors that deal with pain. The list is not very long here unfortunately. So there’s a few resources I use that they could call to access or at least talk to somebody if they feel willing to do that. The MD is not always the best place to send people to because I find they don’t have enough time and often times people don’t feel comfortable going there because there’s where they got a diagnosis and it’s not really well explained to them there and often times it’s been described as a disease and you have a problem and I can’t do anything about it. And then obviously harping on talking to family. Like talking to people about it and trying to encourage people to be [open], in some cases some people are not really open at sharing how they’re feeling with their family members or members of their household. And I think being straight with the people around them trying to explain to them that that’s important so that they can better understand what they are dealing with (20 chiropractor mid career).  ***Little to no discussion about emotional impact of OA***  In our medical history we’ve asked about any mental health history so they told us whether they have anxiety or depression or whatever it is but we don’t really go into details about how the OA is impacting those (07 physiotherapist late career).  We do talk about stress and we’ll do some discussion about mindfulness but we don’t delve a lot into the mental health side of things. From my perspective and what my scope is when I am treating people is that we touch on that but unfortunately we’re not getting heavy into that aspect of their arthritis and how it’s affecting them within the scope of what I do (08 occupational therapist late career).  ***Encourage patient to discuss with family physician***  I would advise that they connect with their family physician. I might put it in my notes to the family physician because we usually send our notes to them. Indicate it in my note that... activity is limited by osteoarthritic pain or knee pain, if they can be further evaluated if they’ve never been evaluated and managed accordingly. I just also discuss with the patient to have that discussion with their family physician because then they will refer them and then manage them in an on-going way (28 nurse practitioner mid career). | ***Assess directly through discussion or assessment tools***  I think [the emotional side] often comes out, whether it’s prompted or not is another question. But they will ask the patients “how are you doing? How is this affecting you life?” So if they make any mention of mental health concerns or experiences they will prod that further. But beyond that, I don’t know, surgeons, they are not mental health professionals so I think that’s where it ends frankly, unless obviously we’re very worried about it (30 executive healthcare late career)  ***Screening tools available to address emotions***  Questions describe how does your patient describe the pain experience. That would be an acknowledgement of pain regardless of early, moderate or advanced [OA] stage. The yellow flag questions are psychosocial risk factors for developing chronicity. When you go through that, if you find that there are significant yellow flags and maybe your pain characterization is quite high despite your physical examination showing high function and being an early stage of osteoarthritis. Then you need to go off into another tool to assess depression, anxiety and change focus… the screening is there so that you don’t miss that pain and psychosocial factors have an impact on mental well-being. But it does take you into a whole different set of resources because regardless of whether your mental health concerns are related to osteoarthritis or any other medical condition; once you swing into mental health support those supports are generic regardless of condition… the OA tool under non-pharmacological self-management refers to mental health counsellor if available (15 policymaker quality-improvement late career).  A lot of primary care practitioners have the electronic medical record which have a lot of flow sheets [with] a lot of guidance documents in there… So if there are some concerns [with mental health], you can walk them through the flow sheet of what it is that you need to get addressed in that visit itself… I do think that there are counselling tools that are available at point of care that the providers do utilize. I do think that they have enough in-house to manage at least the initial counselling. I don’t know about once the diagnosis [of a mental health disorder] is made, do they have the knowledge related to (19 healthcare executive late career).  ***Offer resources or supports to manage emotional reactions to OA***  [The clinicians] will always mention [mental health concerns] in their dictation back to the primary care provider. If there are talks of self harm or they’re just not coping at all, we have an algorithm that we follow with three levels: green, yellow, red. Level 1 is a mental health line that they can call in Alberta. Level 2 is the 24 hour crises line… level III emergency services will be engaged (30 executive healthcare late career). |
| **Manage uncertainty**  Offer rationale for tests or treatment, describe likelihood of risks and benefits using words, statistics or pictures | ***Discuss realistic expectations of prognosis or outcomes***  It’s a bit of [a] learning thing for people that for say a strengthening or stretching program that change doesn’t happen tomorrow but happens over regular sessions of exercise and give them some guidelines as to when they’ll start to see some results and hopefully that they can buy-in into it long enough to see the results and then realize the benefit that they’re gonna get (01 physiotherapist late career).  I explain to them that treatments and care plans may change based on the response of their body and that we have a variety of things to start out. I explain to them that there’s not a cure; they’re not gonna magically just have it gone one day. It’s something that you have to manage throughout life and you can manage it very successfully and there’s a lot of modern medicine and physical therapies and modalities to help with that (03 chiropractor early career).  For osteoarthritis I’m not giving them numbers but for example, for injections I tell them that the studies have shown depending on the level of arthritis you will get the response based on more arthritis, less response. I don’t give them percentages however I have some pamphlets in our waiting area for risk of supplementation with the percentages on them… I tell them that osteoarthritis is the most common form of arthritis [and] that it will never stop progressing but we could manage the symptoms… I base my discussion with them on arthritis guidelines and the new research… I explain to them it takes a minimum of 3 consecutive months to do the conservative management, to be consistent with it to be able to find the benefit [from exercise]… for people who are a little bit sensitive about the subject [of weight loss] we discuss about the impact on their joints… and how could weight-loss help with minimizing the pain but the most important for them [is] to understand that weight-loss is not gonna reverse their osteoarthritis (04 physiotherapist late career).  I go by the standards of best practice which say that first line treatment can be addressed through conservative management, mild to moderate arthritis can be addressed, however it’s not a certainty and I explain that there’s many factors that influence pain outside of just the arthritis itself such as pain tolerance, previous experiences with pain, level of conditioning, and it varies from person to person (05 physiotherapist late career).  There is always an element of unpredictability. It’s hard to medically know because people ask “is this gonna get worse or change?” Or they think it’s gonna get really, really, bad and it’s hard to predict the progression of osteoarthritis in certain joints or certain people… our whole clinic is based around ideas or options that they can draw on for self-management and we don’t really jump right to surgery at our clinic... empowering them with lots of education so they understand what osteoarthritis is. I think that that’s not explained throughout the medical journey very well maybe because it’s so common to care providers. They just say, oh you have osteoarthritis and then they just leave. [Patients] don’t know whether that’s a serious or a good thing or a bad thing or a thing that’s gonna get really bad or worse. I’ve had clients come in where one tiny change to a joint and they’ve perceived it that this is a death sentence because they don’t understand what’s going on (06 occupational therapist mid career).  I explain to them that the arthritis doesn’t go away, it progresses slowly over time. But that the rate at which it progresses is impossible to predict and that even that some people on x-ray their arthritis progresses but that doesn’t necessarily mean that their pain or function will, so from a management perspective, conservative management usually when combined also can have a major impact and improve their function and quality of life (07 physiotherapist late career).  Talking about pain and expectations around pain medication that joint [isn’t gonna be] pain-free. You could never promise that. But we talk about parameters… if it hurts more during that activity, if the pain persists for 2 hours after stopping the activity, those are warning signals and that’s your body telling you to be more careful with the joints… [we talk about] principles behind [joint protection]… Focus on discussing pain as a warning signal, not pain as something to completely fear… I feel that sometimes the fear of the unknown is worse… OA can progress slowly but there is a lot of unknowns but [what] we don’t want people to do is catastrophize and stop using their joints or focus on the pain which makes things worse (08 occupational therapist late career).  The first thing that comes up is whether or not it will go away or whether it can be cured. I think it’s important for people to understand that there’s treatment or management and not just hear this from me but understand this from other resources that they can access. That it’s a management journey. All the websites and all the literature we have, we can point them in that direction and say… this is what we understand is the best ways right now to manage this. No one has a crystal ball… nobody has any idea what will happen in the future… I try to be honest with people. I don’t want to make something like, oh it’s not a big deal… it is a big deal... I do tell people that if they don’t take these opportunities to change or to learn what they can then [do], there’s probably a missed opportunity somewhere along the way. So it is important to be not passive about what they do about their health but to be active to make decisions, no guarantees... It’s just a matter of understanding that they have a role to play in what the future will hold (10 occupational therapist late career).  The basis of correct work in my field is restoring the normal function back to the joints. If there is too much damage from the osteoarthritis it may not be possible to restore complete normal function. But any improvement in function even if a joint is degenerating will create a good symptomatic relief… I never use percentages [to explain uncertainty of OA progression]. I don’t think it’s necessary. What’s necessary is to talk about how the joints became degenerated, how the loss of normal function allows that to occur and how restoring function into the joint movements and so on is gonna allow the body to rebuild it because the body always wants to rebuild and maintain itself if possible (11 chiropractor late career).  I let everyone know that [OA] will present differently in different people and everyone responds to care differently. I usually recommend chiropractic care along with other conservative treatment that works for them. Usually I recommend physiotherapy along with massage therapy; those would be my first two in conjunct to chiro… [Patients] do ask if they will ever be a 100% pain-free and usually I would answer that question with there are maybe periods of time where that can occur. And there will be periods of time where they may experience a flare up and flare ups are less likely to occur if they’re doing certain things to manage their pain (13 chiropractor early career).  When we do the educational therapy part of treatment I talk about OA progression, how sometimes it can get really inflamed and feel really uncomfortable and other times you can feel really good. If regular exercise and other treatments that we’ve performed aren’t really helping, then sometimes I might want to refer them to a family physician for orthopaedic surgery… I always like to tell people if you do certain activities, you can get inflamed. Here are the things you can do to help manage your symptoms (16 chiropractor early career).  It’s pretty common to say it’s wear and tear, so it’s not going away, it’s not reversible. And I think people appreciate that. Or they ask it outright and I say, yah you’re right... We’re looking to treat you to function in a way that you want to. Or to get us to as close as we can to that goal. So managing expectations of treatment certainly is part of it because there’s no cure to it. And different treatments and how well they would work. When it comes to non-pharmaceutical things it’s only gonna work as well as the person’s gonna engage into it… physio or home exercises and movement in general takes participation from the patient. So, highlighting that active participation in their treatment is critical for best outcomes (17 family physician late career).  People will ask is this going to get worse? And I will say we don’t have [an] answer [of] whether it would get worse or not. What we do know is that we can provide you with information on helping you to do your activities with less discomfort and I’ll also get into fatigue management, because often fatigue is a significant factor, so I’ll question them about that and we’ll go over some fatigue management principles… say that they’re agreeable to having a splint and they might ask me is this gonna prevent it from getting worse. Well, in a lot of cases I don’t have that scientific research to say that it will prevent it from getting worse. But I will say that it will help to prevent an increase in discomfort while you’re doing that activity (18 occupational therapist late career).  I pretty much go with what best research is out there or suggesting that it’s helpful for treatment. I explain that people are different at the base irrespective of culture... and saying that the OA is not necessarily a disease, it’s something that happens frequently as we age because we are bipedal and we’re under gravity all the time. So a small change in a balance in a joint because of a balance of musculature etc., or injury will likely lead to progression of it or development of it… it’s all individual. So the idea is making people understand that there are things that can be modified to help if it’s really bothering them, then that’s how its gonna hopefully lessen progression possibly but really to lessen symptoms especially (20 chiropractor mid career).  [OA] is something we want to do a good job of managing because ultimately it will progress and it can be unpredictable. It often doesn’t progress in a linear fashion… But setting the expectation that just like it hasn’t developed overnight we’re not gonna be able to completely transform it overnight… also let people know it’s not like if the first thing we tried doesn’t work, it’s not a treatment failure and doesn’t mean we can’t treat your arthritis (23 nurse practitioner early career).  We usually tell people that OA is a mystery, we don’t have a crystal ball, we don’t know whether it’s going to affect a lot of their joints or maybe just one joint; it could be in their hands and not in their hips and their knees or it could be in the knees and nowhere else. Or that it could spread over time. Education is key in understanding what OA can do to you and how it can affect your life, [it’s] so important that they understand that there is no way to know what’s going to happen and that the best strategy is to be well informed about it and be able to identify symptoms… we can’t tell them how it’s going to progress or how bad it’s going to get but we do know that being proactive in recognizing the symptoms and managing it with exercise or physio or ice, that we can help them make their quality of life better, make it manageable, it should not be seen as a disease that gets you out of the workforce that cripples you for life (25 pharmacist mid career).  [I] explain that we don’t know how it’s gonna progress or how much it can… we’re hoping the goal of it is to allow you to be able to do certain activities possibly or with less pain… But we are not sure how much this is gonna do and the person might ask, “is this gonna progress”, yes, likely. How fast, we’re not sure, however what we’re working towards the goal is function and pain management (27 pharmacist late career).  ***Offer statistics about prognosis or outcomes*** For the surgery I have a blurb… I tell them how successful which is depending on hip or knee. So I give them the numbers. I give them as well the risks by numbers and what they are and the warranty on a knee or a hip and I show them the implant and they hold it in their hands (04 physiotherapist late career).  I don’t remember the numbers but there are statistics that [the GLA:D program] provide[s] in their education session… most often these statistics and percentages that we give are for success and failure on arthroplasty (05 physiotherapist late career).  I talk about arthritis [statistics] and timelines…I try not to scare anybody. I also try to let them know that some things are normal and I add in that if you stay active…and healthy…so when I talk about [OA] prognosis I also talk about how there are treatments that do help (16 chiropractor early career).  ***Supplement discussion with visual aids***  We definitely don’t go into the depth of showing them advanced OA joints or anything like that. We have pictures like this is what normal bones look like, this is what cartilage is… this is what cartilage does and this is how it works and then when the cartilage begins to wear - we don’t use “tear” anymore. Again, our studies on pain have demonstrated that sometimes those callus words that doctors will say, like you have the spine of an 80-year-old, that’s what [the patients] hear, that’s what they take in, that’s what they become (08 occupational therapist late career).  Once I complete an assessment and look at the entire picture, I do a lot of education using models and charts; a lot of patients don’t understand why [they] have pain in [their] big toe when [they] have a sore back. I use a lot of the models to show what a scoliosis does to the intervertebral foramen and how that can impact the L-5 nerve which happens to be attached to your big toe… sometimes it’s making them feel like they’re not crazy (09 physiotherapist late career).  I will say it’s the thumb CMC joint [with OA]. Then I will get a skeleton. I’ll explain the configuration of the joint or I’ll show it to them… I’ve also got coloured diagrams of a normal joint and a joint with osteoarthritis so I can show the ligament what effects there is on the soft tissue structures and by in that way explain the importance of joint protection (18 occupational therapist late career).  I do have models particularly of the spine but I do have models of shoulders and knees as well. And if I don’t have a particular joint model, I have a small skeleton which I often will show to patients so that they can understand what normal looks like. I’ll show pictures as well, if I do not have models, or skeletons are all pre-perfect. So they have an idea that surfaces look irregular and then explain my work if they seem to not fully understand what I’m talking about then I definitely go to visual, but most of the time I go to visual (20 chiropractor mid career). | *--* |
| **Share decisions**  Describe treatment or management options, assess interest in shared decisions, provide information to enable shared decisions, suggest factors to consider in making decisions | ***Discuss options for treating or managing OA***  I usually try and give them the best options based on what equipment they have, what they might consider doing for themselves in terms of exercise. What activities that they have felt comfortable with doing in the past or might consider doing. Some people are okay with going to the gym but a lot of people aren’t… make sure they know they can make options at home or in their neighbourhood (01 physiotherapist late career).  We try to explore, in addition to exercise, bracing options in clinic… things that can help alleviate symptoms, to make them more mobile, most functional in a short term so that they can make plans for time off [for surgery] at a different time… we try and manage with other interventions before surgery and that includes injections and any non-surgical option (02 physiotherapist mid career).  I have a checklist of things that I go through which includes weight management, exercises, injection, referrals. So, let’s say I send a referral out for further consult or surgery, then I provide them with all the other conservative management options and how they can go about doing those things, where to find it, how to access it, if there’s any cost involved, preparing them for their expectations as to the outcome (05 physiotherapist late career).  Lots of people come in with set ideas or lack of ideas so I think it’s important for the clients to get that education piece and truly understand what options might exist for them or what options might exist in the future if this starts progressing and then you may want to explore or come back or reach out again because I think it’s also important for people to understand that how they are now may not be how they are in the future and they might be better in the future, not necessarily worse (06 occupational therapist mid career).  When I’m giving recommendations [I] discuss the different options from a fitness perspective and exercise perspective… if people are either not candidates for joint replacements or don’t want to have surgery we recommend different conservative management and besides the GLA:D program recommending individual physio treatment, recommending injections of different kinds, more cortisone than anything else, weight management and on occasion recommending a dietician (07 physiotherapist late career).  I’ve seen whole families where they all march off to have acupuncture every week and that’s what they believe is helpful to them. And sometimes those hardcore beliefs… they get very committed and they’re not open to other approaches… sometimes it’s just helping people to look at alternatives and options and sometimes they’re open to it and sometimes they’re not (09 physiotherapist late career).  Whether it’s a splint or maybe it’s some education, maybe it’s reviewing some information; it could be teaching something. But it’s also a discussion with and about the role that the client will play in addressing that as well. So what is their responsibility towards addressing that and then of course there’s a timeline involved which would be however many sessions that would take and follow-up or reassessing or acknowledging when we’ve addressed something and anything further that needs to happen from there. Is this something you want us to address together? This is what I can offer. This is what we need you to be doing or will need to do (10 occupational therapist late career).  I discuss how we’re gonna deal with [making decisions]. For instance, if you have an OA situation and it involves the feet, the knees and the hips and the lower back we’re gonna talk about foot correction and I always fit them up for orthotics which will re-balance their feet, make their ankles and knees and hips work better. So we discuss all that along with some exercises if necessary to resolve the issue (11 chiropractor late career).  A lot of times physicians, family doctors or surgeons they don’t have a lot of time to spend with patients because they’ve got so many patients. We have one-hour allocated to an initial assessment. That’s a lot of time to hear the patient’s fears, go through the assessment, answer all the questions they are asking, go through an objective exam, review imaging, review treatment options… I feel like [patients] do feel reassured at the end of the appointment because it was comprehensive enough to capture… everything they needed to express… the patient comes in and I can see sometimes that they’re [in fear]… but then by the end of it, you go through their symptoms and impact and you talk about treatment options… different types of exercise, the balance between rest and activity, gait-aids or external supports like a brace. You may talk about heat versus cold. You may talk about physio in the community, like aquafit yoga. For some patients you may even talk about mindful meditation, relaxation, and stress reduction. We talk about activity modification… and when you review their imaging to determine options from a surgical or injection perspective. What are their risks? What are the benefits? (12 physiotherapist late career).  When I go over joint protection with them I’ll go over all the principles, be it hands, be it legs; say it’s hands and I’ll say splinting is one way of instituting joint protection principles. It’s not the only way. And then in discussing that with them, we have lots of samples. I will show them the samples, they can even try samples on to determine whether that would be of benefit to them. If it’s a splint or they’re wanting something to do a craft or knitting or taking their dog for a walk and the type of leash that they’re holding. I will ask them to bring that in so that we can have a look, we’re doing an activity analysis and we’re having a look at how they’re using their hands. And then be able to continue that discussion of whether a splint would be advantageous or not and they can try some on to get an idea if that would be something that they would like or would be amenable to trying (18 occupational therapist late career).  If they are here for particularly OA, I just give them suggestions on different options that can be done whether I’m the one that’s doing it or somebody else... Obviously you got to figure out if a person is comfortable with being touched, if a patient’s comfortable with a treatment being dressed or undressed. So if somebody is not comfortable with being undressed; that usually comes a lot in sign language and depending on the culture that I know that if a patient doesn’t want to go in a gown just to see their back, I will not send them to a massage therapist. But in that case, I do know some massage therapists that are open to treating over clothes so then I explain those possibilities to patients as well. And then they can decide to go that way or not if they want. And then just explaining every avenue whether it’s lifestyle, losing weight, or doing exercise or varying the level of activity or diet or doing chiropractic care to just help mobilize joint or just addressing the soft tissue that are creating more compression on the joint that could be creating more pain. I just discuss all those options and then I always get a feel for whether they’re more comfortable with a male or female and based on that information, I will refer out accordingly if something else is needed for treatment… I usually explain to women that a multidisciplinary approach is best… [and] that every practitioner is a bit different so their approach might be slightly different but typically I try to explain to them what a visit would look like if they were to go see a physio or a massage therapist. I always reinforce the fact that if they go somewhere else to a referral and they do not feel comfortable that they do not need to continue with that care. And they need to make sure [to] just be forward with the person that they’re working with because they’re professionals and they will understand that if the patient’s not comfortable or doesn’t like certain things that it needs to be communicated with that practitioner. At the same time, if they tell me that they have a hard time communicating that information with that practitioner, I’m happy to share information with that practitioner or having a discussion with the patient present with them or just me with the practitioner alone (20 chiropractor mid career).  I think a lot of people don’t take pain meds because they don’t feel like they should need to or they don’t like the idea of taking them a lot. So try talking [about] longer acting ones because… if you’re taking [Tylenol or Advil] for months or years on end, taking something every 4 to 6 hours isn’t feasible as if we can go to a longer acting medication… I’ll usually ask people what worked well for them in the past or what hasn’t? … people often know what worked for [them] in the past and what hasn’t from pain medication. I usually tell them we address this problem from multiple different angles and we don’t have to address all of those today or right away. But what do you think are the best steps that we could take now that will be easiest to implement and then go from there. For some people it can be like, exercise would be something I definitely can do… other people will say, I don’t want to start oral pain meds but then you can be like what about something you could put on topically on the joint? I try and frame it that there’s not one answer for this. It involves doing a few different things and approaching [it] from different angles in terms of strength, mobility and managing the symptom. So where do you want to start today as a first step because it can be daunting… changing your entire lifestyle that’s not feasible but you have to start somewhere… there’s not a one size fits all approach for OA… it *does* matter what you want and feel and what does and doesn’t work for you is important and can help people be empowered and being like, “I want to hear your feedback and I want to reassess this and we don’t have to do everything today” (23 nurse practitioner early career).  Providing all the options to them and making sure they understand… a lot of times people think there’s a magic pill that they can just take and it’ll make it all go away and we have to spend a lot of time explaining that there is no magic pill. There are different medications that can be used to try and manage the symptoms but exercise is also a very good way of managing and improving their symptoms (25 pharmacist mid career).  There’s the treatment algorithm… we start [with] the non-pharmacological, maybe diet, movement, sleep, staying active and then we can start going through the over the counter products which can include your Tylenol [which] is kind of first line, if it’s effective or not will be up to the patient… then we’re looking at our topicals… if that’s not effective I might be able to initiate a prescription for… gel… then afterwards we’re going to our oral NSAIDs if there’s no contraindications… usually I’ll say, here’s what the literature says. Here’s the order we go in things and often people with like OA will have already tried Tylenol. They’ll likely tried an NSAID and then it’s like well did that work okay for you? Or did you have any adverse effects? A lot of people will get stomach upset with the NSAIDs. So it’s like, okay well this might be an option for you but if you want to take this regularly, we might have to look into some stomach protection for you or making sure you’re eating with it. But typically I just show them here’s your choices, like first line, second line, third line and leave it up to them… It’s a shared decision, it’s a shared approach… they’ll come from a doc with a prescription, like oh, okay well why did you see the doctor today? Okay, is this a new medication for you? Did the doctor diagnose you with a new medical condition? And go from there and just to make sure it’s the right prescription… another thing too which is really important is family planning. With women of child bearing age, it’s like, are you planning on having children? Or are you pregnant currently? Or is there a possibility that you could be pregnant right now? If we’re gonna be starting like an oral NSAID, we should really rule out pregnancy before we start (26 pharmacist mid career).  ***Describe risks and benefits of different treatment options***  Some surgeons will say let’s do surgery whereas some might say let’s just wait until it’s bone-on-bone and you’re miserable. I think that there’s a case for waiting until it’s severe because the surgery itself is substantial and painful and so making sure that they’re doing it for the right reasons, but I think just the ability for patients to have that discussion about the benefits, the risks, what can we do in the interim, conservatively how long might that last… we use models in clinic and there’s a lot of back and forth because there’s lots of patients that can go for surgery, but they could continue on with conservative management meaning injections or physio or bracing. And it’s really a patient-specific discussion because sometimes people get to a point where their pain is limiting them enough that they’re not being as active as they could be. But because of not being as active as they could be they’re not experiencing as much pain. So, they’re still sleeping through the night. So, it’s having a discussion with those people that there’s a risk and benefit (02 physiotherapist mid career).  I do like telling patients that I’m here to help them understand osteoarthritis and the treatment options and hopefully understand some of the pros and cons of what’s available to them so that they can make an informed decision (06 occupational therapist mid career).  Often talk about pain management, weight loss and exercise is trying to keep the symptoms at bay or well controlled. And that ultimately over time, I usually explain it as some of the cushioning and lubrication in the joint, with wear and tear brakes down and once you start to get what’s causing pain which is areas of bone rubbing against each other or little bone... We can’t take that away other than surgery but surgery itself is invasive and causes pain and swelling and needs a lot of dedicated rehab. If we can try and address this with rehab and strengthening activities that fit into your life it usually works better for people than getting to the point where you can’t use your joint and we need to get to surgery and dedicated rehab that is really gonna interrupt your mobility and encouraging pain meds… things like joint injections which are a bit of a band-aid solution but sometimes we need band-aid solutions that gets your pain controlled to a point that you can better engage in some activity or physiotherapy or mobility exercises (23 nurse practitioner early career).  I’ll lay-out the information for you and then I leave the decision to you. I’ll discuss the risk and benefits of both and how efficacious is this one over the other but is this gonna be okay to take long term like for oral NSAIDs just with their cardiac risks (26 pharmacist mid career).  A lot of people worry about side effects of medication. I wanted to state in terms of put it in perspective of, okay, we’re not saying acetaminophen or certain ibuprofen or NSAID or the topical thing has no side effect but the perspective would be using it within the safety limit, and addressing the personal risk factor for those side effects. Are they having a previous history of stomach bleed, no. What is the age? Or do they have alcohol? Do they smoke? All those risks, personalizing it because I think when the shared decision moment comes, when my patient is making the decision, she can only make the best decision for herself when she have these information or have these perspective shared prior. If the decision-making stop because [the patient is] worrisome of side effect, my person maybe robbed of really not having the opportunity to assess the benefit to try it. I feel that as a pharmacist, laying out the perspective, potential side effect and really personalizing it and putting boundaries to how we can make this use of medication or whatever treatment safe is so critical and to present that at that junction when my person iis entering that shared decision-making. Otherwise it’s really not fair. They’re getting part of the picture… but they don’t know the full thing (27 pharmacist late career).  ***Prioritize patient over family opinion***  Some of the family members tend to decide on behalf of the patient themselves. I don’t accept it… sometimes I bring in the iPad [to translate] even with the family member in because I would like the decision to come from the patient themselves… so I stop and I talk to the family member and I tell them it’s not your knee, it’s the patient’s knee, and the patient has to feel comfortable going in for surgery… because I’ve seen lots of times patients really do not want to go for surgery but they are pushed by their families (04 physiotherapist late career).  Women, especially Indian women, are supposed to do everything and not complain. And even if they have pain their family members will minimize that pain… lots of families, they even don’t let them take any Tylenol or anything. Meanwhile they are really in pain, if they are advanced osteoarthritis and they are at the level where they cannot walk at all, they are in pain and… some people will deny for their parents to take medication... I think it’s not only convincing the patient, sometimes we have to convince the family members that it’s okay to give [painkillers] (04 physiotherapist late career).  I always try to make sure I’m addressing the patient rather than the translator. And then whatever they translate, I make sure that they actually translate and don’t give me a response prior to them translating it to the person… because a lot of times it’s the children of the patient that come in and they are trying to make decisions for their parents (05 physiotherapist late career).  I’m pretty strict and diligent because often times I’ll explain it and then the translator will say, okay. I always ask them to translate so that the patient is understanding what I’ve said and it’s not a family member just saying, oh I’ll explain it to them later… I get them to make sure that they re-explain it. So getting the patient themselves involved to understand what the recommendations are but also let them make a decision as to what they think they can and can’t do... Often times it’s my age generation or younger bringing in their parents and doing the translating and it’s very frequent that they’ll say, oh they hate exercise. They’re not doing exercise, talking to me about their parents and oh they’ll never do that and brushing it off that way. That’s pretty common (07 physiotherapist late career).  I’m definitely looking at the client when I’m talking and I’ll look between the two as I’m talking in English. I’ll be looking at their face and then the person I’m talking to back and forth trying to engage them and then showing as much as I can physically with pictures or… we have a kit, we’ll pull things out and physically show them. Or if we’re talking about a splint I’ll physically be showing them an option. So try and do as much as we can to ascertain that they’re actually understanding everything (08 occupational therapist late career).  ***Allow patient to lead next steps in care***  In the end, [the patients], with guidance from the therapist, need to lead what their next steps are going to be. When you’ve done a good assessment and identify what their major activities of daily living concerns are around the arthritis I think that’s the direction that the treatment should take… it’s a balance because you want to give them all that education, prepare them for the next steps but you can tell when a client’s not quite understanding the whole picture. Or if they’ve come in with really strong pre-existing ideas of what this therapy or treatment’s gonna look like, sometimes that’s hard to change. Sometimes that is gonna take more than one appointment because you’re spending so much time establishing rapport and trust and providing education… you have to be respectful of the client too because sometimes the next steps aren’t gonna be what you think they should be. So the next steps might be you really want them to come back for another appointment but they’re not going to even if you’ve tried everything, or your next steps might be, I think you would benefit from a custom splint or a physical device and they just might not be ready for that change or open to a device (06 occupational therapist mid career).  We know that when [people] make choices for themselves the power of the mind is very powerful. I’m all about like “of the things I’ve discussed today… Is there anything you want me to dig in more? Do you want more information? What would be helpful to you? You may have come across things that you find are really helpful, tell me about those”… some people want everything and some people want nothing and then there’s something in between… I will say to them you can leave here today and just think about all the things we’ve talked about and I always offer them a follow-up and say, in the follow-up you may want to circle back to some of the things we’ve talked about today and that’s okay. None of it’s a closed door (09 physiotherapist late career).  When I give a report of findings to a patient after examining them I make sure that they understood what I’m telling them. Without interfering with the decision [to continue with chiropractic care] because… it’s not my body it’s their body (11 chiropractor late career).  I tell [patients] that it’s always up to them. If they want to see the best results to be consistent with care and the suggested lifestyle changes that I’ve recommended (13 chiropractor early career).  Any treatment is the patient’s decision. I can’t force anybody to do anything or take anything. And I never try. I use other patients as examples because I find that people respond to that if they know that somebody else did something and it helped… I always say, if you’re reaching a point of pain that’s not ideal and you need to stop and back off, let me know and we’ll go over a different kind. But if it’s a little bit of discomfort that might be tolerable that may be helpful and we’ll follow up every 2 to 3 months. But I never force treatments on anybody. But I encourage it if I think it’s gonna be beneficial. And for my regular patients it’s because we already have a rapport built, so I guess they trust my opinion… I make sure that they understand the diagnosis and they understand the goals of treatment before we talk about what treatment is… [I] go over it if they have any questions about it or ask them to come back and ask questions if that’s the case. There’s always a follow up (17 family physician late career).  We always like to have a plan, some type of a smart goal and suggestions are put on the table. We usually give 2 or 3 options and say this is what we’re recommending, we let them choose what works with their lifestyle, their schedule and then we set a time and say, okay we want you to do this exercise for the next month or 3 months… but we need to have a plan because you don’t know how successful you’ve been unless you have a goal, you have a target. So we always talked about what it is they’re shooting for. Is it no pain in the knee? Is it minimal pain? Is it pain managed enough that they can sleep at night? You find what is of most interest to them and then that’s their goal and you help them develop their treatment plan and then you have follow-up for that treatment plan… You need to find a strategy or modality that [patients] do believe in and willing to try… physicians have to understand that in order for their plan to be successful the patient has to buy into it. They can’t be told what to do, they need to have a conversation and understand what they’re being asked of or what their options are and being able to play a role in the decision (25 pharmacist mid career).  You have to make a decision [that] the patient in on board with. You just can’t go by a treatment algorithm and say, well no, this is first line, you have to take this, what if they don’t want to take that or they’ve taken it before? Or if there’s some reason they don’t want to take it then you can’t choose that. On paper, yah it might be the best thing for them but if they’re not gonna take it then there’s no point (26 pharmacist mid career).  I think too, to give them breathing room… and hear from them. Maybe me talking less so they digest the information because they have their life commitment or story… how much realistically at this moment in time they’re willing to take care of themselves one way or the other, I think we don’t know, I cannot assume. I need to hear from them of their plan (27 pharmacist late career).  ***Tailor treatment options to patient circumstances, preferences or needs***  [I provide] all the possible treatment options for them to explore… [and help them identify options] based on what they’re comfortable doing… [I ask some patients if they want to] try a couple of exercises? I [will] demonstrate exercises… then [patients] can try [with me] and see if they are painful… because sometimes they’re fearful that the exercise is gonna make them worse… so I try to strategize. I also try to look at what they [have tried] and like to do and then I try to incorporate the treatments around that… there’s so many options for OA… I try to push or persuade all patients to be involved in some sort of exercise-based care in addition to any [other] treatment options available (12 physiotherapist late career).  After I discuss what my diagnosis is and what I think is going on I give them a few different treatment options that we can do over time… I try to give them different options and what I think might work best for them. For example, if someone came in and they were very swollen and very sore and they wanted something done that day to help ease some of the symptoms, I would offer something like acupuncture… and then get more into exercise and rehabilitation when they’re less inflamed and when they’re more able to physically do it… so it depends on the person… we always ask people [on intake forms] what their goals are and their health history… so I tailor treatment options [based on] their goals as well (16 chiropractor early career).  ***Support patients to pursue alternative complementary medicine or treatment options***  There’s a couple little hand-outs or things I’ve given people of who might be involved in my care… I try to introduce them early if people have adjunct providers they like go to. I try not to discourage that because they’re often meaningful for people, they’re like I like to go to naturopath or whatever…. These things don’t necessarily have a ton of “evidence”, so that doesn’t mean that they’re not beneficial for people and that they can’t be helpful in providing you pain relief or symptom relief. I often say I’m not really an expert in helping you navigate some of the adjunctive providers but you can certainly do that in addition to the things that we’ll try from a medical perspective to manage it. Often times those things get dismissed… from my perspective as a medical provider, that’s not going to harm and if it provides benefit, fantastic… and my domain remains the medical piece… it’s a several pronged approach so whatever helps and brings you comfort is great (23 nurse practitioner early career).  We currently, scientifically don’t have a solution to necessarily slow or prevent or change the course of [OA] or disease modifying and so my belief is taking that non-judgemental sense to take a full landscape of what [the patient is] currently doing… they might ask, “what about this?” And then often I may not know the strategies that they are employing, but once that communication is open, then there is a flow of what is being done or what is being considered. I let them know I don’t know… if this is for symptom control and they they do see benefit, then I’m neutral about that. Sometimes as a pharmacist too, when we ask about the complementary alternative or herbal stuff, I do let them know especially with [certain things] being ingested, it is challenging to confirm the quality or safety. So that’s another piece of efficacy and safety, my stance on it… The goal is to understand how to manage the symptom but not getting into unsafe situation (27 pharmacist late career). | ***Policies and tools encourage shared decision making between patients and providers***  I don’t know of what’s happening in practice but the policies that are being developed are patient and the family-centred care priority and there’s a greater push for healthcare providers to have a collaborative decision-making approach where the patients are being informed (14 government policymaker mid career).  It is using a motivational interviewing technique or a behavioural management approach where you’re asking, “what do you think you want to work on?” versus, this is what I want you to do. You always do some education. Here’s your diagnosis and here are a number of treatments that may help you feel better. What do you feel you want to work on? If that person starts off by saying, I don’t have the money for physio. I don’t have the time for group classes. You want to figure out what the patient is able to do and then partner with them in setting out a successful plan. There are tools [like The Action Planning Tool] that help you walk through those questions to be able to do action planning and goal setting but with the patients' goals and context foremost and not the providers saying what you want them to do. But that allows you to customize regardless of socioeconomic status… as far as a multi-ethnic lens, this tool has been translated to French but nothing beyond that… we may have some GLA:D programs that are provided in different languages according to the community and that’s about training the therapist with a cultural diversity lens so that they may have language or cultural skills. I believe the Arthritis Society has some multi-language access (15 policymaker quality-improvement late career).  ***Tailor treatment options to patient circumstances, preferences or needs***  [The clinician] will always ask what they’ve tried already and what they’re willing to try. Educating them about what’s available and then talking about whether that sounds like something they’d be willing to try… I don’t know if there’s a conversation about affordability because some of the options cost $300 plus dollars. There might be a conversation about support at home or barriers to accessing the recommendations… If a patient can’t do something for themselves, we’re probably sending them on for surgical consultation but there’d be a conversation about what support they have at home. And certainly we would talk to them about what they want. So what are their expectations now that they are in our program, some patients simply just want to see a surgeon and that’s the end of it and vice versa. Some patients are happy to have a specialist assess their joint pain and be sent away with a plan (30 executive healthcare late career). |
| **Enable self-care**  Set expectations for follow-up care, offer advice on self-care, provide take-home information, refer to other sources of information or support | ***Offer self-care education, advice, or support***  There is some teaching going on that is not all passive and hands-on treatment, it’s a lot of active empowering activities that will help people get better (01 physiotherapist late career).  We have models in clinic that show the progression of OA in the knees and hips and educating on the importance of staying mobile (02 physiotherapist mid career).  I like to go through what OA is, that it’s something that happens to most people as we age, there’s wear and tear... I get a lot of confusion from patients coming in; they’re like I have osteoporosis… they mix that up quite a bit. So then go on to explain what osteoarthritis is and it’s not oh, my bones are gonna break and I can’t do anything… I like to give a definition, make sure they understand it’s not osteoporosis (03 chiropractor early career).  I think education is key as long [as] it really is self-management… I’ve educated the client about what is osteoarthritis without being scary. What are the basics of what’s happening in the joint? … What do we look for? What are we expecting? And then just keeping the door open, if you have any problems contact me… It’s enabling the individual because it’s about self-management… they got to go out and make choices about how they do things or make choices about what’s going on (08 occupational therapist late career).  Helping them understand what the best practice literature says in terms of ways that they can work with their team, their whole healthcare providers; ways that they can look at activity, exercise that we know is supportive towards both pain management, joint flexibility and strength (09 physiotherapist late career).  We provide a lot of information. We can do it in some different formats… we have so many great resources and we have a number of great classes, education classes (10 occupational therapist late career).  People who were referred by their family doc with osteoarthritis to the clinic were put into the education program and they use to say, why am I not seeing a rheumatologist? And we said, because first line is education. It was classroom education, we used powerpoints with images. They came for an afternoon for 6 weeks in a row and they got education in a classroom along with exercise. We mixed it up, so that they got both. We had a physician explain to them what goes on in the body with osteoarthritis. Then we had a kinesiologist do exercises with them… every week they got exercises to work on whether it was for the knees, the hip, the shoulders, the hands, whatever. We used various modalities to educate them… there is a new clinic in <city name> that has just opened up and they’re resuming the education program… the fee was minimal and if someone couldn’t afford it, we would waive the fee (25 pharmacist mid career).  ***Offer self-care resources, education, or advice tailored to individual needs***  In a pharmaceutical sense, discuss what dosing would be and potential side effects and things not to do. If we’re talking physio or chiro or massage, prepare them that [it] may hurt a little bit after the treatment but that’s the goal that can actually mean we’re tackling the right thing and that we need to keep going on it. I always tell people that home exercises or your activities is essential, so if that’s not something you receive from another your therapist that you go to, then I advise that you go see another therapist…. Saying this isn’t gonna get better overnight. It takes time. It took you probably 30 years to get arthritis. So it’s not gonna be quick improvement. And also weight-loss… we talk about diet and how important [it is] because excess weight on the joints is obviously gonna cause issues. And then exercise is tricky when you’re looking at other than walking, right? If people don’t have the means to get to a pool or a gym or something like that, that’s gonna limit your availability of types of exercises but trying to cater it to that person’s abilities to access (17 family physician late career).  In terms of resources outside of surgery, if they need physiotherapy but they don’t have insurance benefits, if they’re from a lower socio-economic status or they just even can’t access those options we give funded options. So, either the GLA:D program right now or we look for OHIP funded clinics in the area. There are some OA programs through the Arthritis Society that we link people up to for exercise (02 physiotherapist mid career).  I tell them every person is very different. I look at a best educated guess of what’s gonna work based on their symptoms and how they’re responding to treatment and that’s why we make changes as we go through. We may start by saying, “do some walking” but we find that actually walking is really hard on them, so swimming is better... Following up to make sure like “did this work?” With maybe no longer playing basketball or no longer walking outside but using an elliptical machine because it’s easier on your knees… asking the questions and working with them is really the best way to do it in my practice… If there are language barriers sometimes I like to send them to websites that can be translated by Google (03 chiropractor early career).  We tell them the prices about the GLA:D Canada program [and] also about the injections… Usually for people who cannot accommodate, cannot afford the GLA:D Canada program… I send them to the Arthritis Society. And sometimes we send them to the Ontario covered physiotherapy programs (04 physiotherapist late career).  I explain to them what the best practice recommendations are and I try to find ways to integrate it into whatever it is they do in their normal life… for example, there might be a Muslim woman who has to pray 5 times a day and she has to get down on her knees; try to integrate some sort of activity in that fashion (05 physiotherapist late career).  There’s programs throughout the city that we can offer. The GLA:D program is one of them. And then recommending physio and depends on the socio-economic background, there’s a lot of people who don’t have benefits for physio which makes it tougher… often I ask if people have benefits that would cover physio… in [city name] there’s a pilot project with the GLA:D program so we can refer people and it’s covered. So that’s something I really focus on to get people who don’t have the benefits for physio to get in the GLA:D program because it’s a great program… it’s good for everybody but when it’s accessible to people who normally couldn’t afford it, it’s really nice that [they] can have the option (07 physiotherapist late career)  All of our sessions… are so different from person-to-person. Based on the standard x-ray referral I couldn’t tell you what I would do with them. It’s not cookie cutter. It’s not like, okay well that x-ray needs this splint and recommend this activity. That’s where the whole session with seeing people one-to-one comes in… where the client would report to me, what they want to do, need to do, have to do on a daily basis. That’s where the diversity comes in (08 occupational therapist late career).  Physio and all those things aren’t covered under our provincial insurance. So if they didn’t have private insurance that’s a different discussion because you’re probably more likely to go to a medication before you try physio because physio wait list here for non-insured individuals is probably like a year. I usually hand out the American Association of Orthopaedic Surgeon exercises for OA patients especially if they don’t have private insurance. I’ll give them that to start with. And that would change depend on their literacy and things like that if they couldn’t read the instructions I gave them it would be more important to get in-person physiotherapy or start them on a medication sooner (17 family physician late career).  ***Prescribe tailored physical activity***  Seeing what their daily activity living is and trying to involve them with this is your day, this is how it can work around. I try to make it very tailored in that sense and ask them “what are some times that you have available to do an exercise? When are you free, is it better at night? Is it better in the morning? Do you have someone to do it with?” Because it’s always better to have a partner, you tend to be more reliable on keeping up with your exercises… trying to plan a day or plan a part of their day where they can incorporate ways of managing OA... I’ll try to give them 3 or 4 exercises to do. I don’t like to overwhelm them. So I prepare them with that. We go through them in the office. I can direct them to videos of it on-line if they forget. I schedule a follow-up so I can see how they’re doing. I can see if they’re doing the exercises correctly because I usually ask them on the second treatment to do them for me (03 chiropractor early career).  Sometimes I teach some about the exercises myself and I review them with them (04 physiotherapist late career).  I offer them some specific exercises that the research supports in terms of their conditions (09 physiotherapist late career).  ***Provide take-home information to reinforce self-care advice***  Giving them hand-outs with standard exercises that we use but depending on the patient if some things are not needed in those exercises or they need something else then we do custom exercises and we will email them to them. So making sure that they have whatever resources we’ve talked about; that if we do bracing in the clinic that we actually teach them how to put the brace on and take it off and that that they know when and how long to use it. For some of the injections we have hand-outs from injectable vendors so that they have the information they need… they have a hand-out that explains the GLA:D program and we’ll give them the website if they want more information… it’s a lot of information [in the consult] so making sure they have stuff to take with them after that as a resource to look at once they’ve gone home (02 physiotherapist mid career).  We send electronic documentation [of recommendations] to their email… if it’s exercises there are pictures… same thing with our education that we send out if people are going forward for joint replacements… it’s a slideshow (07 physiotherapist late career).  I offer them exercises. I ask them would you like me to give you those and I’ve never had anybody say no… I give them a follow-up email just some video lecture material, very brief material that again, from the best practice in terms of backs in arthritis or backs in general (09 physiotherapist late career).  I will provide them with handouts that describe the principles of joint protection. If it’s a splint, I give them a handout on wearing instructions and cleaning instructions of the splints and I’ll be very specific with that… I will provide them, depending again on language, I will provide them with the Mary Pack Arthritis Program website if they’re wanting specific information. I will provide them with information on how to access our virtual education classes because they can self-register for those. With the splints we’re a very specific in terms of the wearing regime and we write that down as well as obviously talking about it. So written and verbal [instruction] (18 occupational therapist late career).  I often will try and get people to see something like a physiotherapist if somebody wants or I provide a ton of people [with] ortho info hand-outs and knee conditioning and hip conditioning exercises that they like, you don’t need to go home and follow this exercise program (23 nurse practitioner early career).  [We] have a discussion about OA... what increases the risk of an individual having OA… The different options, the management basically. And not just the medication part, the other lifestyle management that’s involved in that, and how to improve their mobility… then you reinforce with the brochure too; for those who can read it. When I discuss with patients, if I have a brochure or leaflet that explains, I give that to them. So they can always go back to it and read it if they forget because we give them a lot of information, so those brochures help them, it’s like a refresher (28 nurse practitioner mid career).  ***Help patients understand how to overcome possible barriers of self-care***  I think it’s important to understand what the barriers are for them for the next step too. Is it driven by finances if we’re suggesting a specific thing that cost money but might make a huge difference? [Like] “I understand that or I’m hearing that this isn’t something you’re interested anymore, can you tell me a little bit about that? Are you scared about the medication? Or worried about something? Or is finances a barrier? Or is it just right now is stress level too high really to work on the next steps?” (06 occupational therapist mid career).  Sometimes when people get into chronic pain situations and they’re already dealing with perhaps some very challenging personal life situations. A lot of it can feel very insurmountable. I try to help people set some goals and… I use a choices and change approach… “I have some ideas on how you could perhaps make some short-term changes that would allow you to begin to feel better. Would you like to hear them? … here’s what the literature says, here’s what I know from practice. Is there anything on this list that you think might be helpful to you? And then how can we go forward with it? What kind of plan do you want to make for yourself? And then how will we know that it’s actually been a positive or not?” I’m just trying [to] make it a bite-size approach to care (09 physiotherapist late career).  ***Referral to programs or professionals***  If it’s conservative management, providing them with the referrals that they need, whether it’s referral for physiotherapy (02 physiotherapist mid career).  I like to work with other health professionals. Whereas I’m a chiropractor and I do adjustments and some exercise, I’m not as trained as say an athletic therapist or a physiotherapist. So if they need further or more tailored or a very structured weekly meeting then I’ll refer them to a physio or an athletic therapist. I also find that massage tends to help them quite a lot and I work with massage therapists a lot too (03 chiropractor early career).  If the patient is open for [discussing weight loss], we could discuss sometimes even referring back to the family doctor for bariatric surgery depending on the BMI and some of them we just refer them to the Canada Nutrition Guidelines and back to their family doctor for some other nutritional or weight-loss programs in the community… we offer them different ways of getting their functional fitness prior to surgery which could be a home program, could be the Arthritis Society that could help as well with education. It could be the GLA:D Canada program. It could be just also physiotherapy… [if] there is a real functional decline and there is a real yellow flag or even if there is any red flags I tell them right away to their family doctor or send them to emerg if there is anything very serious. I follow-up with the surgeons if I need to if there are any red flags... If I find any fracture for example, or anything regarding to the bone. But any constitutional red flags I redirect them to their family doctor. Any yellow flags or any fall risk I refer them to the Outreach Program [which] will assess them for any assistance at home if needed or give them more guidelines about what they need to be more safe at home. And if we feel sometimes patients need occupational health and safety assessment… mostly we redirect to physio, GLA:D, home exercise program or the Arthritis Society. Our mostly redirected is to Arthritis Society. Also if during our assessment we find that another specialty is needed… I could send [them] to [their] family doctor with information about the website, where to refer with all the information to send the patient to the right location (04 physiotherapist late career).  We try and give people some tools that they can adopt to feel that they have some control over their arthritis or some pretty diverse toolbox that they can draw on in certain situations and it’s not a one prescription for everyone… we have some physical paper resources, we have some places that we can refer them to if they’re wanting to really delve into more some guided programs so we can self-refer to a few different programs… It’s a variety of some websites or hand-outs or us one-on-one guiding or if they really feel like this is a pretty big, big, big thing in their life and they want to work on it in a more guided way then we’ll refer them somewhere (06 occupational therapist mid career).  Within physiotherapy there’s certainly a very strong focus on osteoarthritis but I would say helping patients to access clinicians and services who have more of an interest in that area because therapists are very diverse in their practices. Some are very interested in sports medicine… trying to get [patients] to understand that it’s good to not just look at hiring a physiotherapist but to do some inquiries in terms of where their interest and backgrounds are and their experience in dealing with arthritis (09 physiotherapist late career).  I want to support them in being their best advocate for themselves to being able to manage and navigate the healthcare system in a way that’s gonna be effective. So I might point them in the direction, again that just helps guide them through that and supports them because there’s a number of fabulous resources for that, it maybe that the client wants to explore something else that’s not my area. Like for example, nutrition or eating differently or healthy (10 occupational therapist late career).  In some cases I have to refer [patients] to medical doctor’s or people who are orthopaedic people who can take them to a step further that goes beyond what I can do (11 chiropractor late career).  Usually I would write down the names of other practitioners in the area who I usually refer to… I am more specific in my referrals instead of just generally saying, you should try massage therapy, I would send them to a specific provider (13 chiropractor early career).  We might recommend seeing someone like a physiotherapist, sometimes we’ll have occupational therapists come into the home, depending on their age and stairs and that sort of thing… Sometimes you will end up seeing an orthopaedic surgeon and that doesn’t mean you’ll have surgery but to assess your joints and what your threshold would be for intervention. I often say, sometimes it involves our sports medicine doctor and things like joint injections which are a little bit of a band-aid solution… people are usually hesitant upfront for joint injections (23 nurse practitioner early career).  If they’re not on any medications, then I would advise them to see the family physician. If they are on something, I ask them to go back and speak to the prescriber to see is it managing your pain well? Is it controlling it well? … If it's not, then I just counsel them to maybe speak to their rheumatologist or speak to their family physician again and see if they can think of other things or other medications (28 nurse practitioner mid career).  ***Set expectations for follow-up care***  Because our patient population are elderly, most of them, they need to have someone to go back to if they feel they are not being attended to… We always provide them with our phone numbers to give us a call if they need any assistance in regards of their knees or their appointment… Lots of people use it of course but lots of people do not use it. They just get the comfort and they feel there is that attachment on a personal level because we understand how bad is the outcome of their arthritis on their life… Sometimes I follow-up with some patients who I feel are at risk of regression especially if they have very advanced osteoarthritis and they decide not to go for a surgery (04 physiotherapist late career).  Providing [my] email address and contact phone number for any further questions or, if they’re not to come back to me, I always let them know they can always contact me if they have any questions. If there’s people that are gonna be coming off my list, like I’m discharging them off and I’m referring them onto another area, then I always give them a timeline, get back to me within 6 months or a year, otherwise I’ll need to get a new referral (05 physiotherapist late career).  I think with that education piece it’s important for care providers to also help the client them feel confident that you will to the best of your ability be able to be an advocate for them or help them navigate what’s the next step if something else is going on or something new is coming up (06 occupational therapist mid career).  I always offer them a follow-up and I tell them that I keep their file open for 3 months so they can call back… [and] those who will say, well I’ve changed that I’m taking this medication and I’ll say, connect with your doctor on that, make sure they’re aware and it doesn’t do anything to impact the other medications you’re taking or your pharmacist… I tell them I write a report to their doctor, everything I told you is exactly what will be in the report. I offer them a copy of the report if they want it and I encourage them to circle back to their family doctor who will have that in-line with all of their other health issues. Because there’s such a connection to all of it (09 physiotherapist late career).  I want them to learn how to [self-manage] and to guide them and know that they can come back for a follow-up in 3 months if they had a question or concern or they needed support for something, they can reach out. We do emails and phone calls and things like that (10 occupational therapist late career).  We’ve created a hand-out for our patients [with] possible next steps that we recommend… I’ll say [based on our conversation today], this is what I’d like you to do for follow-up… I have a binder of resources [so] if I talked about pool therapy, I have a list of all the pools that are available that offer aquafit classes. If I talk about mindful meditation, I have a hand-out on mindful meditation… then I leave it open for any further questions, if you or anyone from your family would like to connect with us, or if [it is] confusing for [them] to understand or navigate, please feel free to call me anytime (12 physiotherapist late career).  I’ll give them a treatment plan that I send to them in an email and if we do any exercises or stretches I give them a hand-out and I also send them an email of <program name> so they can track their own progress… usually we’ll do that for like a month maximum and then when the plan is starting to expire, we’ll get a notification at that time and have the patient come back in so we can reassess (13 chiropractor early career).  I will provide them with information to take to the front desk to make another appointment. I will provide them with my phone number so that if they have any questions that they’re able to contact me before the next appointment (18 occupational therapist late career).  Say this isn’t something that we can fix but it’s important to manage and stay on top of and that you should still come in if the pain is bothering you because sometimes we have to adjust what we’re doing and ultimately there might not be a quick easy solution but we want to talk to you about how the pain’s affecting their life and also usually encouraging medications or looking at what medications depending on their co-morbidity are actually feasible for them to take long term… with medication, there are side effects to everything and everything we do is a trial. So sometimes if you start something if it’s not working at all, let’s stop it… I often say follow up is important. You should come back if things are going well and if they aren’t we should revisit this and not wait until the pain is out of control… come in before that so we can start to adjust and maybe add or take things away (23 nurse practitioner early career).  We want you to call us if something’s not going well or it’s going worse. Otherwise, we’ll see you in 3 months… always with support that they can come back, they can call you or they can come back earlier if they feel things really aren’t going well (25 pharmacist mid career).  Well typically with the new start of medication especially, it’s like, okay well here’s two weeks or here’s a month of medication and I want to see you back in a month or two weeks to see how you’re doing. And if you’re not doing well or you’re having side effects from the medication then come in earlier… I’d like to see you again in 30 days to see if we need to change anything or if you’re doing better… With a follow up, it’s always like see me, your doctor, a lot of people to get to see their doctor it takes six weeks. So the thing about pharmacists is that we can be accessed all the time, we’re very accessible. So at least it’s a good stepping stone if there is something a physician has to do or if they need a further physical assessment then they should see their physician but we can always answer some questions in the interim because most physicians aren’t gonna see them in two weeks (26 pharmacist late career).  ***Explain the benefits of physical activity in managing OA***  If they don’t have to have surgery, they can still try other things but with a decline in their mobility overtime that can affect the rest of their health. So you start to gain weight a little bit, then you start to have blood pressure issues; so really making sure that they’re aware that we understand we don’t want to push a surgery on them, however our main concern is make sure that they’re staying active to protect their heart and everything else in their health as well and that we do on a patient-by-patient basis… We do spend quite a bit of time on education and teaching our patients that exercise, mobility, moving is not gonna make the joint worse (02 physiotherapist mid career).  I like to make sure that they understand that movement is key; gentle low impact movement and that sitting is actually not a good thing or resting all the time (03 chiropractor early career).  I explain that even if you’re not deciding now for a surgery you want to decide later or even you never want to decide on the surgery, functional fitness is very important for their well-being and their ability to cope with the arthritis. Lots of people find comfort in this because whenever they have the pain in their hips or knees they think something is really bad and they guard it and pamper it and they don’t do anything with it. And the biggest challenge is to convince them that it’s okay to move (04 physiotherapist late career).  There’s lots of people out there that are scared. If it hurts they think if they do more they’re gonna harm themselves. And so doing the education just because it hurts doesn’t mean you’re harming your knee more, you have to listen to your body to some extent but getting used to any exercise program could even take a month for your body to realize this is actually good for me and that the muscles are taking over more and helping with your pain (07 physiotherapist late career).  What we do know about joints is that flexibility of the joint is certainly contributory to better joint health… And that muscle control is a big part of how our limbs are supported and the importance of elements of posture… understanding that exercise is actually going to potentially slow down the disease progress and what are the various specialties or other types of resources that they could pursue that there is some support for (09 physiotherapist late career).  There is a certain level of education around, you don’t want to overuse the joint but then there becomes a fear of healthy movement as well. We know clearly that healthy movement doesn’t actually exacerbate osteoarthritis. And that healthy movement is important as well as other techniques. We try and focus more on the basic mechanics of what is going on; the factors that come into it, of course genetics, environment, previous injury and abnormal use and focus on that it’s still a joint that works although it’s sometime painful (08 occupational therapist late career).  ***Include family members in care plan***  I would make sure that if I have a family member there that they’re on board for the plan and they understand what needs to happen. With some patients I would go to their daughter and say, I want her to come in next week at this time are you able to bring her? And I want you to try and help her do some of these exercises. So I make sure whoever else is there is on board. I always like to give people something to take with them like exercise instruction or some information about what’s going on. But generally I always try to get them to come back at least for a few more follow ups just to see how things are going and if they need anymore help with completing their treatment plan or getting information (16 chiropractor early career). | ***Offer self-care education, advice, or support***  We do these small group “Ask Anything” sessions for people who have come to at least one education session and maybe just want to know a bit about surgery. They want to know a bit about injections. They want to know a little bit about other options for them that they didn’t get in the class. So it gives them an hour to come and we do that with two educators. So we do an in-person one now and we also have webinars and but we do them as meetings so you can see each other... [they] are meant to be more small group and have a little bit more time for interaction… For us on the education team, I think the rapport gets developed because our classes interconnect. People might come to my hip and knee osteoarthritis class that I’m teaching in-person at a community centre, and then next week they can come back to our nurse’s class is teaching on pain management. And then our physios are going to teach on exercise and from the education standpoint our classes kind of interconnect, they build on one another, so [patients] have that ability to have follow up education without having like a one stop shop and they have one class and it's over... We do a pole walking class where they can come out and try pole walking with us and we bring the poles and people can practice that. So they have that opportunity but we don’t provide that one-to-one piece that unfortunately was lost when they closed the clinics which was a real pity (24 leader healthcare late career).  ***Offer self-care resources, education, or advice tailored to individual needs***  A lot of [our education] is based on patient’s feedback of what people are asking; like we had a curious about cannabis class that our nurse started teaching. With COVID, she started doing a meditation class and there’s a sleep and stress class. Then we had people who were saying that they were having difficulty now that they were working from home and setting up their computers at home. So I started doing a working from home ergonomic class on-line. But it’s not specific to a specific diverse population, like a cultural population specifically (24 leader healthcare late career).  Policies that allow for a rolling intake versus a specified time intake are going to allow for more applicants to take part [in self-management programs]… you can come and join one session, two sessions or three sessions before [becoming] self-directed and a lot of the programs [such as] GLA:D are not like that. You’ve got to start at one and complete six weeks and those six weeks might repeat four times a year. So [offering] resources and programs that don’t have those scheduled time barriers. Virtual programs are very important for that. Giving women the ability to make decisions so therefore in your action planning there has to be a menu of options that allows someone to choose what they’re comfortable with. I think in the literature even in the GLA:D literature they try to show more diversity in exercises for example, so that it becomes familiar and welcoming. At <university name>, we ran a Muslim swimming group… all the windows were covered during Muslim swimming time and it was only Muslim women who were allowed during that time… the privacy that they required was respected. So there are different ways that communities can promote activity and exercise that is culturally safe (15 policymaker quality-improvement late career).  We have an early touch point which we call MSK screening. Our wait time to see an operative surgeon is a year plus. So we don’t like the idea of a patient sitting on a wait list for 14 months only to come in to find out, well you don’t need surgery, so here are a few things you can do and send them on their way… we have one sports medicine physician who will assess the patient as an individual and send them away with an individual plan which you know is a checklist basically. They attend different things that a patient can try from injection, weight loss, walking aids, bracing, depending on the patient we recommend different things (30 executive healthcare late career).  **C*onduct ongoing assessment and follow up of what self-care options are/are not working***  When the clinics were open [before COVID] there was that opportunity to have that one-to-one connection with healthcare professionals and for that patient to then come back for further assessment. It’s not like going to a physio clinic where you go maybe every week and have one-to-one treatment. It was more of an assessment, come up with an action plan with this client or patient and then that client or patient goes and works on that action plan and maybe they come back to the clinic two or three months later and say okay, I’ve done this and l lost some weight. What do I do next? (24 leader healthcare late career).  ***Help patients understand how to overcome possible barriers of self-care***  My classes and focus on the client, so you’re working from a client-centred approach. All our classes are evidence-based, we follow clinical guidelines and I mean really empowering the clients to know that they have things that they can do themselves because I think a lot of people will my class and say, “I feel so much more empowered to be able to do something about this” because a lot of people feel that as soon as they get that diagnosis, the only thing they have to do is get a joint replacement and it's so not that way. So it’s kind of demystifying that and I think they leave the classes feeling, “I thought everything was over... [but] there are actually things I can do” ... giving patients that ability to empower themselves to do something about (24 leader healthcare late career).  ***Explain the benefits of physical activity in managing OA***  The GLA:D program... that’s a great hip and knee research-based program that you’ve got people who go through that and are thinking they’re getting a joint replacement and 7 weeks later they’ve made such improvements that they’re not. Really empowering the client to make sure that they feel there are steps that they can do to make those improvements but people say, oh well I have to take a drug or I have to get the surgery. It’s like, well no, let’s look at the research. What does the evidence really prove and it’ll prove that there’s thousands of research studies, if we can exercise, we can strengthen the muscles around the joint, we can offload the joint, we can keep that weight down, we can use our hips and knees in different ways to do everyday activities. That can make a really big difference. [When clients] have the chance to take the poles out [in our pole-walking class] and we go for a walk outside and they have that ability to say, “oh this really does work for me” and empowering them that there are other options out there (24 leader healthcare late career).  ***Provide take-home information to reinforce self-care advice***  We also send every patient away, surgical or not, with a guide that we call “Living your Best Life with OA” … it has general education about what OA is and it has managing OA… asking them to ask themselves why is taking action important. What will help me the most right now? What can I do to reach my goals? What can I do in the next two weeks? … it’s pretty basic stuff. Activity and exercise, healthy eating, managing pain, mental and emotional well-being and supportive therapies, like physio, bracing, heat and cold. And then there’s one or two pages per topic explaining more explicitly what that means and why it’s important. Nutrition, managing pain… we offer them various web resources… We talk about the GLA:D program. And then there are four pages of worksheets… identifying in writing who your motivator is. Considering why you might not do some of these things; I don’t have enough time, I don’t have enough money, I have too many other responsibilities to focus on. I don’t know where to start. And then there’s a treatment menu for people living with OA… There was a partner tool developed with this which was a “resource inventory” I think they called it… detailing all of the actual programs where they could access this care in the region or the zone… But maintaining that was a beast and I think they didn’t keep it up (30 executive healthcare late career). |

Barriers to OA care

***Clinicians****: What barriers do you face in providing first-line OA care or advice to diverse women? What other barriers might limit diverse women’s access to OA care and advice?*

***Execs/Policy****: What do you think are the barriers faced by diverse women in accessing early diagnosis and management of OA?*

| Theme | Clinicians | Executives/Policy-makers |
| --- | --- | --- |
| PATIENT-LEVEL | ***No family doctor to manage OA or refer to others***  Access to primary care [is] a big issue because most of our programs need a referral from a family doctor [and] a lot of women that are of diverse backgrounds or maybe a marginalized population may not have a family doctor… our biggest barrier is actually getting them in [the door] (02 physiotherapist mid career).  I’m in B.C. and I’m sure it’s all across Canada but I think there’s like 2 million people that don’t have access to a family doctor right now or nurse practitioner so that’s a huge barrier (06 occupational therapist mid career).  The bigger issue is probably if someone doesn’t have a family doctor then it’s harder for them to get a referral to access services like ours. It’s harder for them to get a diagnosis of osteoarthritis. It may be harder for them to access x-rays to get confirmation of that arthritis (10 occupational therapist late career).  A lot diverse women in Newfoundland do not have a medical doctor. No family doctor. So there is no referring physician if they need one and that is a barrier. I’ve had to send women to the emergency room before for a second opinion on things (13 chiropractor early career).    If they can’t get a family doctor, that’s a barrier. Dealing with that in a walk-in system and in an emergency room system isn’t really adequate because it’s a long-term management issue (17 family physician late career).  If I’m thinking generally about some of the barriers to care, it will just be referral, maybe some patients don’t even have family physician, it becomes hard to even get the care you need. To the person who will refer them and then the wait times when they are referred to see the specialist (28 nurse practitioner mid career)  ***Cost - no health benefits for therapy or cannot afford to take time off work***  It costs money. And people who don’t have a lot of money don’t come. We don’t see a big portion of the population because people that are struggling just basic needs, they’re not gonna be able to afford [physiotherapy]… financial restraints limits people and even to go to the pool or that sort of thing. If it was recommended and they wanted to, some people don’t have the transportation or the means to pay (01 physiotherapist late career).  Generally it’s monetary barriers. We’re not covered entirely by provincial healthcare here and not everyone has third-party insurance coverage (03 chiropractor early career).  A huge barrier is finances. Even in our program has limitations of who we can accept or not accept and so when you’re telling clients, just go to a private physio, most people don’t have money for that. Or they might not have extended health benefits that cover them (06 occupational therapist mid career).  Physio is super expensive, so the majority of people can’t afford it. To go to physio regardless of your socio-economic [status], to have to pay for it on your own… [it’s] too expensive and too overwhelming to get to (07 physiotherapist late career).  I think funding barriers are huge, that would be the number one… in terms of offering interventions if [patients] do not have OHIP funded care meaning they don’t qualify under the current Community Physiotherapy Clinic guidelines of the Ministry of Health, they are left to pay out of pocket for a lot of the interventions that are helpful and that often is just not possible (09 physiotherapist late career).  The Medicare system does not cover chiropractic properly. In fact, in Manitoba chiropractic care, the government contributes a very small amount to chiropractic care for 7 visits. For instance, my fee is $50 a visit and [patients] may get $7 or $8 from Medicare or something like that (11 chiropractor late career).  I’m private healthcare so they’ll have to pay out of pocket…a lot of the diverse women I treat who have OA are older, they’re on pension…so paying out of pocket is a really big issue (16 chiropractor early career).  Access to therapies like physio and massage and chiropractor… it’s probably a 12 to 18 month wait time for a non-acute [patient]. You can put them on the list but it’s not ideal. So people who don’t have access to private therapies like that would definitely really struggle more or don’t improve as quickly (17 family physician late career).  We’re very fortunate in that we can apply [for funding] if [the patient is] on disability. We can apply to the Ministry for funding for custom-made splints. And we’re often able to get funding but not always. So I would say cost for splints [is a barrier]. Our treatment fortunately people don’t need to pay for, it’s part of their healthcare plan. For sessions they don’t to pay for but any splints that are provided for them they do, so that would be a barrier if we can’t find funding or if they’re not able to pay (18 occupational therapist late career).  Probably cost honestly and not having any subsidies; depending on where they’re coming from are; if it is an immigrant or not or somebody that’s been here for a long time. Depending on the job they’re in, some of them don’t have insurance coverage. So the cost will definitely drive some people away from it (20 chiropractor mid career).  I think cost is a big one especially when you look at arthritis management. Physiotherapy is really hard to get covered. You can get like 6 OHIP covered sessions and you wait forever to get into one of the covered clinics. Joint injections aren’t covered by OHIP… The resources that are available for people and what we know works well is mostly stuff that’s not covered by OHIP (23 nurse practitioner early career).  The challenge with the GLA:D program is the cost. A lot of people do not have insurance and these immigrant women probably would not. And the cost of the GLA:D program is usually quite exorbitant. It’s geared more to the younger population who have early diagnosis of knee or hip issue, but it’s also restricted by its availability… [Also] some of these doctors will recommend specialty topical for sore knees and [they] cost $200 and the jar only lasts a month. I don’t think doctors realize that they give the prescription to the patient and the patient goes to the pharmacy to discover it’s gonna cost $200 and says, whoa, no I can’t do that. Then I may often get a call of I saw the doctor and this is what he suggested but I can’t afford this. So we have to go back to the options and what works for the patient, what is feasible (25 pharmacist mid career).    One of the big barriers is [drug cost]… The topical compounded prescriptions can be quite pricey if you do not have coverage. So most public plans will cover those and I know a lot of people I do see have the federal coverage if they’re new to Canada… But a lot of people I’ve noticed, don’t have drug coverage… we’ll start with our cheap over the counters, they’re reasonably priced… But when you’re looking into getting a compounded diclofenac like 100g of 10% you can look at like $50-80 depending on where you’re getting it… I think access to physio and OT is pretty limited, even dieticians. Most of them have to be done as outpatients or through Alberta Health Services. There’s really not too many and if there are private clinics it’s expensive… And even physios in Alberta now, they can write orders for x-ray but it’s not covered by Alberta Health anymore (26 pharmacist mid career).  Coverage [for medication]… we lack structured exercise program and especially with the pandemic situation there’s the parks and rec program… the GLA:D program, access is a challenge because of coverage, payment and whatnot. And same is if we talk about weight management healthy eating, so coaching on that is also dependent on access (27 pharmacist late career).  ***No or limited technology access or ability***  Pool exercises are a pretty standard modality for hip and knee problems… a pool needs to be available or affordable. We have a pool in one community here and it’s a retirement community… it’s overused. There’s too many people in it for the aqua-exercises… There’s almost no personal space. I know that has turned off some people. Especially with COVID concerns people are not keen to be so close to people… they’ve given up the exercise that has sort of held them together for a while. And with the restrictions… they’ve closed down the gyms for a while but also people haven’t resumed because it’s so hard to get back into it… or maybe they’re still scared of exposure because there’s still a lot of COVID around. So there’s a lot of reasons why people are less active. They’ve given up what they did have established (01 physiotherapist late career).  In [the] pandemic, when we look at programs in the community, a lot of groups got put on hold and haven’t been restarted. Like fall prevention groups and older adult exercise groups or mobility groups, run by the OT’s and PT’s or maybe medical providers. A lot of stuff got put on hold for pandemic and just hasn’t really picked back up or if it has it’s picked up in a very limited capacity and there’s massive waitlists for it now (23 nurse practitioner early career).  Our program does have a lot of Zoom classes and those are well received but they are a barrier for some clients that don’t have technology or internet or even a cell phone or they’re really marginalized or English second language because all those classes are in English (06 occupational therapist mid career).  We have [arthritis program name] classes through [health organization name] that are offered B.C.-wide. But they’re English and they’re offered on Zoom which is sometimes tricky really if you’re not tech savvy… it’s almost like COVID has fast-forwarded everyone’s tech ability to be able to least log onto Zoom which is great in some ways and different in some others (08 occupational therapist late career).  The time it takes is a barrier and whether or not it can be done virtually or on-line versus in-person I still believe that in-person education and care is far better in the early stages of OA than virtual. But it’s that accessibility for treatment (25 pharmacist mid career).  ***Women delay seeking care (unclear who to see, time constraints, other commitments)***  We have seen in clinic women that have OA they could use surgery however, for whatever reason, let’s say they’re a primary caregiver for someone else or they have children in the home and they can’t afford to be off work or even off their feet for recovery from surgery… there are some men that are also caregivers but generally that is something that pops up for our female patients, “I can’t have surgery because I’m looking after these people” (02 physiotherapist mid career).  Making sure that they either come in or that they go see someone else like physio or me or they spend the time at home to focus on it. As we work with diverse women I find a lot more of them are working later in life or they’re working multiple jobs and so sometimes it’s hard to get the time for them (03 chiropractor early career).  I think that diverse women sometimes present really, not tough, but they put themselves last so they’re often potentially lower socio-economic status or working a lot of jobs, they’re raising children, they might be single moms. A lot of the diverse women I work with are grandparents but they’re raising their children’s children and they have a lot of stresses going on in their life. So they’re not the very good at putting themselves first or prioritizing what’s going on with their health (06 occupational therapist mid career).  There’s so many other issues that they have to deal with and get used to and learn about, that something like fitness is probably on the back burner for a lot of people because they’ve got so much else on their plate (07 physiotherapist late career).  I’ll try and encourage people to book a dedicated visit for themselves, when you think about women and often if [they’re] there as a caregiver for others or with children or with other priorities I feel like often times… [they] bring up this concern at the very end of the visit, like oh by the way, my knees have been driving me crazy [and if] they’re being seen with other people [they] don’t always necessarily get that dedicated time (23 nurse practitioner early career).  They may not be able to access a healthcare professional to help them with their OA care. Whether it’s a financial issue? Whether it’s work, it’s family? I think the biggest barrier and frustration is that these people could be helped but these women may not be able to get to and utilize the services and the supports that are out there for them… if they have lots of children or they’re a daycare provider for grandchildren then they’re restricted. They don’t have the flexibility to just go for an appointment. And if they’re working and they can’t give up income to come to an appointment, that is a barrier as well (25 pharmacist mid career).  A lot of the women may not prioritize their health, their self-care and especially if we are talking about exercise program, eating right and all that… More in certain groups. If we’re talking about the group that is still mobile, able to do certain things but needing to care for the family, the spouse or sometimes in the 60’s or even early 70’s, a lot of OA patients are still working. That could be a barrier in terms of yes, I’m in pain but I’m not able to dedicate the time, the energy to go through these first line measures or management strategy (27 pharmacist late career).  Mobility itself can be one that limits people from coming in. I think the chronicity of OA is such that people will live for a while in pain and things slowly get worse. If you fall and end up with a huge swollen knee, people come in and be like hey, is this broken? But if you’ve been living with it for months and it’s sometimes bad, sometimes good, I think it can be like kind of a barrier that people don’t really know when they should come in (23 nurse practitioner early career).  Whether it’s the diverse women or other people, they all need to be educated and have the access to preventative care or proactive care and education. They’re so many women sitting at home suffering because they don’t know what to do, where to go or they’re too shy to complain to their spouse or even to go to the doctor… another fear that they have is they’re afraid of surgery. When they have knee pain everyone assumes they’re gonna have a knee to joint replacement and that’s not true… so there’s that fear, that I don’t want to say anything because I don’t want a joint replacement (25 pharmacist mid career).  Transportation to come to do appointments [is a] barrier (16 chiropractor early career).  Geographical barriers; you can’t get from A to B. A lot of my patients live far away from my practice so doing in clinic appointments all the time is not a feasible option. So you may not be getting as timely care as appropriate (17 family physician late career).  Transportation for patients who come in and see us. We’re doing more in terms of virtual care but not everybody is adept at Zoom or FaceTime, those kinds of things. Sometimes it’s proximity; we are in <city name> and it can be difficult for people outside of the city to make it in to see us. So we do look for other resources on <geographic area> for them… Unfortunately [they] would have to pay but [something] like a hand therapy, a private clinic that might have a hand therapist for doing splinting (18 occupational therapist late career)    Distance is a barrier as far as how far do they have to go for it because it’s the transport or transit cost or the whole idea of how do I get there if I don’t have a car and I can’t drive myself. Accessibility of course, if they can’t do stairs, is there an elevator, is there a ramp? (25 pharmacist mid career)  The time it would take them to book Wheel-Trans, and some want to be accompanied, and I’m just thinking about a specific patient who it’s only her friends who accompany her most of the time and [worried] her friends think of [her as] a burden, so requesting that they get somebody else to accommodate that patient for her appointment. So those are kind of barriers, because she has no family members, so the only time she can [go] would depend on if there’s somebody available to accompany her for her appointment (28 nurse practitioner mid career)  because pharmaceutical care in Canada is so much different than other places, so the expectation sometimes are difficult to establish because in a lot of other countries the pharmacist just gives the drugs and that’s kind of it. They don’t really understand the disease state and it’s kind of like, I want to talk to my doctor… but here we have a little bit more of an expanded scope, so establishing that we’re able to do a little bit more for them I think, especially here in Alberta. Like I have my prescribing authority, so I can initiate drug treatment without having to see a physician and something like osteoarthritis is something that I would be comfortable prescribing medication in… so that is probably one of the bigger barriers is just knowing what we can do for them and that we’re not just there to dispense the medication. We’re there to actually assess if the medications indicated [are] safe, it’s gonna be effective and if they’re gonna have any adverse reactions to it, just like the classic 4 categories of assessing a prescription… Indicated, effective, safe and then adherence (26 pharmacist mid career).  Even though we try and get our name out and the word out, and we do have a healthy referral base; lots of people still don’t know about our services (06 occupational therapist mid career).  We get people in and they say, oh I’m gonna tell my friend [about this program], but that’s so minimal compared to what the grand scope needs to be… [a patient said to me I’ve] shown others this great multi-lingual program based out of [city name] that does all of Canada where they get on-line access education services; I would say, I didn’t know about that and that’s fantastic but how do we get that communicated to everyone who’s actually dealing with arthritis. I don’t know the answer to that (08 occupational therapist late career).  ***Language barriers prevent help seeking or challenge communication***  Definitely language barriers can be an issue… I think there are some nuances that are lost within [translating] (03 chiropractor early career).  There’s a language barrier, so [the] availability of interpreters [is a barrier] and even if you explain things in English sometimes people don’t understand what it is that’s being talked about when you’re talking about medical terminology (05 physiotherapist late career).  I think sometimes the message gets misinterpreted or that some of the medical information it’s hard to translate or hard if their family member doesn’t have high medical literacy. Then we need to adjust how we’re explaining things and really bring it down to a lower grade level. So, we’re not using a lot of technical terms that are gonna have to be translated or don’t translate as well or easily. Because often the person that’s accompanying, if it’s a family member their English is second language as well. They just have maybe a little bit better English than their family member… because if they’re not understanding what we’re talking about then that’s not gonna be very valuable (06 occupational therapist mid career).  We have English and French hand-outs only… one of the negative things I find about the GLA:D program however is that there can’t be a language barrier, other than English or French (07 physiotherapist late career).  We have some other resources… a book called *Positive Coping with Chronic Conditions…* but it is English; lots of reading, higher education level so that wouldn’t really be appropriate if the person wasn’t as fluent in English reading and writing; it wouldn’t be as helpful… we do have translation services available to us as well if language is a barrier. But I don’t know of any classes offered in different languages… We do some of our hand-outs in different languages but the tricky thing as an occupational therapist is you’re hoping there’s a straight crossover of the translation. But you’re not always entirely sure (08 occupational therapist late career).  One of the gaps, we’ve been talking about this for a while, it’s just how much information is available in other languages that’s been translated over. I know that’s on our radar. It’s just a matter of resources to access interpreter services to be able to help translate documents… Accessing interpreters on a timely basis or being able to be creative about how we can present information to people when they have a language a barrier because I think the barrier’s probably more in accessing our services opposed to us actually working with clients (10 occupational therapist late career).  My job is to see what the problem is, identify how it got there and give that person information on how they can correct and restore function and sometimes that is difficult to do because of linguistics (11 chiropractor late career).  Here in Yukon the government has some OA info-sessions that they put on for free [and posters] in community centres. That’s helpful but it’ll be a bit harder for immigrant women especially those who don’t speak or can’t read English so they wouldn’t be able to understand the posters or know about the info-sessions… the general awareness of what OA is and the different areas of your body it could be in… and where to go first or what to do… that information maybe isn’t getting across to diverse women (16 chiropractor early career).  Language barrier can also be one obviously in <city name>. We’re pretty good in our big institution about having resources available in different languages but when you look at the richness of your conversation, the amount of health literacy… I use translators but I just feel like when you look at trying to set expectations and understand patients’ preferences… some of that can be lost with language barriers depending on if people are using interpreters (23 nurse practitioner early career).  Making sure they understand is probably your biggest challenge whether it’s a language level or whether it’s the language itself and they speak a different language (25 pharmacist mid career).  I’ve worked in pharmacies [where] the pharmacist speaks Arabic for example, where the majority of the patients will speak Arabic. So when I’m filling in for him, they’ll be like “you know what, I’ll come back next week when they’re here”. And that’s fair too, that’s their pharmacist as well… But I think the biggest part is having somebody there who can interpret [the information] to their caregiver or their relative or friend (26 pharmacist mid career).  The language barrier is one of them. If you’re going to speak to somebody about their health condition and they don’t understand English as well because that’s [not their] language of communication (28 nurse practitioner mid career).  Sometimes I prefer the interpreters much more than the family members because I would like to explain everything about the surgery, everything about the other conservative management in detail and I would like them to understand every single thing that I’m saying. And if I feel the family members are not providing full translations for my patient… sometimes some family members are really pushy (04 physiotherapist late career).  I have to just have faith that all the information I’m saying is actually being translated which can make things tricky particularly if there’s a family dynamic there that you can feel (08 occupational therapist late career).  ***OA dismissed by clinicians due to age, not considered serious, particularly if women do not self-advocate/ask questions***  At a primary-care provider level, I think OA gets brushed off a lot, I mean we hear it from our clientele all the time, that they’ve been told there’s nothing they can do and that they should just live with it and there’s no resources provided at that level (06 occupational therapist mid career).  I feel like a lot of doctors don’t take things seriously… I really feel like the lack of diagnosis and then the lack of response from the primary care physician is one of the biggest barriers… getting the diagnosis and getting the referral or the knowledge of the doctors of where to go from there as well, like we want to see people with small things happening before they become huge issues in knee and joint replacement (08 occupational therapist late career).  We are an outpatient clinic and we see inflammatory arthritis and complex OA, is what our referrals say. So sometimes the barrier is that people don’t get referred and at times, it’s the patient has heard from their neighbour that they were here and that it can be a good resource for them. So barrier is not getting the referral, is the doctors thinking that it doesn’t constitute complex OA. And as OTs we always argue that, because a CMC joint can be very complex in terms of the effect on an individual’s [activities of daily living] (18 occupational therapist late career).  One of the things some of the women talk about is, okay I do discuss first with my family physician and nobody seems to understand that the kind of pain I’m living with and the medication is not working well (28 nurse practitioner mid career).  When I did the education program for people with osteoarthritis I used to say, never leave the physician’s office with a prescription that you do not intend to fill because it wastes their time and your time. They’re thinking you’re gonna take it for 3 months and come back and report on how you did. Whereas you’re gonna go 3 months more in pain and come back and say I never took it. I stressed with them that they need to be on board with whatever is recommended. And if something is recommended that they don’t agree with, that they need to be strong enough to speak up and [say] I’m sorry, I can’t do that. Sometimes having a family member with them helps them to express that because they may whisper in a family member’s ear, I can’t do that. But they don’t have the courage to say it to the doctor (25 pharmacist mid career).  ***Challenge adopting new or unfamiliar activities such as exercise***  Patient’s knowledge on what they can reasonably expect I think is another barrier… sometimes people when they get into hopelessness they just don’t feel anybody has an answer… So helping them understand what the reasonable expectation [is] from a best practice approach and that maybe you just didn’t get in the hands of a clinician and then what a clinician could reasonably do for them. I find when people are in the hands of people who really have an interest and an expertise and then they feel better they’re like oh wow, why didn’t I find you sooner. So that whole lesson of even knowing what to expect I think is a real barrier… someone who’s got a really chronic condition and real hopelessness and they’re just looking for someone to solve it. They’ll tend to lean into those more passive treatments… there’s a great attractiveness to someone fixing me and passive care. I think that physio tends to sometimes work in contrast to other healthcare professionals who provide more of a “I will apply this to you and make you better.” Where physio tends to generally take a much more of a self-managed, “let me teach you, let me help you be independent, let me guide you” approach… But some [patients] have leaned in and they’re not better and they’re then opening up towards the more self-managed active interventions and a lot of them don’t want medical management, they don’t want pills (09 physiotherapist late career).  Their expectations [is a barrier]. Is their expectation to be pain-free because sometimes that may not be a reality. Depending on severity of their symptoms. Is their expectation to be pain-free without putting in active rehab exercise. Meaning they’re looking for something like an injection or a pill that’s gonna take away all the pain but they don’t realize that it’s a combination of everything. The pill or the injection will take away like say the acute symptoms but you need to be doing exercises for long term maintenance of your OA symptoms (12 physiotherapist late career).  Often times if someone’s first diagnosed or you have a flare, you’re motivated to get on top of it. But so many times people are like, well I’ve tried that, it didn’t work. And [I’ll] be like, how much did you try and how often and for how long? Are we taking it consistently and in optimized doses? Did you do physio like 3 times and it didn’t make a big difference and was that active or passive rehab? So many people they’re like, I’ve been doing rehab for 6 months and I’m like, could you tell me about what you do in rehab? And it’s like, I get ultrasounds, I get massage, but none of that is actual strengthening or mobility, it’s not active rehab… I think we use often these big terms too, being like you should do physio, you should do exercise and people don’t know necessarily what that means as a dedicated therapy for OA. Like exercise isn’t just do any activity… it’s movement and strengthening that’s targeted at whichever joints are affected and strengthening those surrounding muscles and limiting impact for them. I think we sometimes use umbrella terms and people don’t necessarily know how to tailor those to [OA] (23 nurse practitioner early career).  Consistency from the providers but also in the patients… for doing exercises or go through phases of being really good about weight loss and diet. I think it can be a lot of, like yah I did that for a while. [We ask] were your symptoms better when we were doing all this? [They say] yes, but you know then life happens and stress happens and changes happen and I think the fact that it’s so chronic the consistency in it can be hard (23 nurse practitioner early career). | ***No family doctor to manage OA or refer to others***  The biggest barrier is do these individuals actually have access to a primary care provider. We’re in a crisis here right now where 1.3 million Ontarians don’t have access… I think access is a huge, huge barrier to care (19 executive healthcare late career).  Your first place to go to is having a family doctor... they can’t get a diagnosis unless they have a family doctor and we’re all pretty aware of what a disaster that is right now. People don’t have access to a family doctor... You can’t get an x-ray; I guess you go to a walk-in clinic. It’s your only other option. So I think that’s a major barrier, getting that diagnosis from the beginning (24 leader healthcare late career).  [a barrier] for us, it might be referral… we would very much value having and helping that [diverse women]. I don’t know if we get as many referrals, so the barrier is maybe primary care, all things kind of do start with primary care and are as many diverse women getting knowledge of our program? (29 executive charity late career).  To access our care you need a primary care physician, so whether or not there’s any data to suggest diverse women don’t have access to primary care. If they don’t though they can’t get here, so that certainly would be a barrier. So if you’re new to Canada, don’t have a family physician, you would have a hard time getting your foot in the door here. That’s just be design of the system and the way it always has been and those words make me think it’s probably not the right way to do things anymore but that’s how it’s been (30 executive healthcare late career).  ***Language barriers prevent help seeking or challenge communication***  Language is a huge barrier to care… individuals coming from other countries who maybe don’t speak the language, they’re not going to necessarily know what to ask and I think that becomes a challenge. Primary care is also not funded for translation services for Community Health Centres. So even if women do come in, often they’ll come in with their kids but they won’t come in with a medical translator or they won’t come in with somebody that can actually translate what’s going on. And then the primary care provider doesn’t necessarily know what to ask because they don’t have a good full picture if again, they’re lucky enough to have a primary care provider. So I think those translation and access to culturally sensitive language tools are a huge problem (19 healthcare executive late career)  I think culturally, it could be language barriers, not having the ability to express exactly what they’re feeling or not feeling heard or you go to your family doctor and you’ve got multiple health problems and that might not be if you’ve got high blood pressure and you know other health issues that that ends up at the bottom of the list. It doesn’t get addressed (21 policymaker quality-improvement late career)  Obviously there aren’t gonna be enough health professionals speaking the languages of the people that need care. So there’s a language barrier for sure… The language disconnect works both ways so as a clinician I’d be frustrated of not being able to the mother tongue of the person (22 executive research late career)  If their first language isn’t English and they’re looking at the classes and say, oh it's all great, but you don’t have it in the language I speak... even if you have our section on the website perhaps you speak Korean and we don’t have Korean on there. So what are you gonna do?... The 5 of us that are educators none of us specifically speak another language to the ability that we could teach another language. So that would be a limitation, like we’re not teaching a series of classes in Punjabi or something like that which would probably be really helpful. A lot of the times family members might send their daughter or their son to come to the class and then they take that information home to mom or dad. But something that would be lovely [is] if we could have more translation or ability to reach other populations. But there’s also a cultural thing, not everyone’s gonna come to a class. Not everyone is gonna want that kind of environment (24 leader healthcare late career)  I know we are dropping the ball is supporting patients who don’t speak English or don’t speak English well. As a clinic who’s external to Alberta Health Services, we do not have access to their translation services…. None of our teaching materials are translated into any other languages… So when a patient does [not speak English]; we start right from the beginning asking the patient to bring a family member who can translate for them. So that’s not perfect and frankly, it’s a little bit too much I think responsibility on the patient (30 executive healthcare late career)  ***Women delay seeking care (unclear who to see, time constraints, other commitments)***  Some groups might be more susceptible to knee or hand OA just because of cultural or lifestyle factors... But might not present themselves for treatment because maybe culturally it’s just been accepted that this is just normal aging, you have to live with the pain and things like that (21 policymaker quality-improvement late career).  The policy is that designated physiotherapy clinics in Ontario receive funding for persons over 65 or under 18 for “x” number of physiotherapy sessions. Well there are not necessarily designated clinics geographically dispersed in all communities and therefore there maybe communities at risk that don’t have access without significant transportation…if I am over 65, incidence of OA being higher, and I don’t have private insurance, great! There’s a government funded program but not in my neighbourhood. And the designated physiotherapy clinics are not solely OHIP clinics. They are private clinics that also deliver insured services being OHIP covered physiotherapy…they want to set up their clinics where they can get the mix of private and public funding to make their clinic work and that tends not to be in the areas of highest need or areas where there may be lower priced housing and high-density housing where someone who has lower income, less education, a newcomer to the country might initially be living (15 policymaker quality-improvement late career).  I suspect there might be inequities just in general in how comfortable women feel in speaking up and being heard in medical appointments. There maybe a bit of an authoritative the feeling of less than equal, so I’m afraid to speak up because I’m worried the doctor will think I’m stupid… Maybe that they may not feel as comfortable asking the right, like the questions because of that stigma and the paradigm that has unfortunately existed in the medical world a bit (29 executive charity late career)  If you have to spend 4 hours where there’s no scheduled appointment and just go, show-up and take a turn, the inefficiencies… it’s hard to take care of kids and other people… That a group that we’re calling diverse women will take care of, that’s a barrier as well (22 executive research late career).  Culturally there maybe things on the home front… where they may feel like that they’re dismissed a bit when they have pain and even at home. And then they’re not as readily going to access care as well… Many women are caregivers to either kids and/or parents and/or both and they play that role and therefore it limits their ability to access appointments or get to appointments or put their health first (29 executive charity late career).  I would say we do have some cultures who, the women are the primary caretakers in the home and are reluctant to be rendered helpless for at least a few weeks after surgery… I think there is a toll on women who are the caretakers in their home when they can’t do everything that they previously did… I think that there’s some pressure there (30 executive healthcare late career).  People will congregate or they’ll go and live in communities together but that community may not have [team-based care] there. And as much as you want to provide care close to home it becomes an issue then where women maybe with multiple children are living in these communities without any care there and then if they do need access to care, they go to get on a bus, making it very challenging for them to get there, especially if they’re struggling with the basic social determinants of health, housing and many of them are working during the regular hours. So even to take time off work many don’t have things like sick pay (19 healthcare executive late career).  ***OA dismissed by clinicians due to age, not considered serious, particularly if women do not self-advocate/ask questions***  There could be a stint of stigma… there is the risk of you know just pawning it off on “you just have aging pain” or “its not a big deal” (29 executive charity late career)  When people do go to their healthcare provider because they have an ailment that would look like arthritis; one of the things that we certainly observe is that far too often people say, “oh it’s just arthritis”. That kind of undermining of the severity of the disease or the potential impact of the disease is something that is important for everybody to spend a lot more time acknowledging and there’s research done about when men versus women get access to treatment based on the same kind of symptoms, quite frequently men are getting treated earlier than the women are. So there’s definitely things there that needs to be understood better and need to be overcome to make sure that people are receiving equitable care that they deserve (31 executive charity late career).  Ageism is becoming more and more prominent and its something that I don’t think we’re as in tunely aware of either, like that it’s a problem… if you took a step further and say, diverse women who are of a certain age I think there’s even probably more gaps in equity which is a huge challenge and… thinking of diversity not just the culture and race but with age potentially. I think there could be something there in terms of us being aware of that and looking for future focus that how we can support… older women who have a diverse background, the challenges they might have with access and equity are probably huge (29 executive charity late career).  ***Cost - no health benefits for therapy or cannot afford to take time off work***  Some might not even have access to a family doctor to even talk to them about not having insurance; osteoarthritis can be managed very well through physiotherapy and occupational therapy but one, they might not even know about these services, two, they might not have the funds to pay. So much of it is private now and it’s not part of their health insurance plan or they don’t have extended coverage. So paying out of pocket is just not something they could even think of. It’s just too expensive, the financial barriers (21 policymaker quality-improvement late career).  OA medication now is expensive… $20 a month and $40 a month can make a difference for people as well. And that gets to the issue of national pharma care (22 executive research late career).  Generally speaking when we talk about age I think there are some limitations because physiotherapy is an out of pocket expense… go spend $100 or $150 a session when they don’t know for certain that that’s gonna help. GLA:D is expensive. Sorry, the age thing, is because they don’t have employer benefits. Often they are retired (30 executive healthcare late career).  ***Challenge adopting new or unfamiliar activities such as exercise***  Once you get that diagnosis, it’s knowing what else is out there. You have such a short period of time with your family doctor anyway, they’re gonna tell you, oh well you’ve got arthritis, here’s your x-ray results, take some Tylenol and go do some x-ray or go do some exercise. So the first thing there is if family doctors know about <program name>, then they can say, you can come through this education program, you can learn about different [options]… t and that’s a great thing within <health system name> but other areas don’t have that. So where do those people go? They either go to the arthritis website and they go to maybe some of their classes which are sometimes peer-led versus led by healthcare professionals. So you’re getting a different sort of take on that (24 leader healthcare late career). |
| CLINICIAN-LEVEL | ***Patients may not disclose personal information***  It’s a touchy subject on how to appropriately ask those questions because for researchers to ask “what’s your income” patients are often like “why do you need to know that information?” (02 physiotherapist mid career)  It can be difficult especially as a male. I know some patients, I’m not able to do their injections for example, for modesty and cultural reasons.I’ll have to work with like a woman or another pharmacist to give that injection which is a barrier. So there are barriers sometime in disclosure of information to me because I’m a guy… it would never be directly said to me that I wouldn’t be able to get that. It would just be kind of like an omission thing (26 pharmacist mid career).  ***Ensuring women have a female clinician and/or private space***  Being a male sometimes it would be best if it was a female; [someone] they might be more comfortable with I think it’s often addressed, but in terms of clothing and having dressing rooms so women feel comfortable because [they] might take certain clothing off to show certain parts of their body. Some may not be comfortable with that and especially again, if it’s a male (05 physiotherapist late career).  ***Lack of intersectional training, awareness or support***  The understanding of what somebody might have to go through in their day-to-day life based on their culture is important. It may not be well understood for some people… like the Muslim women who have to pray on her knees. It may not be recognized if they don’t kneel whereas for them kneeling in their culture is quite important with regard to prayer… I don’t know if there’s answer to getting a better understanding of someone’s culture other than just spending more time. So maybe that’s another resource issue, to spend more time with that person to understand a little bit more of their background (05 physiotherapist late career).  I haven’t had the same opportunities for training geared towards other ethnic women groups. It’s been more Indigenous, so that would be interesting and I haven’t been presented with those. I haven’t really come across as many resources (06 occupational therapist mid career).  I think maybe a better understanding of just cultures in general... I feel like I’m sensitive to people’s upbringings and where they’re coming from but at the root of it all, I come from Caucasian background and where I grew up is very much like here in the Yukon which is non-diverse. It’s pretty much been strictly Caucasian… I’m not judging people but sometimes I don’t have the questions that come to mind when you’re asking me earlier about how do you ask them about OA and how it’s affecting them? Sometimes there’s things I don’t quite think about firsthand because I have not been other than university and chiropractic school. I grew up in a very remote area therefore, I didn’t have as much of that exposure, so that just doesn’t come naturally for me (20 chiropractor mid career)  There are a lack of guidelines especially when it comes to diverse women. That’s something that was barely touched on in school or in any sort of continuing education courses I’ve done. I haven’t seen things tailored to diverse women (13 chiropractor early career).  There’s so much information out there and people have asked me questions and I know what the evidence says from a literature perspective but I don’t know all the different questions that people have come and asked me. And some of the treatment options may be culturally based… I’ve had a lot of patients… who have different herbal concoctions… and I’ve never heard of some of the herbs that they’ve talked about. I don’t know to speak to whether or not it’s effective or not effective in terms of pain remedies. I think there is so much diversity with how different people treat pain and some of the stuff I’m just not familiar with and I just think it is a barrier (12 physiotherapist late career).  ***Short appointment time limits ability to develop rapport, and discuss concerns and management***  We’re constrained by space as well as time. So unfortunately the first thing we do with people is weigh them and take their height and then introduce myself and then explain the process... We have a consent that we get them to sign and explain the different parts of [the] assessment which includes the medical history, the joint history (07 physiotherapist late career).  I work in a very consultation-based model of care where I triage patients for surgical intervention and a lot of them do have OA. It is a lot harder to establish rapport and relationships because it’s [typically] a one-time visit. You have to establish a relationship from the beginning…when you greet them in the waiting room, the hi, how are you? Is this your name? Am I pronouncing it right? Who’s here with you today? Nice to meet you. Are you okay with them coming into the appointment with you? Then I may [say] I like the color of your sweater… I only have a minute to establish a relationship with somebody. Are they really going to tell me everything that I’m asking them because I don’t know if they trust me as a healthcare provider or not… I try to talk about non treatment-based things to establish that rapport because I only have a minute for them to either “trust me” or “not trust me” because we’re walking them to the treatment room where I then begin a physical exam or assessment… I usually start the exam with introducing myself, my name, the purpose of the assessment and how I’m here really to help them to understand their condition…but it’s harder to establish rapport in that scenario versus when I was a clinician who assessed and then provided treatment [regularly]… that’s when I really think I established relationships with individuals… I ’m asking them questions about their health. And then there are times where they talk about things other than their health… you establish rapport just by having conversations, one-on-one with the patient related to their injury… that was the best way to have a patient reveal all of the ways that their OA was impacting them through that constant dialogue (12 physiotherapist late career).  There is a lack of time because my appointments are 15-minutes. So there’s only so much that [I can] do (13 chiropractor early career).  Time is a barrier obviously. I only have 10-minute appointments, so you’re trying to explain things to people as easily as they can understand it (17 family physician late career).  I think a lot of it is time in the clinical setting… ultimately the length of your appointment visits are definitely a barrier to having these good comprehensive holistic conversations and assessment (23 nurse practitioner early career).  Sometimes in the rush of time in a clinician visit, the time is stressed and these people don’t have the opportunity to offer or express their concerns with the recommended treatment plan. It needs to be a shared care plan… I’ve worked with clinicians who have taken as much time as it takes to have a conversation with the patient, to answer their questions, to explain to them what’s going on and what’s coming or what could be coming. Some clinicians are good and regardless they will make the time but I would say that’s the minority of clinicians. The clinic that I worked in, after the clinician left the room I was able to stay with the patient and answer further questions from them. If I was answering their questions where they felt more relaxed, less rushed and they said to me, I really can’t afford the medications, I don’t have insurance, then I would go back to the clinician and say, Mrs. So and so can’t afford it. What other options do we have for her? I had the luxury of being there to buffer but most clinics don’t run that way. They don’t have a second healthcare professional there who can do follow up and answer the questions that those people have. And these women who struggle with language and education, they need more time (25 pharmacist mid career).  Because there’s staff storages, and it’s reactive, it’s not proactive medicine right now. A lot of patients I think suffer for that and diverse women especially right?... I think typically women are a little more complex than men when it comes to medications and understanding especially when you bring into like child-bearing risks and teratogenic risk and things like that. And especially in OA, like it's quite a bit more prevalent in women. So how do I spend enough time if I’m always feeling crunched for time to properly assess and make sure that the medications or education they’re getting on their condition is ample and it’s not (26 pharmacist mid career).  OA is not that hard to treat it’s just hard to be consistent in the follow up. I think that is sometimes lost with clinician’s time and ability to follow up and to have all those conversations. It’s less not knowing what you should do as actually taking the time to do it. When you’re seeing people in short episodic encounters and often times with more than one issue, I think taking the time to [ask] what’s your understanding? What are your expectations?... I think that often gets deferred to be you should talk to an OT or [PT]… you often don’t have the time based on the pressures of the area you work in to do as comprehensive of a conversation as you know you should. And I think we aren’t great at looping back and assessing people’s own understanding. Sometimes because [they’ve] known [they] have OA for 5 years but you just assume they understand what it is. Or you assume someone has taken the time (23 nurse practitioner early career). | ***Lack of intersectional training, awareness or support***  There is work needed and probably underway to include cultural appropriateness approaches in the curriculum and institution where healthcare providers are being trained to understand… there are gaps in understanding the specific needs of these different populations and a subset of immigrant women… there is lack of awareness and education on the healthcare provider… a lot is still needed for healthcare providers to be aware how different populations [are] and in this case, immigrant women… Not understanding how immigrant women live different lives and that impacts their physical and mental health (14 government policymaker mid career).  As much as there is definitely an understanding by all providers that they need to do better when it comes to ensuring they’re providing the most culturally sensitive care to everybody walking through the door, it is challenging. You’re dealing with people that have been practicing for a number of years and for them to even understand what it means to provide culturally sensitive care at the provider lens, I don’t think that is well understood. I think the new grads definitely get it. I think some of the non-physicians who have gone through additional training also get it… the leadership that often exists in healthcare are not necessarily reflective of the community that you live in. They don’t understand the lived experience. When somebody’s coming in and has just come from another country and filled with trauma, PTSD, they don’t get that. They’ve never lived it and so they may not come off asking the right questions or do it in a very sensitive way and there is always this, why did the women need to see a female doctor? When there’s all these males available. Those kinds of things are part of the culture (19 healthcare executive late career).  part of it could be language barriers, just culturally not understanding the cultural beliefs of that person and maybe not taking the time to learn about it. Being in a clinic where you’re rushed and you don’t have the time to spend with interpretation, so time could be a factor and spending the additional time it would take to have some interpretation to help show them the proper exercises (21 policymaker quality-improvement late career).  There might be certain things that’s culturally unusual for a group, they may less likely to take it on board… I could suggest an injection to someone and their culture doesn’t go for injections… in their culture they might like massage, and as a clinician you got a range of options and you want to fit that with… the patient choices and shared decision-making. So by not being aware of the cultural impact on patient choices, you’re at a disadvantage, I suppose you can ask, but that’s just the mismatch between my culture and the patient’s culture… [and for] example, Muslim women not feeling comfortable or not being allowed to go to a doctor on their own, that’s actually an issue because of culture… as a clinician you try and link people with other services and in an ideal scenario you know what the services are and who the good practitioners are… you’re completely on top of what needs to be done when you’re in your home territory. And if the person’s from a long way away and then it’s different cultural barriers… you can’t do step, 2, 3, 4 and 5 out of the treatment plan because you don’t know what they are so you just have to be more generic… it can be a lot less prescriptive and then it relies on that person trying to find those [resources] (22 executive research late career).  ***Short appointment time limits ability to develop rapport, and discuss concerns and management***  For patients who get diagnosed with OA they don’t have that ability to have psycho-social support and family doctors are overwhelmed and now even with the closure of the <program name> clinics, their option in their 7-minutes to see the patient is to refer them to surgery and so they go see a surgeon and sit on a waitlist for 2 years to see the surgeon... the only thing we can do is continue to educating GP’s and family doctors to at least provide the education piece to get people something as soon as their diagnosed. It’s not everything, I’m certainly not claiming that, but the numbers for OA are going up to 25% of the population... where do all these people go? What services do they have? That’s why it’s such a pity that the clinics were closed because that was such a great place for people to be able to go. And it was a great service for GP’s because they don’t have the time or the ability to be able to help the person other than say, take some Tylenol and go get some exercise (24 leader healthcare late career).  I’ll say generally it’s very hard to change how surgeons do their consults… I often wonder if they they stick to [talking about] the joint because they only schedule let’s say 15 minutes to meet with a new patient and that’s because we have to right? We have so many people waiting for care, so we can’t schedule hour long appointments… perhaps, [and] this is my assumption, you know you don’t want to open a “can of worms” and start talking about something that’s maybe not within their perceived scope and they don’t think its relevant to their upcoming surgery. So they just don’t go there, that’s my guess (30 executive healthcare late career).  I think the general comment is that our health system is under such stress right now, so to provide quality care that’s in another topic is hard. So it’s really the time people have with their healthcare provider to sort through their issues and understand what’s going on (31 executive charity late career)  One of the challenges that we face as a system is the way that the billing structure is set up. You can get paid the same amount if you are taking care of a well 24-year-old male or very diverse individuals. Our system isn’t set up to look at the complexity of people as they’re coming in. I think that creates a disincentive, we haven’t caught up to the diversity that is Canada and it’s not even the diversity as it pertains to people coming to the countries. It’s also diversity in the number of chronic diseases an individual presents themselves with. I mean morally it’s not right, but why wouldn’t you want a well practice if there really isn’t an incentive for you to take on a more complex [case] where you actually have to put out a bit more effort to go and look for translation services or a bit more effort to find culturally sensitive specialist (19 healthcare executive late career).  ***Clinicians lack knowledge about OA***  The OA tool [developed by the College of Family Physicians of Canada, Arthritis Alliance of Canada, and the Centre of Effective Practice] provides evidence-based patient education…there are many, many programs, on-line programs, funded home physiotherapy, community-based education. The barriers are the provider. If your provider is hooked into the OA tool, the Arthritis Society and the quality standards, there’s a lot there for the patient. If they are not hooked into those 3 key pillars, then their patients won’t get those resources (15 policymaker quality-improvement late career). |
| SYSTEM-LEVEL | ***Long wait times for tests or referrals to specialists***  There are wait times, so things that may happen acutely you can’t see for weeks and then it might get worse and then you’re dealing with something that could have been dealt with [earlier] (17 family physician late career).  As a medical provider there’s lots I can do but ultimately you’re usually are looking at either Allied Health or perhaps referral to specialists and there’s huge waitlists right now. It’s challenging if I refer someone to an orthopaedic surgeon for a really bad hip or knee OA and they’re gonna be seen in 4 to 6 months (23 nurse practitioner early career).  I’ll write [a] recommendation, then you book your family doctor appointment and in your 6 weeks or 3 months and then he’ll write you a req for an x-ray and then you can go get your x-ray, then we’ll book a follow up appointment in 2 months to review your x-ray (26 pharmacist mid career).  ***No coordinated system of interdisciplinary care***  The most important things that we are trying again and again and again is communicating with family doctor and making them aware that sending patients all over the place is not helping. Streamlining things will help much better. Getting the patients to have the education with us is much better than just finding out from surgeries. And to do that we’re trying our best to communicate with family doctors (04 physiotherapist late career).  Most of the patients that come here for OA specific tend to be getting diagnosis from the doctor. Some of them come here out of their own volition, [but] if it’s a referral from a medical doctor I would say that that’s lacking here as far as chiropractic care. They tend to refer more to physio all the time and it think it’s from lack of understanding of what we are providing for care. At least here in the Yukon anyways, there seems to be a lot of reluctance. We’ve been trying to improve that but that’s moving very slowly (20 chiropractor mid career).  And a big barrier for me is documentation by their pharmacists. Like they have all their knowledge about these patients in their heads but it’s not down on paper… I find in community is a big barrier is documentation is poor… with our electronic health record in Alberta we can usually see hospital discharge, consults, consultations with specialists and family doctors. We can usually see that stuff which is really helpful… big barrier is documentation and just patients will be calling pharmacy, they’ll have 12 medications and then I’m looking on their profile and there’s not one condition listed. That’s one thing I’d like to see change moving forward. We’ve done it a little bit with Alberta Health Services is that any as needed medication has to have an indication but even community I think the indication part is really tough because you see mental health medications… you see an anti-inflammatory, what is this for? Is it OA? Is it an adjunct to their RA treatment? … in community we have so many different pharmacies using so many different programs and their notes don’t get uploaded to the electronic health records. So anything like your internal programs is not provincially accessed. I think if there was a system that these notes had to be uploaded or were uploaded to an electronic health records then there might be some accountability (26 pharmacist mid career).  Arthritis care isn’t something that is a standard thing through all of [our] health regions, so that’s a huge problem as well (08 occupational therapist late career).  ***Shortage of healthcare professionals or services prioritized for conditions of greater severity***  Staffing is another issue… if you’re able to switch a male therapist with a woman therapist; is that available or not (05 physiotherapist late career).  We have 1.6 OT’s for a huge geographic region for arthritis and there’s just no way to not grow a wait list or have a wait list (06 occupational therapist mid career).  I guess the government’s making some changes, not around arthritis right now but in general they are looking at funding more spots for OT’s and PT’s because we’re in a place where there’s a lot of shortage across all disciplines. Like not just physicians, the Allied Health as well that would work with that (06 occupational therapist mid career).  Unfortunately there’s not enough staffing and therapies have been cut down to next to nothing. And the roles of therapies are much more towards get this person out of the hospital as opposed to how can we ensure that they… maintain optimal mobility and are loading their joints in ways that are going to support best joint health… one of the biggest issues is that lack of recognition that we are actually taking people into a worse joint health situation… we’re creating so much secondary complications by a healthcare system that does not, anymore, prioritize mobilizing people at the highest level possible and that could just be getting them out of bed with a lift frequently or able to stand and walk (09 physiotherapist late career).  [In Yukon] we don’t have enough family doctor’s and that’s usually the first point of care for a lot of immigrants or established Canadians. I did have a few patients who went to the doctors office and the doctor didn’t have time to explain or tell them what to do or where to go because that wasn’t their doctor, it was their kids doctor who let them in for 5 minutes. I think a barrier is not having that first point of care for referrals or for triaging (16 chiropractor early career).  Our focus has to be on inflammatory and complex OA. We can’t just take a simple early knee and accept them into our program unfortunately just because of our program limitations (08 occupational therapist late career).  ***Therapists have limited scope of practice***  The main limitations that for example, the GLA:D Canada program [patients] could go there but if they have insurance we cannot give them [a] prescription that could be covered by their insurance. I would like to have that ability to write a prescription and the patient could go for exercise prescription and they could go for GLA:D Canada and it will be covered by their insurance. Another thing is orthotics, and I understand that the patient could walk-in and have orthotics but if they have insurance they need a prescription and I need it to be signed by a family doctor. And sometimes the family doctor is not seeing them for the initial assessment… I would like to facilitate things and have the patient able to reach any of the plans that we are offering them (04 physiotherapist late career).  The way nurse practitioners are assisting doctors with medical conditions. I think physiotherapists could also potentially be an MSK assistant for the family doctor but we are all available in Ontario but the ministry will not cover us, the government, Ontario Health will not cover an advanced practice for a physiotherapist (04 physiotherapist late career).  ***Therapist services not publicly-funded***  Unfortunately across Canada, OT and PT for public access has really shifted to mostly post-op and like acute neuro. I’ve been working 15 years and, in my career, we went from being able to see basically anyone for out-patients to very, very limited… Many extended health benefits providers don’t cover occupational therapy in their plans which is crazy. And so, there’s less and less private OT options especially around arthritis, they can only survive privately basically if there’s funding from Ministry to see children or Work Safe because you can’t bill out to Canada Health for your OT services if you’re a private clinician (06 occupational therapist mid career).  We have a list of clinics and I don’t like any of the clinics... It almost seems like a waste of time to say, oh you can go here, you get 5, 4 visits for free just to tick off the box that you’ve got your physio but it’s not real physio… for the OHIP provision it’s 4 or 5 visits. To have enough follow-up to make sure somebody’s on a good routine that they’re gonna carry it out, I don’t think it’s enough visits. But I also really feel that the clinics are just there to grab their 4 visits worth of money and not really be patient-centred (07 physiotherapist late career).  When you look at things like the adjunctive coverage, again, most of these adults are older and don’t have private benefit plans. I think strategically there’s not adequate coverage for things like physiotherapy or occupational therapy other than initial assessments that people have access to and we know that these things are beneficial (23 nurse practitioner early career).  ***Lack of focus and funding for public health campaigns to prevent OA***  At high level there’s not very much medical funding that goes into preventative care as a country, we’re reactive in our medical system (06 occupational therapist mid career).  I think it’s maybe some considered funding... So if you say… we’re gonna put a lot of healthcare dollars into this [program] instead of putting the healthcare dollars into joint replacements, we’re gonna put major healthcare dollars into just educating people about pain and about healthy movement… the problem is you can’t really prove you prevented something… but I know that OA is gonna be so much more expensive than it even is now and it’s a huge part of our aging population that it’s a hard push to do proactive management. Our healthcare is setup to be reactive, not proactive and that’s the problem with OA (08 occupational therapist late career). | ***Long wait times for tests or referrals to specialists***  It could be hard, getting appointments both in primary care particularly for specialist's care… Specialist care appointments are a nightmare in Canada for many specialities and the healthcare system isn’t coping with the demand…that’s not a secret… [it’s] not earth shattering and it’s inarguable and well documented... the healthcare system’s coming apart at the seams and many patients end up not bothering because it’s too difficult and too long… people are on massive long waiting list for knee replacements and joint replacements (22 executive research late career).  ***No coordinatd system of interdisciplinary care***  I’d be lying to say it wasn’t a gap because we don’t have that ability to have that on-going one-to-one follow up. That’s why our nurse started that sleep and stress class because it was coming out more and more. Obviously the huge emotional side of arthritis, the loss of roles, the loss of activity, and then COVID on top of that. It became very evident that it is a real concern. We don’t have a psychologist on our team which is one thing that we would have loved to have had. <Other program name>, they have two social workers and they see patients on a more one-to-one basis. With our education program we don’t have that ability so it is a gap in service... we started doing a program called Step to Success which was small groups, like maximum 6 people, it was done on Zoom and it was supposed to be for people who maybe come to one or two of our classes but needed a little bit more help at goal setting and things like that. But what we discovered when we started running them, was it ended up becoming a really emotional, difficult thing for us a lot of people. There was a lot of tears. There was a lot of people who were really struggling with getting a handle on things with their osteoarthritis and their loss of role and it really was starting to become evident that it was a bit more beyond our scope... we can teach them about stuff but we didn’t have the ability [to] follow them which would be a really wonderful thing to have because I do think it is a real need in the osteoarthritis world. Like people just say, oh you’ve got a bad knee and that’s just it. But if they can’t work, they can’t do their sports, they’ve lost family stuff, it’s a huge thing. And in our scope just as educators, it was getting beyond what we could do. So we actually stopped doing the Steps to Success because it was becoming really evident that we had stepped into something a little bit more than we had expected (24 leader healthcare late career).  if somebody has osteoarthritis, there’s not an OA expert that people get referred to. It’s a little bit different with inflammatory arthritis where you go to a rheumatologist typically. But for OA there’s not that OA expert, so there’s a gap in care where I don’t think anybody really is fully responsible for making sure people living with osteoarthritis get all the care that they need until you are at the point where you need a joint replacement or something where you don’t have any other options… there’s certainly pieces there that I think can be supported whether it’s by a medical doctor, a physiotherapist or advanced trained physio’s or other people (31 executive charity late career).  ***Shortage of healthcare professionals or services prioritized for conditions of greater severity***  You don’t have enough of the providers, it doesn’t matter what distribution channel we have; [Zoom is] helpful but it doesn’t solve the problem of insufficient patient providers. And we know there are insufficient rheumatologists in rural areas and other areas so there’s a geographic barrier and it’s not covered by Zoom because of there being there been insufficient numbers. And then primary care, medical doctors, there not enough of those. So we need more solutions [to] extended healthcare, nurse providers, physician assistants… but those systems aren’t in place (22 executive research late career).  ***Lack of focus and funding for public health campaigns to prevent OA***  As we really think about where OA fits, it’s part of preventative care and preventative care is primary care. And we don’t really have a strong primary care system in this country. It is a very acute centric system (19 healthcare executive late career)  Physiotherapy is a good example which is evidence-based care and because the cost is a barrier… given the evidence strength behind physiotherapy exercise and strengthening exercises for OA and hip… all of that is a massive gap… and I’d argue a gap that should be prioritized given the return on investment if that was done. And that’s for all people and then that’s gonna be more so for marginalized women (22 executive research late career).  ***Lack of diversity among healthcare professionals***  Or are we really stuck in the same patriarchy when it comes to healthcare because it is not a diverse field. It’s slowly changing but it’s not diverse and a lot of individuals that are considered diverse are in low paying jobs in healthcare. But the PSW’s; they are not the ones that are gonna be providing the much-needed care and things like counselling for OA, that’s not where we’re training people up to be (19 healthcare executive late career).  ***Lack of policies or programs specific to women with OA***  When we think of diversity… we think geographically, we think culturally. With arthritis care, yah I don’t know if we think as much other than like some one-off’s, like I said the one webinar on Arthritis Talks, it was specific to women. I’m not into the detail of our content but I do think there’s content that we would deliver that would be geared specifically towards women. But I don’t think there’s often a deliberate thought of diverse women, I don’t think we thought about it necessarily as much that way as we should. So I think a barrier is just a gap in knowledge of this chiasm that might exist (29 executive charity late career).  Most research is done in white wealthy people and men, that’s a fact about research, it’s inarguable, and in that context I’m aware that people are researching, and I’ll speak to funding as well, funding traditionally hasn’t gone to groups such as diverse women and so it’s harder for people to search in that space. So on the other hand, I know that some people are doing excellent work just what those barriers but it’s a small research domain and it needs to be better supported by funding (22 executive research late career). |

Strategies to improve OA care for diverse women

***Clinicians:*** *What strategies would help you (or other clinicians) to provide OA care and advice to diverse women? What could help patients to access, understand and comply with OA first-line treatment and advice?* *What healthcare system strategies or policies are needed to improve access to and person-centred OA care for diverse women?*

***Execs/policy:*** *What strategies are needed to ensure that diverse women access the OA care and advice they need? What infrastructure, resources or approaches are needed to prompt, incentivize, or implement those strategies?*

PATIENT-LEVEL

Offered to persons with OA to improve knowledge, confidence, behaviour, OA symptoms, OA status, or quality of life

| Theme | Clinicians | Executives/Policy-makers |
| --- | --- | --- |
| Education or advice about OA and OA self-care | ***Meetings in community settings***  Group education will be the best because whenever you see other people living with the same condition as you, and each one living at a different level and different perception of the disease, it gives more education [and] insight to do more and to manage their arthritis better… that’s why I like to refer to the Arthritis Society because they have these education programs (04 physiotherapist late career).  ***Meetings in community settings (multiple languages, women-specific)***  Access for OA exercise classes rather than a one-on-one physio would be more economic… people have to be educated to realize the benefit of exercise and then to carry on with the classes once they’ve been taught the benefits of exercise and the education… Maybe to divide it [into] classes of different languages… having early access to fitness program for newer immigrants or people who are just learning that they have OA and don’t speak English or French to be able to get that access… I think getting a group of women together that speak the same language and they’re learning this in the same language, you’re building a comradery at the same time and realizing there’s other people in the same boat as you… [the information] could go to them in the right language; they could take ownership… get the information for themselves and manage it themselves… instead of having their children get [the information] to them (07 physiotherapist late career).  ***Meetings in community settings (multiple languages, Canada-wide)***  What would be wonderful to see is if group classes were offered in every province in different languages and to be small enough that… it can be tailored a little bit but not so small that we don’t service a lot of people at the same time but not so huge that people don’t feel like they’re sitting and listening to recording (08 occupational therapist late career).  Maybe some brochures, factual information that I can provide a patient so they can see it… for me that would be probably the best thing; offering strategies for me to provide better care… there’s a knee program you can do and it’s all in a group session. Being able to have the space to provide that for individuals might be helpful to get diverse women and other people all included in one space and making them see that tons of people have a similar problem. So they can relate to each other. Connecting people with other individuals that have a similar thing; connecting people that are of the same background will be helpful as well because then they can relate even more… [having the sessions] in my clinic would be nice. I don’t really have much space but I think it could potentially work at some point. Because it’s a small space, there’s not tons of people around but then it’s [a] more intimate area and people can actually talk to each other a little bit easier while they exercise. I would prefer that or a small gym space would be good. Just nothing that includes the whole public and everybody, like more than 100 people, I feel like that’s getting a little much (20 chiropractor mid career).  ***Information or resources delivered in healthcare settings***  Brochures… that would have different lifestyle recommendations. Things that they could be doing on their own at home to help manage their OA related to diet and exercise and things like that… are always a good resource to have in the office that you could hand-out or email to them (13 chiropractor early career).  ***Information or resources delivered in healthcare settings (multiple languages)***  When I go to my doctor’s office they have a bunch of posters on the wall and I don’t think I’ve ever seen an osteoarthritis poster. I think that community centres and all kinds of public places could have more information and in different languages… that you could read it and it’s [the] appropriate language that you can understand and learn what this is or ask questions. Where do you go to ask questions? I think having information as important as the poster for herpes or things like that, that are in the doctor’s office (10 occupational therapist late career).  ***Information or resources delivered in community settings (tailored, multiple formats)***  Community exercise programs or diet programs that were accessible and free would probably reduce the burden of osteoarthritis or at least the symptom burden... Geographically you want to make it in multiple centres so that people don’t have to be driving a long way to get there. You also want to make it age appropriate. If you had an exercise class for people with OA who are 45 they probably wouldn’t want to be in a class someone who was 85, so tailoring it to the age group or skill level or intensity of the exercise goals. If people were coming from different cultural or languages, make sure that there’s someone who either speaks that language or can get assistance for them and having videos or pictures to help explain exercises so you’re not trying to follow along after somebody who’s speaking a language you don’t really speak. And making it available on-line so people can do it in the comfort of your own home (17 family physician late career).  Being more available to do education within say a community centre because that’s [what we do with the Indigenous] population, we’re going out; to go to a health fair that might be put on by one of the nations, right? But if we did that also within the Indian population here, find a facility where that might be possible to do some education there. I’m just thinking about that or within the Asian population here as well. So to go to them [in a way] that is culturally safe (18 occupational therapist late career).  Language is important. Getting people resources that are meaningful to them. Some of the OA stuff, for example, is return to sport and some people don’t play sports. So being able to have something that meets the needs of people… what you’re giving a 50-year old with knee arthritis is different than something you give a 80-year old significant arthritis in the hip [offering] different groups of people of similar age group… is there a women’s only groups, older women’s groups… a lot of older women or depending on their background wouldn’t be comfortable going to a big exercise program (23 nurse practitioner early career).  ***Education materials (tailored, multiple formats, community-based)***  Having more materials to hand out for patients to take home with different modifications and accommodations for them…to make things more simple or more tailored to them. Or a website that I can point them to would be good especially one that has different languages. If I could find anymore supervised community-based exercise programs. [In Yukon], we have quite a great Filipino association [with] different sports programs, fundraisers and community events for Filipino Canadians. Maybe if we could get more community-based options for info-sessions or supervised exercise or just a place to put information for the targeted group… based on the age group that I’ve had paper handouts [a piece of paper or booklet with lots of diagrams and pictures] would be best. If there’s younger or more tech savvy people then having a website would be okay… [Information could include] What is their condition? The different types of symptoms that they might be experiencing. The different types of general treatment options or people they can get in touch with to get more information… [exercises] they can do at home, different positions that might be relieving, different stretches… a little bit about progression of disease and percentages so they know [OA] affects a lot of people and they’re not alone (16 chiropractor early career).  ***Education materials (tailored, multiple formats)***  It would be great if there was a program or an exercise program… something they could log into or a YouTube channel that does some exercises for them that’s based visually so we don’t have to worry about language barriers. Or if there’s drop-in stuff that’s provided by the Regional Health Association that does some talks. Something that’s mainly visual so that we don’t have to worry about the language barrier and that’s easy to access, they can access it at midnight or they can access it a 4pm if they have shift work… [and] it would be really handy to have a pamphlet that’s available in a number of different languages that I can download and print off in the office. Or a hand-out that calms their nerves [about their diagnosis] … then give them some general ideas and maybe a couple exercises in the different languages so that they can really understand it… I can access them on a website and [if] I need Hindi or Tagalog, I can print it off right away (03 chiropractor early career).  ***Education materials (lay language, multiple languages)***  Taking information that’s medical and simplifying it... More projects to help with that and getting their information in their language would be ideal… [infographics] are pretty standard these days because it helps cross language barriers with pictures… but it’s not a simple as putting together an infographic, it’s making sure the verbiage is grade 6 reading and understanding level. It requires time and resources and people to put those things together… [it should be disseminated on] the internet. I don’t see any better way really. I don’t think social media would be a good way either because, I just see information on websites (05 physiotherapist late career).  ***Online classes***  More diversity and offerings in terms of language that the classes are offered in and access the classes on-line and that people can just access on their own… We’ve had great reception to the online classes that are offered through our program (08 occupational therapist late career).  ***Education materials (tailored, multiple formats, multiple languages)***  It’s easily available, people can access it wherever they live. We probably need to be in multiple different ways, maybe not always an in-person class. It could be education online or it could be written information. It could be websites, but you would have readily available information and access to it and then depending on whatever language, joint or whatever part of the body, there be resources available for that (10 occupational therapist late career).  A lot of these women don’t have daycare to come to the program. So virtual programs were done during COVID and then people said, I still can’t find the time on that day, so we gave them access to recorded sessions. I think on-line education if they have internet is another good way to reach these people… they do it from their own home on their time schedule, on their timeframe… but I work with a geriatric population and virtual care is next to impossible because they don’t have laptops, and with these immigrant women they probably won’t have access to a laptop either. So whether it’s phone care or something that is close by in the communities that they can go to you need the space for these people can come that is accessible for them. That’s not too far to go and that has flexible hours because maybe they can only come in the evening or on weekends (25 pharmacist mid career).  Patient tools in multiple languages that can least give them an idea of the condition as well as medication, pamphlets in multiple languages that are disease-specific… evidence-based, not expert opinion… good information, just like a clinical practice guideline would… They should be available on one Canada-wide site (26 pharmacist mid career).  If there’s a phone line they can access to discuss with somebody in their own language; have a translation line that will explain it, [like] Telehealth (28 nurse practitioner mid career).  ***Resources or sessions delivered by healthcare providers (multiple settings, formats)***  Most people find about their OA or get a diagnosis from their medical doctor. And what I find is that most of them are like “you’ve got it” and then that’s sort of it. They don’t give them anything else. It would be great if a medical doctor or a nurse practitioner could say, “here’s what it is, here are some things you could do for it. You could go see a physio, you could go see a chiropractor” (03 chiropractor early career).  It’d be nice if the primary care providers would be able to do a tiny bit of that education [about OA] but let their clients understand what that is or direct them to some place… I think it has to be probably multi-prong because patients learn in different ways and their access to technology is really diverse. And so it can’t just be an on-line module that the physician hands out… [or] there are numbers in B.C. that [patients] can connect with a dietician on a phone; like an information [hotline]. Sometimes clients are really comfortable on the phone just calling in and not have to go to another in-person or virtual appointment (06 occupational therapist mid career).  I’m really a firm believer about the GLA:D program to have it accessible in different languages… [or] there’s lots of people out there that are scared… if you try something once and it hurts, don’t give up [and] instead of just telling somebody to go off and do it, having coaching which would come within an exercise program, oh it hurts, well maybe back off the tension and do it for only 5 minutes (07 physiotherapist late career).  If I can Google… “Canadian Orthopaedic exercise booklet” which included stretching and strengthening that would be ideal so I can have something to print off and give to the patient in the appointment because you never know when you’re seeing them again. If that was in multiple languages that would be handy too. And had a website version where you can go and look up videos, examples of it on-line… having something they can reference in their own home to read about helps because, in an attended appointment, going through everything you don’t have the time to really get into the nitty gritty and although there’s follow up, you hope, there may not be (17 family physician late career).  I feel like the medical community could be a little bit better in trying to provide that information of what’s available out there and these are options of care that could be helping you... especially in a small community because there’s two kinds of thoughts here. People are really active and they’ll do whatever it takes to make themselves better because otherwise you can’t do your everyday activities or you want to be doing. And then there’s the people that are just into their doctors. I guess us [as practitioners] being more out there and putting posters up or just showing up at somebody’s workplace and having blurbs of discussion on, hey this is happening, this is common and we can help… having more word out there would be helpful of what’s possible and this is not an ending for you. This just means you got to do something about it… I think best would be if you’re doing something in-person. Webinars could be helpful it’s just whether you’re getting enough people to connect. You have to put the word out there that people know that it’s going on; that’s the thing. Or if you could do a webinar in two different workplaces and then they make their workers attend the webinar, or in-person, going into an office… It [would] be nice to have a counsellor that can come in and maybe hold a little discussion on how to manage pain and how to approach pain on the psychology side of things with OA because pain is not necessarily meaning that you’re making things worse. It’s often just a signal, therefore finding ways to restructure how we think about pain could be helpful. Providing that for patients would be good… then we could maybe touch more people at once whether it’s provided in-person or it’s provided via webinar. I mean webinar would be nice but then not everybody wants to be or can be on-line but at least through webinar you can attend and not show your face if you’re shy about it as well. So it gives people that option (20 chiropractor mid career).  ***Education on joint health***  Osteoarthritis messaging, if it’s done right, is uniform enough that someone who just has a sore knee but doesn’t have osteoarthritis isn’t gonna be harmed by taking a class… we’re not gonna get all these people into a doctor who says, okay yes, you have OA and now you should attend these classes. It’s gonna probably have to be a lot of self-referral, so if you accidentally get someone in who just has a sore knee but doesn’t have OA that what they learn is gonna be… about joint protection and proper movement and pain management [and] healthy movement (08 occupational therapist late career).  There is a stronger push on general education. It’s [an] aging population but it’s also population of people who have also got traumatic arthritis… helping them understand what is acupuncture about; when is it helpful. What does literature say about that kind of stuff (09 physiotherapist late career).  ***Patient toolkit for how to engage in self-care throughout arthritis stages***  Some tools to talk to patients or a toolkit to be like “this is your green zone where you’re good, keep doing what you’re doing. This is your yellow zone where your symptoms are starting to get worse. Here’s when you should see someone, here are things you can start to do at home” ... Something for patients to have a tool… then when someone has those conversations with them you can have a tailored approach to say okay what are we gonna add if your symptoms are getting worse? What’s worked well? Do we need to reengage with whatever type of support you are getting? ... Little action plans that people can bring to whatever provider they see (23 nurse practitioner early career). | ***Information or resources delivered in community settings (tailored, multiple formats, languages)***  There needs to be more work done in developing instruments and tools or revisiting the ones that are currently available to tailor them to different marginalized populations [and] more specifically immigrant women population… Find their champions, if it’s a Muslim immigrant woman newcomer, find champions at the mosque or leaders within their community to understand the needs of those women and where the gaps are and what kind of resources [they need] and then direct them to the resources… for immigrant women there’s some non-profit organizations and then community health boards in areas where there’s more immigrant populations… you can find out where these women are facing challenges or where there can be more education and resources provided (14 government policymaker mid career).  Maybe through community health centres where there might be a larger population of a group of people that might be doing more of a group education type of program where you could reach a larger group because maybe these people aren’t going to see their family doctor about it or maybe they don’t have a family doctor or they’re not going to see a physiotherapist who might say, there’s something we can do about [your knee pain] and there’s exercises you can do to control the progression... I think clinicians who are from a similar cultural background might understand the cultural beliefs and be able to understand; maybe if you’re treating somebody from an Asian background, they might be more open to doing exercises differently. Maybe something like tai-chi or something that isn’t your traditional western medicine but could be helpful with OA. So finding other types of exercises that would be similar to the programs to keep them active or maybe involving their children to help them to get to programs like if there’s programs at community centres or where they could be involved with people from similar cultural backgrounds and feel more comfortable going. Maybe taking some of these clinical programs out of more of a clinical setting and into more of a community setting where you could bring together groups of people from similar cultural backgrounds and they might be more comfortable doing them together in those groups (21 policymaker quality-improvement late career).  More education and information available out in the community. In churches or places of worship or places where they go to socialize, maybe senior centres… even bringing some of these programs like GLA:D into community centres rather than being based out of a hospital or a clinical setting. If there were a way to have them run through the parks and recreation community centres where these people are going for taking their kids to swimming lessons or if they’re going and doing other activities, having these available to them in those community settings or senior centres and moving away from a clinical setting. I know it’s more challenging because you’ve got the equipment, [but] even just functional type of exercise training, you don’t need a lot of equipment (21 policymaker quality-improvement late career).  The Arthritis Society does this where they have patient reps and patient volunteers that come in and spoke to people. So they would be a group I would connect with… [there is] a patient committee that reviews any printed materials to make sure the language is okay, the readability is okay so we’re very cognisant of literacy levels, the simplicity of it to increase access to all different groups... But if you had a patient advocacy group or maybe a focus group to come and have a look... it would be nice to have somewhere you could touch base with and say hey, I’m teaching a new class, it's gonna be about sleep and stress or whatever it is. Do you have any input into this, this is the material we’re planning on teaching. What do you think?... Or if they were able to reach out and say, this has been a real challenge for our group or population would you consider running a class on these topics (24 leader healthcare late career).  I do believe that community-based exercise that’s focused specifically potentially on diverse women may help with compliance adherence. There’s some women that may not feel comfortable exercising at a YMCA with a bunch of men or a bunch of white men, there may be opportunities even for Arthritis Society Canada or other bodies to connect diverse women as peers potentially and build out some programming and supports specifically for that group… It could look like in-person type education in group programming at local YMCA’s and drop-ins or where similar diverse women could exercise and learn together. It could be education groups on Zoom that are specifically tailored to that group… part of it too, is how do we find [them], unless people specifically put this in the referral that they are from a certain background or certainly from a gender point of view, that’s part of the demographic data right? Not everybody is comfortable in sharing some of the other stuff, so how do we link individuals together, so there’s some work to do there I think; building a community almost maybe of awareness but for clinicians and for people (29 executive charity late career).  ***Resources or sessions delivered by healthcare providers (multiple languages)***  I think they should put the clinics back in place [and] have that one-to-one ability to have the OT and PT right as soon as they’re diagnosed. And then for diverse groups then you can bring in a translator so you could have that ability to have a conversation that’s like individual-specific for you and done in a language that you speak and develop that rapport and have an action plan of something you’re gonna work on right from when you’re diagnosed.... clients and patients who do get diagnosed with osteoarthritis right away [should] have somewhere to go. This is not a condition that’s gonna have some magical medical intervention other than the surgical piece but what research shows is that it’s exercise, it’s the weight loss, it’s using your joints... that’s the stuff that is gonna improve their health, improve their ability to stay independent, reduce healthcare costs because they’re not coming in for surgery to begin with because they’ve been able to do these things to manage it. So giving patients access to that early education and especially if you can have that one-to-one piece would be so ideal... you have more of that one-to-one ability to have that time to really develop that rapport, have that person you can come back to again. Have someone you can connect with on that more personal level (24 leader healthcare late career).  ***Education materials (multiple languages)***  There definitely needs to be consideration for offering education materials in other languages… it wouldn’t be an easy endeavour to translate everything but certainly that just requires time and expertise (30 executive healthcare late career). |
| Regular follow up from doctors to monitor OA condition and self-care | Repetition, doing phone follow up [from occupational therapists] (18 occupational therapist late career).  Being able to hear about OA and talk to different providers about it would be helpful. Often when you see your nurse practitioner or physician again, it’s the first point of diagnosis and you don’t really know what to ask yet… [we need] either nurses or someone else that can ask questions or intentionally do a meaningful follow up is important to like probe, to be like how are things going? What’s working well? Do you have questions? As opposed to leaving it all in the hands of patients to follow up. (23 nurse practitioner early career). | -- |
| Consider patients’ cultural needs and economic circumstances when offering treatment, or self-care advice and/or programs | Treatment options need to be more culturally sensitive. I do know that aquafit is one of our biggest recommendations with OA. But I don’t necessarily think aquafit is culturally sensitive… because we’re trained in a North American site, we don’t know what exercises are done elsewhere that are just as beneficial for OA. We’ve adapted things like tai-chi and yoga from other cultures. I feel like there must be other forms of exercise like dance or something that would be equally beneficial and that diverse women might embrace more (12 physiotherapist late career).  There should be a question if they’re under 65, do you have insurance for medications? If they say no then that certainly should be a red flag on the chart for the clinician. That whatever you prescribe is going to be expensive and possibly a barrier… maybe you’re gonna go for a generic [brand] that’s going to be cheaper… physiotherapy is another one. Physiotherapy is often recommended as treatment but a lot of people don’t have the money to pay. So don’t send someone off for physiotherapy to see if it’ll help their shoulder if they can’t afford it. Some of the management strategies for osteoarthritis come with a cost that these immigrant women won’t be able to afford and that needs to be taken into consideration (25 pharmacist mid career). | -- |

CLINICIAN-LEVEL

Offered to healthcare professionals to improve knowledge, confidence, behaviour, or how they provide OA care (e.g., skills)

| Theme | Clinicians | Executives/policy-makers |
| --- | --- | --- |
| Medical or continuing education on diagnosing and managing persons with OA | ***Deliver to healthcare providers***  Family doctors should be informed more about the treatment of osteoarthritis. Lots of family doctors are sending [patients] left and right, sometimes a 20-year-old with meniscus tear, he’s not an arthritis patient (04 physiotherapist late career).  Education, at least of family doctors or nurse practitioners, [is] becoming more and more common even anyone in healthcare who is their first-line person seeing these individuals; lots of education about early signs, what to look for, what to expect, when to refer, what is out there in terms of programming (08 occupational therapist late career).  I wonder if physicians… and physiotherapists who are often the frontline of health promotion and prevention should have some of their focus re-looked at... Prevention starts with alignment and posture and muscle balances and all of those areas… amongst the professionals themselves perhaps within their own professional practices identifying maybe a bit more their knowledge and expertise in those areas… if there was more of a provincial strategy and a regional strategy and regional education support that goes a long way to engaging clinicians in a world where they’re struggling because there’s not enough of them and people are constantly trying to pull them all in 12 different directions (09 physiotherapist late career).  Maybe some sort of continuing education. I think webinars are a good way to go especially now. It seems like a lot of the continuing education that I’m doing is virtual these days (13 chiropractor early career).  Other providers are better at [OA treatment] conversations than some medical providers are, if you look at physio and OT and people that are in the home or look at more a functional application for people. For nursing too, having some training around how do you talk about OA? What are the expectations? Trying to implement that this is chronic and we should follow it up because it often gets dismissed (23 nurse practitioner early career).  ***Deliver through professional organizations and/or associations or conferences (multiple formats)***  Get the information out… through a professional organization such as the Canadian Physiotherapy Association because those are the ones that we’re all often going back to, so that’s a good place for us to find out if these things are happening… The most efficient and easiest would be webinars… I think a virtual format, something over the internet would be probably be best because it’s sometimes difficult to allocate time and resources to going to certain conferences, or if somehow the resources existed to have people come into the facilities and deliver it over a lunch break (05 physiotherapist late career).  Our professional associations are there to educate us on these things; going through the Health Authorities… But as for the private practice physio you’d have to probably target the professional organization or as the head physio working in the hospital (08 occupational therapist late career).  I think the colleges and the professional association certainly could do more in those areas [of cultural sensitivity] to provide leadership and support and education particularly Ontario Physiotherapy Association. They do some but nothing specific (09 physiotherapist late career).  It’s always good to have access to education funding and time to be able to attend that because things in OA care do change and evolve and even currently as an OA site we’re a little outdated in some of our actual approaches and practices (06 occupational therapist mid career).  I personally try to keep abreast with information that comes from a number of different sources for example, there’s the Arthritis Consumers Expert Group, the Arthritis Society. I have co-workers who attend various different education conferences and they share updates on osteoarthritis that have come out… information comes from a number of different sources and discussion with co-workers; it comes up within our team that a client has come in and these are the things that have been really helpful or presented… there’s lots of good information out there and it’s just a matter of we have to spend time to look at it and discuss it and often we’re updating our resources in our own patient and client services and hand-outs (10 occupational therapist late career).  Ultimately people go the big websites for information. When you look at big orthopaedics releases and resources when you look up stuff for patients that’s where you find them… having published information and resources that people can use are helpful. In terms of reaching the most diversity of providers, some of the big conferences are a good way to go about it as well as the adjunctive providers, like your physio’s, your OT’s, the ones that go back to their teams, say okay, I just did this really interesting talk and can actually relay a bunch of it back to our team or think of an idea in my localized clinic setting that we could start to trial a different approach (23 nurse practitioner early career).  At the clinic I was at we would have guest speakers come and talk about a program or screening or new approaches at conferences. There’s a lot of talk of the diseases we all know but what’s new and what can we do better… language and appreciating the burden of something like OA specifically in women and diverse women and maybe how their care can be more easily missed. Sharing some lived experiences of people could be helpful. Not to demonstrate ways in which the healthcare system has necessarily failed them but to demonstrate barriers to access… presentations or even things that don’t have to be brand new approaches or new meds or totally new therapeutic. There’s a lot of talks at presentations right now and primary care forums on how do we do better at stuff we see every day and that is a big burden on the healthcare system (23 nurse practitioner early career).  ***Educate family physicians on role of chiropractic care in OA management***  Sometimes we have some little conferences that are offered here especially with workers comp. So trying to communicate with the doctors here and explain what our approach is to care and not just by manipulation because that seems to be the current understanding. Number two, trying to find out where they’re coming from. We’ve had some people strictly say to patients don’t go see a chiropractor. So that’s been unfortunate but I mean that probably happens in any profession. And just sending patients out with letters to their doctors for keeping information current on a patient and what we’re doing as far as care goes. And explaining in that what care we’re providing so that they know that it’s not just a particular solid treatment. And sometimes there’s a phone conversation but that’s not so frequent (20 chiropractor mid career). | ***Deliver through professional organizations and/or associations***  It’s really important to reach the regulators of each profession… like the College of Physicians and Surgeons... A lot of practices are multidisciplinary, interdisciplinary collaborative practices and it’s not just the physicians not providing care… it’s a multidisciplinary environment, increasingly so, and the regulators need to be aware that that should be part of the licensing requirements or their on-going continuing medical education. Starting from curriculum development all the way to training current providers and on-going training and some sort of performance management system where they demonstrate that understanding and they’re applying it (14 government policymaker mid career).  ***Implement accreditation standards for culturally competent OA care***  It should be part of the accreditation standards and licensing board exams and for professionals (14 government policymaker mid career).  ***Inrease training about OA diagnosis and management in medical school curriculum***  Down to the root, when you look at medical training and things like that, and to try to understand how much clinicians actually get training on for example, osteoarthritis care. I think there’s huge opportunities there to make sure that clinicians are much better prepared to deal with Canada’s most common chronic health condition (31 executive charity late career). |
| Education or training on providing person-centred OA care tailored for diverse persons | ***Training about different cultures for practicing healthcare providers (multiple formats)***  We’ve done some implicit bias testing… to increase your awareness and we are working with the equity groups to get little [training] workshops that you can do for staff to increase awareness (02 physiotherapist mid career).  Providing information to healthcare workers about what certain people might be going through that we as other people not in their shoes may not be aware of. So, I guess the strategy would be to research that information and get it out to people like myself in different ways and then provide them with possible resources… Providing strategies to the professionals… of how they can address certain people differently or certain cultures differently or certain or women differently if required (05 physiotherapist late career).  It’d be nice to have expanded training on other ethnic diversities because I haven’t had access to that… the more courses I’ve taken on learning cultural competency… the more you actually practice and then apply it with your clients and not being fearful, that helped a lot. And the role playing always feels a little silly but it actually is valuable (06 occupational therapist mid career).  I think of clinicians being educated and it’s hard. Most of us have experience with certain cultures but it’s impossible to know every culture. But maybe it’s not as much cultural sensitivity training but just cultural training about different areas of the world and different… cultural or belief or approaches to different things (07 physiotherapist late career).  I think sometimes it's even just [having] awareness, like that diverse women may not even be expressing to you their true needs and concerns. I think cultural sensitivity training would definitely be a start because then you’d have time to maybe reflect on how you can do things differently (12 physiotherapist late career).  Having resources and having courses that talk about culture and diversity just helps to bring light to certain things and I’m like, “oh I got to note that”. And make sure I ask those specific questions because unless you ask those specific questions, sometimes the patients not gonna tell you anything about it. Not everybody is forthcoming with that information... Ideally for me, which I don’t even know if it even exists, it would be like whenever you’re going to a seminar about anything… they often do like a case report, or like talk about a case, so going through cases of where people are feeling challenged or had trouble receiving care… going on a case-by-case basis and going through examples, I think that would be helpful for me because I seem to relate to that a lot more. And probably even just a webinar would be fine, just talking about common things that happen in certain cultures (20 chiropractor mid career).  I would like to learn some strategies on handling a language barrier or when dealing with an interpreter or a family member whose interpreting for the client. I feel like that’s something I didn’t really get in school and I have experienced it throughout practice (13 chiropractor early career).  it’s good for everyone to understand diverse women [should] receive OA [care] in different ways right? Based on their own health... Ensuring that family physicians understand because they are usually the gatekeepers right? And they’re point of entry into the system. So that they understand what is it? What they’re looking for? And the patients will be assessed by their family physician and ensure that when they’re referred they will be seen in a timely manner [and] listened to... Continuing education that can be offered to them… things like MedBrief, conferences, talks... if it's talks, organized sessions when it's convenient for a group of physicians who can attend at a convenient time, to discuss it, at conferences... Is there a website they can go to to access and then read up on that and maybe see they need assessment (28 nurse practitioner mid career). | ***Training about different cultures for practicing healthcare providers (multiple formats)***  The Nova Scotia Health Authority and the IWK… are developing frameworks and policies to be more mindful of raising awareness, educating the staff and people who come to access care to understand their needs better and provide the care from a very culturally appropriate place (14 government policymaker mid career).  Through continued education, learning that [clinicians] understand the cultural norm [and] have better cultural sensitivity to the different people and the populations that they’re treating. And maybe through bringing in people with lived experience to share their experiences and have them understand how they felt when they haven’t been heard or when they haven’t been respected in that way. Some sort of cultural sensitivity training would be helpful. I think if they work in a setting where there’s a larger population of [a specific culture] even more intensive type of learning about that culture and understanding it would improve the relationship with their community as well. Just increase awareness of the cultural factors from different backgrounds and understanding them better... there’s some great on-line [training] that are interactive as well... a lot of those that are free, offered on-line or an organization could subscribe to a program and do it as a group. Like a family health team or a group of family physicians they commit to cultural sensitivity and safety learning as a professional development goal and set aside time that they can work through those either on-line or through some professional development, if they were able to do something in-person as well (21 policymaker quality-improvement late career).  There probably are training opportunities. The majority of our surgeons are middle-aged… [and] a good portion of our surgeons are middle-aged white men. So whether or not they’ve been exposed to training related to diversity and diverse women to the extent required to actually have fulsome conversations with them about how their cultural background maybe affecting their care, I don’t know if they’re equipped to have those conversations and thus, don’t have them… like DEI training [in general] which is probably available through various administrative bodies. But probably also, there needs to be a leadership expectation set forth that was addressed as part of standard practice (30 executive healthcare late career).  ***Include training about different cultures in medical school curriculum***  [Cultural sensitivity training] is one of the [medical school] electives for example, in a lot of institutions that put the onus on the student to take. It should be a mandatory training in cultural competency to understand that everyone’s not the same, that people need to ask specific questions for specific populations in order to understand their needs. To have that open mind and then give them certain tools, certain practical one-on-one communication exercises (14 government policymaker mid career).  Getting ahead of the curb… I haven’t been in school in awhile, but in medical schools and then health science schools like physiotherapy, occupational therapy, social work, nursing, could there be a focus on women and diverse women in particular in terms of raising awareness at that level, getting that early [education] (29 executive charity late career)  ***Collaborate with representatives of diverse groups***  Maybe if we had more connections with perhaps different diverse representative groups that we could connect with and say, are we missing things? Are there needs that haven’t been addressed? Are there cultural things we should be thinking about? That would help clinicians. We can look at the research and say, the research says do this or do that but maybe that’s not culturally appropriate. So maybe connecting with different cultural groups... I don’t have a connection with like a Punjabi group who says, oh we really need to consider this or I don’t have a Farsi cultural centre that I call and say, hey what should I know about osteoarthritis or for OA patients? (24 leader healthcare late career). |
| Access to interpreters | The financial resources to acquire a translator or translator help… in an ideal world the translator would be in the medical field… if there is access to translators or free translators or paid translators; how do we find these translators (05 physiotherapist late career).  I would like to have access to interpreters… I know they have American Sign Language interpreters that you can access that but I haven’t really found [help] for people who may not speak English… we have a lot of people who have immigrated from the Philippines, from India… I don’t really know anyone who speaks those languages. So I usually try and get the family member to come in and help interpret (16 chiropractor early career).  Maybe one of the things that will improve the barriers is ensur[ing] that there’s a language translation line because it’s important for people to understand the care you want to provide... [that] the person understands what you’re talking about and you explain the medications they are on, the side effects, options that they have (28 nurse practitioner mid career) | ***Training or funding for medical interpreters***  If there is a language barrier, [there should be] some sort of interpretation services available to support the clinician and the patient so that they can communicate... all the information materials probably can’t be translated. But if you’re working in an area where you have a predominant group of population, then looking to have translation done to some of your education materials so [patients] can be given translated [materials] so there isn’t just English available… we do understand the financial barriers of translating every language. if it is a language that is not common then it may have to be just through interpretation… But if you’re working with a very diverse population, maybe looking at some sort of an investment of it having translation of the education materials for those languages… in hospitals you can call interpretation services that realistically in a smaller physio clinic or a family practice office is probably unfortunately not something that would be available to them. So they probably do have to rely on family members or staff... hiring other staff that speak a more diverse staff as well could maybe help that too… it shouldn’t just be expected that it would be a family member (21 policymaker quality-improvement late career). |
| Assessment tools | When you have heart disease [there’s a] statin decision-making tool… these lovely tools that are clinical decision aids and you can plug in the patient’s information, their age, the duration of symptoms and then it’ll show you… with and without [intervention], what are the differences in outcomes… [we need] decision-making tools to say what’s your threshold if we’re referring to ortho, recommended initial assessment and follow up in 4 to 6 weeks or whatever, assess for falls risk if it’s a hip or a knee arthritis. All of us like things that are easy to follow and help provide consistent care. [We need] more of a dedicated strategy even if it was an extension of osteoporosis but for management in Ontario (23 nurse practitioner early career).  I would like some sort of standardized questionnaire… similar to a pain questionnaire or a neck disability index, that’s an assessment tool… I would have it almost pre-built as a template that the patient would fill out before they came in that would ask them about their OA and their condition. And it could help me further understand more about that patient. The questionnaire might [include questions such as] how long they’ve had the diagnosis of OA, if they’ve had any previous treatment of OA and things that they’re currently doing to help themselves with their diagnosis (13 chiropractor early career). | -- |

SYSTEM-LEVEL

Developed and/or offered by health systems or government to improve access to OA care, advice and support

| Theme | Clinicians | Executives/Policy-makers |
| --- | --- | --- |
| Public health campaign to raise awareness of how to prevent and manage OA | We have an aging population coming up... Giving an education for the public with better information on what osteoarthritis is and who can provide that first line care. So be it a chiro, be it weight management, different options out there for them... Having something right at the doctor’s office would be very important… like a pamphlet of some sort. Although once again with diverse women are you running into a language barrier, probably yes. So that makes it a little bit more difficult. I’m wondering if there’s a campaign that could be run maybe some internet ads… you’d have to figure out where the diverse women are involved in the community. Is it something that should be at kid’s schools? Are they involved in a community centre or their local churches? (03 chiropractor early career).  Having things in clinic like posters or hand-outs goes a long way even to get people to recognize that their symptoms might be [OA] and that this is something you should talk to your doctor about and there’s things that can help… if something’s up on the wall and it’s affecting the person sitting there, they’re more likely to mention it… where you have like poster or an image that’s specific for that population (17 family physician late career).  More educational campaigns or awareness for different types of osteoarthritis in the communities and places and target [certain groups] so they can find this information…there’s one big community centre here and they have posters and advertisements for different types of chronic conditions including osteoarthritis… [include] advertisements [e.g. informational posters, events or info-sessions] on public transit or places where there’s a lot of people such as grocery stores. And even radio, I know a lot of people listen to radio here… [give people] concrete direction… and say if you want to learn more about how to help yourself, then you can contact this organization or go to this place and learn more about it. That would probably be more helpful than just talk to your family doctor (16 chiropractor early career).  We need a lot of positivity in our messaging… Arthritis Society or certain web-bases are trying to share success stories and I like that to be spreading wider because OA, it really affects so many of us… encourage people to take small steps and knowing that small steps can make a difference. I think that is a very powerful message. So it’s actually aligns if we talk about losing 5, 10 % of weight can help keep your diabetes controlled… the message I’m sending to my patients is probably one of your best pain relievers if you [lose weight]…. small steps would really be motivating as opposed to this is just impossible to get done… the message of one side of OA is yes, it’s gonna progress but the other side, focusing more on function in the background of progressing OA, one can continue or even be able to do certain things with optimal management… it could be pain medication, could be adaptable devices, could be healthy weight (27 pharmacist late career).  [OA education] classes also have to be somewhat consistent across the board where if you live in B.C. you’re not getting a drastically different message than someone who lives in Ontario… more proactive management, more continuity between all the provinces… more diversity in our offering… right now there is very limited resources and we’re limited in what we can do and who can reach… I don’t know enough about healthcare politics, I just know that it does vary so drastically province to province but OA is more of a Canada wide issue (08 occupational therapist late career).  Unfortunately a lot of times we see clients that have so far progressed with osteoarthritis; often we hear, I can’t believe I wasn’t sent here 12 years ago, I could have made all these changes and protected my joints… [patients need] early diagnosis and early intervention so they can have a higher quality of life and not have it progress to a point where they’re trying to catch up and struggle or facing surgery… There’s been lots of good studies that talk about [how] doing exercise for example, to hips and knee; prevents people from needing surgery. So there’s already research being done it’s just a matter of putting money into [exercise] as opposed to surgery… And how inexpensive the GLA:D program is but… the people who we’re spending a lot of money on probably could have done better if… they were treated in a conservative way (06 occupational therapist mid career).  We need to be a little more on the proactive side where we’re putting more money into health prevention or health promotion because if people can learn about some of their management of the OA or understand it or recognize the signs of it early and do things differently, they would maybe minimize the impact of it (10 occupational therapist late career).  Osteoarthritis is wear and tear of the joints and it’s preventable. I like to start with patients who are children and maintain them. You correct any problems then you maintain it. [Chiropractors focus on] maintenance and prevention but there isn’t any of that in the medical care system… in terms of the body itself, the musculoskeletal system I think we have to take care of that from an early age (11 chiropractor late career).  If we have policies that really encourage all of us pay attention and working on healthy weight, that will be help a lot of OA symptoms… it’s got to be built early on and accessibility on that and just making that a priority in exercising. Just like when school had the amount of time [young children] need to do physical activity. It would be very interesting if this is implemented in older adults in groups, in workplaces, it will take decades to change but when that happens I think it will do us good… just like it’s part of like brushing our teeth, to have some kind of active time and how we manage the healthy eating… we have to make that accessible, that’s the thing. Right now, it’s more like the healthier approach to meals are much harder to access than less healthy food… I’ve seen elementary school trying having fruit given to all students on one day. It has to be a pattern, it’s not every single day but things like that, makes a lot of sense because then it’s engrained [in] us (27 pharmacist late career). | The most important thing is that people understand that osteoarthritis is not just a normal part of aging, so that awareness piece so that people can figure out that there is something I can do about it and I don’t have to live with pain for the rest of my life. There are things that I can do to manage the pain and maybe slow progression... maybe like in a senior centre if they have TV’s or videos or doing social media too, with a little bit younger population... maybe reaching them through other types of social media like Instagram and Twitter and TikTok, Facebook, some short videos (21 policymaker quality-improvement late career).  I think a public education campaign… we know public education helps in certain awareness… I’ll see bladder cancer ads on <street name… [on] the national level what are the priorities that we should be educating, given the burden of osteoarthritis and the power and the evidence in favour of exercise … I wonder [if] ParticipACTION could be involved… [if] there’s a committee people could say, why don’t we try and get a clear message that early osteoarthritis doesn’t equal knee replacement which many people do believe, and trying to change the thinking… I think that’s an education program that would be a solution… We know there have been instances in public marketing campaigns… there are these success stories… There could be some broad public mass media to support but ultimately, you’ve got to have a targeted audience and segment your market… look within OA, what are we trying to fix and have it at 3 levels, the high risk level where these are the people at a massively high risk of getting osteoarthritis… [deciding] where you’re return investments gonna be, like we could reduce by 1% that’ll make a big difference… [the middle risk is] your sports injuries and people with underlying conditions that are predisposed to osteoarthritis… The low risk is joint general education… delaying the risk of the osteoarthritis by one or two years would be amazing realistically... you got to go and listen to a community and see what they think the key messages are. You need the evidence to go, what are you trying to change; what behaviour you’re trying to change. And then you need to keep sampling it and do tests, evaluation, are we on the right track… follow the principles of effective campaigns and then see what works and then you can go harder on those and scale it up but it’s hard in politics because of the four-year cycle and also the different levels of government… in health it should be across government to support each other so we don’t have any inefficiency. It needs to be targeted locally (22 executive research late career).  We’ve got to get behind the Global Action Plan to Physical Activity... Because that will affect all these other diseases as well and so the argument is that’s gonna be cost-effective… we [need] the infrastructure [like] cycle paths and walking paths and these things and they come back to wealth because… people living in the most impoverished [communities]… you’re kidding yourself if you’re going to go and put a bike path through there… the wealth of the country and the social determinants [are] part of that… you move everyone a little bit from at the levels you can… do what you can where you can… They say community is the biggest determinant of health outcomes… if communities more equitable… if we improve that that’s gonna help diverse women… if the nation was more equitable and there was less poverty… that gets back to prevention because if people have got access to proper food and education advice, basically which we know is the driver of osteoarthritis... then we move on from there to early symptoms and clinical care (22 executive research late career).  The first step is for people to know what kind of resources [are available], educate them, provide them with resources where they can attend sessions and learn about osteoarthritis and how they can prevent it from progressing or deteriorating… move [to] a more upstream approach of health promotion prevention, education (14 government policymaker mid career).  If the government can figure out that by preventing people from going onto to having serious arthritis and not requiring joint replacements is cheaper in the end. So investment in that early intervention, prevention, things like that can save money. So looking at funding mechanisms to support the upstream type of [intervention] so that people don’t end up having joint replacements (21 policymaker quality-improvement late career).  Unfortunately [health promotion] is not something that people want to put money into. It’s the same thing with the clinics and that I think is a real sad part because you have to put that money into these early interventions to help people early on to avoid those long term costs like length of stay and number of surgeries. But if we can invest as a country, as a population and invest in this early intervention it may not seem as exciting, but from a class perspective I think a long term investment of doing that early has a huge savings for all of us as a society, as a population and I just really hope that money and support is given to that early intervention rather than just focusing on that surgical piece because it is such a small percentage of the people that have osteoarthritis go on to get a joint replacement, yet there’s so many people living in pain and with limited ability to do daily activities. If they were able to have access to education and things that they could do or making programs like the GLA:D program which is a fantastic program yet it’s very expensive. So it’s a barrier for some people who financially can’t afford it... I know there’s been talk about making that... a free program and that would be amazing or a reduced cost so that it was accessible to people (24 leader healthcare late career).  There should be the largest focus on health promotion prevention and education. There isn’t much on that really. It’s already too late when people find out; this can be a more upstream approach for people to understand and know what osteoarthritis is and how they can care for themselves from a gender population perspective. Then more specific, like immigrant women have a different way of living and caring for families, so also help supporting, providing resources to care for themselves and know where they can access the resources (14 government policymaker mid career). |
| Self-referral to therapy (for those without family physician) | I wonder if the concept of self-referral to a clinic or to an educational program would be an option to help improve accessibility because if they don’t have a family doctor [and] we need a physician referral for this program, that’s a significant barrier… there would be a series of questionnaires or some way of determining are they exhibiting symptoms of osteoarthritis, for the person who’s triaging the referral on the other end (25 pharmacist mid career). | There probably needs to be mechanism to access care without a primary care provider especially now when they’re so difficult to meet with (30 executive healthcare late career). |
| Expand scope of practice of therapists, pharmacists | We do a really extensive comprehensive assessment. With some training and with presence of some more medical directives, we could streamline the patient into the right channel much better than what we are doing now… like blood work, for example, all my patients who present to me with some inflammatory presentations; if I’m able to send them for tests and the family doctor just sends them to the rheumatologist… if the patient’s coming with the full exam ready for the rheumatologist to decide, [they] could start treatment rather than postponing treatment (04 physiotherapist late career).  I think it would be more economical to run [a clinic] with a physio or an OT than to have a nurse practitioner because the physio and the OT that’s their specialty area… they are the kinds of people who work for the Rapid Access Clinic… And from that model there could always be the option to talk to a dietician or a pharmacist if they want to talk about medication options (25 pharmacist mid career).  There has to be some other ways like a pharmacist referral to a PT; can that be covered by the health system? Right, because why does it have to go through a physician? … I’m not that familiar with the criteria that allows for things to be covered by the healthcare system because I don’t refer because I’m not allowed to. I can say go book a private appointment but I can’t say here’s my consult and now since I’m referring you, you’ll have coverage… nurse practitioners… they can refer and in Alberta, we have a really expanded scope of practice so we can prescribe, we do the injections. I can write labs and then they’re covered by the healthcare system. I can write lab orders which can be really helpful. I think the referral process would be great especially even pharmacists working for a rheumatologist, I mean they’re already at a rheumatologist but can [the pharmacist] write the order to refer to physio? Does it have to be the rheumatologist? (26 pharmacist mid career). | Our team members, they have expertise in special arthritis care. We recognize that, we don’t want to come up with a cookie cutter standards of care where everyone that comes in with OA is treated in the exactly the same way. Some people might come in for one visit and that’s sufficient and our therapist can then discharge them. Others might need 4 or 5 visits or be connected in different ways. I guess we just give our therapist the autonomy to see who’s in front of them and if it is somebody who requires an extra visit or requires something that’s not part of necessarily a standard protocol that we do offer them the ability to do that (29 executive charity late career). |
| Publicly fund therapists | Having better health coverage for the people who are gonna work directly with the OA. Like myself, physio, even nutritionists to help with weight management; possible group exercises, something like a wellness centre. The funding for that would be helpful. A lot of the diverse women that I deal with are either recent immigrants or from lower socio-economic status and [not] having to worry about the financial burden of dealing with their health would make a big difference on them actually continuing with their treatment plans (03 chiropractor early career).  Physio that’s accessible through OHIP… whoever’s making that decision as to “this is where you should go to get good physio advice and good exercise advice” needs to be looked at more from a clinician’s standpoint down at the level where we see what goes on and what’s good and what’s bad and not from a, “oh these people are giving us the cheaper rate and they’ll do it” (07 physiotherapist late career).  I’d like to see if we can improve the access to topical NSAIDs because right now, it’s quite expensive, so a lot of people are not able to access it (27 pharmacist late career). | When I think about income level there absolutely needs to be some consideration on how low income or people without benefits can access physiotherapy and other interventions (30 executive healthcare late career).    People will have different access to various supports. So trying to make sure we get that high quality care to everybody? (31 executive charity late career) |
| Dedicated inter-professional OA clinics | The Rapid Access Clinics are available in every region in Ontario… any patient who presents through emergency with osteoarthritis of hip and knee could be redirected through to us through e-referral, not even printing any papers, just redirect them right away to us… around 47% of this patient population [does not want] surgery. These 47% of referrals going to the surgeon’s offices… [it’s] a waste of time at the surgeon’s offices meanwhile they could have been in the RAC and got all the education they need to manage their osteoarthritis symptoms… if an advanced-practice physiotherapist is at the clinic with a family doctor, they will weed out everything and get this patient to the right location (04 physiotherapist late career).  Our health care system is not set-up to be very accessible [for] clients on low income with no vehicle and limited access to public transportation… they’re faced with having appointments at all these different places… could care be provided in more of a central, a one-stop? Where you go and see your doctor and then see the psychologist if you’re having concerns and see the OT, PT or the social worker, the surgeon (06 occupational therapist mid career).  [If] there was something in the community, like some specific osteoarthritis centre or person, maybe they work with orthopaedics or family medicine, as some sort of liaison to try to coordinate, like a care coordinator. Or even nurse practitioners being utilized more and more these days because they spend more time with their patients in each individual appointment. So having specific osteoarthritis nurse practitioners (17 family physician late career).  Just having more resources. It’s very hard for people with OA, right? We’re here in <city name> but the resources are very limited overall unless they’re gonna go to a private clinic let’s say for physiotherapy. Or we have a hand clinic here but it’s costly. They have to pay for the sessions, plus pay for splints. There’s not the same time given as what we give in terms of education. So having more resources (18 occupational therapist late career).  I think having more access in the hospital possibly would be helpful. And then trying to make some kind of multidisciplinary clinic I think with counsellors or different practitioners.... If there could be some access to those practitioners at the hospital or even here especially even in a multidisciplinary office, we don’t have that. Then it looks like doctors and physio’s and other practitioners are working together. It makes it just more visible that way. Makes it easier for communication between practitioners and then for patients and for practitioners to discuss a patient and then you could probably all sit down a little bit easier with a patient if they needed a more general approach (20 chiropractor mid career).  We need more services, more clinics so that we can better manage it before it progresses to a point where they do need a joint replacement… I think it’s the access to specialists, whether it’s a physician or an allied healthcare professional who can educate these people about what’s going on and different strategies for managing their pain and their symptoms. If doctors had access to an on-line referral service for a virtual program or a clinic in their locality, they need more access to speciality care clinics… specialty clinics are very successful. In Ontario we have the Rapid Access Clinic for people who want or are considering joint replacement surgery and we ran those out of our clinic, people were referred and triaged by an occupational therapist or a physiotherapist with special training. It was determined then and there whether or not they were surgical candidates. Having dedicated space and for osteoarthritis and having it close to [care] hubs… they need specialty clinics and referral centres for people to be sent where they can be educated and make better choices (25 pharmacist mid career). | A lot of this kind of care you don’t actually need a one-on-one with the family doctor or nurse practitioner [and] can be provided by other members of the team. Your nurse could do a lot of the counselling, if there’s medication, a pharmacist in-house could do a lot of the counselling related to that. Many teams have physiotherapists or kinesiologists if exercise is part of the routine. So there’s diversity from the lens of different providers, making sure that you’re getting the right care at the right time (19 healthcare executive late career).  Portioning the level of care to the need could be done much better… if you have medical doctors doing things that nurses could do or 811 phone lines could do or other people could do then that’s not best use of primary care to start off with (22 executive research late career).  We really should have a primary care hub in every community… that’s gonna require a lot of capital support, maybe moving folks away from their solo practices into a group setting, a deeper dive in the community to look at the conditions at the highest concern… You’re saving the system a ton of money because it is all about prevention and maintenance or managing the chronic diseases that you have. But we just don’t do that because we’re very acute centric… there is definitely this realization that there is really no healthcare for everybody in the country. All the Canada Health Act does is guarantees you access to hospital care and physician care. Great, but if you can’t even access a physician then it’s a problem… it isn’t really about the right provider at the right time and it may not be a doctor, it may be a nurse practitioner, anybody working to their full scope, there’s enough work to go around but we just need to enable that… hopefully in our lifetime, people would then identify with a primary care hub as their patient medical home… but that requires political will and a lot of investments (19 healthcare executive late career). |
| Increase number of healthcare professionals for OA |  | We haven’t trained enough doctors and… it doesn’t make sense to train enough doctors. More primary care nurses, extended care nurses, nurse practitioners would be part of the solution. So people get knee pain and then they can talk to a nurse (22 executive research late career) |
| Increase availability of diverse (i.e., gender, ethnicity) healthcare professionals |  | We need more diverse healthcare providers, physicians, nurses… even at the policy-level we need a more diverse representation of people that [are] just like the population of Nova Scotia, not just that entry-level but all the way up to decision-making table. So that we have more diverse perspectives from the bottom all the way up (14 government policymaker mid career). |
| National OA strategy and coordinated pathway for OA care | Canada and the provinces need a much better developed OA strategy and that has to cross from government level to primary care down to the first line clinicians… it needs to be a full drawn-out pathway for a client to follow. That’s a lot of levels of government and working together... At a high level there needs to be the funding and the middle level the Health Authorities should be working together to develop this pathway…We have other medical pathways and they’re not perfect but at least it’s a start; [if you] have a stroke… there’s a pathway I’m gonna follow…there’s not a true OA pathway that works across our province or other provinces… there’s Canadian guidelines to best stroke care. Is there Canadian guidelines for best OA care in Canada and is that followed? Not to my knowledge (06 occupational therapist mid career).  I think technology isn’t being used to its full advantage in community pharmacy... when someone walks into a pharmacy I should be able to pull up their profile and have a really good idea of their health just by looking at that from previous documentation or assessments, but I typically do not. So every time you see somebody you’re just redoing work that should have just be documented and instead of updating you’re redoing. So I think technology in the next 10 years is gonna be huge in pharmacy and those who don’t use it will probably not be able to keep up… having good documentation of their conditions so when they come in we can have a note saying this is a condition we treated like on this date and how are you doing today? … so there’s continuity of care (26 pharmacist mid career).  When you think about screening, often things that get captured well are part of our Ministry-funded screening because providers are incentivised to do it… that’s not to say to pay doctors to talk about OA but just like we’ve got routine diabetic screening now because it’s way better to screen people early and catch it than to deal with all these diabetics that get missed and are huge burden to the healthcare system… we’ve got a diabetes strategy, we’ve got heart disease prevention strategy. We’ve got tools for practitioners and recommended screening guidelines. We don’t have any of that for OA… whether there’s screening or even screening questionnaires to ask people about how their mobility is going can be helpful… Try and build into the screening that already does happen for people… if you look at osteoporosis, we’re supposed to say okay, over 55 you get the [screening test] but based on different risk factors you might revisit this sooner. If we have to add an extension to say what about OA risk? Here are a few red flags that they might have mild to moderate OA and recommended starting point… there are some algorithms to say like, [if you’re moderate] you should be doing NSAID plus injection, if you’re severe, you should be doing x, y, z, referral to ortho (23 nurse practitioner early career). | [The] billing code for clinicians [needs] to be recognized [and] that this is an important use of time [to discuss OA with patients] and they get obviously compensated for it. There needs to be the digital and maybe physical infrastructure to support people getting the right tools, the right information, the right people. Some of it comes under funding but a lot of it comes down to coordination and getting all the different parts of the ecosystem to be connected and talking to each other… how do we look at all of the different resources that are available to the patients and making sure that’s front and centre. That probably needs an overlay that’s coordinated… you can understand what different programs they offer and how they can benefit people in different ways and then you understand how to get the right people into the right programs (31 executive charity late career).  One of the pieces that we know can be a challenge is around triaging patients appropriately. How do we understand when a patient needs urgent care or not and how do we triage patients to make sure that they get to the right care? How do we raise awareness of all the programs that are available first, in Ontario and Canada? That right now is a gap. As far as I know, there’s not an easy place to go for people to understand all of the different places that could be supporting them in their communities with their particular condition. And then the other piece around having a navigator model that helps support that and helps get people to the right places. You could also have advanced trained physiotherapist that could play that role if somebody has some early signs and symptoms that yet doesn’t know what to do next or where to go next; trying to make sure that they get early access to different things whether it’s an external programs, access to individual self-management things they could be doing. I think there’s definitely a role to play around better communication, coordination of the system as is… trying to establish a single source of truth for all of the related MSK supports that people could be accessing… there’s different models you can explore. But the crux of it is that you know you can get the right services to the right people when they need it. That’s the goal (31 executive charity late career). |
| Develop healthcare policy to address the needs of diverse women | I think prioritizing care for both women and diverse women. Recognizing that these patients may require more resources and or different types of resources and or the providers may need more training and sometimes even more time allocated for the assessments. I think it is just that recognition that these are patients in the system that aren’t getting equitable care (12 physiotherapist late career). | There needs to be an overarching equity and diversity and inclusion policy which also covers cultural competency, cultural appropriateness, cultural sensitivity and safety, which would then have more subsets of how to reach specific populations and specific healthcare settings (14 government policymaker mid career). |
